# Energetics of interactions in the solid state of 2-hydroxy-8-*X*-quinoline derivatives (*X* = Cl, Br, I, S-Ph): comparison of Hirshfeld atom, X-ray wavefunction and multipole refinements

**DOI:** 10.1107/S2052252519007358

**Published:** 2019-07-15

**Authors:** Magdalena Woinska, Monika Wanat, Przemyslaw Taciak, Tomasz Pawinski, Wladek Minor, Krzysztof Wozniak

**Affiliations:** a Biological and Chemical Research Centre, Department of Chemistry, University of Warsaw, Zwirki i Wigury 101, Warszawa 02-089, Poland; b Department of Molecular Physiology and Biological Physics, University of Virginia, Charlottesville, VA, USA; c College of Inter-Faculty Individual Studies in Mathematics and Natural Sciences (MISMaP), University of Warsaw, Stefana Banacha 2C, Warszawa 02-097, Poland; d Department of Drug Chemistry, Faculty of Pharmacy, Medicinal University of Warsaw, Stefana Banacha 1, Warszawa 02-091, Poland; e Department of Pharmacodynamics, Centre of Preclinical Research and Technology, Faculty of Pharmacy, Medical University of Warsaw, Stefana Banacha 1, Warszawa 02-091, Poland; f Department of Chemistry, University of Warsaw, Pasteura 1, Warszawa 02-093, Poland

**Keywords:** multipole refinement, X-ray wavefunction refinement, anharmonic thermal motion, high-resolution X-ray crystallography, energy calculations, molecular crystals, intermolecular interactions, charge, spin and momentum densities

## Abstract

In this work, two methods of high-resolution X-ray data refinement: multipole refinement (MM) and Hirshfeld atom refinement (HAR) – together with X-ray wavefunction refinement (XWR) – are applied to investigate the refinement of positions and anisotropic thermal motion of hydrogen atoms, experiment-based reconstruction of electron density, refinement of anharmonic thermal vibrations, as well as the effects of excluding the weakest reflections in the refinement. The study is based on X-ray data sets of varying quality collected for the crystals of four quinoline derivatives with Cl, Br, I atoms and the -S-Ph group as substituents. Energetic investigations are performed, comprising the calculation of the energy of intermolecular interactions, cohesive and geometrical relaxation energy. The results obtained for experimentally derived structures are verified against the values calculated for structures optimized using dispersion-corrected periodic density functional theory. For the high-quality data sets (the Cl and -S-Ph compounds), both MM and XWR could be successfully used to refine the atomic displacement parameters and the positions of hydrogen atoms; however, the bond lengths obtained with XWR were more precise and closer to the theoretical values. In the application to the more challenging data sets (the Br and I compounds), only XWR enabled free refinement of hydrogen atom geometrical parameters, nevertheless, the results clearly showed poor data quality. For both refinement methods, the energy values (intermolecular interactions, cohesive and relaxation) calculated for the experimental structures were in similar agreement with the values associated with the optimized structures – the most significant divergences were observed when experimental geometries were biased by poor data quality. XWR was found to be more robust in avoiding incorrect distortions of the reconstructed electron density as a result of data quality issues. Based on the problem of anharmonic thermal motion refinement, this study reveals that for the most correct interpretation of the obtained results, it is necessary to use the complete data set, including the weak reflections in order to draw conclusions.

## Abstract 2

Two methods of high-resolution X-ray data refinement, multipole refinement and Hirshfeld atom refinement together with X-ray wavefunction refinement are compared using data sets collected for the crystals of four quinoline derivatives containing Cl, Br and I atoms and the -S-Ph group. This study highlights the importance of including the full set of reflections in the refinement to facilitate the correct interpretation of the results, which is shown using the example of anharmonic thermal motion refinement.

## 1. Introduction   

Quinoline is a planar heterocyclic compound consisting of a benzene ring fused with a pyridine ring, with the nitro­gen atom at position 1. Its derivatives are not only common among many natural compounds, but are also utilized by many branches of industry, in particular, they are very important in the synthesis of pharmaceutically active compounds (Solomon & Lee, 2011 ▸). Quinoline is a structural subunit that is particularly efficient in the design of multiple types of drugs and, therefore, quinoline-containing molecules are widely used as initial compounds for the synthesis of substances of biological importance. A vital example of the therapeutic application potential of quinoline-based substances are antimalarial drugs such as quinine, chloro­quine, mefloquine and amodiaquine (Kaur *et al.*, 2010 ▸). Moreover, quinoline derivatives have a broader spectrum of antimicrobial properties, such as antifungal activity (Musiol *et al.*, 2010 ▸; Teichert *et al.*, 2008 ▸; Zhao *et al.*, 1998 ▸; Oliva *et al.*, 2003 ▸; Höfle & Kunze, 2008 ▸; Kunze *et al.*, 1987 ▸; Höfle & Irschik, 2008 ▸; Kitagawa & Tamura, 2008 ▸), *e.g.* dictamine, halpopine, kolbisine, aurachin C and aurachin D; and antibacterial activity (Teichert *et al.*, 2008 ▸; Solomon & Lee, 2009 ▸). Quinoline compounds have also been subject to multiple research studies due to their anticancer effects (Solomon & Lee, 2011 ▸, 2009 ▸), which is conditioned by their activity against many anticancer drug targets (Solomon & Lee, 2009 ▸). Quinoline, as a result of its universal synthetic applications and owing to the fact that it shows a broad spectrum of pharmacological activity, belongs to the group of substances described as generic drug-like motifs (Musiol *et al.*, 2011 ▸). These are, in turn, very versatile so-called privileged motifs (Evans *et al.*, 1988 ▸) (molecular fragments that facilitate ligand binding to a certain type of receptor), which are very important starting points in the process of drug design. Thus, it is necessary to thoroughly investigate such unique molecular frameworks in order to elucidate the connection between their structure and the specific interactions they mediate. In particular, it is crucial to establish the way in which substituents with certain properties influence the qualities of the drug-like motif and change its therapeutic potential.

**Figure 1:**
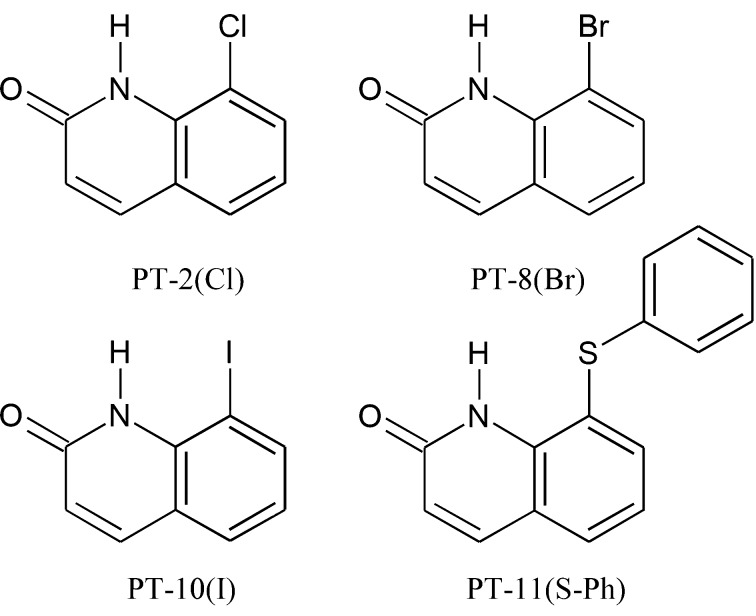
Structures of the quinoline derivatives.

In this work, we present a high-resolution X-ray diffraction study of four derivatives of 2-hydroxy­quinoline (Fig. 1 ▸) substituted at position 8 with a halogen atom (Cl, Br, I) or a phenyl­sulfanyl moiety, referred to as PT-2(Cl), PT-8(Br), PT-10(I) and PT-11(S-Ph), respectively. High-resolution X-ray diffraction experiments are particularly valuable in understanding how the properties of a given molecule alter for different substituents, as this technique allows us to investigate molecular electron density and gain insight into subtle changes in its distribution both qualitatively and quantitatively. Since the effects related to the electron density distribution of the molecule in a crystal are analogous to those of the electron density distribution of the ligand in the active site of an enzyme, high-resolution X-ray diffraction experiments can help us understand the interactions of the therapeutic agent with the target receptor (Malińska *et al.*, 2014 ▸). We investigate two different methods of X-ray data refinement: the multipole refinement (MM) (Hansen & Coppens, 1978 ▸) and X-ray wavefunction refinement (XWR) (Grabowsky *et al.*, 2012 ▸; Chęcińska *et al.*, 2013 ▸) with its first step of structural refinement, HAR (Capelli *et al.*, 2014 ▸).

The first method is a popular and widely applied technique with certain well known limitations, whereas the latter is an alternative solution, which, according to the previous comparative studies (Bučinský *et al.*, 2016 ▸; Bytheway *et al.*, 2002*a* ▸,*b* ▸; Chęcińska *et al.*, 2013 ▸; Dittrich & Jayatilaka, 2012 ▸; Dittrich *et al.*, 2012 ▸; Grabowsky *et al.*, 2013 ▸; Hickstein *et al.*, 2013 ▸; Hudák *et al.*, 2010 ▸; Jayatilaka & Dittrich, 2008 ▸; Jayatilaka *et al.*, 2009 ▸; Woińska *et al.*, 2014 ▸, 2017 ▸), is able to completely overcome or alleviate the flaws of MM. Moreover, MM is based on a complex optimization scheme that requires numerous choices to be made by the user, unlike XWR which does not involve much user intervention.

In this study, two versions of each refinement were performed: based on the full set of collected data, and after rejection of the weakest reflections [the |*F*_o_| ≥ 2σ(|*F*_o_|) condition]. As previously described in the literature (Henn & Meindl, 2014 ▸; Hirshfeld & Rabinovich, 1973 ▸), applying an intensity cut-off is not methodologically correct, since it leads to inconsistencies of residuals with Gaussian distribution, which is a prerequisite for the validity of model parameters and their standard uncertainties obtained from a least-squares procedure. One of the goals of our work was to investigate the influence of intensity cut-off on the effects under analysis, such as anharmonic thermal motion.

Our experimental results are supplemented with exhaustive theoretical calculations, which establish the relation between the structure, the interactions of the molecules and the properties of the crystal. To the best of our knowledge, this work constitutes the first comparison between the energies of interactions in the solid state for molecular geometries obtained from MM and XWR, with periodic theoretical geometry optimization used as the reference point. Aside from being a study of the properties of a molecular framework characterized by well recognized pharmaceutical potential, our work is also a comparison of the capabilities of two methods of X-ray data processing. However, this work is not the first case of XWR performed with refinement of the parameters of anharmonic thermal vibations. The very first study was carried out on the X-ray data set of the compound *N*-(5-acetyl-4-methyl­thia­zol-2-yl)-3-(2,4-di­chloro­phenyl)-acryl­amide, and the results, due to the long process of error correction involving the authors of various software and preceding studies, still remain unpublished (Krzeszczakowska *et al.*, unpublished work ▸).

## 2. Experimental methods and computational details   

### 2.1. Synthesis   

In general, syntheses were performed by the acyl­ation of the substituent aniline at position 2 with cinnamoyl chloride to obtain cinnamamide, which was converted to the quinolin-2-one derivative using aluminium chloride. (Pearson, 2008 ▸) 8-phenyl­sulfanyl-1*H*-quinolin-2-one was obtained by an Ullman-like reaction with thio­phenol (Kwong & Buchwald, 2002 ▸).

#### 2.1.1. *N*-(2-halophenyl)-3-phenyl­acryl­amide   

To the stirred cooled (0°C) solution of 2-haloaniline (0.01 mol) and pyridine (0.02 mol) in di­chloro­methane (100 ml), cinnamoyl chloride (0.01 mol) in di­chloro­methane (*ca* 5 ml) was added slowly. The resulting mixture was stirred overnight, washed with a *ca* 5% aqueous NaHCO_3_ solution, water and brine. The organic layer was dried over anhydrous Na_2_SO_4_ and concentrated *in vacuo* to afford the title compound as a solid, with a yield of 91.0–93.5%. The halogens used were chlorine, bromine and iodine for PT-2(Cl), PT-8(Br) and PT-10(I), respectively (Fig. 1 ▸).

#### 2.1.2. 8-halo-1*H*-quinolin-2-one   

To the hot solution of *N*-(2-halophenyl)-3-phenyl­acryl­amide (0.0075 mol) in chloro­benzene (12 ml), anhydrous aluminium chloride (0.0225 mol) was slowly added. The reaction mixture was refluxed for 3 h, cooled and poured onto crushed ice. The solid was filtered, washed with water and cold ethanol, and dried to afford the title compound as a yellow to light-red solid, yield of 55.2–67.1%. The halogens used were chlorine, bromine and iodine for PT-2(Cl), PT-8(Br) and PT-10(I), respectively (Fig. 1 ▸).

#### 2.1.3. 8-phenyl­sulfanyl-1*H*-quinolin-2-one [PT-11(S-Ph)]   

A mixture of 8-iodo-1*H*-quinolin-2-one (271 mg, 1 mmol), appropriately substituted thio­phenol (1 mmol), CuI (9.5 mg, 0.05 mmol), K_2_CO_3_ (276 mg, 2 mmol), ethyl­ene glycol (0.13 ml) and iso­propanol (3 ml) was stirred in reflux under an inert atmosphere of argon for 24 h. The reaction mixture was then poured into water (*ca* 10 ml) with 15% aqueous ammonia (*ca* 3 ml); di­chloro­methane was added and the organic layer was separated. The aqueous layer was extracted twice with di­chloro­methane, the organic phases were combined, washed with brine, dried over anhydrous Na_2_SO_4_ and purified using flash chromatography on silica gel (di­chloro­methane, then 10% acetone in di­chloro­methane) to afford the title compound as a solid, with a yield of 92.4% (Fig. 1 ▸).

### 2.2. Crystallization   

Crystals of all the investigated compounds were grown from a methanol solution. A mass of 1 mg of each compound was dissolved in 2 ml of methanol; small flasks were closed by silicon plugs with thin needles to allow the solvent to evaporate slowly. After 4–6 weeks, crystals started to appear in all flasks.

### 2.3. X-ray data collection   

High-resolution X-ray experiments were carried out on single crystals at 90 K. The crystals were mounted with Paratone N oil on MiTeGen micromounts. PT-11(S-Ph) was measured on a Bruker AXS Kappa APEX II Ultra diffractometer equipped with a TXS rotating anode (Mo *K*α radiation, λ = 0.71073 Å), four-circle goniometer, multilayer optics and an Oxford Cryosystems nitro­gen gas-flow device (700 Series Cryostream). Lattice parameters and integrated intensities of Bragg reflections were obtained with the *APEX2* software (Bruker, 2013 ▸), which was also used for the application of Lorentz, polarization and oblique incidence corrections. The multiscan absorption correction was performed using *SADABS* (Sheldrick, 1996 ▸). The measurements for PT-2(Cl), PT-8(Br) and PT-10(I) were performed on the Agilent Technologies SuperNova Dual Source diffractometer equipped with a Mo *K*α radiation source (λ = 0.71073 Å), graphite monochromator, Atlas detector and Oxford Cryosystems nitro­gen gas-flow device (Cobra Plus). Unit-cell parameter determination and data reduction were accomplished with the *CrysAlisRED* program (Oxford Diffraction Ltd, 2008 ▸). Numerical absorption correction based on Gaussian integration over a multifaceted crystal model and empirical absorption correction using spherical harmonics, implemented in the *SCALE3 ABSPACK* scaling algorithm were applied (Oxford Diffraction Ltd, 2008 ▸). For all compounds, data scaling and merging were achieved with *SORTAV* (Blessing, 1987 ▸, 1989 ▸, 1995 ▸, 1997 ▸). The data collection and reduction parameters can be found in Table 1 ▸.

### 2.4. Structure determination and further refinement (multipole refinement, Hirshfeld atom refinement and X-ray wavefunction refinement)   

Structure solution was performed using direct methods implemented in the *SHELXS* program (Sheldrick, 2008 ▸). Structural refinement was carried out with the *SHELXL* program (Sheldrick, 2008 ▸) within the independent atom model (IAM) formalism. Atomic displacement parameters (ADPs) were refined for non-hydrogen atoms, whereas for hydrogen atoms, isotropic refinement with geometrically determined positions was performed. The resulting structure was used as the starting model for the subsequent MM, HAR and XWR. In each case, refinement was based on *F* and performed for two different sets of reflections: (i) all the reflections up to the maximal resolution with no intensity restriction and (ii) only reflections fulfilling the |*F*_o_| ≥ 2σ(|*F*_o_|) condition. Statistical weights were applied.

#### 2.4.1. Multipole refinement   

Multipole refinement was performed with the *MoPro* software (Jelsch *et al.*, 2005 ▸) starting from the IAM geometry. The local atomic symmetries and initial multipole parameters were transferred from the UBDB2011 data bank with the *LSDB* program (Jarzembska & Dominiak, 2012 ▸).

For all the analysed compounds, hydrogen atoms were shifted to the average distances from neutron diffraction experiments (Allen & Bruno, 2010 ▸), and their positions were restrained to these distances. In addition, for the high-quality structures of PT-11(S-Ph) and PT-2(Cl), an alternative version of MM with hydrogen positions freely refined was performed (freeXH). In each case, the refinement started with adjusting the scale factor, followed by simultaneous refinement of the scale factor and positions of all atoms (hydrogen atoms treated as already described). Finally, the overall structure scaling factor, atomic positions and ADPs of the non-hydrogen atoms were refined together. At this stage, the anisotropic thermal motions of the hydrogen atoms were treated in two different ways depending on data quality: for PT-11(S-Ph) and PT-2(Cl), ADPs were refined, whereas for PT-8(Br) and PT-10(I), ADPs were estimated with the *SHADE2* server (Madsen, 2006 ▸). Next, multipole parameters were refined by a gradual increase of the level of multipole expansion from monopoles up to hexadecapoles for non-hydrogen atoms and up to quadrupoles for hydrogen atoms (only bond-directed ones). Then, κ parameters for non-hydrogen atoms were refined and finally, all parameters were refined simultaneously. The next step differed for each structure:

(i) For PT-11(S-Ph), the procedure of gradually releasing multipole parameters followed by refinement of κ parameters for non-hydrogen atoms and simultaneous refinement of all the parameters was repeated twice. Then, multipole parameters were allowed to vary with the chosen standard deviation and finally, they were refined with no constraints or restraints.

(ii) In the case of PT-2(Cl), only refinement of κ parameters for non-hydrogen atoms and simultaneous refinement of all the parameters was repeated twice and finally, multipole parameters were restrained to the values from the databank with the selected standard deviation.

(iii) For PT-8(Br) and PT-10(I), the procedure of gradually releasing multipole parameters was followed by estimating hydrogen ADPs with *SHADE2*. These two steps had to be repeated twice until convergence of the ADP values was reached. Next, refinement of κ parameters for non-hydrogen atoms and simultaneous refinement of all the parameters followed by the employment of *SHADE2* for modelling hydrogen thermal motions were performed twice to obtain convergent values of hydrogen ADPs.

The models obtained for each of the compounds according to the described strategies were used to refine anharmonic thermal motions of S, Cl, Br and I atoms. At the last stage, all the previously released parameters were refined again together with the third-order Gram–Charlier coefficients and, finally, third- and fourth-order Gram–Charlier coefficients.

#### 2.4.2. Hirshfeld atom refinement and X-ray wavefunction refinement   

HAR (Jayatilaka & Dittrich, 2008 ▸; Capelli *et al.*, 2014 ▸) and the subsequent X-ray constrained wavefunction fitting, which is the second step of the XWR (Grabowsky *et al.*, 2012 ▸; Chęcińska *et al.*, 2013 ▸) procedure, were performed with the *TONTO* program (Jayatilaka & Grimwood, 2003 ▸; Tonto 3.6.1 github v. v3.2.0–372-g3153a7c). During molecular wavefunction calculations in HAR, in order to include interactions with the crystal environment, the central molecule was embedded in a cluster of atomic charges and dipoles for all the surrounding molecules with at least one atom within 8 Å from the central molecule. The wavefunction was obtained in the course of DFT/BLYP (Hohenberg & Kohn, 1964 ▸; Becke, 1993 ▸; Lee *et al.*, 1988 ▸) calculations with the cc-pVDZ (Dunning, 1989 ▸) basis set used for all chemical elements except iodine, for which the DZP (Barros *et al.*, 2010 ▸) basis set was applied. During HAR, all atomic positions and ADPs were refined without any constraints or restraints. Subsequently, orbital coefficients of the wavefunction were adjusted to minimize the Lagrangian in the X-ray constrained wavefunction fitting procedure in order to obtain electron density reconstructed from experimental structure factors. The value of the λ parameter, controlling the experimental contribution in the molecular wavefunction, was increased as long as calculations of the wavefunction converged in the following sequence of λ steps: 0.1, 0.05, 0.01, 0.005. The final values of λ (λ_max_) and χ^2^ achieved are given in Table 2 ▸.

### 2.5. Theoretical calculations and evaluation of electron density properties   

Theoretical calculations were performed with the *CRYSTAL*09 (Dovesi *et al.*, 2009 ▸) program package at the DFT/B3LYP level of theory (Becke, 1988 ▸; Lee *et al.*, 1988 ▸) with cc-pVDZ basis set (DZP for iodine). Grimme D2 dispersion correction was applied (Civalleri *et al.*, 2008 ▸; Grimme, 2006 ▸). Geometry optimization with periodic boundary conditions and fixed unit-cell parameters was carried out for each structure and used as a benchmark for the results obtained for the experimental geometries resulting from various refinement methods. Energetic calculations included cohesive energy of the crystals, relaxation energy and energy of dimer interactions. Moreover, experimentally reconstructed electron density was evaluated in the course of the analysis of critical points and bond paths using the *VMoPro* module of the *MoPro* package. The cohesive energy was calculated as the difference between crystal lattice energy per molecule and the molecular energy of a molecule in the gas phase, as described in the literature (Civalleri *et al.*, 2008 ▸). BSSE was estimated through the counterpoize method (Boys & Bernardi, 1970 ▸) with ghost atoms selected within a distance of 5 Å from the central molecule. Geometric relaxation energy (further shortened to relaxation energy) was calculated as the difference between the energy of an isolated molecule with the crystal geometry and the energy of a molecule with the gas phase geometry. Energies of interactions of all dimers in the considered crystal structures were calculated with the *Gaussian*09 program (Frisch *et al.*, 2009 ▸) using the same quantum mechanical method and level of theory as in the case of the periodic calculations, including BSSE and D2 dispersion correction.

## 3. Results and discussion   

### 3.1. General evaluation of data quality   

The considered data sets vary in quality [high-quality data sets PT-11(S-Ph) and PT-2(Cl) versus low-quality data sets PT-8(Br) and PT-10(I)]; nevertheless, *R* and *wR* obtained in the course of HAR/XWR/MM refinement in all cases are low (see Tables 2 ▸ and Table S1 of the supporting information). Particularly high values of *wR*, as compared with *R*, are observed in the case of IAM refinement – it is observed that the data sets characterized by lower absorption [PT-11(S-Ph) and PT-2(Cl)] yield outstandingly high values of *wR*. Moreover, the goodness-of-fit values are between 1 and 2 and are surprisingly closer to 1 for the lower quality data sets. Therefore, the described statistical parameters do not reflect the visibly varying quality of the data sets. For the good-quality data sets PT-11(S-Ph) and PT-2(Cl), the values of the maximum positive and negative residual densities are much lower than this in the case of the remaining structures (Tables 1 ▸, 2 ▸ and S1). For PT-11(S-Ph), residual density values are low both in the case of HAR/XWR and MM. In the case of PT-2(Cl), HAR/XWR still results in a relatively narrow range of residual density between −0.5 and 0.5 e Å^−3^; however, in the case of MM, the maxima and minima are significant, irrespective of the applied data intensity cut-off. Fractal dimension plots for PT-11(S-Ph) and PT-2(Cl) are not narrow and the majority are characterized by certain imperfections, such as deviations from the parabolic shape (non-Gaussian character of experimental errors) or the presence of a shoulder in the region of positive or negative values. These phenomena are also reflected by residual density maps, which are often not perfect, although are still acceptable. Residual density maps for PT-11(S-Ph) and PT-2(Cl) obtained with all the applied methods display correct features. The good quality of PT-11(S-Ph) and PT-2(Cl) can also be confirmed by successful refinement of H ADPs both with HAR and MM, quite good values of C—H and N—H bond lengths refined with HAR (see Section 3.5) and the fact that it is also possible to refine hydrogen positions with MM, although the obtained bond lengths (particularly N—H) are often visibly further from the theoretical values from *ab initio* calculations than in the case of HAR. PT-8(Br) and PT-10(I) are poor-quality data sets, highlighted by their extremely wide ranges of residual density (Tables 1 ▸, 2 ▸ and S1), areas of very high values of residual density in the residual density maps (especially around Br and I) and very wide fractal dimension plots which often diverge from the parabolic shape or have a shoulder [on the side of negative values in the case of MM refinement of PT-8(Br) and also harmonic HAR and XWR of PT-10(I)]. The deterioration of MM deformation density maps is significant not only around Br and I, but also in the vicinity of the quinoline ring. In the case of PT-8(Br) and PT-10(I), HAR-derived H ADPs, as well as C—H and N—H bond lengths refined with HAR deteriorate significantly compared with the structures of PT-11(S-Ph) and PT-2(Cl) (see Section 3.5). Multipole refinement of ADPs and positions of hydrogen atoms for the Br and I derivatives is, of course, impossible.

### 3.2. Comparison of HAR/XWR and MM   

**Figure 2:**
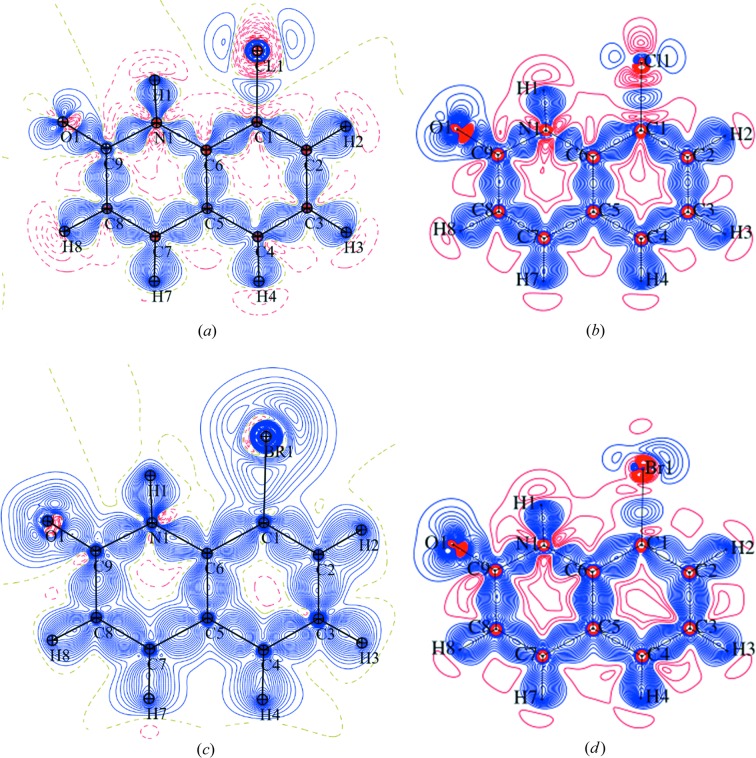
Deformation density maps for high-quality [PT-2(Cl)] and low-quality [PT-8(Br)] data without intensity cut-off. Contour level: 0.05 e Å^−3^. Colours: blue – positive, red – negative. (*a*) PT-2(Cl), MM (freeXH), harmonic. (*b*) PT-2(Cl), XCW, harmonic. (*c*) PT-8(Br), MM, harmonic. (*d*) PT-8(Br), XCW, harmonic.

XWR usually reduces the values of residual density compared with HAR – this effect is quite subtle in the case of PT-2(Cl), PT-11(S-Ph) and PT-8(Br) [the residual density ranges comparison for harmonic HAR/XWR is the following (units: e Å^−3^): PT-11(S-Ph): −0.18 to 0.34/−0.18 to 0.27; PT-2(Cl): −0.49 to 0.35/−0.46 to 0.39, PT-8(Br): −1.23 to 1.13/−1.20 to 1.17], but is found to be significant for PT-10(I) in the case of negative residual density [the residual density ranges comparison for harmonic HAR/XWR is the following (units: e Å^−3^): PT-10(I): −1.54 to 0.71/−1.14 to 0.70]. This may be an indication that the departure from the quantum minimum of energy is too high and that considerable experimental noise starts to visibly influence the fitted model of electron density. Deformation density maps (Fig. 2 and Figs. S33–S50 of the supporting information) are obtained with HAR picture theoretical electron density and are thus free from obviously incorrect features.

For the remaining methods, the quality of deformation density varies with the analysed compound. For PT-2(Cl), both XWR and MM (Fig. 2 ▸) result in a model correctly describing electron density in the regions of bonds, free electron pairs in the case of the oxygen atom, as well as in the vicinity of C and N. In particular, regions of negative electron density around C and N nuclei are observed not only in the XWR deformation density, but also for MM (with rare exceptions), which is uncommon for this refinement method. However, the departure from the theoretical distribution of electron density around Cl is considerable for MM, whereas XWR results in electron density very closely matching the theoretical one, which is expected behaviour since the latter method uses theoretical electron density as the initial model and the former starts from a very coarse-grained model of spherical atoms. When one considers the structure of PT-11(S-Ph), it can be noticed that for MM, negative deformation density regions are present only around very few non-hydrogen nuclei. Moreover, for the oxygen atoms in some MM refinement cases, lone electron pairs are characterized by a certain asymmetry of electron density distribution. Nevertheless, overall both XWR and MM correctly describe the features of electron density and correspond well with the theoretical distribution of electron density of the sulfur atom. For PT-8(Br), electron density in the vicinity of O and Br atoms is substantially deformed in the case of MM, whereas XWR yields only small deformations (Fig. 2 ▸). Certain deformations within the quinoline rings are also noticeable for both refinement techniques. The deformation density distribution obtained with XWR resembles the theoretical distribution much more closely (particularly around O and Br atoms) than in the case of MM. Moreover, regions of negative electron density around the non-hydrogen nuclei can be obtained only with XWR, as it is also the case with the compound PT-10(I). As expected for PT-10(I), the distortions visible on the maps of deformation density are especially significant. In the case of XWR, certain departures from the theoretical distribution are present in the vicinity of iodine and also minor deformations can be observed in the region of the quinoline ring (the deformation density isolines diverge from the symmetry of quinoline rings), whereas the density of the O atom is modelled correctly. In MM, in turn, significant problems with modelling electron density arise, the consequences of which are considerable distortions around all the atoms and bonds (the shape of deformation density diverges from the shape of the rings, positive deformation density is distorted and shifted from the vicinity of the nuclei or the bonds), with particularly deformed density of iodine largely manifesting itself on the density map.

In general, XWR provides better quality results in terms of the properties of electron density than MM. Residual density is flatter [Figs. 3 ▸ and S15–S32; for comparison the residual density ranges comparison for harmonic XWR/MM is the following (units: Å): PT-11(S-Ph): −0.18 to 0.27/−0.21 to 0.32; PT-2(Cl): −0.46 to 0.39/−0.62 to 0.52; PT-8(Br): −1.20 to 1.17/−1.92 to 1.04; PT-10(I): −1.14 to 0.70/−1.95 to 1.82], fractal dimension plots (Figs. S1–S14) are narrower and more symmetrical, particularly in the case of PT-8(Br) and PT-10(I) the difference is huge.

The analysis of X—H bond lengths (Tables S3–S10) shows that, in general, the values obtained with HAR are not as close to the optimized ones as the mean neutron bond lengths. In the case of PT-11(S-Ph) and PT-2(Cl), for which MM also allowed unconstrained refinement of hydrogen positions, the bond-length values obtained with MM are slightly further from the theoretical ones compared with those from HAR, they are also characterized by lower precision. However, H ADPs, which could be refined with both refinement methods for PT-11(S-Ph) and PT-2(Cl) only, are of similar quality for HAR and MM. For PT-8(Br), only HAR enabled refinement of H ADPs; however, as might be expected, the shapes of the resulting ellipsoids are much more distorted compared with those estimated with *SHADE2*. For PT-10(I), refinement of H ADPs was unsuccessful.

We analysed the similarity index (*S*) (Spackman, 1992 ▸) between H ADPs obtained with HAR and MM (Tables 5 and S2). The level of similarity between H ADPs was visibly higher for the good-quality data sets PT-2(Cl) and PT-11(S-Ph) (ranging between 2.31–3.84% and 2.65–2.85%) than for PT-8(Br) and PT-10(I) (values between 11.79–12.93% and 15.35–27.09%). In the case of PT-10(I), the H atoms with non-positive definite (NPD) thermal ellipsoids were not included in the considerations. Bond angles not involving H atoms are in very good agreement with the values from geometry optimization for all types of refinement. The angles involving H atoms diverge more from the geometry-optimized values, with the level of discrepancy dependent on the data quality, and are very similar for HAR and MM. In the case of PT-11(S-Ph), the discrepancies are very small (within 0–1° range). For PT-2(Cl) and PT-8(Br) the divergence is slightly higher (within 0–3° range). In the case of PT-10(I), differences with the theoretical values of up to 7° are observed.

### 3.3. Anharmonicity refinement   

In the case of each structure, not only was harmonic refinement carried out but also refinement of anharmonic thermal motions of the heavy atoms (S, Cl, Br and I) was performed. Two types of anharmonic thermal motion refinement were carried out: one up to third order and one up to fourth order of anharmonicity. Minimal resolution of diffraction data necessary to refine each anharmonicity order for the specified atoms was estimated with *XDPROP* (Kuhs, 1992 ▸), and together with the obtained experimental data resolution it can be found in Table 3 ▸. In order to confirm the presence of anharmonic motion, probability density function was also analysed [see Table 4 ▸ for visualization of probability density functions for the case of PT-2(Cl) and Figs. S62–S65 for the all the crystal structures]. For PT-11(S-Ph), none of the minimal resolution limits have been achieved in the experiments performed, however, there is a slight reduction in residual density [(ρ_min_, ρ_max_) from (−0.18, 0.34) e Å^−3^ to (−0.16, 0.19) e Å^−3^ in the case of HAR and from (−0.21, 0.32) e Å^−3^ to (−0.20, 0.18) e Å^−3^ in the case of MM] after the third-order anharmonicity refinement which is probably because of artificial error absorption by additional parameters introduced into the model. In HAR for PT-11(S-Ph), none of the Gram–Charlier coefficients that were obtained differed significantly from zero (see Tables S19–S22 and Table 3 ▸), which reflects well on this refinement method and which is not the case for MM. The following number of Gram–Charlier coefficients that are significantly different from 0 for the MM [MM(freeXH)] refinement without intensity cut-off: third order 3[4], anharmonic (*n* = 3); third order 4 [4], anharmonic (*n* = 4); fourth order 3 [0], anharmonic (*n* = 4). Moreover, in the case of MM and MM(freeXH) refinement of anharmonic thermal motion of S up to the fourth order, the probability density function had a negative value in certain fragments of space around the S atom, which is an indicator of the absence of anharmonic vibrations. For PT-2(Cl), the experimental data resolution is certainly sufficient to refine third-order anharmonicity for Cl and close to sufficient for the fourth order. MM brings improvement in the case of refinement up to the third order (as well as up to the fourth order), but HAR results in the most outstanding improvement after refinement up to fourth-order anharmonicity – residual density around the Cl atom disappears and its range in the whole unit cell becomes considerably narrower (with a narrower fractal dimension plot). In the case of both methods, there are values of Gram–Charlier coefficients significantly different than 0. Their values, compared between the discussed refinement methods are mostly not equal within one standard deviation, although they usually have the same sign. Nevertheless, in the case of refinement of anharmonic thermal motion up to the fourth order, all of the methods yield probability density functions with negative values in a considerable volume of space close to the Cl atom (in the case of MM this volume is much bigger). Moreover, in the case of HAR performed on the full data set, negative values of the probability density function also appear in the case of refinement of anharmonic motion up to the third order only.

For PT-8(Br), the experimental data resolution is enough to refine the third-order anharmonicity and slightly lower than required to refine the fourth order. However, a slight improvement is noticeable only when the fourth order is included (slight flattening of the negative residual density in the case of both methods). It is observed that probability density function attains negative values in some areas around the Br atom in the case of MM refinement up to the fourth order and HAR refinement up to the third order. For PT-10(I) the experimental data resolution is slightly too low to refine even the third order of anharmonicity for I. However, improvement in terms of residual density is observed for both refinement methods and in terms of refined H ADPs for HAR. For PT-10(I), both HAR and MM yield Gram–Charlier coefficients significantly different than 0 in the case of anharmonicity refinement up to the third order, as well as refinement up to the fourth order. Nevertheless, probability density function is negative in huge fragments of space around the I atom only for MM when anharmonic thermal motion is refined up to the fourth order.

We also analysed the similarity index (*S*) between H ADPs obtained in the course of refinement with and without intensity cut-off (Table 5 ▸) and between H ADPs obtained with XWR and MM (Tables 6 ▸ and S2). In each case, we investigated how including refinement of anharmonicity influenced the level of agreement between H ADPs in the crystal structures. PT-2(Cl) was the only case in which refining anharmonicity increased similarity between H ADPs from XWR and MM – very slightly in the case of third-order anharmonicity refinement [*S* = 3.84/3.51% in the harmonic case and *S* = 3.66/3.31% in the anharmonic (*n* = 3) case – comparison between XWR and MM/MM(free)] and a little bit more visibly after adding the fourth order [*S* = 2.56/2.31% – comparison between XWR and MM/MM(free)]. For PT-11(S-Ph) the similarity was very slightly decreased [harmonic: *S* = 2.69/2.65%, anharmonic (*n* = 3): *S* = 2.72/2.65%, anharmonic (*n* = 4): *S* = 2.85/2.75%], as it also happened in the case of PT-8(Br) [harmonic: *S* = 11.79%, anharmonic (*n* = 3): *S* = 12.80%, anharmonic (*n* = 4): *S* = 12.93%]. For PT-10(I), increasing the order of anharmonicity decreased the similarity between XWR and MM H ADPs [harmonic: *S* = 15.35%, anharmonic (*n* = 3): *S* = 24.06%, anharmonic (*n* = 4): *S* = 27.09%].

### 3.4. Influence of intensity cut-off on the results   

Two versions of each type of refinement were performed: with the commonly applied intensity cut-off |*F*_o_| ≥ 2σ(|*F*_o_|) and without an intensity cut-off. Certain differences and similarities between these two approaches can be noted. For HAR, the number of Gram–Charlier coefficients significantly different than zero is similar with and without intensity cut-off when only third-order or both third- and fourth-order anharmonicity is refined. In particular, for the good-quality data sets, the results are very similar. Regardless of whether intensity cut-off was applied or not, for PT-11(S-Ph), HAR always resulted in no Gram–Charlier coefficients significantly different than 0. For PT-2(Cl), the cut-off and no cut-off types of refinement resulted in non-zero values of the same coefficients (both for refinements up to the third order and up to the fourth order); moreover, the values of those coefficients were very similar. For MM, it is much smaller without intensity cut-off in the case of refinement of the third anharmonicity. Refinement of third and fourth order for MM depends on the structure and the type of refinement (restrained or not restrained H positions). For PT-11(S-Ph), this number is lower for third-order ADPs, and for fourth-order ADPs it is similar. For PT-2(Cl), it is similar for MM cut-off/no-cut-off with restrained H positions and a slightly smaller for MM cut-off/no-cut-off with unrestrained H positions. For PT-8(Br) and PT-10(I), it is much smaller for MM with no-cut-off than for MM with cut-off.

Although the deformation density maps were very similar between the cut-off and no-cut-off cases, some differences were observed for fractal dimension plots and residual density maps (Figs. S1–S32). For PT-2(Cl), PT-10(I) and PT-11(S-Ph), fractal dimension plots are slightly narrower when intensity cut-off is applied. For PT-11(S-Ph), the areas of non-zero residual density are smaller in the case of intensity cut-off. Bigger differences are observed for PT-8(Br), for which fractal dimension plots are significantly narrower when intensity cut-off is applied.

For HAR and XWR of PT-2(Cl) and PT-8(Br), the amount of area of non-zero residual density is bigger with intensity cut-off applied and only in this case the residual density around Cl [which disappears only after (up to) fourth-order anharmonicity refinement] and Br atoms is present. This effect could be avoided by including all reflections. Both of these areas appear similar in the cut-off and no-cut-off cases of PT-10(I). However, only in the cut-off case are there significant amounts of high residual density around the I atom. For MM of PT-2(Cl) and PT-8(Br) with no intensity cut-off, the areas of non-zero residual density are bigger, particularly for PT-8(Br), and the residual density around Cl and Br is present in all cases. However, compared with the no cut-off cases, the residual density distribution around the Cl atom for the cut-off cases of PT-2(Cl) seems to be more spherically symmetrical. Furthermore, the amount of residual density around the I atom in the MM refinement of PT-10(I) is very similar for the cut-off and no-cut-off cases.

The differences between bond lengths in the cut-off and no-cut-off cases in HAR and MM vary among the data sets. Although the differences are mostly systematic, surprisingly, for the higher quality data sets, PT-2(Cl) and PT-11(S-Ph), higher differences are observed compared with the lower quality data sets. The highest differences (discussed jointly for HAR and MM) are observed for PT-11(S-Ph): they are in the quite narrow range −0.003 to 0.005 Å for bonds between the non-hydrogen atoms and in the range −0.05 to 0.037 Å for the bonds between non-hydrogen and hydrogen atoms. In comparison, these differences are significantly lower for PT-8(Br), from −0.0007 to 0.0007 Å for bonds between non-hydrogen atoms and −0.001 to 0.001 Å for bonds between non-hydrogen and hydrogen atoms. In general, applying or removing the cut-off does not influence in any particular way the level of agreement between the experimental and geometry-optimized *X*—H bond lengths. The usage of intensity cut-off also remains without influence on bond angles in the case of HAR and for both refinement methods in the case of good-quality data sets PT-2(Cl) and PT-11(S-Ph). However, for the remaining compounds, only MM refinement based on the full set of reflections is characterized by a similar level of agreement of the experimental and theory-derived bond angles to HAR. When intensity cut-off is applied, the discrepancy between the MM values and the theoretical values increases for PT-8(Br) up to 1°, and for PT-10(I) up to 3°.

The analysis of *S* was performed to compare ADPs of hydrogen atoms obtained using refinement based on two different sets of reflections (Table 5 ▸). It revealed that higher differences between data with and without intensity cut-off for HAR occur for worse-quality data. S attains values of 0.17–0.57% and *S* = 0.75–3.09% for PT-8(Br) and PT-10(I), whereas the values for PT-2(Cl) and PT-11(S-Ph) are between 0.03–0.04% and 0.01–0.03%. In the case of MM refinement, for worse quality data sets PT-8(Br) and PT-10(I), *SHADE2* was used to estimate the H ADPs, which obviously resulted in very high agreement between ADPs obtained with and without intensity cut-off. In the case of PT-2(Cl) and PT-11(S-Ph), for which H ADPs were refined with MM, the level of similarity is visibly lower – much lower than in the case of H ADPs from HAR for these structures and on a comparable level with H ADPs from HAR for the poor-quality data sets [PT-2(Cl): *S* = 0.58–1.28%, PT-11(S-Ph): *S* = 1.23–1.60%]. It is also often observed that free refinement of hydrogen positions increases similarity between H ADPs compared with the refinement with restrained hydrogen positions.

The statistical analysis of the obtained results revealed systematic changes both for the data obtained with the commonly applied intensity cut-off of |*F_o_*| ≥ 2σ(|*F_o_*|) and without intensity cut-off. For instance, *R* and *wR* were generally worse for the data without intensity cut-off. The differences between both data sets reach even 3.24%. Although systematic exceptions were observed for MM of PT-2(Cl), PT-10(I) and PT-11(S-Ph), the differences for these data sets were no higher than 0.07%. Moreover, the GOF parameter was closer to the one for the data without intensity cut-off. The only exceptions occurred for the MM refinements of PT-11(S-Ph). Finally, the values of ρ_min_ and ρ_max_ were mostly lower (between 0.01–1.07 e Å^−3^) and higher (between 0.01–1.08 e Å^−3^), respectively, for the data without intensity cut-off. In the majority of cases, removing the intensity cut-off did not significantly influence the shape and symmetry of the residual density distribution in fractal dimension plots. However, there were also a few cases in which using the full set of reflections made fractal dimension plots more symmetric and closer to the parabolic shape [PT-11(S-Ph) – MM anharmonic *n* = 3 and anharmonic *n* = 4 with restrained and unrestrained H positions; PT-2(Cl) – HAR and XWR anharmonic *n* = 4; PT-8(Br) – HAR, XWR and MM anharmonic *n* = 4]. Although, the statistical parameters were worse for data without intensity cut-off, the results including all reflections were more accurate in terms of agreement between the refined model of electron density and the experimental data, which was confirmed by the analysis of residual density.

### 3.5. Structural features – covalent bonds and ADPs   

**Figure 4:**
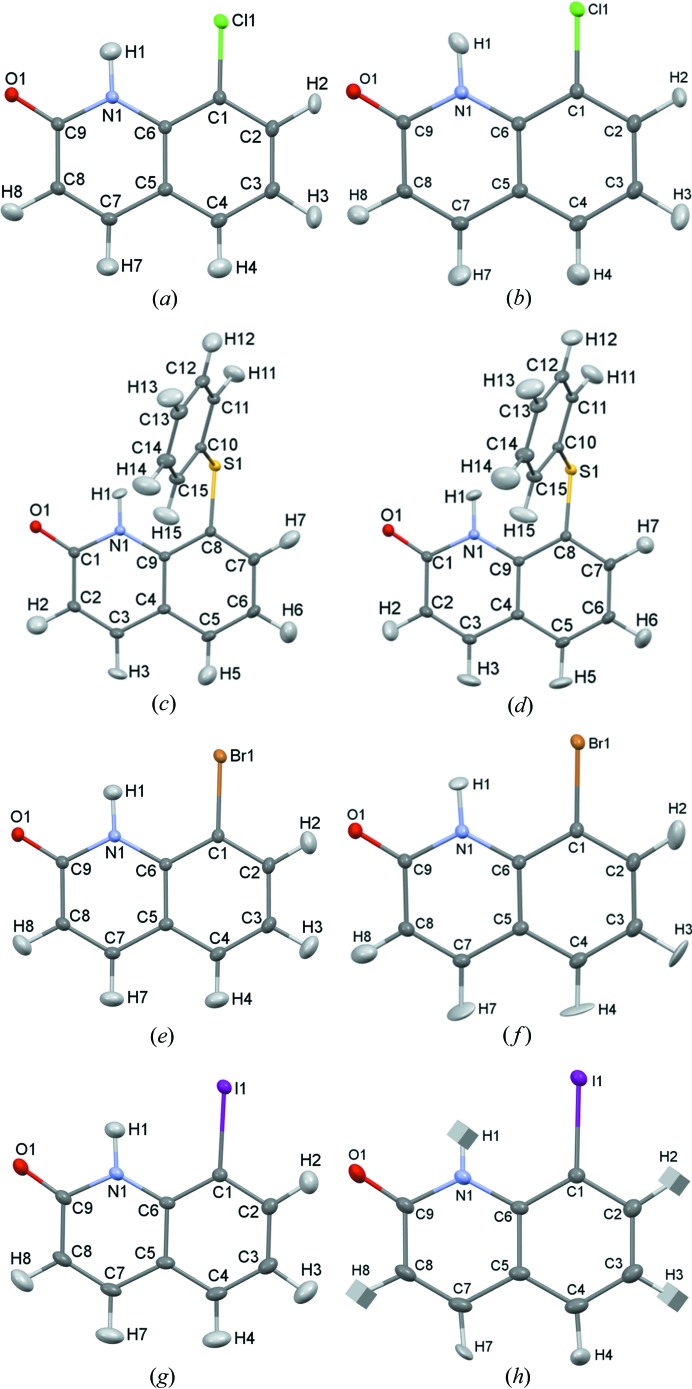
Crystal structures of the investigated compounds (harmonic refinement). (*a*) PT-2(Cl), MM, H ADPs refined. (*b*) PT-2(Cl), HAR, H ADPs refined. (*c*) PT-11(S-Ph), MM, H ADPs refined. (*d*) PT-11(S-Ph), HAR, H ADPs refined. (*e*) PT-8(Br), MM, H ADPs from *SHADE2*. (*f*) PT-8(Br), HAR, H ADPs refined. (*g*) PT-10(I), MM, H ADPs from *SHADE2*. (*h*) PT-10(I), HAR, H ADPs refined.

Crystal structures of the analysed compounds obtained in the course of harmonic HAR and MM refinement are depicted in Fig. 4 ▸. ADPs of hydrogen atoms are shown in all figures, which enables visual assessment of their quality and similarity between various methods of their refinement/estimation. For PT-11(S-Ph) and PT-2(Cl), H ADPs could be refined both with HAR and MM. For PT-8(Br) and PT-10(I), refinement of H ADPs was not possible with MM (therefore *SHADE2* was used in this case) and with HAR, which resulted in a few cases of H ADPs raising CheckCIF A alerts about (almost) NPD ellipsoids. These issues do not hinder the analysis of the bond lengths in PT-8(Br) and PT-10(I), since, as it was shown in the previous works (Woińska *et al.*, 2014 ▸, 2016 ▸), problematic refinement of anisotropic thermal motion of hydrogen atoms did not deteriorate their positions refined based on X-ray data. Increasing the order of anharmonicity does not visibly change H ADPs in MM (both those refined and those estimated with *SHADE2*), as well as H ADPs obtained with HAR in the case of PT-11(S-Ph) and PT-8(Br). For PT-2(Cl), there is a visible change in the shape and inclination of some H ADPs when a higher order of Cl anharmonic motions in HAR is included both in the cut-off and no cut-off cases. For PT-10(I), similar effects are observed; moreover, in the cut-off case, after increasing the order of anharmonicity to *n* = 3, two out of four H ellipsoids stop being (nearly) NPDs (from the N—H bond and from the C—H bond next to the O atom), and after refining *n* = 4 order of Gram–Charlier coefficients, the ellipsoid of the H atom bonded to C becomes (nearly) NPD again. In the no cut-off case of PT-10(I), the ellipsoids of the same H atoms bonded to C atoms remain (nearly) NPD regardless of the order of anharmonicity. There are also some slight changes in the ellipsoids of the heavy atoms for which anharmonic motion is refined. In the case of other non-H atoms there appears to be no visible changes.

All bond lengths can be evaluated by comparison with the values derived from theoretical geometry optimization. Lengths of bonds linking two non-H atom are, in general, equally well estimated with both refinement methods. The *X*—H distances were refined with HAR in every case, however, the result is strongly dependent on the data quality. For MM in the case of the low-quality data sets PT-8(Br) and PT-10(I), *X*—H distances had to be constrained to mean neutron bond lengths and were ‘refined’ with *MoPro*, which means that they were varied within a certain user-defined standard deviation (as a consequence, in the cif we obtain only mean neutron bond lengths rounded accordingly and with the error equal to the user-defined value). Only for the good-quality data sets PT-11(S-Ph) and PT-2(Cl), did MM allow unconstrained refinement of hydrogen positions (however, the refinement with H atoms restrained to mean neutron distances was also performed). Mean neutron distances are generally in quite good agreement with the theoretical distances, as only very typical *X*—H bonds are present in the investigated compounds. The comparison between the *X*–H bond lengths obtained with various methods and the calculated values is as follows:

(i) PT-11(S-Ph) and PT-2(Cl). The bond-length values obtained with MM are generally a little further from the theoretical ones and slightly less precise, as compared with HAR. HAR *X*—H distances are, in turn, only slightly further from the theoretical values than mean neutron bond lengths. These general observations are valid in particular for all C—H bonds and the N—H bond in PT-2(Cl). The only exception is the N—H bond in PT-11(S-Ph), in which the neutron mean value is in perfect agreement with the theoretical result, HAR is slightly less accurate and the value refined with MM is much further from the theoretical value [for comparison: optimized = 1.0311 Å, MM_harm_ = 1.029 (5) Å, MM_harm_(freeXH) = 0.993 (19) Å, HAR = 1.041 (6) Å].

(ii) PT-8(Br) amd PT-10(I): For PT-8(Br), precision of *X*—H bond determination with HAR decreases by one order of magnitude and some start to deviate from the theoretical values. For PT-10(I), this effect is even stronger, leading to clearly underestimated distances for bonds such as N1—H3, C7—H7 and C3—H3. Mean neutron distances are very close to the theoretical values.

### 3.6. Dimer interactions   

**Figure 5:**
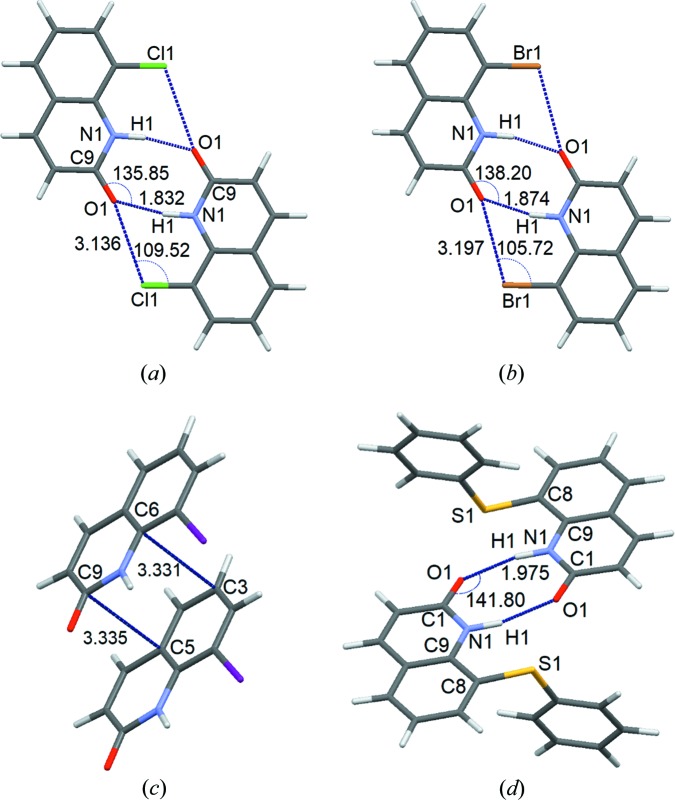
Selected strongly interacting dimers. Dimer interaction energies (in kJ mol^−1^) for harmonic refinement are given in brackets in the order optimized/HAR_anis_/IAM/MM/MM(freeXH). (*a*) PT-2(Cl), dimer 1 (*E* = −60.6/−61.8/−40.1/−60.3/−64.4). (*b*) PT-8(Br), dimer 10 (*E* = −55.7/−53.7/−37.9/−53.6/n/a). (*c*) PT-10(I), dimer 1 (*E* = 26.9/23.4/22.6/23.8/n/a). (*d*) PT-11, dimer 1 (*E* = −27.2/−27.9/−13.3/−26.9/−23.3).

The values of RMSD calculated between the zero-point dimer interaction energies obtained by the supramolecular approach for experimental and the optimized crystal geometries from theoretical calculations are given in Table S23 (the table also contains RMSD values scaled by the mean absolute value of energies of different dimer interactions in the structure calculated for the optimized structures). The strongest intermolecular interactions are presented in Fig. 5 ▸ and Table S24. Dimer interaction energies of all the dimers in the structures are also plotted as a function of centroid separation of dimers together with cohesive energy values in Figs. S54–S61. The observations and conclusions on dimer interactions can be described as follows:

#### 3.6.1. General comparison of HAR, MM and IAM   

All refinement methods (HAR and MM) provide a similar level of agreement of the dimer interaction energies obtained for the experimental geometries with the ones calculated for the optimized geometries (Table S23); the IAM results are, as expected, in much worse agreement with the values from geometry optimization. HAR verified against MM gives similar quality estimated energies of intermolecular interactions. Different positions of hydrogen atoms are probably the main source of differences in the estimated energies, *e.g.* free refinement of hydrogen atoms in MM increases the RMSD value.

#### 3.6.2. Comparison of the investigated compounds   

The lowest RMSD is obtained mostly for PT-11(S-Ph) and in some cases for PT-2(Cl), for which RMSD is quite similar to the values obtained for PT-11(S-Ph). For PT-8(Br), the RMSD is up to 3–4 times as high as for PT-11(S-Ph), for PT-10(I) it is 4–5 times as high as for PT-11(S-Ph) with intensity cut-off and around 2–3 times when intensity cut-off is not applied. For PT-10(I), the agreement of the IAM results with the theoretical ones is surprisingly high (for comparison, RMSD in kJ mol^−1^ attained by IAM amounts to 2.26 for PT-10(I), whereas for PT-2(Cl), PT-8(Br) and PT-11(S-Ph) it is equal to 8.38, 7.30 and 4.56, respectively), to the extent that it is only slightly worse than for HAR in the cut-off case (RMSD = 1.90 kJ mol^−1^ for HAR_anis_, the cut-off case).

#### 3.6.3. Refinement of anharmonic motion   

Refinement of the higher order of thermal motions seems to remain without influence on the interactions of dimers, since it does not change the geometry of the analysed crystal structures.

#### 3.6.4. Influence of intensity cut-off   

For PT-2(Cl) application of intensity cut-off has a very small influence on the value of RMSD, except the case of MM(freeXH) refinement, for which using intensity cut-off increases RMSD at least two times. This is also the case for PT-11(S-Ph), for which, the increase of RMSD in the MM(freeXH) refinement is not as strong. For PT-8(Br), using intensity cut-off causes only very small changes in the value of RMSD, usually a small decrease. PT-10(I), in turn, is the only structure for which a more considerable influence of intensity cut-off on RMSD is observed [*e.g.* RMSD = 1.90 kJ mol^−1^ for HAR_anis_(cut-off) and RMSD = 0.90 kJ mol^−1^ for HAR_anis_(no cut-off); RMSD = 1.32 kJ mol^−1^ for MM (cut-off) and RMSD = 1.12 kJ mol^−1^ for MM(no cut-off)].

#### 3.6.5. Halogen bonds and dimer interactions   

**Figure 6:**
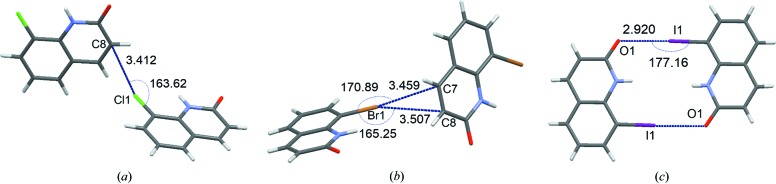
Halogen bonds present in the crystal structures. Dimer interaction energies in kJ mol^−1^ for harmonic refinement are given in brackets in the order optimized/HAR_anis_/IAM/MM/MM(freeXH). (*a*) PT-2(Cl) (*E* = −4.9/−4.7/−5.3/−4.7/−4.5). (*b*) PT-8(Br) (*E* = −7.1/−6.8/−7.2/−6.8/n/a). (*c*) PT-10(I) (*E* = −35.9/−32.4/−31.2/−33.0/n/a).

Halogen bonds are present in the structures of PT-2(Cl), PT-8(Br) and PT-10(I) (Fig. 6 ▸). Dimer interactions in PT-2(Cl) and PT-8(Br) mediated only by halogen bonds [Figs. 6 ▸(*a*) and 6(*b*)] are quite weak [Table S25; energies of interactions obtained with various methods and without intensity cut-off in the order optimized/HAR_anis_/IAM/MM/MM(freeXH) expressed in kJ mol^−1^ are as follows: −4.9/−4.7/−5.3/−4.7/−4.5 for PT-2(Cl) and −7.1/−6.8/−7.2/−6.8/n/a for PT-8(Br)] compared with the strongest dimer interactions in the structures (Table S24), which are stabilized by 2–4 various contacts including other halogen bonds. In the case of PT-2(Cl), the bond path and the bond critical point found between atoms C8 and Cl1 is a confirmation of the presence of the halogen bonds according to the set of criteria proposed by Koch & Popelier (Koch & Popelier, 1995 ▸; Popelier, 1998 ▸) for confirming the presence of hydrogen bonding and also used for the analysis of halogen bonds (Martinez Amezaga *et al.*, 2010 ▸; Mallinson *et al.*, 2003 ▸; Dominiak *et al.*, 2006 ▸). In the case of PT-8(Br), topological analysis reveals the absence of a halogen bond similar to the one found in PT-2(Cl). In the case of PT-10(I), in which two C—I⋯O halogen bonds stabilize the dimer [Fig. 6 ▸(*c*)], the interaction energy (energies of interactions obtained using various methods and without intensity cut-off in the order optimized/HAR_anis_/IAM/MM expressed in kJ mol^−1^ are the following: −35.9/−32.4/−31.2/−33.0) is comparable with the energy of dimer 2, which is another strongly interacting dimer in the structure of PT-10(I). In the case of dimer 2 in PT-10, the strongest dimer interaction is a π–π stacking interaction with energy slightly above −30 kJ mol^−1^, the presence of which is also confirmed by C⋯C and C⋯N bond paths and critical points between atoms from the interacting aromatic rings. In PT-2(Cl), the most strongly interacting dimer is dimer 1 [Fig. 5 ▸(*a*)] with energy of interactions over −60 kJ mol^−1^. The most strongly interacting dimer in PT-8(Br) is dimer 10 [Fig. 5 ▸(*b*)] with energy of interactions over −50 kJ mol^−1^. These dimers have similar geometries and are stabilized by two N—H⋯O hydrogen bonds and two C—Cl(Br)⋯O halogen bonds. The energy of interaction in the strongest dimer interaction in PT-11(S-Ph) [Fig. 5 ▸(*d*)] is slightly more than −50 kJ mol^−1^. This interaction is stabilized by two hydrogen bonds, *i.e.* N—H⋯O. Hydrogen bonds are present only in PT-2(Cl), PT-8(Br) and PT-10(I). In each case, they stabilize the strongest molecular dimers which are bonded (among others) *via* two symmetry related hydrogen bonds (N—H⋯O).

### 3.7. Crystal packing and lattice interactions   

The crystal structure of each compound contains one molecule in the asymmetric unit and belongs to the space group *P*2_1_/*c*. All the compounds share the same molecular subunit of quinoline, however, various substituents contribute to the differences between the crystal lattices of the analysed compounds. PT-2(Cl) and PT-8(Br) share the same type of crystal packing, owing to the same type of intermolecular interactions being present in both (*e.g.* hydrogen bonds N—H⋯O), resulting in very similar molecular dimers in the structures. In the case of PT-11(S-Ph), the same type of hydrogen bond is observed mediating interactions in a similar type of a dimer. However, due to the presence of a much larger substituent (-S-Ph) the crystal structure of this compound differs from the structures of PT-2(Cl) and PT-8(Br). The crystal network comprises layers consisting of quinoline rings with phenyl rings of the substituent sticking out from the layers. In the case of PT-10(I), the iodine substituent characterised by higher electron density and longer bond lengths, forces a different crystal geometry with no hydrogen bonds. The molecules are arranged in non-overlapping wavy layers parallel to the (102) crystallographic planes. Halogen bonds link molecules within one layer, whereas the layers interact with one another *via* π–π stacking interactions of the quinoline rings.

‘Cohesive energies’ of the optimized structures of the investigated compounds are quite similar (Tables 7 ▸ and S26) and point to the comparable stability of the crystals of the investigated compounds. The structures of PT-2(Cl) and PT-8(Br) are characterized by very similar cohesive energy values, since their networks of interactions are also very similar. PT10-(I) is characterized by higher cohesive energy values, which suggests that the interactions in the layered structure contribute to better stabilization. However, the most stable of the structures is PT-11(S-Ph), which is probably due to the fact that in this structure each molecule is involved in interactions within four more dimers than it is in the case of the remaining compounds. It must be noted that all the calculated cohesive energies are zero-point energies, whereas experimental geometries were determined at 90 K. Nevertheless, for PT-2(Cl), PT-8(Br) and PT-11(S-Ph) the differences between cohesive energies for the experimental and optimized geometries are usually not higher than 8 kJ mol^−1^ (or not higher than 20 kJ mol^−1^ for the MM(freeXH) cases). In the case of PT-10(I), these differences are of the order of 10 kJ mol^−1^ for MM and are considerably higher for HAR geometries (of the order of 70–90 kJ mol^−1^). However, there are no significant differences in cohesive energy both for HAR and MM geometries for the structures refined using the full set of reflections and the cut-off set of reflections [0–1 kJ mol^−1^ for PT-2(Cl), PT-8(Br) and PT-11(S-Ph), and 0.3–3.3 kJ mol^−1^]. The only exception are the MM(freeXH) geometries, for which larger discrepancies of 1.2–31.1 kJ mol^−1^ with the optimized geometry are observed.

‘Geometrical relaxation energies’ (Tables 7 ▸ and S27) of all the compounds are usually lower (in many cases much lower) in terms of their absolute value when calculated for the optimized geometries than the values calculated for experimentally derived geometries. In the case of optimized geometries, the structure characterized by the lowest relaxation energy is PT-10(I), which might be caused by the specific geometry of molecular layers present in the structure, which facilitates preserving the original shape of the molecule, or by the fact that the nature of interactions in this structure is different than in the other structures [the strongest dimer interaction is not as strong as the strongest dimer interactions in the other structures, neither does it involve hydrogen bonds, which are not present in PT-10(I)]. The analysis of relaxation energies calculated for experimentally obtained structures shows a considerable difference in trend compared with those observed for theoretical geometries. In this case, the values of relaxation energy seem to be strongly related with the quality of the experimental crystal structure, particularly with the correctness of determining positions of hydrogen atoms. The relaxation energy values from MM with H positions restrained to average neutron distances are in better agreement with the theoretical values compared with HAR. The situation changes when hydrogen positions are freely refined with MM, as observed for PT-2(Cl) and PT-11(S-Ph) for which MM(freeXH) refinement diverges significantly more from the theoretical values than with HAR. For the poorest quality structure PT-10(I), the HAR results are particularly discrepant from the values calculated for the optimized geometry. The corresponding cases of refinement performed on the whole data set and the cut-off set of reflections usually result in fairly similar values of relaxation energy. However, a small increase in similarity to the theoretical values is observed in the case of MM for PT-10(I) and MM(freeXH) for PT-2(Cl) as well as a more significant improvement in the case of PT-11(S-Ph)(freeXH).

## 4. Conclusions   

Both of the studied refinement methods, XWR and MM, enabled successful refinement of positions and ADPs of heavy atoms, which was confirmed by comparison with theoretical bond lengths. XWR enabled refinement of hydrogen positions for each compound, yielding bond lengths in good agreement with the theoretical values for PT-11(S-Ph) and PT-2(Cl) (slightly further from the theoretical values than mean neutron distances), and resulting in bond lengths of considerably deteriorated quality and precision for PT-8(Br) and PT-10(I). In the case of MM, refinement of the hydrogen positions was only possible for the first two compounds, yet the results were less precise and less accurate compared with XWR. For the good-quality data sets, PT-11(S-Ph) and PT-2(Cl), both XWR and MM enabled refinement of hydrogen ADPs, leading to highly similar and reliable values. For the poor-quality data set PT-8(Br), XWR was the only method with which refinement of hydrogen ADPs was possible, resulting, however, in a significantly decreased quality of ellipsoids. For a challenging data set such as PT-10(I), even XWR was unsuccessful in refining hydrogen ADPs. However, it is necessary to remember that in the case of MM, the final solution is reached in the course of a complex optimization procedure involving decisions made by the user, whereas in the case of XWR, the procedure leading to the least-squares solution is much simpler.

XWR, as a method characterized by stronger restraint to theoretical electron density, assures better quality of electron density and allows us to avoid certain incorrect features (*e.g*. deformation of lone electron pairs of O atoms and of electron density of Cl, Br and I atoms), which is particularly visible for the more problematic data sets. Providing higher similarity to the theoretical electron density, XWR at the same time results in higher agreement of the model density with the experimental data – residual density is flatter and fractal dimension plots are narrower and more symmetric.

In the majority of cases, the estimated resolution limits necessary to refine specific orders of anharmonic vibrations of S, Cl, Br and I atoms were not attained in the experiment. Still, for the poorer quality data sets, PT-8(Br) and PT-10(I), both refinement methods resulted in non-zero values of third- and fourth-order Gram–Charlier coefficients, as well as in a visible improvement in the reconstructed electron density and H ADPs [PT-10(I)]. In the case of PT-11(S-Ph), for which experimental resolution was too low to refine Gram–Charlier coefficients for the S atom, only HAR correctly predicted that the values of these parameters should be equal to zero. For PT-2(Cl), the importance of including all reflections in the refinement was particularly prominent, since it cleared the high residual density around the Cl atom, which could otherwise be removed only by third- and fourth-order Gram–Charlier coefficient refinement (for which data resolution was not sufficient). Similarly, for PT-8(Br), refinement performed using the full data set cleared the significant amount of residual density in the vicinity of Br. In the case of MM, including all reflections helped to decrease the number of non-zero third-order Gram–Charlier coefficients in the anharmonic refinement. The analysis of probability density functions does not yield unambiguous results which would confirm that anharmonic motion should not be refined, since in many cases, when there is other evidence showing the absence of anharmonic motion, the probability density function is still positive.

Applying an intensity cut-off does not in any particular way influence the level of agreement between the experimental and theoretical *X*—H bond lengths for HAR and MM, and experimental and theoretical bond angles for HAR. However, for MM, the agreement between experimental and theoretical bond angles is worse after applying a cut-off for poor-quality data sets PT-8(Br) and PT-10(I). In general, removing the intensity cut-off did not significantly influence the shape or symmetry of the residual density distribution in fractal dimension plots, except in a few cases in which fractal dimension plots became more symmetric and closer to a parabolic shape.

Among the investigated compounds, PT-2(Cl) and PT-8(Br) are characterized by the same type of crystal packing resulting from very similar intermolecular interactions in the crystal lattice (*e.g.* hydrogen bonds N—H⋯O and halogen bonds C—*X*⋯O) and, consequently, by comparable stability confirmed by similar values of cohesive energy. The same type of hydrogen bond is observed in PT-11(S-Ph), however, due to the presence of the large substituent, this compound forms a different crystal network, which is also the most stable among the analysed structures. In PT-10(I), no hydrogen bonds are present and the molecules form slabs parallel to the (102) crystallographic planes – the molecules within one slab form dimers interacting *via* halogen bonds, whereas the layers interact *via* π-stacking interactions between neighbouring quinoline rings. This compound is also more stable than the other two halogen derivatives. Since there are no substantial differences in experimental geometries between the refinement performed on the ‘full’ and ‘cut’ data sets, the energetic studies yield values of energy which are very similar between these two cases. Usually, a similar conclusion can be drawn for the comparison of harmonic and anharmonic refinement. The value of geometrical relaxation energy is in relation to the data quality [it attains a particularly high value in the case of PT-10(I)] and is also increased by free refinement of H positions (more significantly in the case of MM). Energies of dimer interactions calculated for the molecular geometries obtained with each refinement method are in similar agreement with the energies obtained for the optimized geometries. Differences in the calculated interaction energies can be attributed to the differences in the positions of hydrogen atoms, which are revealed by the comparison of refinement cases of freely refined versus restrained H positions. Despite the ongoing progress in experimental techniques and methods of data processing, theoretical methods are still the most reliable source of precise information about H positions.

The research presented allows us to conclude that XWR performs slightly better than MM when applied to high-quality data and significantly better when poor-quality data is analysed. Finally, this study highlights the importance of including the complete data set in the refinement, even the weak reflections, as this avoids spurious effects, such as anharmonicity, and facilitates correct interpretation of the results obtained.

## Supplementary Material

Crystal structure: contains datablock(s) cut_off_PT10_HAR_anh3, cut_off_PT10_HAR_anh4, cut_off_PT10_HAR_harm_ani, cut_off_PT11_HAR_anh3, cut_off_PT2_HAR_harm_ani, cut_off_PT8_HAR_ahn3, cut_off_PT8_HAR_harm_ani, no_cut_off_PT10_HAR_anh3, no_cut_off_PT10_HAR_harm_ani, no_cut_off_PT11_HAR_anh3, no_cut_off_PT11_HAR_harm_ani, no_cut_off_PT2_HAR_anh3, no_cut_off_PT2_HAR_harm_ani, no_cut_off_PT-2_MM_anh3, no_cut_off_PT8_HAR_anh3, no_cut_off_PT8_HAR_harm_ani, PT10_IAM, PT11_IAM, PT2_IAM. DOI: 10.1107/S2052252519007358/lt5019sup1.cif

Supplementary Tables and Figures. DOI: 10.1107/S2052252519007358/lt5019sup2.pdf

CCDC references: 1935133, 1935134, 1935135, 1935136, 1935137, 1935138, 1935139, 1935140, 1935141, 1935142, 1935143, 1935144, 1935145, 1935146, 1935147, 1935148, 1935149, 1935150, 1935151, 1935152, 1935153, 1935154, 1935155, 1935156, 1935157, 1935158, 1935159, 1935160, 1935161, 1935162, 1935163, 1935164, 1935165, 1935166, 1935167, 1935168, 1935169, 1935170, 1935171, 1935172, 1935173, 1935174

## Figures and Tables

**Figure 3:**
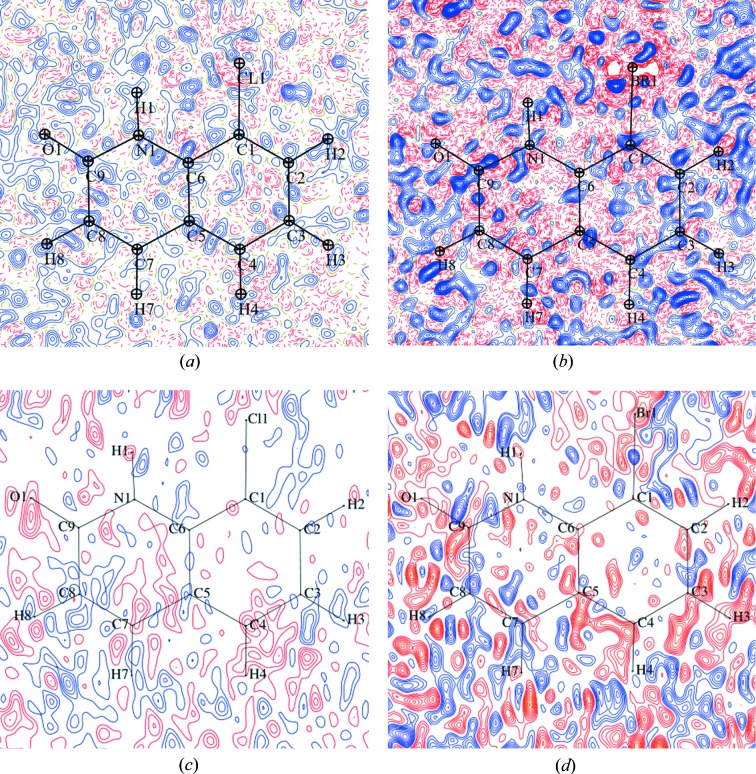
Residual density maps for high-quality [PT-2(Cl)] and low-quality [PT-8(Br)] data without intensity cut-off. Contour level: 0.05 e Å^−3^. Colours: blue – positive, red – negative. (*a*) PT-2(Cl), MM (freeXH), harmonic. (*b*) PT-8(Br), MM, harmonic. (*c*) PT-2(Cl), HAR, harmonic. (*d*) PT-8(Br), HAR, harmonic.

**Table 1:** X-ray data collection and structure refinement details

	PT-11(S-Ph)	PT-2(Cl)	PT-8(Br)	PT-10(I)
Chemical formula	C_15_H_11_NOS	C_9_H_6_NOCl	C_9_H_6_NOBr	C_9_H_6_NOI
Molecular weight, *M*_r_ (g mol^−1^)	253.309	179.595	224.047	271.047
Temperature (K)	90	90	90	90
Crystal system	Monoclinic	Monoclinic	Monoclinic	Monoclinic
Space group	*P*2_1_/*c*	*P*2_1_/*c*	*P*2_1_/*c*	*P*2_1_/*c*
*a* (Å)	8.2172 (2)	10.99751 (13)	11.26337 (9)	4.3040 (2)
*b* (Å)	18.4903 (4)	4.59832 (6)	4.49437 (4)	18.2223 (9)
*c* (Å)	8.7912 (3)	15.46951 (16)	15.88027 (11)	10.5825 (5)
β (°)	115.120 (4)	107.6147 (12)	106.0349 (8)	94.0520 (10)
*V* (Å^3^)	1209.4 (5)	745.614 (15)	772.610 (11)	827.90 (7)
*Z*	4	4	4	4
*d*_calc_ (g cm^−3^)	1.392	1.600	1.926	2.175
*F*_000_	528	368	440	512
Absorption coefficient, μ (mm^−1^)	0.250	0.449	5.260	3.812
Crystal size (mm^3^)	0.176 × 0.220 × 0.238	0.091 × 0.127 × 0.202	0.054 × 0.106 × 0.305	0.048 × 0.071 × 0.230
θ range (°)	2.20-53.43	1.94-66.28	1.88-66.23	2.23-48.60
sin(θ/λ) (Å^−1^)	1.14	1.25	1.25	1.06
Completeness (%)	98.1	93.7	93.7	97.0
Index ranges	−18 ≤ *h* ≤ 16	−28 ≤ *h* ≤ 26	−28 ≤ *h* ≤ 27	−9 ≤ *h* ≤ 9
	0 ≤ *k* ≤ 41	0 ≤ *k* ≤ 11	0 ≤ *k* ≤ 11	0 ≤ *k* ≤ 38
	0 ≤ *l* ≤ 19	0 ≤ *l* ≤ 39	0 ≤ *l* ≤ 40	0 ≤ *l* ≤ 22
No. of measured/unique reflections	221308/14614	186085/13309	191752/13800	77688/8089
No. of parameters/restraints	207/0	133/0	133/0	133/0
*S*[*F* ^2^] (all) (GOF)	1.0847	1.079	1.048	1.122
*R*[*F* ^2^] [*I* > 2σ(*I*)]/(all)	0.0264/0.0306	0.0384/0.0562	0.0291/0.0617	0.0205/0.0264
*wR*[*F* ^2^] [*I * > 2σ(*I*)]/(all)	0.1277/ 0.1318	0.1110/0.1259	0.0613/0.0724	0.0496/0.0516
Δρ_min/max_ (e Å^−3^)	−0.254/0.655	−0.941/0.709	−1.570/1.129	−1.830/1.523

**Table 2:** Statistical parameters of the refinements

		PT-11(S-Ph)				PT-2(Cl)				PT-8(Br)			PT-10(I)		
		HAR	XWR	MM	MM(freeXH)	HAR	XWR	MM	MM(freeXH)	HAR	XWR	MM	HAR	XWR	MM
Harmonic	*R* (%)	2.08	1.97	1.44	1.44	4.59	4.36	2.71	2.71	5.73	5.57	2.54	2.74	2.46	1.86
	*wR* (%)	2.77	2.65	4.71	4.71	3.37	3.18	6.11	6.11	2.94	2.82	4.60	2.72	2.33	4.43
	GOF	1.777	1.697	1.607	1.607	1.426	1.346	1.439	1.439	1.131	1.085	1.053	1.463	1.254	1.257
	ρ_max_	0.34	0.27	0.32	0.33	0.35	0.39	0.52	0.55	1.13	1.17	1.04	0.71	0.70	1.82
	ρ_min_	−0.18	−0.18	−0.21	−0.22	−0.49	−0.46	−0.62	−0.63	−1.23	−1.20	−1.92	−1.54	−1.14	−1.95
	χ^2^	3.16	2.88	n/a	n/a	2.03	1.82	n/a	n/a	1.28	1.18	n/a	2.14	1.57	n/a
	λ_max_	1.130	1.130	1.130	1.130	2.030	2.030	2.030	2.030	9.960	9.960	9.960	6.890	6.890	6.890
															
Anharmonic (*n* = 3)	*R* (%)	2.05	n/a	1.41	1.41	4.58	n/a	2.70	2.70	5.72	n/a	2.52	2.55	n/a	1.63
	*wR* (%)	2.73	n/a	4.62	4.62	3.35	n/a	6.08	6.08	2.92	n/a	4.56	2.52	n/a	3.90
	GOF	1.749	n/a	1.580	1.580	1.417	n/a	1.433	1.433	1.124	n/a	1.048	1.359	n/a	1.117
	ρ_max_	0.19	n/a	0.18	0.19	0.51	n/a	0.52	0.53	1.01	n/a	1.13	0.61	n/a	1.34
	ρ_min_	−0.16	n/a	−0.20	−0.21	−0.34	n/a	−0.51	−0.53	−1.08	n/a	−1.86	−0.80	n/a	−1.14
	χ^2^	3.06	n/a	n/a	n/a	2.01	n/a	n/a	n/a	1.26	n/a	n/a	1.85	n/a	n/a
															
Anharmonic (*n* = 3, 4)	*R* (%)	2.05	1.95	1.41	1.41	4.28	4.19	2.66	2.66	5.66	5.60	2.45	2.45	2.35	1.61
	*wR* (%)	2.72	2.61	4.62	4.62	3.17	3.06	6.01	6.01	2.87	2.75	4.40	2.47	2.27	3.87
	GOF	1.742	1.673	1.579	1.578	1.344	1.295	1.420	1.420	1.106	1.059	1.016	1.333	1.222	1.110
	ρ_max_	0.21	0.16	0.19	-0.21	0.32	0.32	0.56	0.57	0.95	0.92	1.13	0.67	0.66	1.27
	ρ_min_	−0.15	−0.13	−0.19	0.20	−0.31	−0.30	−0.41	−0.43	−0.98	−0.99	−1.57	−0.75	−0.69	−1.11
	χ^2^	3.03	2.80	n/a	n/a	1.81	1.68	n/a	n/a	1.22	1.12	n/a	1.78	1.50	n/a
	λ_max_	1.210	1.210	1.210	1.210	2.260	2.260	2.260	2.260	10.570	10.570	10.570	6.410	6.410	6.410

**Table 3:** Minimal resolution limits required, according to the program *XDPROP* (version 5.42; Volkov *et al.*, 2006 ▸), for the refinement of anharmonic thermal motions of given chemical elements in the studied compounds Numbers of non-zero Gram–Charlier coefficients for various refinement methods (no intensity cut-off applied) and different anharmonicity orders are given in brackets in the order HAR/MM/MM(freeXH).

	Resolution (Å^−1^)							
		PT-11(S-Ph)		PT-2(Cl)		PT-8(Br)		PT-10(I)
Experimental	1.14		1.25		1.25		1.06	
Anharmonic (*n* = 3)	1.25	(0/3/4)	1.12	(4/0/1)	1.15	(2/1/n/a)	1.11	(7/3/n/a)
Anharmonic (*n* = 3, 4)	1.45	(0/4/4) third order	1.29	(5/7/3) third order	1.33	(3/0/n/a) third order	1.28	(7/4/n/a) third order
		(0/3/0) fourth order		(8/7/5) fourth order		(9/1/n/a) fourth order		(9/2/n/a) fourth order

**Table 5:** Similarity index for H ADPs between data with commonly applied intensity cut-off |*F*_o_| ≥ 2σ(|*F*_o_|) and without intensity cut-off

PT-11(S-Ph)	Structure	PT-2(Cl)	PT-8(Br)	PT-10(I)	PT-11(S-Ph)
Harmonic	HAR	0.03	0.53	0.75†	0.02
	MM	1.28	0.00	0.01	1.60
	MM(free XH)	0.82	n/a	n/a	1.60
					
Anharmonic (*n* = 3)	HAR	0.03	0.17	3.09‡	0.03
	MM	1.04	0.00	0.01	1.57
	MM(freeXH)	0.67	n/a	n/a	1.28
					
Anharmonic (*n* = 3, 4)	HAR	0.04	0.57	0.99†	0.01
	MM	0.94	0.00	0.01	1.41
	MM(freeXH)	0.58	n/a	n/a	1.23

† The analysis excludes H2, H3, H8 and H1 for HAR refinement of PT-10(I) due to the presence of zero-positive values.

‡ The analysis excludes H2, H3 and H8 for HAR refinement of PT-10(I) due to the presence of zero-positive values.

**Table 6:** Similarity index for H ADPs between HAR and MM data. Results for data without intensity cut-off

	Structure	PT-2(Cl)	PT-8(Br)	PT-10(I)	PT-11(S-Ph)
	HAR/MM	3.84	11.79	15.35†	2.69
Harmonic	HAR/MM(freeXH)	3.51	n/a	n/a	2.65
					
Anharmonic (*n* = 3)	HAR/MM	3.66	12.80	24.06‡	2.72
	HAR/MM(freeXH)	3.31	n/a	n/a	2.65
					
Anharmonic (*n* = 3, 4)	HAR/MM	2.65	12.93	27.09‡	2.85
	HAR/MM(freeXH)	2.31	n/a	n/a	2.75

† The analysis excludes H2, H3, H8 and H1 for HAR refinement of PT-10(I) due to the presence of zero-positive values.

‡ The analysis excludes H2, H3 and H8 for HAR refinement of PT-10(I) due to the presence of zero-positive values.

**Table 7:** Geometrical relaxation energies and cohesive energies calculated for crystal structures refined without intensity cut-off.

		Geometrical relaxation energy (kJ mol^−1^)				Cohesive energy (kJ mol^−1^)			
	Structure	PT-2(Cl)	PT-8(Br)	PT-10(I)	PT-11(S-Ph)	PT-2(Cl)	PT-8(Br)	PT-10(I)	PT-11(S-Ph)
Harmonic	Optimized	−5.6	−7.3	−3.6	−8.9	−97.2	−96.4	−111.8	−114.3
	HAR_anis_	−9.2	−13.7	−72.6	−15.7	−93.1	−89.4	−41.8	−108.1
	HAR_iso_	−9.2	−13.9	−75.0	−14.9	−93.0	−89.2	−40.2	−108.6
	IAM	−247.9	−273.2	−606.4	404.7	150.4	172.9	486.8	281.1
	MM	−8.5	−5.6	−13.9	−12.2	−94.5	−96.5	−99.9	−110.9
	MM(freeXH)	−21.2	n/a	n/a	−19.4	−81.1	n/a	n/a	−100.9
									
Anharmonic (*n* = 3)	HAR_anis_	−9.37	−13.61	−77.02	−15.7	−93.1	−89.6	−37.2	−108.1
	MM	−8.47	−5.73	−11.6	−12.2	−94.5	−96.5	−101.8	−110.9
	MM(freeXH)	−20.71	n/a	n/a	−18.4	−81.5			−102.1
									
Anharmonic (*n* = 3, 4)	HAR_anis_	−7.94	−14.58	−92.18	−16.12	−94.2	−88.8	−22.1	−107.8
	MM	−8.51	−5.91	−10.97	−12.2	−94.5	−96.3	−102.4	−110.9
	MM(freeXH)	−18.80	n/a	n/a	−18.5	−83.3			−101.9

**Table 4:** Graphical representation of the probability density function at the 50% probability level for various refinement types of the PT-2(Cl) data set

	No intensity cut-off		Intensity cut-off |*F*| ≥ 2σ(|*F*|)	
Refinement type	Anharmonic (*n* = 3)	Anharmonic (*n* = 3, 4)	Anharmonic (*n* = 3)	Anharmonic (*n* = 3, 4)
HAR	[Chem scheme1] 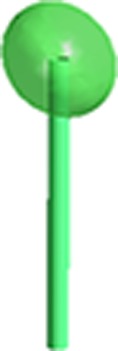	[Chem scheme2] 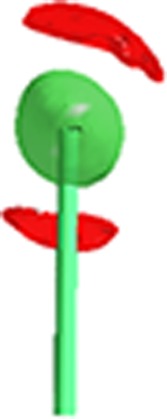	[Chem scheme3] 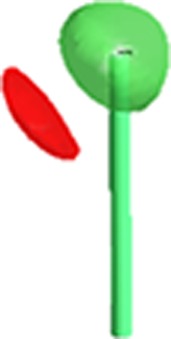	[Chem scheme4] 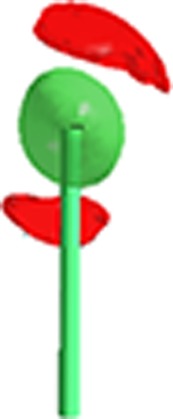
MM	[Chem scheme5] 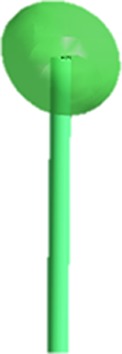	[Chem scheme6] 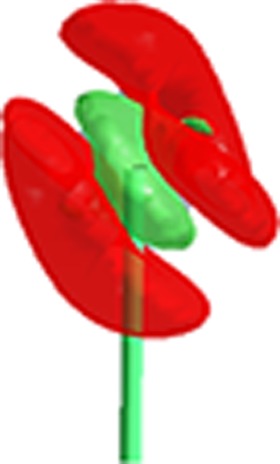	[Chem scheme7] 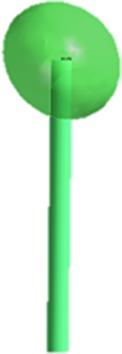	[Chem scheme8] 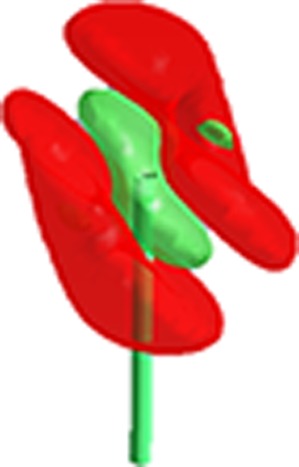
MM(freeXH)	[Chem scheme9] 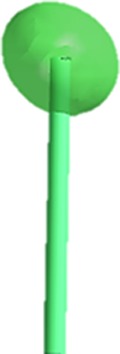	[Chem scheme10] 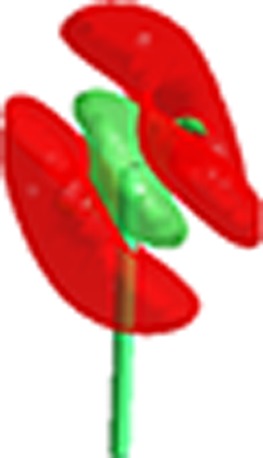	[Chem scheme11] 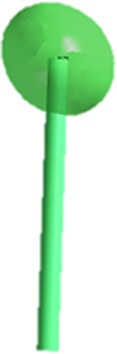	[Chem scheme12] 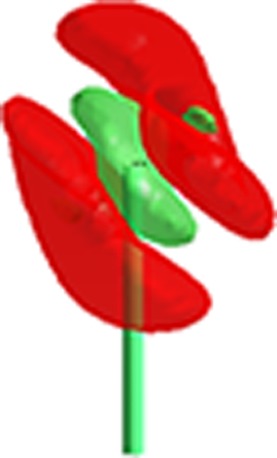
