# Supplementary material for: Energetics of interactions in the solid state of 2-hydroxy-8-X-quinoline derivatives (X = Cl, Br, I, S-Ph): comparison of Hirshfeld atom, X-ray wavefunction and multipole refinements
Source: IUCrJ. 2019 Jul 15;6(Pt 5):868–83. doi: 10.1107/S2052252519007358 (PMC6760436; doi:10.1107/S2052252519007358)
Supplement: Supplementary file 2 [file m-06-00868-sup2.pdf]

IUCrJ

Volume 6 (2019)

Supporting information for article:

Energetics of interactions in the solid state of 2-hydroxy-8-*X*-quinoline derivatives (*X* = Cl, Br, I, S-Ph): comparison of hirshfeld atom, X-ray wavefunction and multipole refinements

Magdalena Woinska, Monika Wanat, Przemyslaw Taciak, Tomasz Pawinski, Wladek Minor and Krzysztof Wozniak

Energetics of Interactions in the Solid State on the Example of 2-Hydroxo-8-X-Quinoline Derivatives (X = Cl, Br, I, S-Ph): Comparison of Hirshfeld Atom, X-ray Wavefunction and Multipole Refinements

1 Statistical and structural parameters

Table 1: Statistical parameters of the refinements (intensity cut-off: $|F| \geq 2\sigma(|F|)$).

		PT-11(S-Ph)				PT-2(Cl)				PT-8(Br)			PT-10(I)		
		HAR	XWR	MM	MM (freeXH)	HAR	XWR	MM	MM freeXH	HAR	XWR	MM	HAR	XWR	MM
harmonic	R	1.61%	1.50%	1.47%	1.46%	2.93%	2.70%	2.71%	2.71%	2.50%	2.43%	2.51%	2.08%	1.80%	1.91%
	wR	2.73%	2.59%	2.57%	2.56%	3.22%	3.02%	3.02%	3.02%	2.64%	2.50%	2.42%	2.65%	2.25%	2.48%
	GOF	1.85	1.76	1.56	1.44	1.59	1.49	1.52	1.52	1.26	1.19	1.16	1.54	1.30	1.46
	ρ_{min}	-0.15	-0.15	-0.20	-0.20	-0.43	-0.36	-0.59	-0.60	-0.85	-0.82	-1.56	-1.46	-1.05	-1.94
	ρ_{max}	0.33	0.26	0.35	0.34	0.33	0.33	0.47	0.47	0.58	0.52	0.63	0.74	0.63	1.93
	χ^2	3.43	3.88			2.52	2.22			1.58	1.42		2.36	1.70	
	λ_{max}		1.180				1.570				7.570			6.060	
anharmonic (n=3)	R	1.58%		1.44%	1.44%	2.92%		2.70%	2.70%	2.49%		2.50%	1.90%		1.69%
	wR	2.68%		2.51%	2.51%	3.19%		3.01%	3.01%	2.62%		2.40%	2.45%		2.24%
	GOF	1.82		1.53	1.53	1.58		1.51	1.51	1.25		1.15	1.42		1.32
	ρ_{min}	-0.14		-0.20	-0.20	-0.42		-0.48	-0.48	-0.80		-1.54	-0.76		-1.05
	ρ_{max}	0.18		0.20	0.20	0.32		0.47	0.47	0.58		0.64	0.64		1.16
	χ^2	3.32				2.49			1.56				2.02		
anharmonic (n=3, 4)	R	1.58%	1.48%	1.44%	1.44%	2.60%	2.51%	2.66%	2.67%	2.43%	2.36%	2.43%	1.80%	1.70%	1.68%
	wR	2.67%	2.56%	2.51%	2.51%	3.01%	2.88%	2.98%	2.98%	2.56%	2.42%	2.34%	2.40%	2.18%	2.22%
	GOF	1.82	1.74	1.53	1.53	1.49	1.43	1.50	1.50	1.22	1.16	1.12	1.39	1.27	1.31
	ρ_{min}	-0.13	-0.11	-0.20	-0.20	-0.29	-0.26	-0.27	-0.48	-1.17	-0.64	-0.68	-1.06	-0.59	-0.59
	ρ_{max}	0.20	0.16	0.21	0.21	0.32	0.32	0.50	0.47	0.73	0.56	0.61	1.19	0.61	0.61
	χ^2	3.29	3.02			2.21	2.03			1.50	1.34		1.94	1.60	
	λ_{max}		1.200				1.635				7.750			6.015	

Table 2: Similarity index for H ADPs between HAR and MM data (intensity cut-off: $|F| \geq 2\sigma(|F|)$).

	Structure	PT-11(S-Ph)	PT-2(Cl)	PT-8(Br)	PT-10(I)
harmonic	HAR/MM	3.21	13.12	17.65*	3.42
	HAR_{anis}/MM(freeXH)	3.18	n/a	n/a	3.45
anharmonic (n=3)	HAR/MM	3.07	12.87	20.65**	3.52
	HAR_{anis}/MM(freeXH)	3.06	n/a	n/a	3.13
anharmonic (n=3, 4)	HAR/MM	2.22	14.38	29.16**	3.39
	HAR/MM(freeXH)	2.14	n/a	n/a	3.21

* The analysis excludes H2, H3, H8 and H1 for HAR refinement of PT-10(I) due to the presence of zero-positive values.

** The analysis excludes H2, H3, H8 for HAR refinement of PT-10(I) due to the presence of zero-positive values.

Table 3: Bond lengths for PT-2(Cl) (intensity cut-off: $|F| \geq 2\sigma(|F|)$).

		MM			MM(freeXH)			HAR		
	opt	harm	anh, n=3	anh, n=3,4	harm	anh, n=3	anh, n=3,4	harm	anh, n=3	anh, n=3,4
C1 - Cl1	1.7536	1.7287(3)	1.7284(3)	1.7284(3)	1.7287(3)	1.7284(3)	1.7284(3)	1.7290(3)	1.7282(3)	1.7283(3)
C9 - O1	1.2439	1.2392(5)	1.2392(5)	1.2391(5)	1.2392(5)	1.2392(5)	1.2391(5)	1.2381(4)	1.2381(4)	1.2378(3)
C6 - N1	1.3786	1.3762(4)	1.3762(4)	1.3763(4)	1.3762(4)	1.3762(4)	1.3763(4)	1.3765(4)	1.3766(4)	1.3766(3)
C9 - N1	1.3829	1.3765(4)	1.3765(4)	1.3764(4)	1.3765(4)	1.3765(4)	1.3764(4)	1.3768(3)	1.3768(3)	1.3768(3)
C1 - C2	1.3863	1.3834(4)	1.3834(4)	1.3834(4)	1.3834(4)	1.3834(4)	1.3834(4)	1.3828(4)	1.3829(4)	1.3830(4)
C1 - C6	1.4127	1.4081(4)	1.4081(4)	1.4081(4)	1.4081(4)	1.4081(4)	1.4081(4)	1.4080(4)	1.4080(4)	1.4079(4)
C2 - C3	1.4036	1.4032(5)	1.4032(5)	1.4031(5)	1.4032(5)	1.4032(5)	1.4031(5)	1.4032(5)	1.4032(5)	1.4029(4)
C3 - C4	1.3854	1.3844(5)	1.3844(5)	1.3844(5)	1.3844(5)	1.3844(5)	1.3844(5)	1.3841(5)	1.3841(5)	1.3840(5)
C4 - C5	1.4107	1.4071(5)	1.4072(4)	1.4072(4)	1.4072(5)	1.4072(4)	1.4072(4)	1.4071(4)	1.4071(4)	1.4074(4)
C5 - C6	1.4227	1.4138(4)	1.4138(4)	1.4137(4)	1.4138(4)	1.4138(4)	1.4137(4)	1.4133(4)	1.4133(4)	1.4131(4)
C5 - C7	1.4376	1.4368(4)	1.4369(4)	1.4369(4)	1.4369(4)	1.4369(4)	1.4369(4)	1.4369(4)	1.4369(4)	1.4371(4)
C7 - C8	1.3558	1.3533(5)	1.3533(5)	1.3534(5)	1.3533(5)	1.3533(5)	1.3533(5)	1.3526(4)	1.3525(4)	1.3525(4)
C8 - C9	1.4541	1.4539(4)	1.4538(4)	1.4539(4)	1.4538(4)	1.4538(4)	1.4538(4)	1.4544(4)	1.4543(4)	1.4544(4)
C2 - H2	1.0870	1.09(1)	1.09(1)	1.09(1)	1.12(3)	1.12(3)	1.12(3)	1.105(7)	1.105(7)	1.096(7)
C3 - H3	1.0891	1.09(1)	1.09(1)	1.09(1)	1.13(3)	1.13(3)	1.13(3)	1.111(8)	1.110(8)	1.106(8)
C4 - H4	1.0915	1.08(1)	1.08(1)	1.08(1)	1.08(3)	1.08(3)	1.08(3)	1.082(8)	1.080(8)	1.078(7)
C7 - H7	1.0914	1.08(1)	1.08(1)	1.08(1)	1.09(4)	1.09(4)	1.09(4)	1.097(8)	1.098(8)	1.090(8)
C8 - H8	1.0870	1.10(1)	1.10(1)	1.10(1)	1.17(3)	1.16(3)	1.16(3)	1.099(8)	1.097(8)	1.088(8)
N1 - H1	1.0359	1.03(1)	1.03(1)	1.03(1)	1.05(3)	1.06(3)	1.06(3)	1.040(8)	1.042(8)	1.035(8)

Table 4: Bond lengths for PT-2(Cl) (no intensity cut-off).

		MM			MM(freeXH)			HAR		
	opt	harm	anh, n=3	anh, n=3,4	harm	anh, n=3	anh, n=3,4	harm	anh, n=3	anh, n=3,4
C1 - Cl1	1.7536	1.7289(3)	1.7286(3)	1.7287(3)	1.7289(3)	1.7286(3)	1.7286(3)	1.7290(3)	1.7283(3)	1.7283(3)
C9 - O1	1.2439	1.2392(4)	1.2392(4)	1.2391(4)	1.2392(4)	1.2392(4)	1.2391(4)	1.2380(3)	1.2380(3)	1.2378(3)
C6 - N1	1.3786	1.3761(4)	1.3761(4)	1.3762(4)	1.3761(4)	1.3761(4)	1.3761(4)	1.3765(3)	1.3765(3)	1.3765(3)
C9 - N1	1.3829	1.3763(4)	1.3764(4)	1.3763(4)	1.3764(4)	1.3764(4)	1.3763(4)	1.3768(3)	1.3768(3)	1.3768(3)
C1 - C2	1.3863	1.3833(4)	1.3833(4)	1.3833(4)	1.3833(4)	1.3833(4)	1.3834(4)	1.3828(4)	1.3829(4)	1.3830(3)
C1 - C6	1.4127	1.4081(4)	1.4081(4)	1.4081(4)	1.4080(4)	1.4081(4)	1.4080(4)	1.4080(3)	1.4080(3)	1.4079(3)
C2 - C3	1.4036	1.4032(5)	1.4032(5)	1.4032(5)	1.4033(5)	1.4033(5)	1.4032(5)	1.4033(4)	1.4033(4)	1.4029(4)
C3 - C4	1.3854	1.3845(5)	1.3845(5)	1.3846(5)	1.3844(5)	1.3844(5)	1.3845(5)	1.3841(4)	1.3841(4)	1.3840(4)
C4 - C5	1.4107	1.4072(4)	1.4072(4)	1.4073(4)	1.4072(4)	1.4072(4)	1.4073(4)	1.4071(4)	1.4071(4)	1.4073(3)
C5 - C6	1.4227	1.4138(4)	1.4138(4)	1.4137(4)	1.4139(4)	1.4138(4)	1.4138(4)	1.4133(3)	1.4133(3)	1.4132(3)
C5 - C7	1.4376	1.4366(4)	1.4366(4)	1.4367(4)	1.4367(4)	1.4366(4)	1.4367(4)	1.4369(4)	1.4369(4)	1.4371(3)
C7 - C8	1.3558	1.3536(4)	1.3536(4)	1.3536(4)	1.3535(4)	1.3535(4)	1.3536(4)	1.3526(4)	1.3526(4)	1.3525(4)
C8 - C9	1.4541	1.4537(4)	1.4537(4)	1.4537(4)	1.4536(4)	1.4536(4)	1.4537(4)	1.4544(3)	1.4543(3)	1.4544(3)
C2 - H2	1.0870	1.084(8)	1.084(8)	1.084(8)	1.12(2)	1.12(2)	1.12(2)	1.106(7)	1.106(7)	1.096(6)
C3 - H3	1.0891	1.083(8)	1.083(8)	1.083(8)	1.12(2)	1.12(2)	1.12(2)	1.111(7)	1.110(7)	1.105(7)
C4 - H4	1.0915	1.083(7)	1.083(7)	1.083(7)	1.08(3)	1.09(3)	1.09(3)	1.082(7)	1.080(7)	1.078(7)
C7 - H7	1.0914	1.084(7)	1.084(7)	1.083(7)	1.08(3)	1.08(3)	1.08(3)	1.096(7)	1.097(7)	1.091(7)
C8 - H8	1.0870	1.083(9)	1.083(9)	1.083(9)	1.17(2)	1.17(2)	1.16(2)	1.099(7)	1.097(7)	1.088(7)
N1 - H1	1.0359	1.030(6)	1.030(6)	1.030(6)	1.04(3)	1.04(3)	1.04(3)	1.039(8)	1.041(8)	1.035(7)

Table 5: Bond lengths for PT-8(Br) (intensity cut-off: $|F| \geq 2\sigma(|F|)$).

		MM			HAR		
	opt	harm	anh, n=3	anh, n=3,4	harm	anh, n=3	anh, n=3,4
C1 - Br1	1.8713	1.8834(6)	1.8832(6)	1.8832(6)	1.8836(5)	1.8834(5)	1.8834(5)
C9 - O1	1.2422	1.2359(9)	1.2358(9)	1.2357(9)	1.2362(7)	1.2361(7)	1.2361(7)
C6 - N1	1.3837	1.3776(7)	1.3780(8)	1.3781(8)	1.3784(7)	1.3784(7)	1.3784(7)
C9 - N1	1.3824	1.3780(8)	1.3776(7)	1.3776(7)	1.3769(7)	1.3769(7)	1.3769(6)
C1 - C2	1.3785	1.3843(8)	1.3844(8)	1.3843(8)	1.3841(8)	1.3841(8)	1.3841(8)
C1 - C6	1.3987	1.4078(8)	1.4077(8)	1.4077(7)	1.4074(7)	1.4074(7)	1.4074(7)
C2 - C3	1.4002	1.4012(9)	1.4012(9)	1.4012(9)	1.4010(9)	1.4010(9)	1.4009(9)
C3 - C4	1.3842	1.3827(10)	1.3827(10)	1.3827(9)	1.3833(9)	1.3833(9)	1.3834(9)
C4 - C5	1.4086	1.4074(8)	1.4075(8)	1.4077(8)	1.4068(8)	1.4068(8)	1.4069(8)
C5 - C6	1.4212	1.4142(7)	1.4142(7)	1.4142(7)	1.4140(7)	1.4141(7)	1.4140(7)
C5 - C7	1.4372	1.4361(8)	1.4361(8)	1.4361(8)	1.4364(8)	1.4364(8)	1.4365(8)
C7 - C8	1.3550	1.3517(9)	1.3516(9)	1.3517(9)	1.3514(8)	1.3514(8)	1.3514(8)
C8 - C9	1.4549	1.4544(8)	1.4545(8)	1.4544(7)	1.4548(8)	1.4548(7)	1.4547(7)
C2 - H2	1.0868	1.083(1)	1.083(1)	1.083(1)	1.08(1)	1.08(1)	1.08(1)
C3 - H3	1.0891	1.083(1)	1.083(1)	1.083(1)	1.10(1)	1.10(1)	1.10(1)
C4 - H4	1.0910	1.083(1)	1.083(1)	1.083(1)	1.05(1)	1.05(1)	1.05(1)
C7 - H7	1.0909	1.083(1)	1.083(1)	1.083(1)	1.08(2)	1.08(2)	1.08(1)
C8 - H8	1.0878	1.083(1)	1.083(1)	1.083(1)	1.05(2)	1.05(2)	1.05(2)
N1 - H1	1.0338	1.030(1)	1.030(1)	1.030(1)	1.02(1)	1.02(1)	1.02(1)

Table 6: Bond lengths for PT-8(Br) (no intensity cut-off).

		MM			HAR		
	opt	harm	anh, n=3	anh, n=3,4	harm	anh, n=3	anh, n=3,4
C1 - Br1	1.8713	1.8836(6)	1.8834(6)	1.8834(6)	1.8836(5)	1.8834(5)	1.8834(5)
C9 - O1	1.2422	1.2352(9)	1.2351(9)	1.2351(9)	1.2361(6)	1.2360(6)	1.2360(6)
C6 - N1	1.3837	1.3783(8)	1.3783(8)	1.3785(8)	1.3784(6)	1.3784(6)	1.3784(6)
C9 - N1	1.3824	1.3777(7)	1.3777(7)	1.3777(7)	1.3769(6)	1.3769(6)	1.3769(6)
C1 - C2	1.3785	1.3838(8)	1.3839(8)	1.3837(8)	1.3841(7)	1.3841(7)	1.3841(7)
C1 - C6	1.3987	1.4078(8)	1.4078(8)	1.4077(7)	1.4074(6)	1.4073(6)	1.4073(6)
C2 - C3	1.4002	1.4014(9)	1.4014(9)	1.4014(9)	1.4009(8)	1.4009(8)	1.4008(8)
C3 - C4	1.3842	1.383(1)	1.383(1)	1.3827(9)	1.3833(8)	1.3833(8)	1.3833(8)
C4 - C5	1.4086	1.4076(8)	1.4077(8)	1.4078(8)	1.4068(7)	1.4069(7)	1.4069(7)
C5 - C6	1.4212	1.4140(7)	1.4141(7)	1.4141(7)	1.4140(6)	1.4140(6)	1.4140(6)
C5 - C7	1.4372	1.4358(8)	1.4358(8)	1.4359(8)	1.4363(7)	1.4363(7)	1.4364(7)
C7 - C8	1.3550	1.3517(9)	1.3517(9)	1.3517(9)	1.3515(7)	1.3515(7)	1.3514(7)
C8 - C9	1.4549	1.4547(8)	1.4547(8)	1.4546(7)	1.4548(7)	1.4549(7)	1.4548(7)
C2 - H2	1.0868	1.083(9)	1.084(6)	1.083(8)	1.08(1)	1.08(1)	1.08(1)
C3 - H3	1.0891	1.083(7)	1.083(7)	1.083(7)	1.10(1)	1.10(1)	1.10(1)
C4 - H4	1.0910	1.084(6)	1.084(6)	1.083(6)	1.05(1)	1.05(1)	1.05(1)
C7 - H7	1.0909	1.083(6)	1.083(6)	1.083(6)	1.08(1)	1.08(1)	1.08(1)
C8 - H8	1.0878	1.08(1)	1.08(1)	1.082(9)	1.05(1)	1.05(1)	1.05(1)
N1 - H1	1.0338	1.030(6)	1.031(6)	1.030(6)	1.02(1)	1.02(1)	1.02(1)

Table 7: Bond lengths for PT-10(I) (intensity cut-off: $|F| \geq 2\sigma(|F|)$).

		MM			HAR		
	opt	harm	anh, n=3	anh, n=3,4	harm	anh, n=3	anh, n=3,4
C1 - I1	2.1138	2.0872(9)	2.0845(8)	2.0843(8)	2.0881(8)	2.0857(7)	2.0858(7)
C9 - O1	1.2424	1.236(2)	1.236(2)	1.236(2)	1.231(1)	1.232(1)	1.231(1)
C6 - N1	1.3784	1.376(1)	1.376(1)	1.376(1)	1.376(1)	1.376(1)	1.376(1)
C9 - N1	1.3835	1.373(1)	1.373(1)	1.373(1)	1.374(1)	1.374(1)	1.374(1)
C1 - C2	1.3892	1.387(1)	1.387(1)	1.387(1)	1.385(1)	1.385(1)	1.385(1)
C1 - C6	1.4121	1.404(1)	1.404(1)	1.404(1)	1.405(1)	1.405(1)	1.405(1)
C2 - C3	1.4043	1.403(2)	1.403(1)	1.403(1)	1.401(1)	1.401(1)	1.401(1)
C3 - C4	1.3863	1.380(2)	1.380(2)	1.380(2)	1.377(2)	1.378(1)	1.379(1)
C4 - C5	1.4097	1.404(2)	1.404(1)	1.404(1)	1.404(1)	1.404(1)	1.404(1)
C5 - C6	1.4192	1.411(1)	1.411(1)	1.411(1)	1.410(1)	1.409(1)	1.409(1)
C5 - C7	1.4401	1.435(2)	1.435(1)	1.435(1)	1.435(1)	1.434(1)	1.435(1)
C7 - C8	1.3576	1.354(2)	1.356(2)	1.356(2)	1.354(2)	1.355(1)	1.355(1)
C8 - C9	1.4547	1.451(1)	1.450(1)	1.450(1)	1.450(1)	1.451(1)	1.451(1)
C2 - H2	1.0879	1.083(1)	1.083(1)	1.083(1)	1.08(2)	1.09(2)	1.09(2)
C3 - H3	1.0906	1.083(1)	1.083(1)	1.083(1)	1.00(2)	1.05(2)	1.04(2)
C4 - H4	1.0921	1.083(1)	1.083(1)	1.083(1)	1.09(2)	1.08(2)	1.08(2)
C7 - H7	1.0888	1.083(1)	1.083(1)	1.083(1)	1.01(2)	1.02(2)	1.02(2)
C8 - H8	1.0895	1.083(1)	1.083(1)	1.083(1)	1.05(2)	1.07(2)	1.07(2)
N1 - H1	1.0167	1.030(1)	1.030(1)	1.030(1)	0.92(2)	0.89(2)	0.88(2)

Table 8: Bond lengths for PT-10(I) (no intensity cut-off).

		MM			HAR		
	opt	harm	anh, n=3	anh, n=3,4	harm	anh, n=3	anh, n=3,4
C1 - I1	2.1138	2.0862(9)	2.0833(8)	2.0833(8)	2.0881(8)	2.0857(7)	2.0858(7)
C9 - O1	1.2424	1.235(2)	1.236(2)	1.236(2)	1.231(1)	1.232(1)	1.231(1)
C6 - N1	1.3784	1.377(1)	1.377(1)	1.377(1)	1.376(1)	1.3759(9)	1.3757(9)
C9 - N1	1.3835	1.374(1)	1.374(1)	1.374(1)	1.374(1)	1.374(1)	1.3736(9)
C1 - C2	1.3892	1.387(1)	1.388(1)	1.388(1)	1.385(1)	1.385(1)	1.385(1)
C1 - C6	1.4121	1.404(1)	1.404(1)	1.404(1)	1.405(1)	1.405(1)	1.405(1)
C2 - C3	1.4043	1.403(2)	1.402(1)	1.402(1)	1.402(1)	1.401(1)	1.401(1)
C3 - C4	1.3863	1.379(2)	1.380(2)	1.380(2)	1.377(1)	1.378(1)	1.379(1)
C4 - C5	1.4097	1.405(2)	1.405(1)	1.405(1)	1.404(1)	1.404(1)	1.404(1)
C5 - C6	1.4192	1.411(1)	1.411(1)	1.411(1)	1.409(1)	1.409(1)	1.4093(9)
C5 - C7	1.4401	1.436(2)	1.435(1)	1.435(1)	1.435(1)	1.434(1)	1.434(1)
C7 - C8	1.3576	1.353(2)	1.354(2)	1.354(2)	1.354(1)	1.355(1)	1.355(1)
C8 - C9	1.4547	1.452(1)	1.452(1)	1.451(1)	1.450(1)	1.451(1)	1.451(1)
C2 - H2	1.0879	1.082(9)	1.082(8)	1.082(8)	1.07(2)	1.09(2)	1.09(2)
C3 - H3	1.0906	1.083(8)	1.085(9)	1.083(9)	1.00(2)	1.04(2)	1.03(2)
C4 - H4	1.0921	1.08(1)	1.08(1)	1.08(1)	1.09(2)	1.08(2)	1.08(2)
C7 - H7	1.0888	1.08(1)	1.08(1)	1.08(1)	1.02(2)	1.03(2)	1.02(2)
C8 - H8	1.0895	1.08(1)	1.08(1)	1.08(1)	1.05(2)	1.07(2)	1.07(2)
N1 - H1	1.0167	1.03(1)	1.03(1)	1.03(1)	0.92(2)	0.89(2)	0.88(2)

Table 9: Bond lengths for PT-11(S-Ph) (intensity cut-off: $|F| \geq 2\sigma(|F|)$).

		MM			MM(freeXH)			HAR		
	opt	harm	anh, n=3	anh, n=3,4	harm	anh, n=3	anh, n=3,4	harm	anh, n=3	anh, n=3,4
C8 - S1	1.7895	1.7667(1)	1.7661(2)	1.7661(2)	1.7667(1)	1.7661(2)	1.7661(2)	1.7672(2)	1.7665(2)	1.7666(2)
C10 - S1	1.7980	1.7742(2)	1.7728(2)	1.7728(2)	1.7742(2)	1.7728(2)	1.7728(2)	1.7747(2)	1.7733(2)	1.7733(2)
C1 - O1	1.2446	1.2390(2)	1.2390(2)	1.2390(2)	1.2390(2)	1.2390(2)	1.2390(2)	1.2382(2)	1.2382(2)	1.2382(2)
C1 - N1	1.3844	1.3775(2)	1.3776(2)	1.3776(2)	1.3775(2)	1.3775(2)	1.3776(2)	1.3777(2)	1.3777(2)	1.3777(2)
C9 - N1	1.3815	1.3818(2)	1.3819(2)	1.3818(2)	1.3818(2)	1.3818(2)	1.3818(2)	1.3821(2)	1.3821(2)	1.3821(2)
C1 - C2	1.4575	1.4585(2)	1.4585(2)	1.4585(2)	1.4585(2)	1.4585(2)	1.4585(2)	1.4586(2)	1.4585(2)	1.4586(2)
C2 - C3	1.3577	1.3563(3)	1.3563(3)	1.3563(3)	1.3563(2)	1.3563(3)	1.3563(3)	1.3557(3)	1.3558(3)	1.3558(3)
C3 - C4	1.4381	1.4383(2)	1.4382(2)	1.4382(2)	1.4382(2)	1.4382(2)	1.4382(2)	1.4384(3)	1.4384(3)	1.4384(3)
C4 - C5	1.4091	1.4069(2)	1.4069(2)	1.4069(2)	1.4069(2)	1.4069(2)	1.4069(2)	1.4066(2)	1.4066(2)	1.4066(2)
C4 - C9	1.4230	1.4141(2)	1.4142(2)	1.4142(2)	1.4141(2)	1.4142(2)	1.4142(2)	1.4142(2)	1.4142(2)	1.4142(2)
C5 - C6	1.3866	1.3870(3)	1.3869(3)	1.3870(3)	1.3870(3)	1.3869(3)	1.3870(3)	1.3860(3)	1.3860(3)	1.3860(3)
C6 - C7	1.4017	1.4008(3)	1.4009(2)	1.4009(2)	1.4008(2)	1.4009(2)	1.4009(2)	1.4007(3)	1.4008(3)	1.4007(3)
C7 - C8	1.3933	1.3923(2)	1.3922(2)	1.3922(2)	1.3923(2)	1.3922(2)	1.3922(2)	1.3921(2)	1.3920(2)	1.3920(2)
C8 - C9	1.4184	1.4147(2)	1.4147(2)	1.4147(2)	1.4146(2)	1.4147(2)	1.4147(2)	1.4141(2)	1.4142(2)	1.4142(2)
C10 - C11	1.4013	1.3978(2)	1.3979(2)	1.3979(2)	1.3978(2)	1.3979(2)	1.3978(2)	1.3977(2)	1.3978(2)	1.3978(2)
C10 - C15	1.4013	1.3997(2)	1.3997(2)	1.3997(2)	1.3997(2)	1.3997(2)	1.3997(2)	1.3990(2)	1.3990(2)	1.3990(2)
C11 - C12	1.3967	1.3951(3)	1.3951(3)	1.3951(3)	1.3951(3)	1.3951(3)	1.3951(3)	1.3951(3)	1.3950(3)	1.3950(3)
C12 - C13	1.3983	1.3989(3)	1.3988(3)	1.3988(3)	1.3989(3)	1.3989(3)	1.3989(3)	1.3983(4)	1.3982(3)	1.3982(3)
C13 - C14	1.3983	1.3966(3)	1.3966(3)	1.3966(3)	1.3966(3)	1.3966(3)	1.3967(3)	1.3960(3)	1.3962(3)	1.3962(3)
C14 - C15	1.3973	1.3948(3)	1.3948(3)	1.3948(3)	1.3948(3)	1.3949(3)	1.3949(3)	1.3947(3)	1.3948(3)	1.3947(3)
C2 - H2	1.0888	1.083(2)	1.083(2)	1.083(2)	1.09(2)	1.08(2)	1.08(2)	1.075(7)	1.076(7)	1.073(7)
C3 - H3	1.0936	1.083(2)	1.083(2)	1.083(2)	1.11(2)	1.11(2)	1.11(2)	1.084(7)	1.084(7)	1.084(7)
C5 - H5	1.0914	1.083(2)	1.083(2)	1.083(2)	1.05(2)	1.06(2)	1.06(2)	1.073(7)	1.074(7)	1.074(7)
C6 - H6	1.0885	1.083(2)	1.083(2)	1.083(2)	1.07(2)	1.07(2)	1.07(2)	1.073(7)	1.072(7)	1.071(7)
C7 - H7	1.0897	1.083(2)	1.083(2)	1.083(2)	1.10(2)	1.11(2)	1.11(2)	1.080(7)	1.083(7)	1.081(7)
C11 - H11	1.0912	1.083(2)	1.083(2)	1.083(2)	1.14(2)	1.14(2)	1.14(2)	1.109(7)	1.109(7)	1.108(7)
C12 - H12	1.0891	1.083(2)	1.083(2)	1.083(2)	1.12(2)	1.12(2)	1.12(2)	1.078(7)	1.079(6)	1.077(6)
C13 - H13	1.0898	1.083(2)	1.083(2)	1.083(2)	1.10(2)	1.10(2)	1.10(2)	1.068(7)	1.069(7)	1.068(7)
C14 - H14	1.0903	1.083(2)	1.083(2)	1.083(2)	1.11(2)	1.12(2)	1.12(2)	1.090(8)	1.090(8)	1.089(8)
C15 - H15	1.0871	1.083(2)	1.083(2)	1.083(2)	1.05(2)	1.05(2)	1.05(2)	1.081(6)	1.082(6)	1.081(6)
N1 - H1	1.0311	1.030(2)	1.030(2)	1.030(2)	0.98(2)	0.99(2)	0.98(2)	1.041(6)	1.042(6)	1.042(6)

Table 10: Bond lengths for PT-11(S-Ph) (no intensity cut-off).

		MM			MM(freeXH)			HAR		
	opt	harm	anh, n=3	anh, n=3,4	harm	anh, n=3	anh, n=3,4	harm	anh, n=3	anh, n=3,4
C8 - S1	1.7895	1.7667(2)	1.7662(2)	1.7662(2)	1.7667(2)	1.7662(2)	1.7662(2)	1.7672(1)	1.7665(2)	1.7666(2)
C10 - S1	1.7980	1.7741(2)	1.7729(2)	1.7729(2)	1.7742(2)	1.7729(2)	1.7729(2)	1.7747(2)	1.7733(2)	1.7734(2)
C1 - O1	1.2446	1.2389(3)	1.2389(3)	1.2389(3)	1.2389(3)	1.2389(3)	1.2389(3)	1.2382(2)	1.2382(2)	1.2382(2)
C1 - N1	1.3844	1.3776(2)	1.3777(2)	1.3777(2)	1.3776(2)	1.3776(2)	1.3777(2)	1.3777(2)	1.3777(2)	1.3777(2)
C9 - N1	1.3815	1.3818(2)	1.3818(2)	1.3818(2)	1.3818(2)	1.3818(2)	1.3817(2)	1.3821(2)	1.3821(2)	1.3821(2)
C1 - C2	1.4575	1.4584(2)	1.4583(2)	1.4583(2)	1.4584(2)	1.4583(2)	1.4583(2)	1.4586(2)	1.4585(2)	1.4586(2)
C2 - C3	1.3577	1.3566(3)	1.3566(3)	1.3566(3)	1.3566(3)	1.3566(3)	1.3566(3)	1.3558(3)	1.3558(3)	1.3558(3)
C3 - C4	1.4381	1.4383(3)	1.4383(3)	1.4383(3)	1.4384(3)	1.4383(3)	1.4383(3)	1.4384(2)	1.4383(2)	1.4384(2)
C4 - C5	1.4091	1.4068(3)	1.4068(2)	1.4068(2)	1.4068(3)	1.4068(2)	1.4068(2)	1.4066(2)	1.4066(2)	1.4066(2)
C4 - C9	1.4230	1.4141(2)	1.4142(2)	1.4142(2)	1.4141(2)	1.4142(2)	1.4142(2)	1.4142(2)	1.4142(2)	1.4142(2)
C5 - C6	1.3866	1.3867(3)	1.3866(3)	1.3866(3)	1.3867(3)	1.3866(3)	1.3866(3)	1.3860(3)	1.3860(3)	1.3860(3)
C6 - C7	1.4017	1.4005(3)	1.4005(3)	1.4005(3)	1.4004(3)	1.4005(3)	1.4005(3)	1.4007(3)	1.4008(3)	1.4008(2)
C7 - C8	1.3933	1.3926(2)	1.3925(2)	1.3925(2)	1.3926(2)	1.3925(2)	1.3925(2)	1.3921(2)	1.3920(2)	1.3920(2)
C8 - C9	1.4184	1.4145(2)	1.4146(2)	1.4146(2)	1.4146(2)	1.4146(2)	1.4146(2)	1.4141(2)	1.4142(2)	1.4142(2)
C10 - C11	1.4013	1.3979(2)	1.3979(2)	1.3979(2)	1.3979(2)	1.3979(2)	1.3979(2)	1.3977(2)	1.3977(2)	1.3977(2)
C10 - C15	1.4013	1.3998(2)	1.3998(2)	1.3998(2)	1.3998(2)	1.3998(2)	1.3998(2)	1.3989(2)	1.3989(2)	1.3989(2)
C11 - C12	1.3967	1.3954(3)	1.3954(3)	1.3954(3)	1.3954(3)	1.3954(3)	1.3954(3)	1.3951(3)	1.3950(3)	1.3950(3)
C12 - C13	1.3983	1.3990(4)	1.3990(4)	1.3990(4)	1.3990(4)	1.3990(4)	1.3990(4)	1.3983(3)	1.3982(3)	1.3982(3)
C13 - C14	1.3983	1.3962(3)	1.3962(3)	1.3962(3)	1.3962(4)	1.3963(3)	1.3963(3)	1.3960(3)	1.3962(3)	1.3962(3)
C14 - C15	1.3973	1.3949(3)	1.3949(3)	1.3949(3)	1.3949(3)	1.3949(3)	1.3949(3)	1.3948(3)	1.3948(3)	1.3948(3)
C2 - H2	1.0888	1.083(5)	1.083(5)	1.083(5)	1.12(2)	1.11(2)	1.11(2)	1.075(7)	1.076(7)	1.074(7)
C3 - H3	1.0936	1.083(6)	1.083(6)	1.083(6)	1.09(2)	1.09(2)	1.09(2)	1.084(6)	1.084(6)	1.084(6)
C5 - H5	1.0914	1.082(6)	1.083(6)	1.083(6)	1.06(2)	1.06(2)	1.06(2)	1.073(6)	1.075(6)	1.074(6)
C6 - H6	1.0885	1.084(6)	1.083(6)	1.083(6)	1.09(2)	1.09(2)	1.09(2)	1.073(7)	1.072(6)	1.071(7)
C7 - H7	1.0897	1.083(4)	1.083(4)	1.083(4)	1.11(2)	1.11(2)	1.11(2)	1.080(7)	1.082(7)	1.081(7)
C11 - H11	1.0912	1.083(6)	1.084(6)	1.084(6)	1.13(2)	1.11(2)	1.13(2)	1.109(7)	1.110(7)	1.109(7)
C12 - H12	1.0891	1.083(7)	1.083(7)	1.083(7)	1.10(2)	1.102(4)	1.10(1)	1.078(6)	1.079(6)	1.077(6)
C13 - H13	1.0898	1.08(1)	1.08(1)	1.08(1)	1.09(3)	1.10(2)	1.10(2)	1.069(7)	1.069(7)	1.068(7)
C14 - H14	1.0903	1.083(7)	1.083(7)	1.083(7)	1.11(3)	1.12(3)	1.12(3)	1.090(7)	1.090(7)	1.089(7)
C15 - H15	1.0871	1.083(6)	1.083(6)	1.083(6)	1.08(2)	1.08(1)	1.08(1)	1.081(6)	1.082(6)	1.081(6)
N1 - H1	1.0311	1.029(5)	1.030(4)	1.030(4)	0.99(2)	1.00(2)	1.00(2)	1.041(6)	1.042(6)	1.042(6)

Table 11: Angles for PT-2(Cl) (intensity cut-off: $|F| \geq 2\sigma(|F|)$).

		MM			MM(freeXH)			HAR		
	opt	harm	anh, n=3	anh, n=3,4	harm	anh, n=3	anh, n=3,4	harm	anh, n=3	anh, n=3,4
C1 C2 C3	120.56	120.30(4)	120.30(4)	120.30(4)	120.30(4)	120.30(4)	120.30(4)	120.29(3)	120.29(3)	120.29(3)
C1 C2 H2	118.73	119.9(8)	119.9(8)	119.8(8)	119.7(10)	119.7(10)	119.7(10)	120.6(4)	120.5(4)	120.2(4)
C2 C3 C4	119.57	119.65(4)	119.65(4)	119.65(4)	119.65(4)	119.65(4)	119.65(4)	119.65(3)	119.65(3)	119.66(3)
C2 C3 H3	119.82	122.4(9)	122.5(9)	122.5(9)	122(1)	123(1)	123(1)	121.3(4)	121.3(4)	121.2(4)
C3 C2 H2	120.71	119.8(8)	119.8(8)	119.9(8)	120(1)	120(1)	120(1)	119.1(4)	119.2(4)	119.5(4)
C3 C4 H4	120.31	118.6(10)	118.7(10)	118.7(9)	119(1)	119(1)	119(1)	120.0(4)	120.0(4)	120.0(4)
C4 C3 H3	120.61	117.9(8)	117.8(8)	117.8(8)	118(1)	118(1)	118(1)	119.1(4)	119.0(4)	119.1(4)
C5 C4 C3	120.59	120.54(4)	120.54(4)	120.54(4)	120.54(4)	120.54(4)	120.53(4)	120.53(3)	120.53(3)	120.52(3)
C5 C4 H4	119.11	120.8(10)	120.7(10)	120.8(9)	121(1)	121(1)	121(1)	119.4(4)	119.5(4)	119.5(4)
C5 C6 C1	117.81	118.24(3)	118.24(3)	118.24(3)	118.24(3)	118.24(3)	118.24(3)	118.23(2)	118.24(2)	118.24(2)
C5 C7 C8	121.42	121.13(3)	121.14(3)	121.13(3)	121.13(3)	121.13(3)	121.13(3)	121.15(3)	121.15(3)	121.14(2)
C5 C7 H7	119.11	119.7(9)	119.6(9)	119.5(9)	120(1)	120(1)	120(1)	119.1(5)	119.2(4)	119.2(4)
C6 C1 C2	121.23	121.16(3)	121.16(3)	121.16(3)	121.16(3)	121.16(3)	121.16(3)	121.17(3)	121.17(3)	121.17(2)
C6 C5 C4	120.22	120.10(3)	120.10(3)	120.10(3)	120.10(3)	120.10(3)	120.10(3)	120.11(3)	120.11(3)	120.11(2)
C6 C5 C7	117.45	117.65(3)	117.65(3)	117.65(3)	117.65(3)	117.65(3)	117.65(3)	117.64(2)	117.64(2)	117.64(2)
C6 N1 C9	124.34	123.93(3)	123.92(3)	123.92(3)	123.92(3)	123.92(3)	123.92(3)	123.89(2)	123.89(2)	123.90(2)
C6 N1 H1	121.09	121.2(8)	121.2(8)	121.1(8)	121(1)	121(1)	121(1)	122.1(5)	122.0(5)	121.5(5)
C7 C5 C4	122.32	122.25(4)	122.25(4)	122.25(4)	122.25(4)	122.25(4)	122.25(4)	122.25(3)	122.25(3)	122.25(2)
C7 C8 H8	120.65	121.1(9)	121.2(9)	121.1(9)	121(1)	121(1)	121(1)	122.6(4)	122.5(4)	122.5(4)
C8 C7 H7	119.48	119.2(9)	119.2(9)	119.3(9)	119(1)	119(1)	119(1)	119.7(5)	119.6(4)	119.6(4)
C9 C8 C7	121.31	121.15(3)	121.15(3)	121.15(3)	121.16(3)	121.15(3)	121.15(3)	121.17(3)	121.17(3)	121.18(2)
C9 C8 H8	118.04	117.7(8)	117.7(8)	117.8(8)	118(1)	118(1)	118(1)	116.2(4)	116.3(4)	116.3(4)
C9 N1 H1	114.53	114.8(7)	114.8(7)	114.9(7)	115(1)	115(1)	115(1)	114.0(5)	114.0(5)	114.5(5)
Cl1 C1 C2	118.80	119.37(3)	119.37(3)	119.37(3)	119.37(3)	119.37(3)	119.37(3)	119.36(2)	119.37(2)	119.37(2)
Cl1 C1 C6	119.97	119.47(3)	119.47(3)	119.47(3)	119.47(3)	119.47(3)	119.47(3)	119.47(2)	119.46(2)	119.46(2)
N1 C6 C1	122.69	121.96(3)	121.95(3)	121.95(3)	121.96(3)	121.95(3)	121.95(3)	121.93(2)	121.93(2)	121.93(2)
N1 C6 C5	119.50	119.80(3)	119.80(3)	119.80(3)	119.80(3)	119.80(3)	119.80(3)	119.84(2)	119.84(2)	119.83(2)
N1 C9 C8	115.97	116.34(3)	116.34(3)	116.34(3)	116.33(3)	116.34(3)	116.34(3)	116.31(2)	116.31(2)	116.30(2)
N1 C9 O1	120.97	121.07(3)	121.07(3)	121.07(3)	121.07(3)	121.07(3)	121.07(3)	121.08(3)	121.08(3)	121.08(2)
O1 C9 C8	123.07	122.59(4)	122.59(4)	122.59(4)	122.60(4)	122.60(4)	122.59(4)	122.61(3)	122.61(3)	122.61(2)

Table 12: Angles for PT-2(Cl) (no intensity cut-off).

		MM			MM(freeXH)			HAR		
	opt	harm	anh, n=3	anh, n=3,4	harm	anh, n=3	anh, n=3,4	harm	anh, n=3	anh, n=3,4
C1 C2 C3	120.56	120.29(3)	120.30(3)	120.29(2)	120.29(3)	120.29(3)	120.29(3)	120.29(2)	120.29(2)	120.29(2)
C1 C2 H2	118.73	119.2(5)	119.2(5)	119.3(5)	119.1(8)	119.1(8)	119.2(8)	120.5(4)	120.5(4)	120.2(3)
C2 C3 C4	119.57	119.65(3)	119.65(3)	119.65(3)	119.66(3)	119.65(3)	119.66(3)	119.65(2)	119.65(2)	119.66(2)
C2 C3 H3	119.82	122.5(6)	122.6(6)	122.6(6)	122.(1)	123.(1)	123.(1)	121.3(4)	121.4(4)	121.2(4)
C3 C2 H2	120.71	120.5(5)	120.5(5)	120.4(5)	120.6(9)	120.6(9)	120.6(9)	119.2(4)	119.2(4)	119.5(3)
C3 C4 H4	120.31	118.7(7)	118.8(7)	118.8(7)	119.(1)	119.(1)	119.(1)	120.0(4)	120.0(4)	120.1(4)
C4 C3 H3	120.61	117.9(6)	117.8(6)	117.7(6)	117.9(9)	117.8(9)	117.8(9)	119.0(4)	119.0(4)	119.1(4)
C5 C4 C3	120.59	120.53(3)	120.52(3)	120.52(3)	120.53(3)	120.53(3)	120.52(3)	120.53(2)	120.53(2)	120.52(2)
C5 C4 H4	119.11	120.8(7)	120.6(7)	120.7(7)	121.(1)	121.(1)	121.(1)	119.4(4)	119.5(4)	119.4(4)
C5 C6 C1	117.81	118.23(2)	118.23(2)	118.23(2)	118.23(2)	118.23(2)	118.23(2)	118.23(2)	118.23(2)	118.24(2)
C5 C7 C8	121.42	121.13(2)	121.13(2)	121.13(2)	121.12(2)	121.13(2)	121.12(2)	121.15(2)	121.15(2)	121.14(2)
C5 C7 H7	119.11	119.6(6)	119.5(6)	119.4(6)	120.(1)	120.(1)	119.(1)	119.1(4)	119.2(4)	119.2(4)
C6 C1 C2	121.23	121.17(2)	121.17(2)	121.17(2)	121.17(2)	121.17(2)	121.17(2)	121.17(2)	121.17(2)	121.17(2)
C6 C5 C4	120.22	120.11(2)	120.11(2)	120.11(2)	120.11(2)	120.11(2)	120.11(2)	120.11(2)	120.11(2)	120.11(2)
C6 C5 C7	117.45	117.65(2)	117.65(2)	117.66(2)	117.65(2)	117.65(2)	117.65(2)	117.64(2)	117.64(2)	117.64(2)
C6 N1 C9	124.34	123.93(2)	123.93(2)	123.93(2)	123.93(2)	123.93(2)	123.92(2)	123.89(2)	123.89(2)	123.90(2)
C6 N1 H1	121.09	121.5(5)	121.5(5)	121.4(5)	121.(1)	121.(1)	121.(1)	122.1(4)	122.0(4)	121.5(4)
C7 C5 C4	122.32	122.24(2)	122.23(2)	122.23(2)	122.24(2)	122.24(2)	122.23(2)	122.25(2)	122.25(2)	122.25(2)
C7 C8 H8	120.65	121.2(5)	121.2(5)	121.1(5)	121.4(10)	121.5(10)	121.4(10)	122.6(4)	122.6(3)	122.7(3)
C8 C7 H7	119.48	119.3(6)	119.4(6)	119.4(6)	119.(1)	119.(1)	119.(1)	119.7(4)	119.6(4)	119.6(4)
C9 C8 C7	121.31	121.15(2)	121.14(2)	121.14(2)	121.16(2)	121.15(2)	121.15(2)	121.17(2)	121.17(2)	121.18(2)
C9 C8 H8	118.04	117.7(5)	117.6(5)	117.7(5)	117.4(8)	117.4(8)	117.4(8)	116.2(4)	116.3(3)	116.2(3)
C9 N1 H1	114.53	114.6(5)	114.5(5)	114.6(5)	114.5(10)	114.5(10)	114.6(10)	113.9(4)	114.0(4)	114.5(4)
CL1 C1 C2	118.80	119.36(2)	119.37(2)	119.37(2)	119.36(2)	119.37(2)	119.36(2)	119.36(2)	119.36(2)	119.37(2)
CL1 C1 C6	119.97	119.463(18)	119.459(18)	119.460(18)	119.465(18)	119.462(18)	119.462(18)	119.47(2)	119.46(2)	119.46(2)
N1 C6 C1	122.69	121.97(2)	121.97(2)	121.97(2)	121.97(2)	121.97(2)	121.96(2)	121.93(2)	121.93(2)	121.93(2)
N1 C6 C5	119.50	119.80(2)	119.80(2)	119.80(2)	119.80(2)	119.80(2)	119.80(2)	119.84(2)	119.84(2)	119.83(2)
N1 C9 C8	115.97	116.34(2)	116.34(2)	116.35(2)	116.34(2)	116.34(2)	116.34(2)	116.31(2)	116.31(2)	116.30(2)
N1 C9 O1	120.97	121.08(3)	121.08(3)	121.08(2)	121.08(3)	121.08(3)	121.08(2)	121.08(2)	121.08(2)	121.08(2)
O1 C9 C8	123.07	122.58(3)	122.58(3)	122.58(3)	122.58(3)	122.58(3)	122.58(3)	122.61(2)	122.61(2)	122.61(2)

Table 13: Angles for PT-8(Br) (intensity cut-off: $|F| \geq 2\sigma(|F|)$).

		MM			HAR		
	opt	harm	anh, n=3	anh, n=3,4	harm	anh, n=3	anh, n=3,4
Br1 C1 C2	118.37	118.90(5)	118.89(5)	118.90(5)	118.90(4)	118.89(4)	118.89(4)
Br1 C1 C6	121.39	119.95(5)	119.95(5)	119.95(5)	119.94(4)	119.94(4)	119.94(4)
C1 C2 C3	121.37	120.47(7)	120.46(7)	120.47(7)	120.47(5)	120.46(5)	120.47(5)
C1 C2 H2	119.79	119.7(6)	119.5(6)	119.5(6)	117.9(8)	117.8(8)	117.7(8)
C2 C3 C4	119.47	119.50(7)	119.51(7)	119.50(7)	119.48(5)	119.48(5)	119.48(5)
C2 C3 H3	120.15	118(1)	118(1)	118(1)	121.3(8)	121.3(8)	121.4(8)
C3 C2 H2	118.83	119.8(6)	120.0(6)	120.0(6)	121.6(8)	121.7(8)	121.8(8)
C3 C4 H4	120.57	120(1)	120(1)	120(1)	121.5(9)	121.4(9)	121.4(9)
C4 C3 H3	120.37	122(1)	122(1)	122(1)	119.1(8)	119.0(8)	118.9(8)
C5 C4 C3	120.14	120.63(8)	120.63(7)	120.63(7)	120.64(5)	120.63(5)	120.63(5)
C5 C4 H4	119.29	119(1)	119(1)	119(1)	117.9(9)	118.0(9)	117.9(9)
C5 C6 C1	118.89	118.06(5)	118.07(5)	118.07(5)	118.06(5)	118.07(5)	118.06(5)
C5 C6 N1	118.53	119.49(6)	119.49(6)	119.48(6)	119.47(5)	119.47(5)	119.47(4)
C5 C7 C8	121.61	121.21(6)	121.22(6)	121.21(6)	121.21(5)	121.21(5)	121.21(5)
C5 C7 H7	118.71	118(1)	118(1)	118.4(10)	119.6(9)	119.6(9)	119.7(8)
C6 C1 C2	120.25	121.15(7)	121.15(7)	121.15(6)	121.16(5)	121.17(5)	121.16(5)
C6 C5 C4	119.88	120.17(6)	120.17(6)	120.16(6)	120.18(5)	120.18(5)	120.18(5)
C6 C5 C7	118.02	117.79(6)	117.79(6)	117.80(6)	117.83(5)	117.82(5)	117.82(5)
C6 N1 C9	124.93	124.14(5)	124.14(5)	124.15(5)	124.10(4)	124.11(4)	124.10(4)
C6 N1 H1	121.19	120.6(8)	120.7(8)	120.9(8)	122.5(9)	122.6(9)	122.6(9)
C7 C5 C4	122.09	122.03(7)	122.03(7)	122.03(6)	121.99(5)	122.00(5)	121.99(5)
C7 C8 H8	121.11	123.2(7)	123.3(7)	123.1(7)	119.5(8)	119.7(8)	119.5(8)
C8 C7 H7	119.68	120(1)	120(1)	120.3(10)	119.2(9)	119.1(9)	119.1(8)
C8 C9 O1	122.86	122.62(7)	122.62(7)	122.63(6)	122.61(5)	122.60(5)	122.61(5)
C9 C8 C7	120.94	121.17(6)	121.17(6)	121.18(6)	121.11(5)	121.11(5)	121.12(5)
C9 C8 H8	117.94	115.6(7)	115.6(7)	115.7(7)	119.3(8)	119.2(8)	119.4(8)
C9 N1 H1	113.86	115.2(8)	115.1(8)	114.9(8)	113.2(9)	113.2(9)	113.2(8)
N1 C6 C1	122.58	122.44(7)	122.44(6)	122.45(6)	122.46(5)	122.46(4)	122.46(4)
N1 C9 C8	115.96	116.19(6)	116.19(6)	116.18(6)	116.27(5)	116.27(5)	116.27(5)
N1 C9 O1	121.19	121.19(6)	121.19(6)	121.19(6)	121.13(5)	121.13(5)	121.13(5)

Table 14: Angles for PT-8(Br) (no intensity cut-off).

		MM			HAR		
	opt	harm	anh, n=3	anh, n=3,4	harm	anh, n=3	anh, n=3,4
Br1 C1 C2	118.37	118.90(4)	118.89(4)	118.89(4)	118.90(4)	118.89(4)	118.89(4)
Br1 C1 C6	121.39	119.95(4)	119.95(4)	119.95(4)	119.94(3)	119.95(3)	119.95(3)
C1 C2 C3	121.37	120.48(5)	120.47(5)	120.48(5)	120.47(5)	120.47(5)	120.47(5)
C1 C2 H2	119.79	119.6(6)	119.5(6)	119.4(5)	117.9(7)	117.8(7)	117.7(7)
C2 C3 C4	119.47	119.49(6)	119.49(6)	119.49(5)	119.48(5)	119.48(5)	119.48(5)
C2 C3 H3	120.15	121.(1)	121.(1)	121.(1)	121.3(7)	121.3(7)	121.5(7)
C3 C2 H2	118.83	119.9(6)	120.0(6)	120.0(6)	121.6(7)	121.7(7)	121.8(7)
C3 C4 H4	120.57	120.8(9)	120.7(9)	121.0(9)	121.4(8)	121.3(8)	121.4(8)
C4 C3 H3	120.37	119.(1)	119.(1)	119.(1)	119.1(7)	119.0(7)	118.9(7)
C5 C4 C3	120.14	120.63(5)	120.62(5)	120.63(5)	120.64(5)	120.63(5)	120.63(5)
C5 C4 H4	119.29	118.5(9)	118.6(9)	118.4(9)	117.9(8)	118.1(8)	118.0(8)
C5 C6 C1	118.89	118.08(4)	118.07(4)	118.08(4)	118.07(4)	118.07(4)	118.07(4)
C5 C6 N1	118.53	119.49(4)	119.49(4)	119.48(4)	119.47(4)	119.47(4)	119.47(4)
C5 C7 C8	121.61	121.25(5)	121.25(5)	121.24(5)	121.21(4)	121.21(4)	121.21(4)
C5 C7 H7	118.71	118.9(10)	118.9(10)	118.9(9)	119.5(8)	119.6(8)	119.6(7)
C6 C1 C2	120.25	121.15(5)	121.15(5)	121.16(5)	121.16(4)	121.16(4)	121.16(4)
C6 C5 C4	119.88	120.17(5)	120.17(5)	120.16(4)	120.18(4)	120.18(4)	120.18(4)
C6 C5 C7	118.02	117.79(4)	117.78(4)	117.79(4)	117.83(4)	117.83(4)	117.83(4)
C6 N1 C9	124.93	124.14(5)	124.14(5)	124.15(4)	124.11(4)	124.11(4)	124.11(4)
C6 N1 H1	121.19	121.3(7)	121.4(7)	121.6(7)	122.4(8)	122.5(8)	122.4(8)
C7 C5 C4	122.09	122.05(5)	122.05(5)	122.05(5)	121.99(4)	121.99(4)	121.99(4)
C7 C8 H8	121.11	122.1(6)	122.1(6)	121.9(6)	119.6(7)	119.8(7)	119.6(7)
C8 C7 H7	119.68	119.9(10)	119.8(10)	119.8(9)	119.3(8)	119.2(8)	119.1(7)
C8 C9 O1	122.86	122.63(5)	122.63(5)	122.64(5)	122.61(4)	122.61(4)	122.61(4)
C9 C8 C7	120.94	121.16(5)	121.16(5)	121.17(5)	121.11(4)	121.11(4)	121.12(4)
C9 C8 H8	117.94	116.8(6)	116.7(6)	117.0(6)	119.3(7)	119.1(7)	119.3(7)
C9 N1 H1	113.86	114.5(7)	114.4(7)	114.2(7)	113.3(8)	113.3(8)	113.3(8)
N1 C6 C1	122.58	122.43(4)	122.43(4)	122.44(4)	122.46(4)	122.46(4)	122.46(4)
N1 C9 C8	115.96	116.17(4)	116.17(4)	116.17(4)	116.26(4)	116.26(4)	116.26(4)
N1 C9 O1	121.19	121.19(5)	121.20(5)	121.19(5)	121.13(4)	121.14(4)	121.13(4)

Table 15: Angles for PT-10(I) (intensity cut-off: $|F| \geq 2\sigma(|F|)$).

	opt	MM harm	MM anh, n=3	MM anh, n=3,4	HAR harm	HAR anh, n=3	HAR anh, n=3,4
C1 C2 C3	121.09	120.4(1)	120.4(1)	120.4(1)	120.40(9)	120.45(8)	120.44(8)
C1 C2 H2	119.03	115(1)	115(1)	115.9(10)	116.(1)	116.(1)	116.(1)
C1 C6 C5	119.42	119.51(9)	119.50(8)	119.50(8)	119.52(8)	119.49(7)	119.49(7)
C2 C3 H3	119.48	123(1)	122(1)	121(1)	119.5(8)	118.5(8)	118.7(8)
C3 C2 H2	119.87	125(1)	124.1(10)	123.6(10)	124.(1)	123.(1)	123.(1)
C3 C4 C5	120.09	120.1(1)	120.1(1)	120.1(1)	120.17(8)	120.16(8)	120.15(7)
C3 C4 H4	121.55	116.6(8)	116.1(7)	116.1(7)	116.(1)	113.9(9)	114.0(9)
C4 C3 C2	119.99	120.2(2)	120.2(1)	120.2(1)	120.30(8)	120.27(8)	120.27(8)
C4 C3 H3	120.53	117(1)	118(1)	119(1)	119.8(8)	120.8(8)	120.6(8)
C4 C5 C7	122.20	122.2(1)	122.2(1)	122.2(1)	122.31(8)	122.28(7)	122.27(7)
C5 C4 H4	118.35	122.9(8)	123.5(7)	123.5(7)	124.(1)	125.7(9)	125.6(9)
C5 C7 C8	121.51	121.4(1)	121.4(1)	121.4(1)	121.52(8)	121.53(7)	121.53(7)
C5 C7 H7	118.37	115.2(8)	114.9(7)	115.1(7)	113.(1)	113.1(9)	113.1(9)
C6 C1 C2	119.57	119.9(1)	119.94(9)	119.94(9)	119.91(8)	119.90(7)	119.92(7)
C6 C5 C4	119.85	119.8(1)	119.80(10)	119.80(10)	119.69(8)	119.72(8)	119.73(8)
C6 C5 C7	117.93	117.98(9)	117.98(8)	117.99(8)	118.00(8)	118.00(7)	118.00(7)
C6 N1 C9	125.85	125.6(1)	125.54(10)	125.54(10)	125.63(7)	125.59(7)	125.62(7)
C6 N1 H1	117.94	122.1(8)	122.4(8)	122.5(8)	119.(1)	119.(1)	119.(1)
C7 C8 H8	121.48	120.8(10)	123.8(9)	124.5(9)	124.(1)	125.(1)	126.(1)
C8 C7 H7	120.12	123.4(8)	123.7(7)	123.5(7)	125.(1)	125.3(9)	125.4(9)
C9 C8 C7	121.23	121.1(1)	121.0(1)	121.0(1)	120.94(8)	120.89(8)	120.90(8)
C9 C8 H8	117.27	118.1(10)	115.2(9)	114.5(8)	115.(1)	114.(1)	113.(1)
C9 N1 H1	116.20	112.0(8)	111.7(8)	111.6(7)	116.(1)	115.(1)	115.(1)
I1 C1 C2	118.98	119.15(8)	119.15(7)	119.15(7)	119.26(7)	119.29(6)	119.28(6)
I1 C1 C6	121.43	120.91(6)	120.90(5)	120.90(5)	120.82(6)	120.80(5)	120.79(5)
N1 C6 C1	122.27	122.0(1)	121.98(9)	121.99(9)	122.06(7)	122.04(6)	122.06(6)
N1 C6 C5	118.31	118.5(1)	118.51(9)	118.50(9)	118.42(7)	118.46(7)	118.45(7)
N1 C9 C8	115.13	115.48(9)	115.55(8)	115.56(8)	115.47(8)	115.51(7)	115.49(7)
N1 C9 O1	120.32	120.1(1)	120.1(1)	120.1(1)	120.17(8)	120.18(7)	120.19(7)
O1 C9 C8	124.55	124.5(1)	124.3(1)	124.3(1)	124.36(8)	124.31(8)	124.31(8)

Table 16: Angles for PT-10(I) (no intensity cut-off).

	opt	MM harm	MM anh, n=3	MM anh, n=3,4	HAR harm	HAR anh, n=3	HAR anh, n=3,4
C1 C2 C3	121.09	120.50(8)	120.50(8)	120.50(8)	120.40(8)	120.45(8)	120.44(8)
C1 C2 H2	119.03	116.1(9)	116.8(8)	116.9(8)	116.(1)	116.(1)	116.1(9)
C1 C6 C5	119.42	119.60(8)	119.59(7)	119.59(7)	119.53(7)	119.50(7)	119.50(7)
C2 C3 H3	119.48	123.(1)	122.(1)	121.(1)	119.5(8)	118.5(7)	118.7(8)
C3 C2 H2	119.87	123.4(8)	122.7(8)	122.6(8)	124.(1)	123.(1)	123.3(9)
C3 C4 C5	120.09	120.19(10)	120.18(9)	120.18(9)	120.18(8)	120.16(7)	120.15(7)
C3 C4 H4	121.55	118.1(7)	117.0(6)	116.9(6)	115.9(9)	114.0(9)	114.0(8)
C4 C3 C2	119.99	120.18(10)	120.17(9)	120.18(9)	120.30(8)	120.27(7)	120.27(7)
C4 C3 H3	120.53	117.(1)	118.(1)	119.(1)	119.7(8)	120.8(7)	120.6(7)
C4 C5 C7	122.20	122.32(10)	122.30(9)	122.30(9)	122.31(8)	122.28(7)	122.27(7)
C5 C4 H4	118.35	121.4(7)	122.5(6)	122.7(6)	123.7(9)	125.6(8)	125.6(8)
C5 C7 C8	121.51	121.50(10)	121.52(9)	121.51(9)	121.53(8)	121.54(7)	121.53(7)
C5 C7 H7	118.37	117.6(8)	117.1(7)	117.2(7)	113.(1)	113.2(9)	113.2(9)
C6 C1 C2	119.57	119.81(8)	119.83(8)	119.83(8)	119.90(7)	119.89(7)	119.91(7)
C6 C5 C4	119.85	119.72(8)	119.72(7)	119.72(7)	119.69(8)	119.72(7)	119.73(7)
C6 C5 C7	117.93	117.96(8)	117.97(7)	117.98(7)	118.00(8)	118.00(7)	118.00(7)
C6 N1 C9	125.85	125.53(8)	125.53(8)	125.53(8)	125.63(7)	125.59(6)	125.63(6)
C6 N1 H1	117.94	122.1(7)	121.8(7)	121.8(7)	118.(1)	119.(1)	119.(1)
C7 C8 H8	121.48	119.6(9)	122.1(7)	122.2(7)	124.(1)	125.(1)	126.(1)
C8 C7 H7	120.12	120.8(8)	121.3(7)	121.2(7)	125.(1)	125.2(9)	125.3(9)
C9 C8 C7	121.23	121.04(9)	120.94(9)	120.94(8)	120.93(8)	120.88(7)	120.88(7)
C9 C8 H8	117.27	119.3(9)	116.9(8)	116.8(8)	115.(1)	114.(1)	113.(1)
C9 N1 H1	116.20	112.1(7)	112.4(7)	112.4(7)	116.(1)	115.(1)	115.(1)
I1 C1 C2	118.98	119.22(6)	119.22(6)	119.22(6)	119.27(6)	119.29(6)	119.28(6)
I1 C1 C6	121.43	120.96(6)	120.94(5)	120.94(5)	120.83(5)	120.81(5)	120.80(5)
N1 C6 C1	122.27	121.90(8)	121.91(7)	121.91(7)	122.05(7)	122.03(6)	122.05(6)
N1 C6 C5	118.31	118.49(8)	118.50(7)	118.49(7)	118.42(7)	118.46(7)	118.45(6)
N1 C9 C8	115.13	115.46(8)	115.53(7)	115.53(7)	115.47(8)	115.51(7)	115.49(7)
N1 C9 O1	120.32	120.08(10)	120.13(9)	120.13(9)	120.18(8)	120.18(7)	120.20(7)
O1 C9 C8	124.55	124.45(11)	124.34(10)	124.34(10)	124.35(8)	124.30(7)	124.31(7)

Table 17: Angles for PT-11(S-Ph) (intensity cut-off: $|F| \geq 2\sigma(|F|)$).

			MM			MM(freeXH)			HAR	
	opt	harm	anh, n=3	anh, n=3,4	harm	anh, n=3	anh, n=3,4	harm	anh, n=3	anh, n=3,4
C1 C2 C3	121.24	121.14(1)	121.14(1)	121.14(1)	121.14(1)	121.14(1)	121.14(1)	121.16(2)	121.16(2)	121.16(2)
C1 C2 H2	117.24	117.4(6)	117.3(5)	117.3(5)	117.5(8)	117.4(8)	117.4(8)	118.6(4)	118.5(4)	118.4(4)
C1 N1 C9	124.76	124.42(2)	124.42(2)	124.42(2)	124.43(1)	124.42(2)	124.42(2)	124.42(1)	124.42(1)	124.42(1)
C1 N1 H1	114.71	114.9(4)	115.1(4)	115.0(4)	114.8(7)	114.9(8)	114.9(8)	114.7(4)	114.8(4)	114.7(4)
C10 C11 C12	119.37	119.36(2)	119.36(2)	119.36(2)	119.36(2)	119.36(2)	119.36(2)	119.33(2)	119.34(2)	119.34(2)
C10 C11 H11	121.46	121.4(5)	121.6(5)	121.7(5)	120.9(7)	121.1(7)	121.2(7)	120.9(4)	120.8(4)	120.8(4)
C10 C15 C14	120.12	119.99(2)	120.00(2)	120.00(2)	119.99(2)	120.00(2)	120.00(2)	119.97(2)	119.98(2)	119.98(2)
C10 C15 H15	119.99	120.5(4)	120.6(4)	120.6(4)	120.4(8)	120.6(8)	120.5(8)	120.2(4)	120.3(4)	120.3(4)
C11 C10 C15	119.84	120.09(1)	120.08(1)	120.08(1)	120.09(1)	120.09(1)	120.08(1)	120.11(2)	120.10(2)	120.10(2)
C11 C10 S1	116.36	116.14(1)	116.18(1)	116.18(1)	116.143(10)	116.18(1)	116.18(1)	116.13(1)	116.17(1)	116.17(1)
C11 C12 C13	121.23	121.02(2)	121.02(2)	121.02(2)	121.02(2)	121.02(2)	121.02(2)	121.04(2)	121.04(2)	121.04(2)
C11 C12 H12	118.20	118.0(6)	118.0(6)	118.1(6)	117.8(7)	118.0(8)	118.0(8)	118.3(4)	118.4(4)	118.4(4)
C12 C11 H11	119.16	119.2(5)	119.0(5)	119.0(5)	119.7(7)	119.5(8)	119.5(8)	119.8(4)	119.8(4)	119.8(4)
C12 C13 C14	118.96	119.12(2)	119.13(2)	119.13(2)	119.13(2)	119.13(2)	119.13(2)	119.11(2)	119.12(2)	119.12(2)
C12 C13 H13	120.42	120.3(5)	120.3(5)	120.4(5)	120.1(9)	120.2(9)	120.2(9)	120.6(4)	120.7(4)	120.7(4)
C13 C12 H12	120.57	121.0(6)	120.9(6)	120.9(6)	121.2(8)	121.0(9)	121.0(9)	120.7(4)	120.6(4)	120.6(4)
C13 C14 C15	120.46	120.41(2)	120.41(2)	120.40(2)	120.41(2)	120.40(2)	120.40(2)	120.43(2)	120.42(2)	120.42(2)
C13 C14 H14	120.08	121.0(5)	121.0(5)	121.0(5)	121.0(8)	121.0(9)	121.0(9)	120.9(4)	120.9(4)	121.0(4)
C14 C13 H13	120.61	120.5(5)	120.5(5)	120.5(5)	120.8(9)	120.6(9)	120.6(9)	120.2(4)	120.1(4)	120.2(4)
C14 C15 H15	119.89	119.5(4)	119.4(4)	119.4(4)	119.6(7)	119.4(8)	119.5(8)	119.8(4)	119.7(4)	119.7(4)
C15 C10 S1	123.77	123.74(1)	123.72(1)	123.72(1)	123.75(1)	123.72(1)	123.72(1)	123.74(1)	123.71(1)	123.71(1)
C15 C14 H14	119.41	118.6(5)	118.6(5)	118.5(5)	118.6(8)	118.6(8)	118.6(8)	118.7(4)	118.6(4)	118.6(4)
C2 C1 N1	115.79	116.04(2)	116.04(2)	116.04(2)	116.04(2)	116.04(2)	116.04(2)	116.02(1)	116.02(1)	116.02(1)
C2 C1 O1	123.57	123.14(2)	123.14(2)	123.14(2)	123.14(2)	123.14(2)	123.14(2)	123.16(2)	123.16(2)	123.16(2)
C2 C3 C4	121.29	121.07(2)	121.07(2)	121.07(2)	121.07(2)	121.07(2)	121.07(2)	121.08(2)	121.07(1)	121.07(1)
C2 C3 H3	120.48	121.6(5)	121.7(5)	121.7(5)	122.0(8)	121.9(8)	121.9(8)	121.5(4)	121.5(4)	121.5(4)
C3 C2 H2	121.52	121.4(6)	121.6(5)	121.6(6)	121.4(9)	121.5(9)	121.5(9)	120.2(4)	120.4(4)	120.4(4)
C3 C4 C5	122.15	122.14(2)	122.14(2)	122.14(2)	122.14(2)	122.14(2)	122.14(2)	122.17(2)	122.16(2)	122.17(1)
C3 C4 C9	117.95	118.03(2)	118.04(2)	118.04(2)	118.03(2)	118.04(2)	118.04(2)	118.02(1)	118.03(1)	118.03(1)
C4 C3 H3	118.24	117.4(5)	117.2(5)	117.3(5)	117.0(6)	117.0(7)	117.0(7)	117.4(4)	117.5(4)	117.5(4)
C4 C5 C6	120.69	120.67(2)	120.67(2)	120.67(2)	120.67(2)	120.67(2)	120.67(2)	120.68(2)	120.69(2)	120.68(2)
C4 C5 H5	118.50	117.9(4)	117.9(4)	117.9(4)	118.0(7)	117.9(8)	117.9(8)	118.5(4)	118.6(4)	118.5(4)
C4 C9 C8	118.92	119.31(2)	119.31(2)	119.31(2)	119.31(2)	119.31(2)	119.31(2)	119.30(1)	119.30(1)	119.30(1)
C4 C9 N1	118.89	119.23(2)	119.23(2)	119.23(2)	119.23(2)	119.23(2)	119.23(2)	119.23(1)	119.22(1)	119.22(1)
C5 C4 C9	119.90	119.83(2)	119.82(2)	119.82(2)	119.83(2)	119.82(2)	119.82(2)	119.81(2)	119.80(2)	119.80(2)
C5 C6 C7	119.36	119.31(2)	119.31(2)	119.31(2)	119.31(2)	119.31(2)	119.31(2)	119.34(2)	119.34(2)	119.34(2)
C5 C6 H6	120.78	121.8(6)	121.7(6)	121.8(6)	122.0(9)	121.8(10)	121.8(10)	121.3(4)	121.4(4)	121.5(4)
C6 C5 H5	120.81	121.4(4)	121.4(4)	121.5(4)	121.3(8)	121.4(9)	121.5(9)	120.8(4)	120.7(4)	120.8(4)
C6 C7 C8	121.59	121.46(2)	121.46(2)	121.46(2)	121.45(2)	121.46(2)	121.46(2)	121.42(2)	121.42(2)	121.42(2)
C6 C7 H7	120.35	120.0(5)	119.9(4)	119.9(4)	120.0(8)	120.0(8)	120.1(8)	120.0(4)	120.0(4)	120.0(4)
C7 C6 H6	119.86	118.9(6)	119.0(6)	118.9(6)	118.7(9)	118.9(10)	118.9(10)	119.4(4)	119.2(4)	119.2(4)
C7 C8 C9	119.50	119.39(1)	119.39(1)	119.39(1)	119.39(1)	119.39(1)	119.39(1)	119.41(1)	119.41(1)	119.41(1)
C7 C8 S1	118.32	118.87(1)	118.88(1)	118.88(1)	118.87(1)	118.88(1)	118.88(1)	118.86(1)	118.87(1)	118.87(1)
C8 C7 H7	118.06	118.6(5)	118.6(4)	118.6(4)	118.5(7)	118.5(7)	118.5(7)	118.6(4)	118.5(4)	118.6(4)
C8 C9 N1	122.18	121.45(1)	121.45(1)	121.45(1)	121.45(1)	121.45(1)	121.45(1)	121.47(1)	121.47(1)	121.47(1)
C8 S1 C10	103.87	103.379(7)	103.457(9)	103.457(9)	103.379(7)	103.457(9)	103.457(9)	103.387(8)	103.461(9)	103.460(9)
C9 C8 S1	121.99	121.56(1)	121.55(1)	121.55(1)	121.56(1)	121.55(1)	121.55(1)	121.55(1)	121.54(1)	121.54(1)
C9 N1 H1	120.52	120.6(4)	120.5(4)	120.5(4)	120.8(9)	120.6(9)	120.6(9)	120.8(4)	120.8(4)	120.9(4)
N1 C1 O1	120.64	120.82(2)	120.82(2)	120.82(2)	120.82(2)	120.82(2)	120.82(2)	120.82(1)	120.82(1)	120.81(1)

Table 18: Angles for PT-11(S-Ph) (no intensity cut-off).

		MM			MM(freeXH)			HAR		
	opt	harm	anh, n=3	anh, n=3,4	harm	anh, n=3	anh, n=3,4	harm	anh, n=3	anh, n=3,4
C1 C2 C3	121.24	121.148(15)	121.149(14)	121.149(14)	121.147(15)	121.149(14)	121.148(14)	121.16(2)	121.16(2)	121.16(2)
C1 C2 H2	117.24	117.4(5)	117.3(5)	117.3(5)	117.4(8)	117.3(8)	117.3(8)	118.6(4)	118.5(4)	118.4(4)
C1 N1 C9	124.76	124.423(13)	124.421(13)	124.421(13)	124.423(13)	124.422(13)	124.422(13)	124.42(1)	124.42(1)	124.42(1)
C1 N1 H1	114.71	115.0(4)	115.1(4)	115.1(4)	114.9(8)	115.0(8)	115.0(8)	114.7(4)	114.8(4)	114.7(4)
C10 C11 C12	119.37	119.347(16)	119.350(15)	119.351(15)	119.347(16)	119.351(15)	119.351(15)	119.33(2)	119.34(2)	119.34(2)
C10 C11 H11	121.46	120.7(5)	120.9(5)	120.9(5)	120.5(7)	120.7(7)	120.7(7)	120.8(4)	120.8(4)	120.8(4)
C10 C15 C14	120.12	119.990(17)	119.993(16)	119.993(16)	119.991(17)	119.994(16)	119.994(16)	119.97(2)	119.98(2)	119.98(2)
C10 C15 H15	119.99	120.3(4)	120.4(4)	120.4(4)	120.3(7)	120.4(7)	120.4(7)	120.2(3)	120.3(3)	120.3(3)
C11 C10 C15	119.84	120.093(15)	120.089(14)	120.089(14)	120.093(15)	120.089(14)	120.089(14)	120.11(2)	120.10(2)	120.10(2)
C11 C10 S1	116.36	116.137(13)	116.167(13)	116.168(13)	116.136(12)	116.166(13)	116.167(13)	116.13(1)	116.17(1)	116.17(1)
C11 C12 C13	121.23	121.006(19)	121.007(19)	121.007(19)	121.006(19)	121.007(19)	121.007(19)	121.04(2)	121.03(2)	121.03(2)
C11 C12 H12	118.20	118.2(5)	118.3(5)	118.3(5)	118.1(8)	118.3(7)	118.3(7)	118.3(4)	118.3(4)	118.4(4)
C12 C11 H11	119.16	120.0(5)	119.8(5)	119.7(5)	120.1(7)	119.9(7)	119.9(7)	119.8(4)	119.8(4)	119.8(4)
C12 C13 C14	118.96	119.14(2)	119.14(2)	119.14(2)	119.14(2)	119.14(2)	119.14(2)	119.11(2)	119.12(2)	119.12(2)
C12 C13 H13	120.42	120.0(5)	120.1(5)	120.1(5)	119.9(9)	120.0(9)	120.1(9)	120.6(4)	120.7(4)	120.7(4)
C13 C12 H12	120.57	120.8(5)	120.7(5)	120.7(5)	120.9(9)	120.7(8)	120.7(8)	120.7(4)	120.6(4)	120.6(4)
C13 C14 C15	120.46	120.413(19)	120.410(19)	120.409(19)	120.411(19)	120.408(19)	120.408(19)	120.43(2)	120.42(2)	120.42(2)
C13 C14 H14	120.08	120.9(5)	120.9(5)	121.0(5)	120.9(9)	120.9(9)	121.0(9)	120.9(4)	120.9(4)	121.0(4)
C14 C13 H13	120.61	120.9(5)	120.7(4)	120.7(4)	121.0(9)	120.8(9)	120.8(9)	120.2(4)	120.1(4)	120.2(4)
C14 C15 H15	119.89	119.7(4)	119.6(4)	119.6(4)	119.7(8)	119.6(8)	119.6(8)	119.8(3)	119.7(3)	119.7(3)
C15 C10 S1	123.77	123.750(12)	123.724(12)	123.723(12)	123.750(12)	123.724(12)	123.724(12)	123.74(1)	123.71(1)	123.71(1)
C15 C14 H14	119.41	118.7(5)	118.7(5)	118.6(5)	118.6(8)	118.6(8)	118.6(8)	118.7(4)	118.6(4)	118.6(4)
C2 C1 N1	115.79	116.035(13)	116.035(13)	116.035(13)	116.035(13)	116.035(13)	116.035(13)	116.02(1)	116.02(1)	116.02(1)
C2 C1 O1	123.57	123.139(16)	123.139(16)	123.140(16)	123.139(16)	123.139(16)	123.140(16)	123.15(2)	123.16(1)	123.16(1)
C2 C3 C4	121.29	121.060(16)	121.059(15)	121.059(15)	121.060(16)	121.059(15)	121.059(15)	121.08(1)	121.07(1)	121.07(1)
C2 C3 H3	120.48	122.0(4)	121.9(4)	121.9(4)	122.1(8)	122.0(8)	122.0(8)	121.6(4)	121.5(4)	121.5(4)
C3 C2 H2	121.52	121.4(5)	121.6(5)	121.5(5)	121.4(9)	121.5(9)	121.5(9)	120.2(4)	120.4(4)	120.4(4)
C3 C4 C5	122.15	122.140(16)	122.142(16)	122.142(16)	122.139(16)	122.142(16)	122.142(16)	122.17(1)	122.16(1)	122.16(1)
C3 C4 C9	117.95	118.024(13)	118.027(13)	118.027(13)	118.024(13)	118.027(13)	118.027(13)	118.02(1)	118.03(1)	118.03(1)
C4 C3 H3	118.24	116.9(5)	117.0(4)	117.0(4)	116.9(7)	116.9(7)	116.9(7)	117.3(4)	117.4(4)	117.4(4)
C4 C5 C6	120.69	120.666(16)	120.669(16)	120.669(16)	120.666(16)	120.669(16)	120.669(16)	120.68(2)	120.69(2)	120.68(1)
C4 C5 H5	118.50	118.5(4)	118.4(4)	118.4(4)	118.5(8)	118.4(8)	118.4(8)	118.4(4)	118.6(4)	118.5(4)
C4 C9 C8	118.92	119.296(13)	119.299(13)	119.298(13)	119.296(13)	119.299(13)	119.298(13)	119.30(1)	119.30(1)	119.30(1)
C4 C9 N1	118.89	119.246(13)	119.243(12)	119.244(12)	119.246(13)	119.244(13)	119.244(12)	119.23(1)	119.22(1)	119.22(1)
C5 C4 C9	119.90	119.836(14)	119.830(13)	119.831(13)	119.837(14)	119.831(13)	119.831(13)	119.81(2)	119.80(2)	119.80(1)
C5 C6 C7	119.36	119.325(17)	119.325(17)	119.325(17)	119.324(17)	119.324(17)	119.324(17)	119.34(2)	119.34(2)	119.34(2)
C5 C6 H6	120.78	121.4(5)	121.2(5)	121.2(5)	121.4(9)	121.2(9)	121.2(9)	121.3(4)	121.4(4)	121.5(4)
C6 C5 H5	120.81	120.9(4)	120.9(4)	121.0(4)	120.9(9)	120.9(9)	121.0(9)	120.9(4)	120.8(4)	120.9(4)
C6 C7 C8	121.59	121.454(15)	121.454(15)	121.454(15)	121.455(15)	121.455(15)	121.455(15)	121.42(2)	121.42(2)	121.42(2)
C6 C7 H7	120.35	119.6(5)	119.7(5)	119.7(5)	119.7(8)	119.8(8)	119.8(8)	119.9(4)	120.0(4)	120.0(4)
C7 C6 H6	119.86	119.3(5)	119.4(5)	119.4(5)	119.3(9)	119.4(9)	119.4(9)	119.3(4)	119.2(4)	119.2(4)
C7 C8 C9	119.50	119.384(14)	119.383(14)	119.385(14)	119.383(14)	119.383(14)	119.384(14)	119.41(1)	119.41(1)	119.41(1)
C7 C8 S1	118.32	118.870(11)	118.880(12)	118.879(12)	118.870(11)	118.880(12)	118.879(12)	118.86(1)	118.87(1)	118.87(1)
C8 C7 H7	118.06	118.9(5)	118.9(5)	118.9(5)	118.9(8)	118.8(7)	118.8(7)	118.6(4)	118.6(4)	118.6(4)
C8 C9 N1	122.18	121.454(13)	121.453(13)	121.454(13)	121.454(13)	121.453(13)	121.453(13)	121.46(1)	121.47(1)	121.47(1)
C8 S1 C10	103.87	103.382(8)	103.455(9)	103.455(9)	103.382(8)	103.455(9)	103.455(9)	103.387(7)	103.461(9)	103.460(9)
C9 C8 S1	121.99	121.560(10)	121.555(11)	121.554(11)	121.561(10)	121.556(11)	121.555(11)	121.55(1)	121.54(1)	121.54(1)
C9 N1 H1	120.52	120.6(4)	120.5(3)	120.5(3)	120.7(8)	120.6(8)	120.6(8)	120.8(4)	120.8(4)	120.9(4)
N1 C1 O1	120.64	120.821(15)	120.822(15)	120.820(15)	120.821(15)	120.822(15)	120.820(15)	120.82(1)	120.81(1)	120.81(1)

Table 19: Anharmonic Gram-Charlier coefficients obtained in 3rd-order-only anharmonic thermal motion refinement (intensity cut-off: $|F| \geq 2\sigma(|F|)$). Values higher than three standard uncertainties highlighted with cyan.

	PT-11(S-Ph)			PT-2(Cl)			PT-8(Br)		PT-10(I)	
	HAR	MM	MM(freeXH)	HAR	MM	MM(freeXH)	HAR	MM	HAR	MM
U_{111}	0.00(3)	-0.00002(2)	-0.00002(2)	0.000018(9)	0.00004(1)	0.00004(1)	-0.000002(7)	0.000008(8)	-0.0010(1)	-0.0009(1)
U_{112}	0.0(2)	-0.00005(1)	-0.00005(1)	0.00005(1)	0.00013(4)	0.00013(4)	0.00004(1)	0.00011(4)	0.00013(2)	0.00050(5)
U_{113}	0.0(2)	0.00025(3)	0.00025(3)	0.000017(5)	0.00004(1)	0.00004(1)	0.000007(3)	0.000003(9)	-0.00005(3)	0.00013(8)
U_{122}	0.0(2)	-0.000016(4)	-0.000016(4)	0.00004(3)	-0.0000(1)	-0.0000(1)	-0.00003(3)	-0.00020(9)	-0.000029(4)	-0.00009(1)
U_{123}	0.00(3)	0.00005(1)	0.00005(1)	0.000020(8)	-0.00004(4)	-0.00004(4)	0.000013(6)	-0.00003(3)	0.000027(4)	0.00012(2)
U_{133}	0.0(2)	0.00007(2)	0.00007(2)	0.000004(4)	-0.000022(9)	-0.000022(9)	-0.000004(3)	-0.000020(6)	-0.00001(1)	0.00000(3)
U_{222}	0.00(1)	0.000005(1)	0.000005(1)	0.0002(2)	0.0001(2)	0.0001(2)	-0.0001(2)	-0.0000(2)	0.000043(1)	0.000047(1)
U_{223}	0.0(2)	0.000032(3)	0.000032(3)	-0.00014(3)	-0.00029(7)	-0.00029(7)	-0.00015(2)	-0.00041(6)	0.000000(1)	0.000009(4)
U_{233}	0.0(2)	0.000001(7)	0.000001(7)	-0.000013(8)	-0.00007(2)	-0.00007(2)	-0.000016(6)	-0.00007(2)	0.000014(2)	0.000043(7)
U_{333}	0.00(2)	0.00009(1)	0.00009(1)	-0.000020(4)	-0.000010(3)	-0.000010(3)	-0.000010(3)	-0.000003(2)	0.000059(8)	0.000067(7)

Table 20: Anharmonic Gram-Charlier coefficients obtained in 3rd-order-only anharmonic thermal motion refinement (no intensity cut-off). Values higher than three standard uncertainties highlighted with cyan.

	PT-11(S-Ph)			PT-2(Cl)			PT-8(Br)		PT-10(I)	
	HAR	MM	MM(freeXH)	HAR	MM	MM(freeXH)	HAR	MM	HAR	MM
U_{111}	0.00(3)	-0.00002(1)	-0.00002(2)	0.000017(8)	0.00003(6)	0.00003(1)	-0.000003(6)	0.00001(8)	-0.0010(1)	-0.0009(1)
U_{112}	0.0(2)	-0.00004(9)	-0.00004(1)	0.00005(1)	0.0001(3)	0.0001(4)	0.00004(1)	0.0001(4)	0.00013(2)	0.0005(5)
U_{113}	0.0(2)	-0.00002(3)	-0.00002(4)	0.000017(4)	0.00005(8)	0.00006(9)	0.000007(3)	-0.0002(9)	-0.00006(3)	-0.00009(1)
U_{122}	0.0(2)	0.0002(1)	0.0002(3)	0.00004(3)	0.00004(6)	0.00004(1)	-0.00003(3)	0.000005(9)	-0.000029(3)	0.0002(8)
U_{123}	0.00(3)	0.00005(1)	0.00005(1)	0.000019(7)	-0.00004(3)	-0.00004(4)	0.000012(5)	-0.00004(3)	0.000027(4)	0.0001(2)
U_{133}	0.0(2)	0.000029(3)	0.00003(3)	0.000004(3)	-0.0003(5)	-0.0003(6)	-0.000004(2)	-0.0004(6)	-0.00001(1)	0.000008(4)
U_{222}	0.00(1)	0.000005(1)	0.000005(1)	0.0002(1)	0.0001(9)	0.0001(2)	-0.0001(1)	-0.0001(2)	0.000044(1)	0.00005(1)
U_{223}	0.0(1)	0.00004(2)	0.00004(2)	-0.00013(2)	-0.000021(9)	-0.00002(8)	-0.00016(2)	-0.00002(6)	0.000000(1)	0.00001(3)
U_{233}	0.0(1)	0.000004(6)	0.000004(7)	-0.000013(7)	-0.0001(1)	-0.00006(2)	-0.000015(5)	-0.00007(2)	0.000015(2)	0.00004(6)
U_{333}	0.00(2)	0.00007(6)	0.00007(1)	-0.000019(4)	-0.00001(2)	-0.000009(3)	-0.000011(3)	0.000003(2)	0.000059(8)	0.00007(7)

Table 21: Anharmonic Gram-Charlier coefficients obtained in 3rd and 4th order anharmonic thermal motion refinement (intensity cut-off: $|F| \geq 2\sigma(|F|)$). Values higher than three standard uncertainties highlighted with cyan.

	PT-11(S-Ph)			PT-2(Cl)			PT-8(Br)		PT-10(I)	
	HAR	MM	MM(freeXH)	HAR	MM	MM(freeXH)	HAR	MM	HAR	MM
U_{111}	0.00(3)	-0.00002(2)	-0.00002(2)	0.000019(8)	0.00004(1)	0.00004(1)	0.000000(7)	0.000010(8)	-0.0009(1)	-0.0009(2)
U_{112}	0.0(2)	-0.00005(1)	0.000006(1)	0.00005(1)	0.00013(4)	0.0002(2)	0.00004(1)	0.00011(4)	0.00013(2)	0.00051(5)
U_{113}	0.0(2)	0.00025(3)	0.00009(1)	0.000016(4)	0.00003(1)	-0.000010(3)	0.000007(3)	0.000002(9)	-0.00005(3)	0.00013(8)
U_{122}	0.0(2)	-0.000016(4)	-0.00005(1)	0.00003(3)	-0.0000(1)	0.00013(4)	-0.00002(3)	-0.00019(9)	-0.000028(3)	-0.00010(1)
U_{123}	0.00(3)	0.00005(1)	-0.000016(4)	0.000019(7)	-0.00004(4)	-0.0000(1)	0.000013(6)	-0.00003(3)	0.000026(4)	0.00012(2)
U_{133}	0.0(2)	0.00007(2)	0.00025(3)	0.000005(3)	-0.000021(8)	0.00003(1)	-0.000004(2)	-0.000020(6)	-0.00001(1)	0.00000(3)
U_{222}	0.00(1)	0.000005(1)	0.00007(2)	0.0002(1)	0.0002(2)	-0.000021(8)	-0.0001(2)	0.0000(2)	0.000042(1)	0.000048(1)
U_{223}	0.0(2)	0.000033(3)	0.000033(3)	-0.00013(2)	-0.00029(7)	-0.00029(7)	-0.00015(2)	-0.00041(6)	-0.000001(1)	0.000010(4)
U_{233}	0.0(1)	0.000001(7)	0.000001(7)	-0.000013(7)	-0.00007(2)	-0.00007(2)	-0.000015(6)	-0.00007(2)	0.000014(2)	0.000046(7)
U_{333}	0.00(2)	0.00009(1)	0.00005(1)	-0.000017(4)	-0.000010(3)	-0.00004(4)	-0.000010(3)	-0.000003(2)	0.000058(8)	0.000071(8)
U_{1111}	0.0(1)	0.00002(2)	0.00002(2)	-0.000061(4)	-0.000046(6)	-0.000046(6)	-0.000024(3)	-0.000037(3)	-0.0023(2)	0.0005(2)
U_{1112}	0.(2)	-0.000001(9)	0.000001(0)	-0.000001(5)	0.00002(3)	-0.0008(2)	-0.000014(5)	-0.00007(2)	-0.00004(2)	-0.00012(9)
U_{1113}	0.(2)	-0.00002(3)	-0.000007(9)	-0.000027(2)	-0.000067(7)	-0.000009(1)	-0.000006(1)	-0.000029(5)	-0.00014(4)	0.0003(1)
U_{1122}	0.00(8)	0.000000(4)	-0.000001(9)	-0.000070(9)	0.00012(7)	0.00002(3)	-0.000046(8)	-0.00022(6)	-0.000022(3)	0.00016(2)
U_{1123}	0.(1)	-0.00002(1)	-0.00002(3)	-0.000002(3)	-0.00000(3)	-0.000067(7)	-0.000001(3)	0.00002(3)	0.000001(5)	0.00004(6)
U_{1133}	0.0(2)	-0.00003(3)	0.000000(4)	-0.000022(1)	-0.000056(6)	0.00012(7)	-0.0000063(8)	-0.000043(4)	-0.00010(1)	0.00043(7)
U_{1222}	0.(1)	-0.000004(1)	-0.00002(1)	0.00006(3)	0.0005(2)	-0.00001(3)	0.00007(4)	0.0005(2)	-0.000002(1)	-0.000009(4)
U_{1223}	0.(2)	-0.000014(5)	-0.00003(3)	-0.000002(5)	0.00022(6)	-0.000056(6)	0.000027(5)	0.00037(5)	-0.0000039(9)	-0.00002(1)
U_{1233}	0.(1)	-0.00000(1)	-0.000005(1)	-0.000002(2)	-0.00000(1)	0.0005(2)	-0.000001(2)	-0.00001(2)	-0.000003(2)	-0.00002(2)
U_{1333}	0.(2)	-0.00005(2)	-0.000014(5)	-0.000017(1)	-0.000025(3)	0.00022(6)	-0.0000061(7)	-0.000010(2)	-0.000019(6)	0.00006(2)
U_{2222}	0.0(2)	0.000001(0)	-0.00001(1)	-0.0021(2)	-0.0008(2)	-0.00000(2)	0.0010(2)	0.0005(2)	-0.0000045(5)	0.000003(1)
U_{2223}	0.(2)	-0.000002(1)	-0.00005(2)	0.00002(3)	0.0001(1)	-0.000025(3)	-0.00002(3)	-0.0002(1)	-0.0000009(4)	-0.000003(2)
U_{2233}	0.(2)	-0.000004(3)	-0.000002(1)	-0.000035(5)	-0.00004(3)	0.0001(1)	0.000009(5)	-0.00011(2)	-0.0000054(5)	0.000020(3)
U_{2333}	0.0(2)	-0.000002(6)	-0.000004(3)	-0.000004(2)	-0.000006(8)	-0.00004(3)	0.000000(2)	0.000006(7)	-0.000002(1)	-0.000004(5)
U_{3333}	0.(2)	-0.000008(9)	-0.000002(6)	-0.000027(1)	-0.000009(1)	-0.000006(8)	-0.000006(1)	-0.000002(1)	-0.000035(5)	0.000048(5)

Table 22: Anharmonic Gram-Charlier coefficients obtained in 3rd and 4th order anharmonic thermal motion refinement (no intensity cut-off). Values higher than three standard uncertainties highlighted with cyan.

	PT-11(S-Ph)			PT-2(Cl)			PT-8(Br)		PT-10(I)	
	HAR	MM	MM(freeXH)	HAR	MM	MM(freeXH)	HAR	MM	HAR	MM
U_{111}	0.00(3)	-0.00002(2)	-0.00002(1)	0.000019(7)	0.00003(6)	0.00003(9)	-0.000001(6)	0.00001(7)	-0.0010(1)	-0.0009(1)
U_{112}	0.0(2)	-0.00004(1)	-0.00004(1)	0.00005(1)	0.00011(3)	0.0001(4)	0.000038(9)	0.0001(4)	0.00013(1)	0.00051(5)
U_{113}	0.0(2)	-0.00002(4)	-0.00002(4)	0.000016(4)	0.000052(8)	0.00006(9)	0.000007(3)	-0.0002(9)	-0.00005(3)	-0.00009(1)
U_{122}	0.0(2)	0.0002(3)	0.0002(3)	0.00004(3)	0.000035(9)	0.000035(1)	-0.00003(2)	0.000004(9)	-0.000028(3)	0.0002(8)
U_{123}	0.0(2)	0.00003(3)	0.00003(3)	0.000005(3)	-0.00025(5)	-0.00025(6)	-0.000004(2)	-0.0004(6)	-0.00001(1)	0.000008(4)
U_{133}	0.00(3)	0.00005(1)	0.00005(1)	0.000019(6)	-0.000041(3)	-0.00004(3)	0.000013(5)	-0.00003(3)	0.000026(4)	0.00013(7)
U_{222}	0.00(1)	0.000005(1)	0.000005(1)	0.0002(1)	0.00014(9)	0.00015(1)	0.0000(1)	-0.0001(2)	0.000042(1)	0.00005(1)
U_{223}	0.0(1)	0.00004(2)	0.00004(2)	-0.00012(2)	-0.00002(6)	-0.00002(7)	-0.00016(2)	-0.00002(6)	-0.000001(1)	0.000009(3)
U_{233}	0.0(1)	0.000004(7)	0.000004(7)	-0.000013(6)	-0.000061(4)	-0.00006(2)	-0.000014(5)	-0.00006(2)	0.000014(2)	0.00004(4)
U_{333}	0.00(2)	0.00007(1)	0.00007(1)	-0.000016(3)	-0.0000088(2)	-0.000009(3)	-0.000010(3)	-0.000003(2)	0.000058(7)	0.00007(7)
U_{1111}	0.00(9)	0.00001(2)	0.00002(2)	-0.000060(3)	-0.00004(2)	-0.00004(5)	-0.000025(3)	-0.00005(3)	-0.0023(2)	0.0001(2)
U_{1112}	0.(2)	0.000004(1)	0.000004(2)	0.000000(4)	0.000025(2)	0.000025(2)	-0.000017(4)	-0.0001(2)	-0.00005(2)	-0.00013(5)
U_{1113}	0.(2)	-0.00003(3)	-0.00003(5)	-0.000026(2)	-0.00007(4)	-0.00007(7)	-0.000006(1)	-0.00004(4)	-0.00014(4)	0.0003(9)
U_{1122}	0.00(8)	-0.0000142	-0.00001(1)	-0.000068(8)	0.0002(5)	0.00019(6)	-0.000052(7)	0.0003(5)	-0.000022(3)	-0.000020(4)
U_{1123}	0.(1)	-0.000002(1)	-0.000002(1)	-0.000002(2)	0.000086(7)	0.00009(9)	-0.000003(2)	-0.0002(9)	0.000000(5)	-0.000003(2)
U_{1133}	0.0(2)	-0.000002(1)	-0.000002(2)	-0.000022(1)	-0.000002(2)	-0.000001(2)	-0.0000064(7)	-0.00002(2)	-0.00010(1)	-0.00002(1)
U_{1222}	0.(1)	-0.000001(5)	-0.000001(2)	0.00006(3)	0.00013(5)	0.00013(6)	0.00004(4)	-0.0005(5)	-0.000002(1)	0.0001(1)
U_{1223}	0.(2)	-0.000004(4)	-0.000004(4)	-0.000003(5)	-0.000031(2)	-0.00003(3)	0.000024(4)	-0.0002(2)	-0.0000038(9)	0.000010(3)
U_{1233}	0.(1)	-0.000004(6)	-0.000004(6)	-0.000002(2)	-0.000005(5)	-0.000005(7)	-0.000003(2)	0.000001(6)	-0.000002(2)	-0.000005(5)
U_{1333}	0.(1)	-0.00003(4)	-0.00003(1)	-0.0000169(9)	-0.000057(4)	-0.00006(6)	-0.0000061(7)	-0.00005(4)	-0.000019(6)	0.0003(5)
U_{2222}	0.0(2)	0.0000004(0)	0.0000004(1)	-0.0021(1)	-0.00096(8)	-0.001(2)	0.0008(2)	-0.001(2)	-0.0000045(5)	0.000001(2)
U_{2223}	0.(2)	-0.00001(2)	-0.00001(4)	0.00002(2)	-0.000001(2)	-0.0000004(3)	-0.00002(3)	-0.00001(3)	-0.0000009(4)	0.000001(2)
U_{2233}	0.(2)	-0.00005(2)	-0.00005(2)	-0.000035(5)	-0.000025(2)	-0.000025(3)	0.000007(4)	-0.00001(2)	-0.0000054(5)	0.00004(2)
U_{2333}	0.0(2)	-0.000004(1)	-0.000004(6)	-0.000004(2)	0.0004(1)	0.0004(1)	-0.000001(2)	0.0006(2)	-0.000002(1)	-0.00001(7)
U_{3333}	0.(2)	-0.00001(1)	-0.00001(1)	-0.000027(1)	-0.000009(0)	-0.000009(1)	-0.0000063(9)	-0.000004(1)	-0.000035(5)	0.00003(1)

2 Energy of interactions

Table 23: Columns 1-4: RMSD (units: kJ/mol) between the dimer interaction energies obtained by the supramolecular approach for experimental and optimised crystal geometries. Columns 5-8: RMSD divided by mean absolute value of dimer interaction energy calculated for the optimised structures. The results are presented in the format: (with intensity cutoff / no intensity cutoff).

		RMSD [kJ/mol]				RMSD/mean(‖E(optimised)‖) [%]			
	Structure	PT-2(Cl)	PT-8(Br)	PT-10(I)	PT-11(S-Ph)	PT-2(Cl)	PT-8(Br)	PT-10(I)	PT-11(S-Ph)
harmonic	HAR_{anis}	0.64/0.63	0.86/0.96	1.90/0.94	0.36/0.39	3.7%/3.6%	5.3%/5.9%	11.0%/5.4%	1.7%/1.9%
	HAR_{iso}	0.55/0.50	0.96/1.03	1.62/0.90	0.32/0.35	3.2%/2.9%	5.9%/6.3%	9.4%/5.2%	1.6%/1.7%
	IAM	8.38	7.30	2.26	4.56	48.8%	44.9%	13.2%	22.2%
	MM	0.36/0.38	1.03/1.09	1.32/1.12	0.28/0.34	2.1%/2.2%	6.3%/6.7%	7.7%/6.5%	1.4%/1.7%
	MM(freeXH)	1.60/0.82			1.70/1.46	9.3%/4.8%			8.3%/7.1%
anharmonic (n=3)	HAR_{anis}	0.74/0.69	0.87/0.89	1.78/0.90	0.40/0.41	4.3%/4.0%	5.3%/5.5%	10.3%/5.2%	1.9%/2.0%
	MM	0.36/0.38	1.07/0.84	1.32/1.10	0.32/0.33	2.1%/2.2%	6.6%/5.2%	7.7%/6.4%	1.5%/1.6%
	MM(freeXH)	1.68/0.37			1.61/1.29	9.8%/2.1%			7.9%/6.3%
anharmonic (n=3, 4)	HAR_{anis}	0.37/0.38	0.85/0.89	1.83/1.37	0.42/0.45	2.1%/2.2%	5.2%/5.5%	10.7%/7.9%	2.0%/2.2%
	MM	0.37/0.39	1.03/1.00	1.34/1.11	0.32/0.33	2.1%/2.3%	6.3%/6.1%	7.8%/6.4%	1.6%/1.6%
	MM(freeXH)	1.69/0.64			1.61/1.38	9.8%/3.7%			7.9%/6.7%

Table 24: Energies of interactions of selected strongly interacting dimers. Units: kJ/mol. The results are presented in the format: (with intensity cutoff / no intensity cutoff).

	Structure<br>Dimer	PT-2(Cl)<br>dimer1	PT-8(Br)<br>dimer10	PT-10(I)<br>dimer2	PT-11(S-Ph)<br>dimer1
	optimised	-60.6	-55.7	-32.2	-52.8
harmonic	HAR_{anis}	-61.8/-61.8	-54.1/-53.7	-34.3/-34.3	-53.6/-53.6
	HAR_{iso}	-61.5/-61.4	-53.8/-53.5	-34.1/-34.2	-53.5/-53.5
	IAM	-40.1	-37.9	-35.1	-38.6
	MM	-60.3/-60.2	-53.4/-53.6	-33.6/-33.4	-52.4/-52.2
	MM(freeXH)	-64.4/-62.4			-47.5/-48.6
anharmonic (n=3)	HAR_{anis}	-62.1/-62.0	-54.0/-53.9	-34.4/-34.4	-53.8/-53.7
	MM	-60.3/-60.2	-53.3/-54.0	-33.6/-33.4	-52.2/-52.3
	MM(freeXH)	-64.6/-60.2			-47.8/-49.1
anharmonic (n=3, 4)	HAR_{anis}	-60.6/-60.7	-54.1/-54.0	-34.4/-34.5	-53.8/-53.9
	MM	-60.3/-60.1	-53.4/-54.0	-33.6/-33.3	-52.2/-52.3
	MM(freeXH)	-64.6/-61.9			-47.8/-48.8

Table 25: Energies of halogen bonds. Units: kJ/mol. The results are presented in the format: (with intensity cutoff / no intensity cutoff).

	Structure	PT-2(Cl)	PT-8(Br)	PT-10(I)
	optimised	-4.9	-7.1	-35.9
harmonic	HAR_{anis}	-4.7/-4.7	-6.8/-6.8	-32.3/-32.4
	HAR_{iso}	-4.7/-4.7	-6.8/-6.8	-32.4/-32.4
	IAM	-5.3	-7.2	-31.2
	MM	-4.7/-4.7	-6.8/-6.8	-33.0/-33.0
	MM(freeXH)	-4.5/-4.5		
anharmonic (n=3)	HAR_{anis}	-4.7/-4.7	-6.8/-6.8	-32.2/-32.2
	MM	-4.7/-4.7	-6.7/-6.8	-33.0/-33.1
	MM(freeXH)	-4.5/-4.5		
anharmonic (n=3, 4)	HAR_{anis}	-4.7/-4.7	-6.8/-6.8	-32.0/-32.0
	MM	-4.7/-4.7	-6.8/-6.8	-32.9/-33.0
	MM(freeXH)	-4.5/-4.6		

Table 26: Cohesive energies. Units: kJ/mol. The results are presented in the format: (with intensity cutoff / no intensity cutoff).

	Structure	PT-2(Cl)	PT-8(Br)	PT-10(I)	PT-11(S-Ph)
	optimised	-97.2	-96.4	-111.8	-114.3
harmonic	HAR_{anis}	-93.1/-93.1	-89.5/-89.4	-42.2/-41.8	-108.1/-108.1
	HAR_{iso}	-92.9/-93.0	-89.2/-89.2	-40.7/-40.2	-108.6/-108.6
	IAM	150.4	172.9	486.8	281.1
	MM	-94.3/-94.5	-95.9/-96.5	-98.0/-99.9	-110.9/-110.9
	MM(freeXH)	-79.0/-81.1			-92.9/-100.9
anharmonic (n=3)	HAR_{anis}	-93.1/-93.1	-90.6/-89.6	-38.1/-37.2	-108.1/-108.1
	MM	-94.3/-94.5	-96.0/-96.5	-98.6/-101.8	-110.7/-110.9
	MM(freeXH)	-80.3/-81.5			-92.9/-102.1
anharmonic (n=3, 4)	HAR_{anis}	-94.2/-94.2	-89.0/-88.8	-24.6/-22.1	-107.8/-107.8
	MM	-94.3/-94.5	-95.8/-96.3	-99.1/-102.4	-110.6/-110.9
	MM(freeXH)	-79.0/-83.3			-133.0/-101.9

Table 27: Geometrical relaxation energies. Units: kJ/mol. The results are presented in the format: (with intensity cutoff / no intensity cutoff).

	Structure	PT-2(Cl)	PT-8(Br)	PT-10(I)	PT-11(S-Ph)
harmonic	optimised	-5.6	-7.3	-3.6	-8.9
	HAR_{anis}	-9.3/-9.2	-13.7/-13.7	-72.2/-72.6	-15.7/-15.7
	HAR_{iso}	-9.3/-9.2	-13.9/-13.9	-74.5/-75.0	-14.9/-14.9
	IAM	-247.9	-273.2	-606.4	-404.7
	MM	-8.5/-8.5	-5.9/-5.6	-15.5/-13.9	-12.4/-12.2
	MM(freeXH)	-23.9/-21.2			-27.5/-19.4
anharmonic (n=3)	HAR_{anis}	-9.5/-9.4	-13.6/-13.6	-75.8/-77.0	-15.7/-15.7
	MM	-8.3/-8.5	-5.8/-5.7	-14.6/-11.6	-12.6/-12.2
	MM(freeXH)	-22.8/-20.7			-27.6/-18.4
anharmonic (n=3, 4)	HAR_{anis}	-8.0/-7.9	-14.5/-14.6	-89.7/-92.2	-16.15/-16.12
	MM	-8.4/-8.5	-6.0/-5.9	-14.0/-11.0	-12.7/-12.2
	MM(freeXH)	-21.8/-18.8			-27.9/-18.5

3 Fractal dimension plots

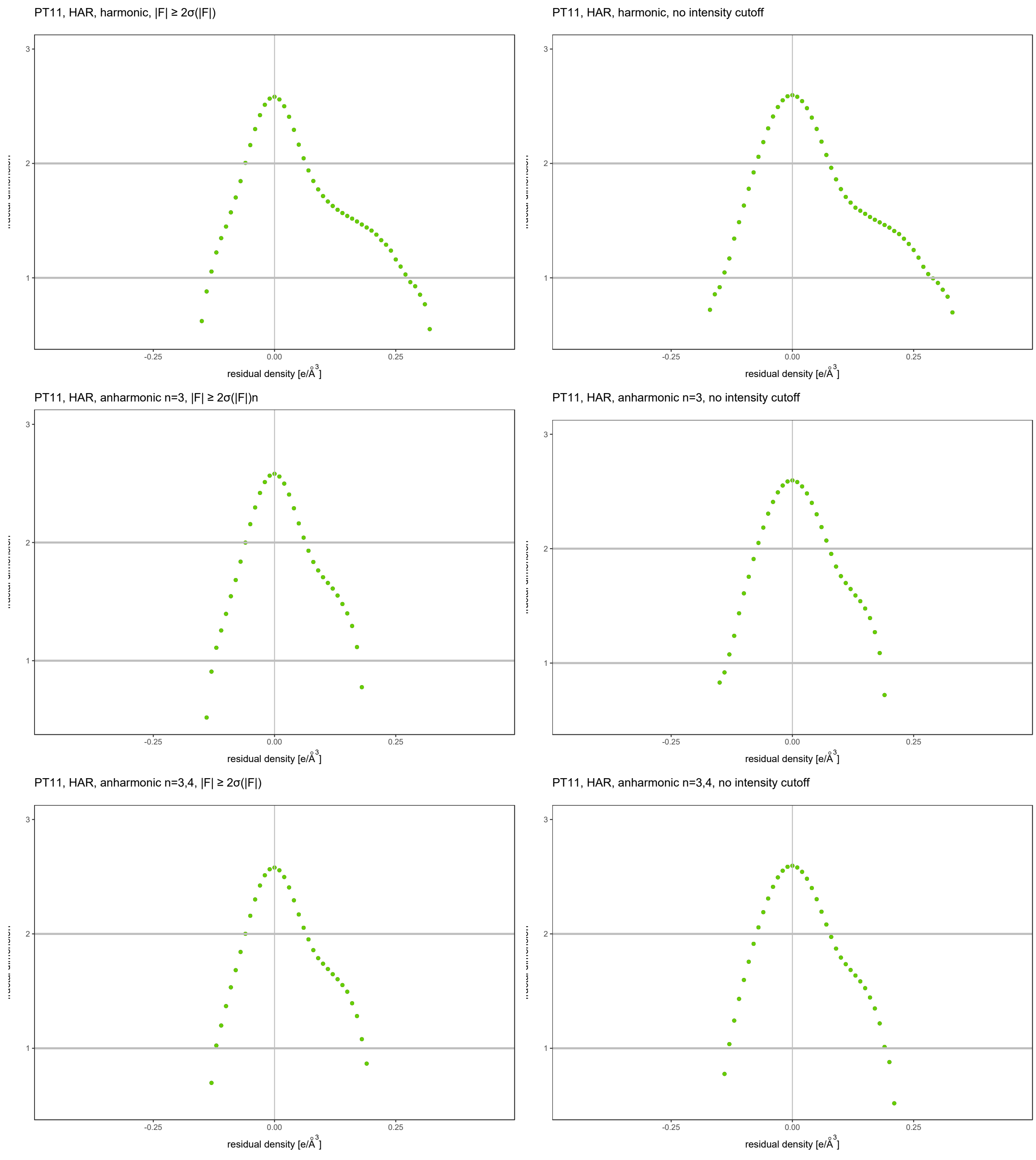

Figure 1: Fractal dimension plots for PT-11, HAR, left: $|F| \geq 2\sigma(|F|)$ and right: all reflections included.

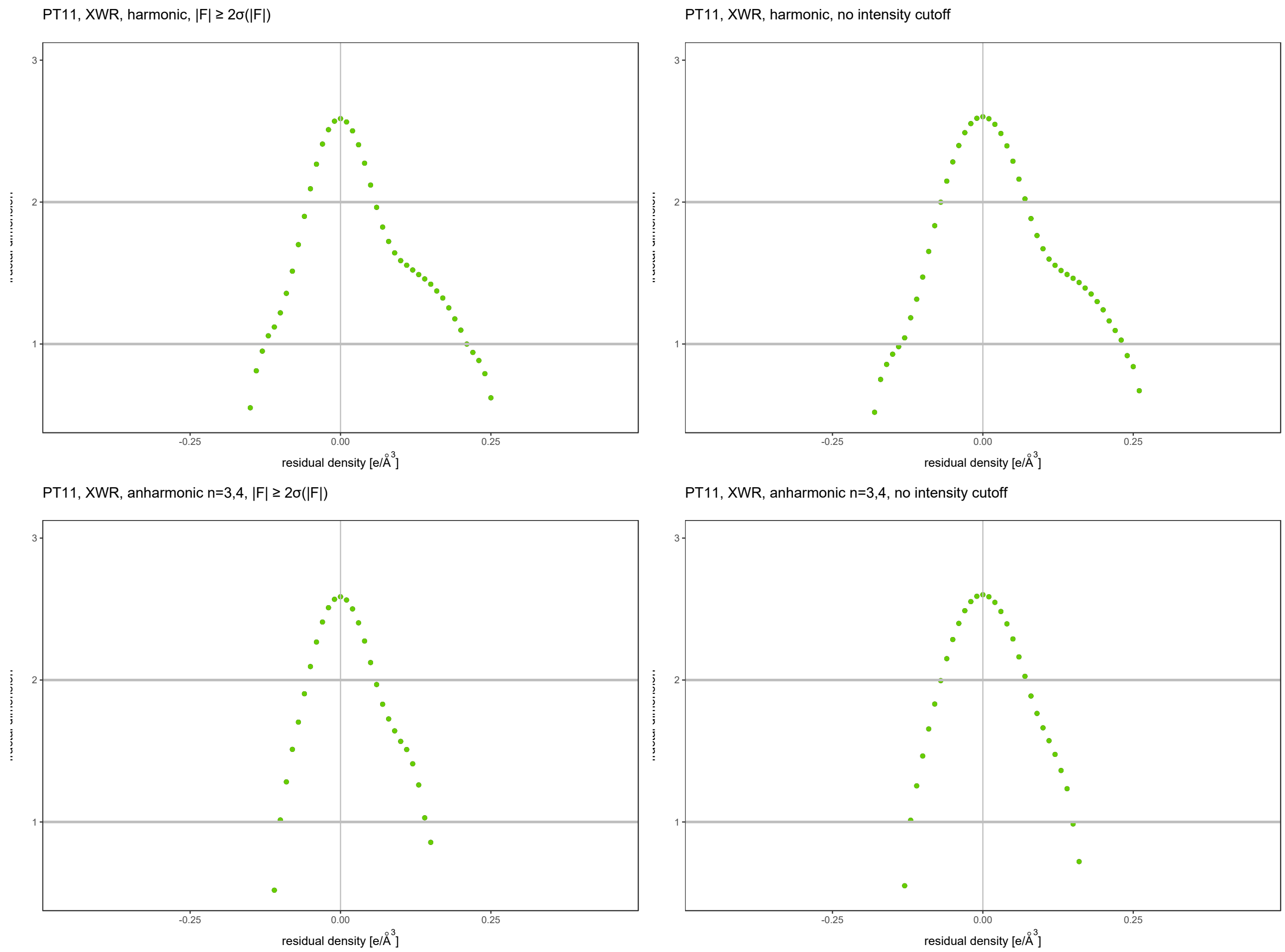

Figure 2: Fractal dimension plots for PT-11, XWR, left: $|F| \geq 2\sigma(|F|)$ and right: all reflections included.

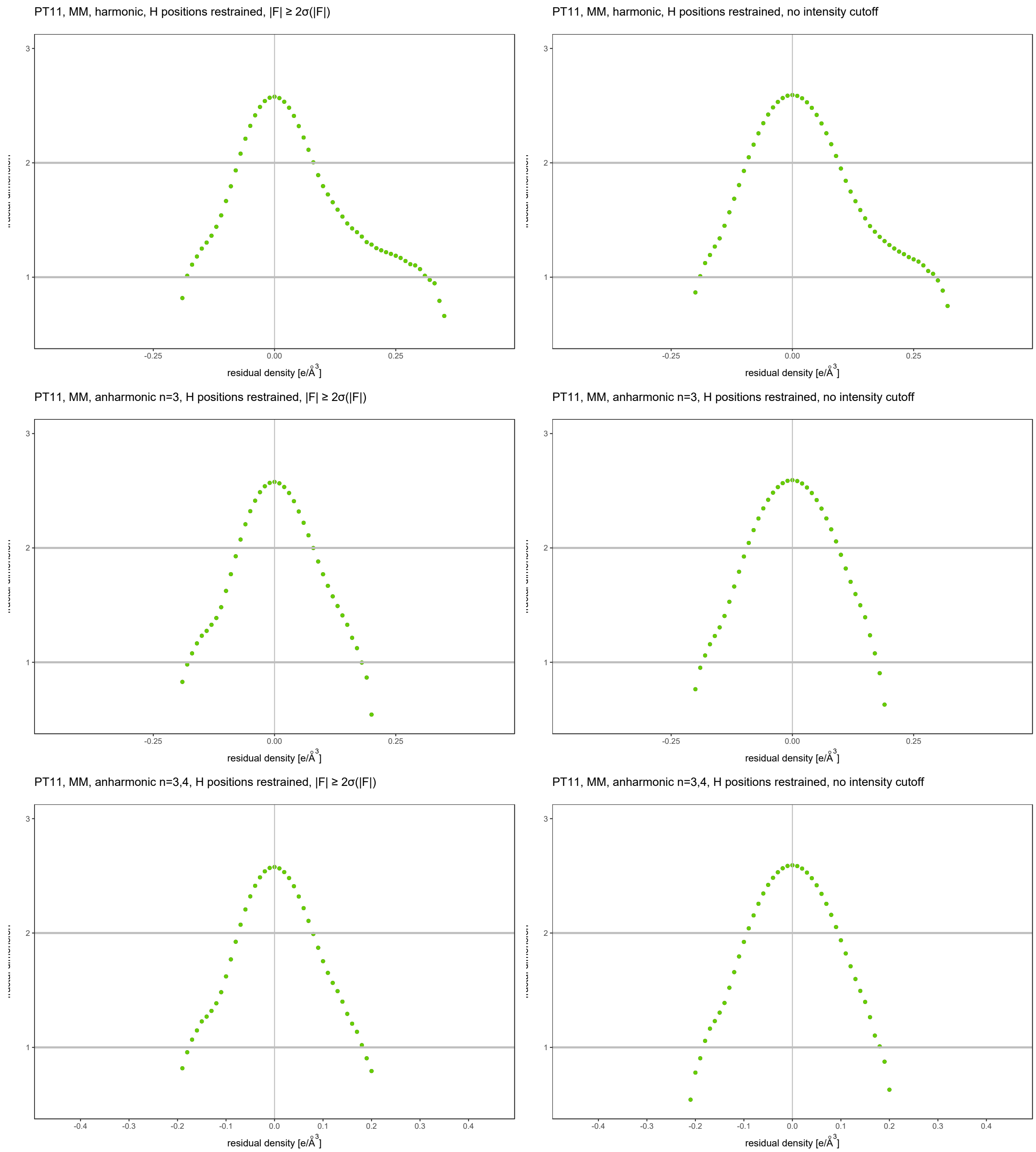

Figure 3: Fractal dimension plots for PT-11, MM, restrained X-H distances, left: $|F| \geq 2\sigma(|F|)$ and right: all reflections included.

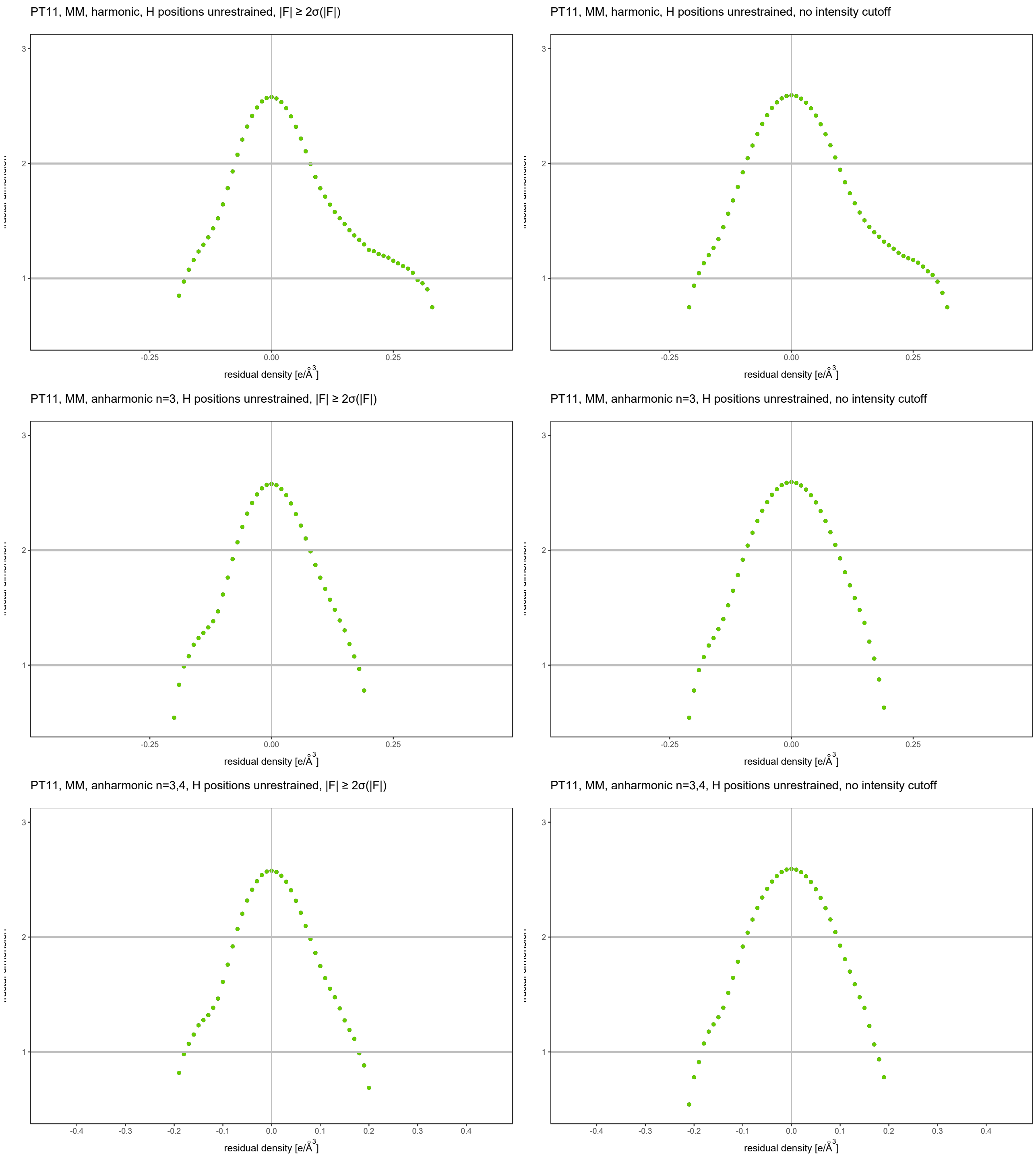

Figure 4: Fractal dimension plots for PT-11, MM, unrestrained X-H distances, left: $|F| \geq 2\sigma(|F|)$ and right: all reflections included.

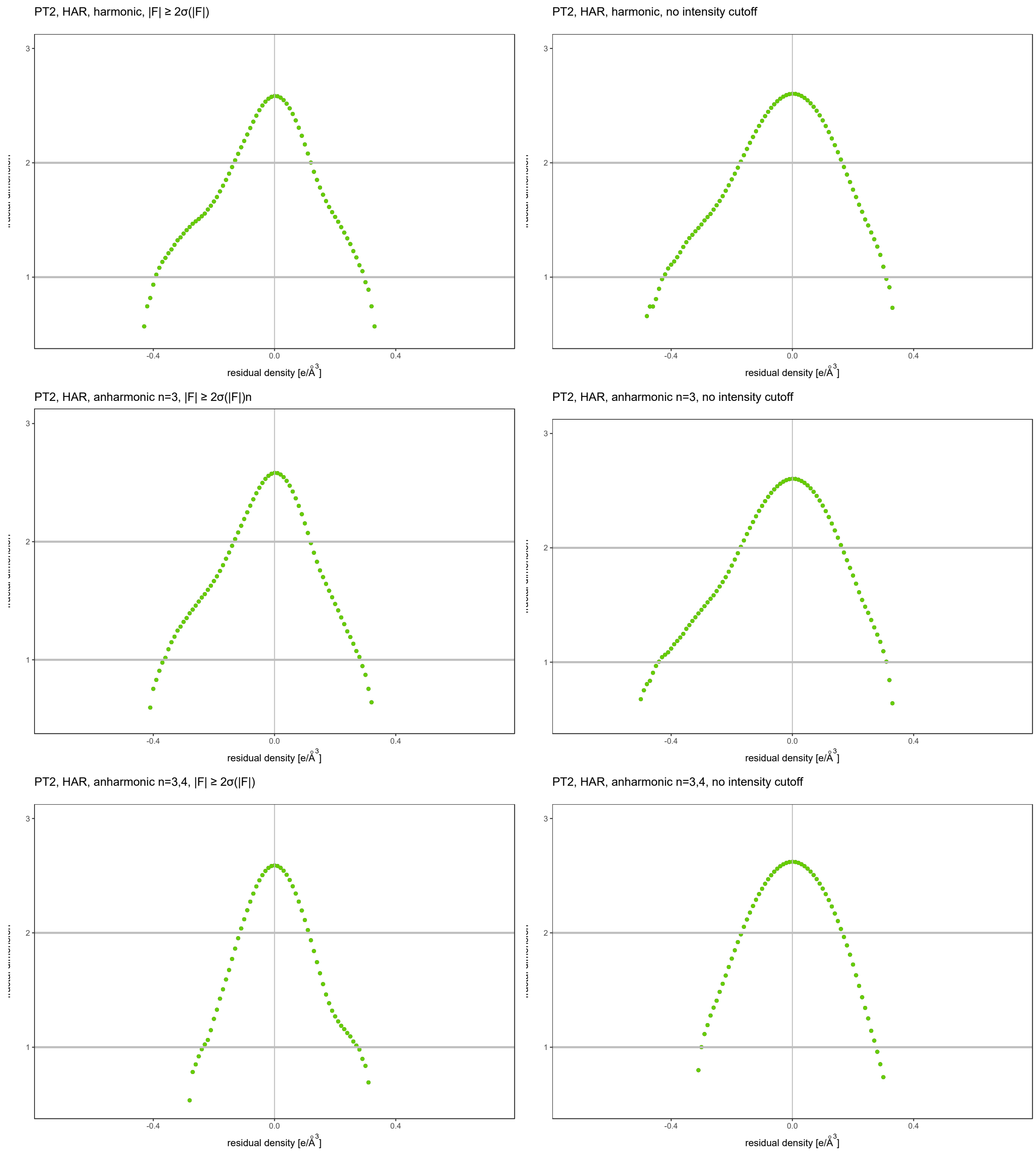

Figure 5: Fractal dimension plots for PT-2, HAR, left: $|F| \geq 2\sigma(|F|)$ and right: all reflections included.

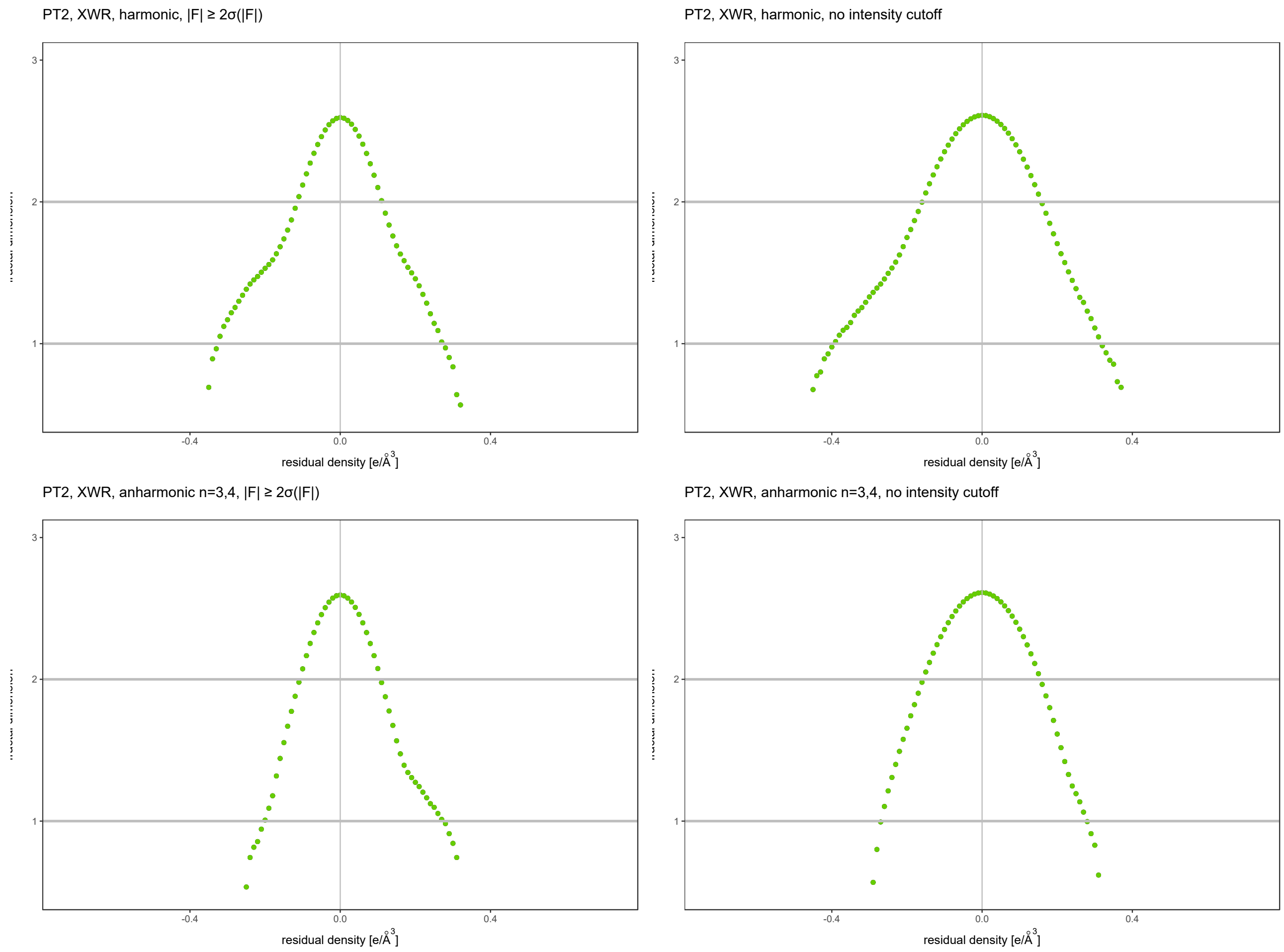

Figure 6: Fractal dimension plots for PT-2, XWR, left: $|F| \geq 2\sigma(|F|)$ and right: all reflections included.

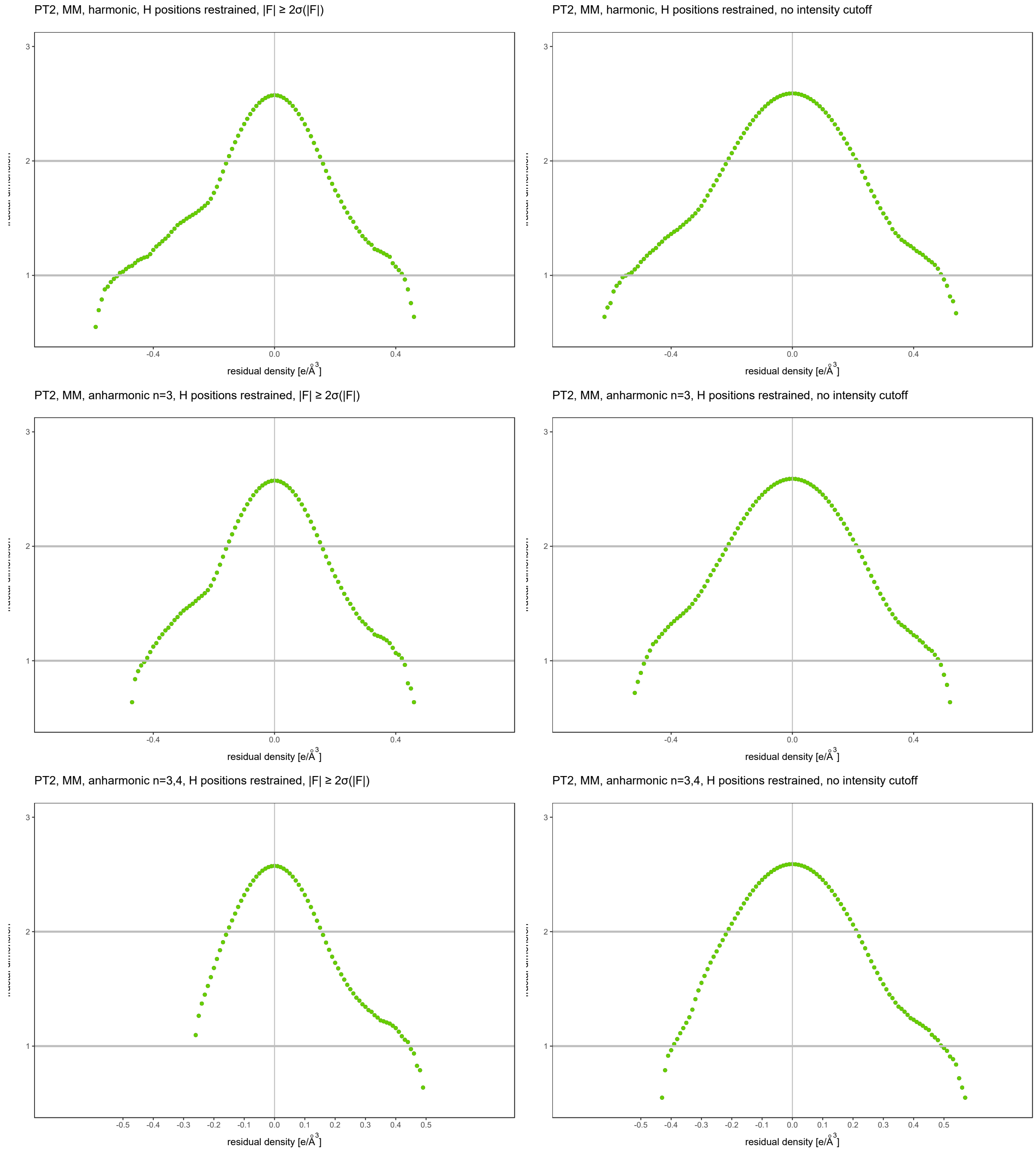

Figure 7: Fractal dimension plots for PT-2, MM, restrained X-H distances, left: $|F| \geq 2\sigma(|F|)$ and right: all reflections included.

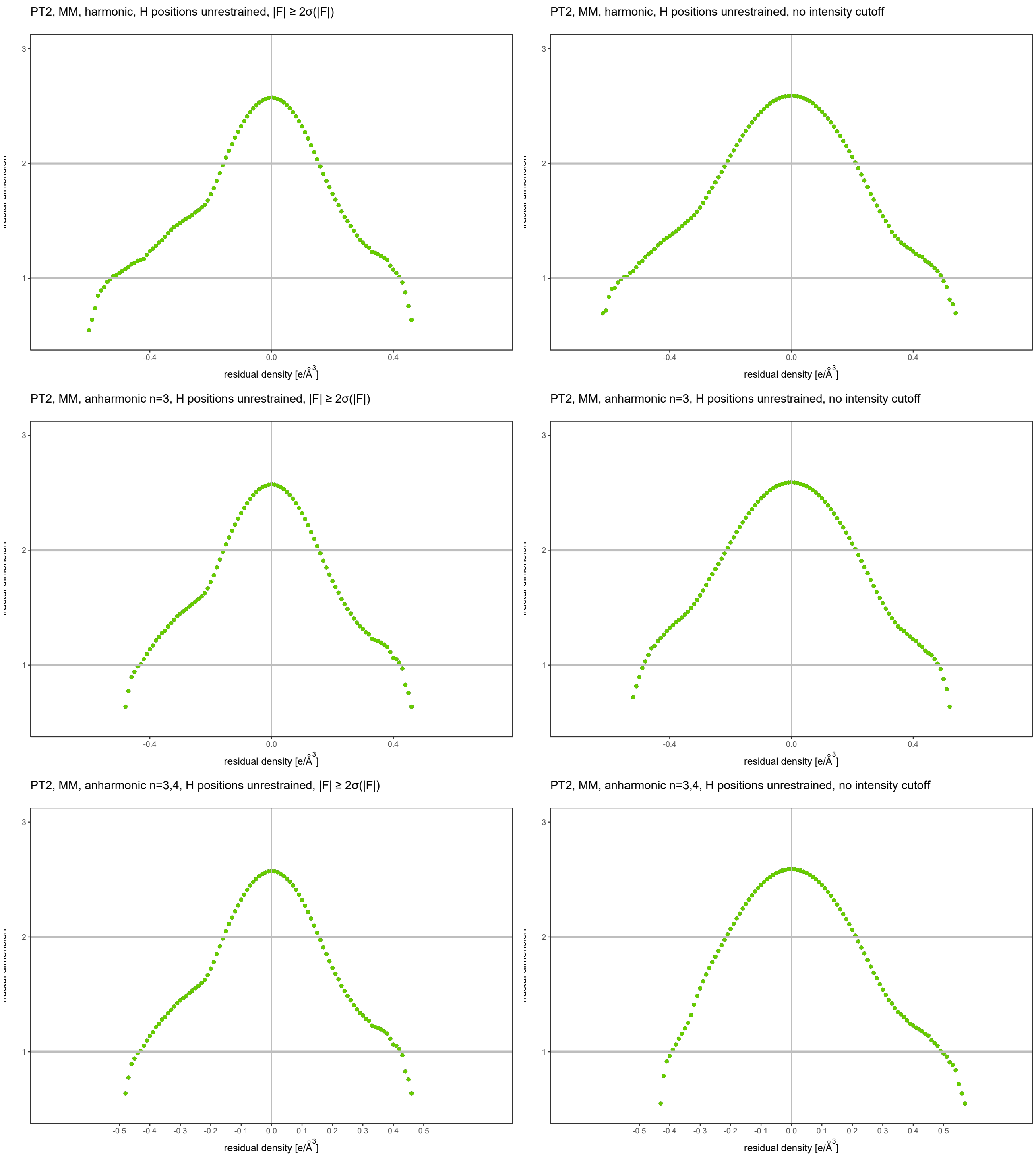

Figure 8: Fractal dimension plots for PT-2, MM, unrestrained X-H distances, left: $|F| \geq 2\sigma(|F|)$ and right: all reflections included.

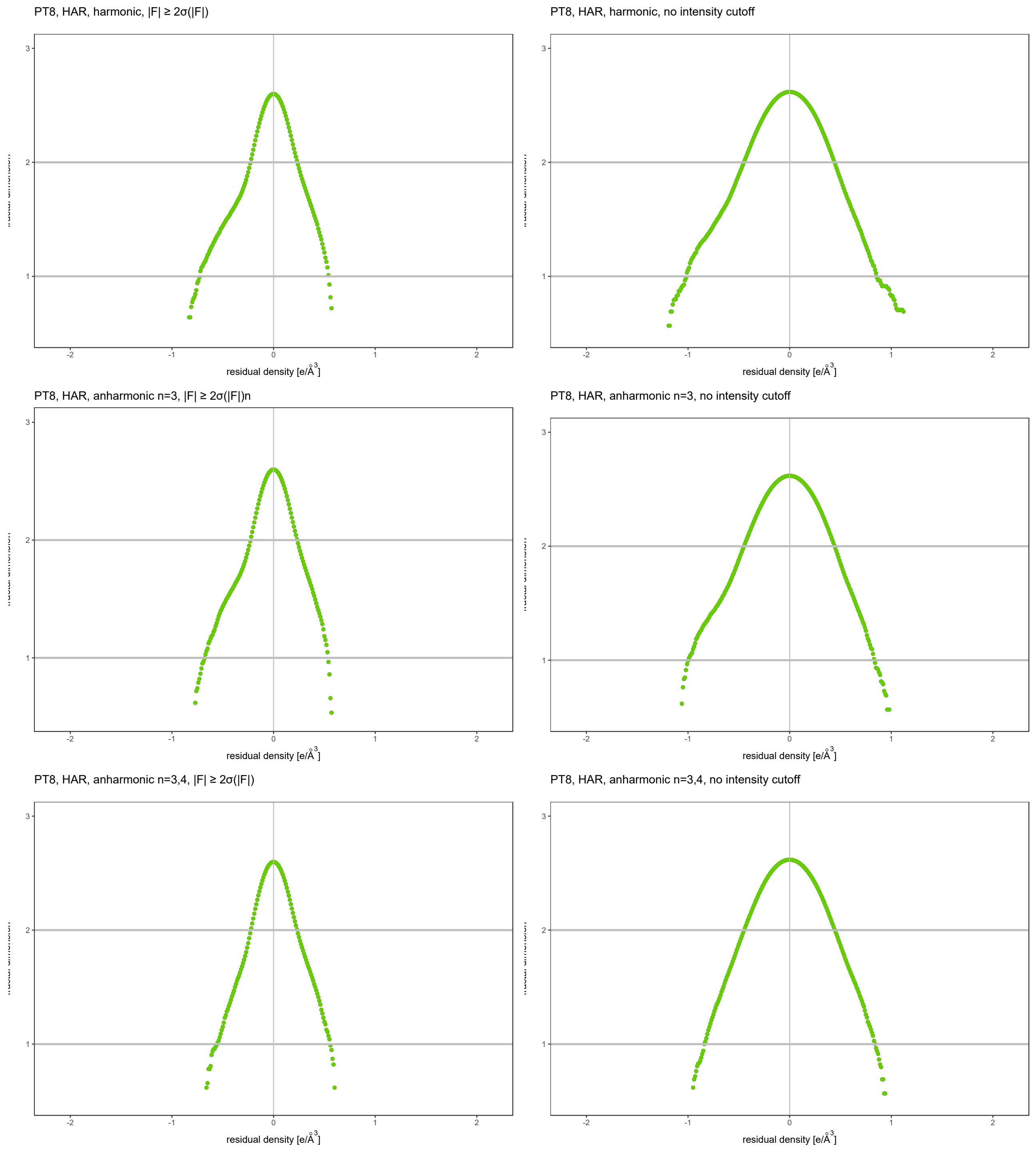

Figure 9: Fractal dimension plots for PT-8, HAR, left: $|F| \geq 2\sigma(|F|)$ and right: all reflections included.

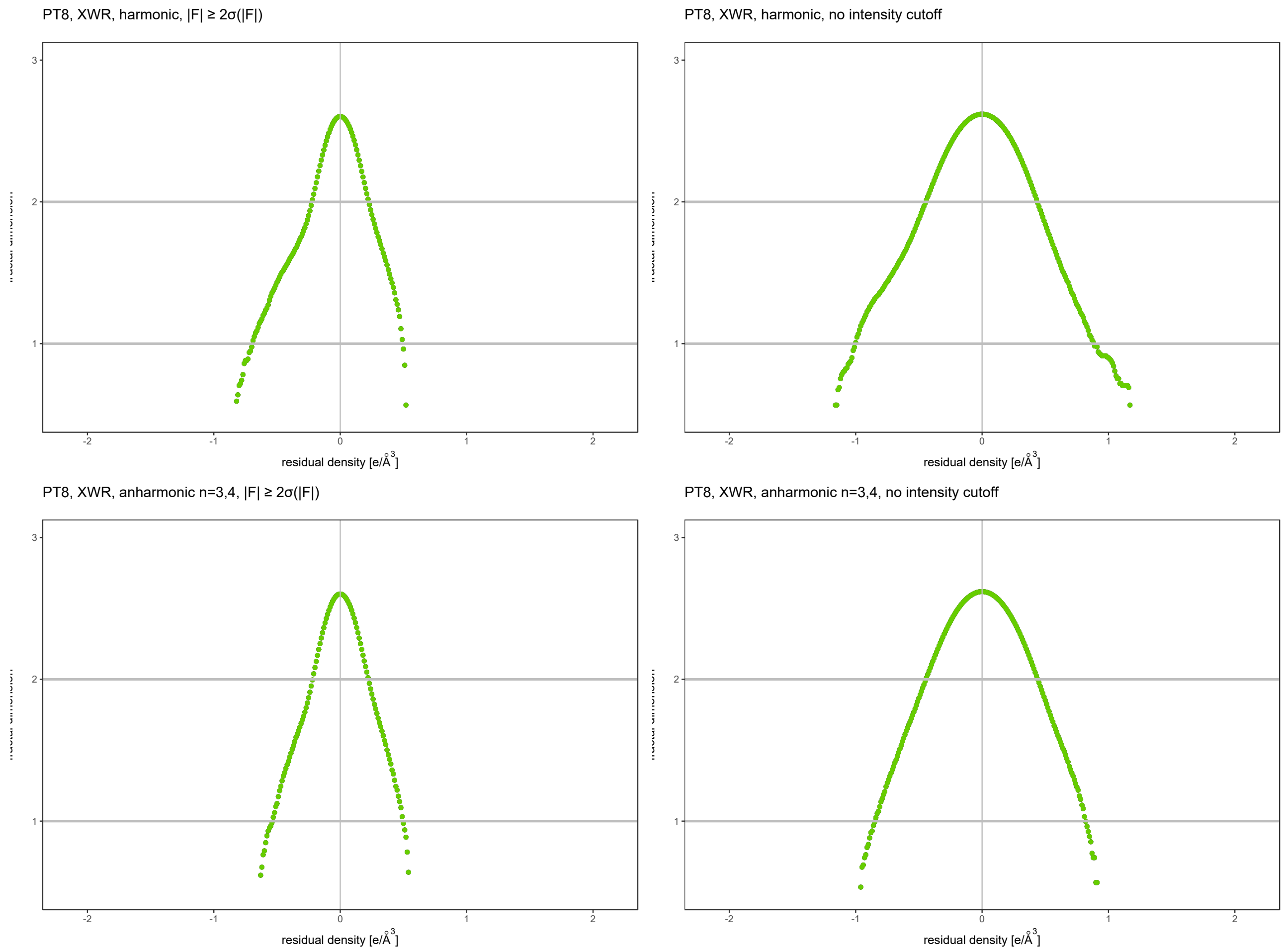

Figure 10: Fractal dimension plots for PT-8, XWR, left: $|F| \geq 2\sigma(|F|)$ and right: all reflections included.

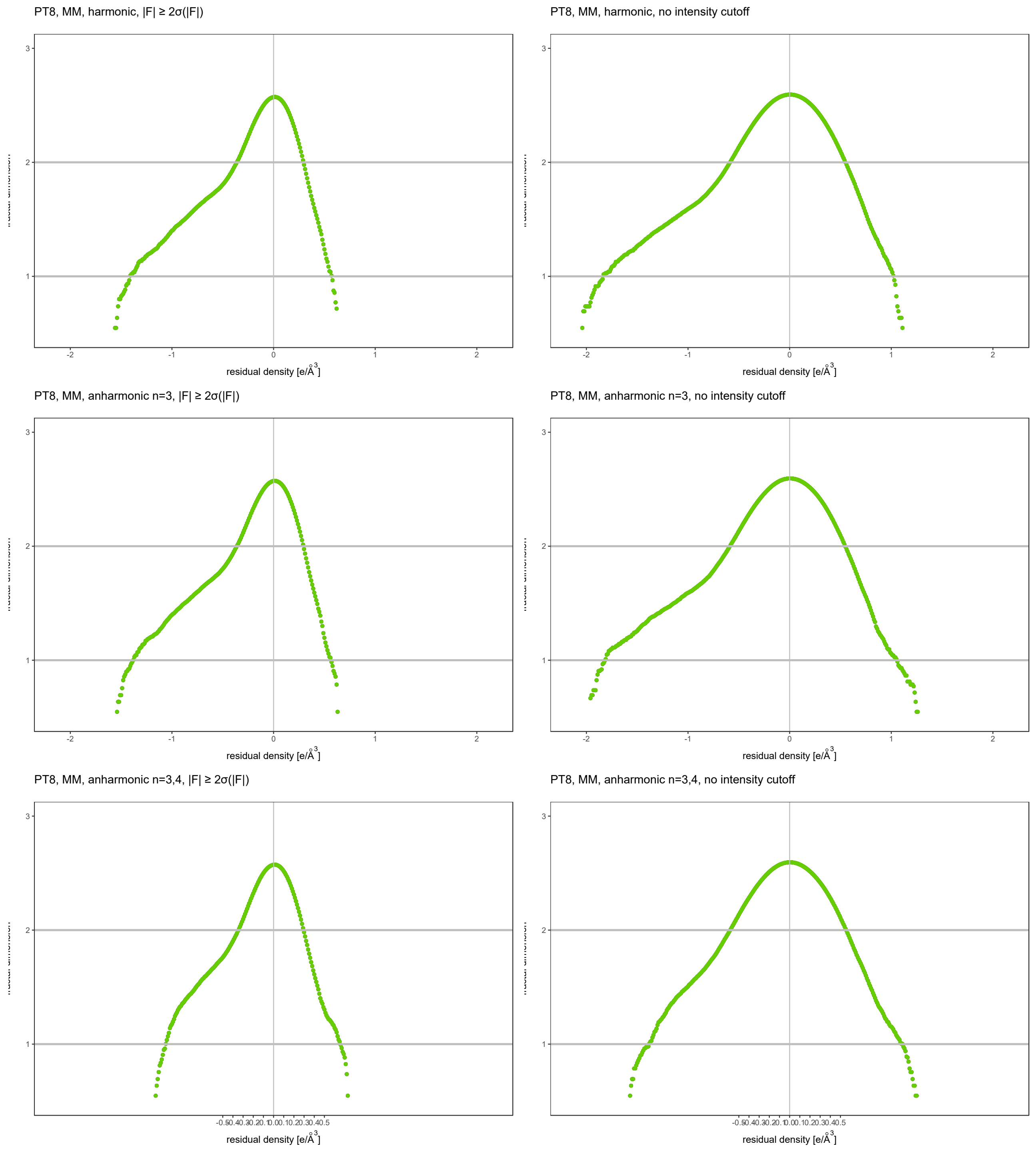

Figure 11: Fractal dimension plots for PT-8, MM, restrained X-H distances, left: $|F| \geq 2\sigma(|F|)$ and right: all reflections included.

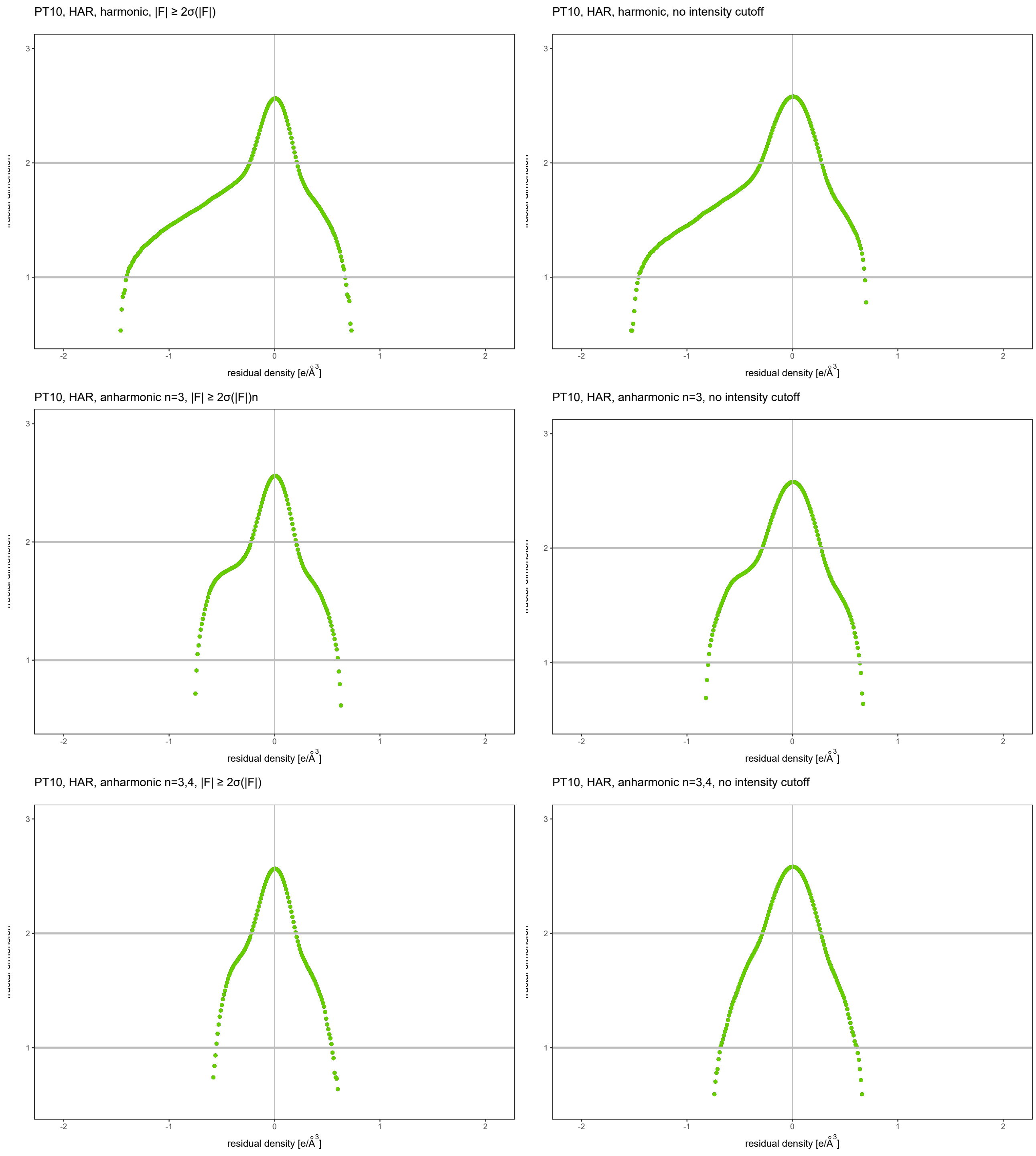

Figure 12: Fractal dimension plots for PT-10, HAR, left: $|F| \geq 2\sigma(|F|)$ and right: all reflections included.

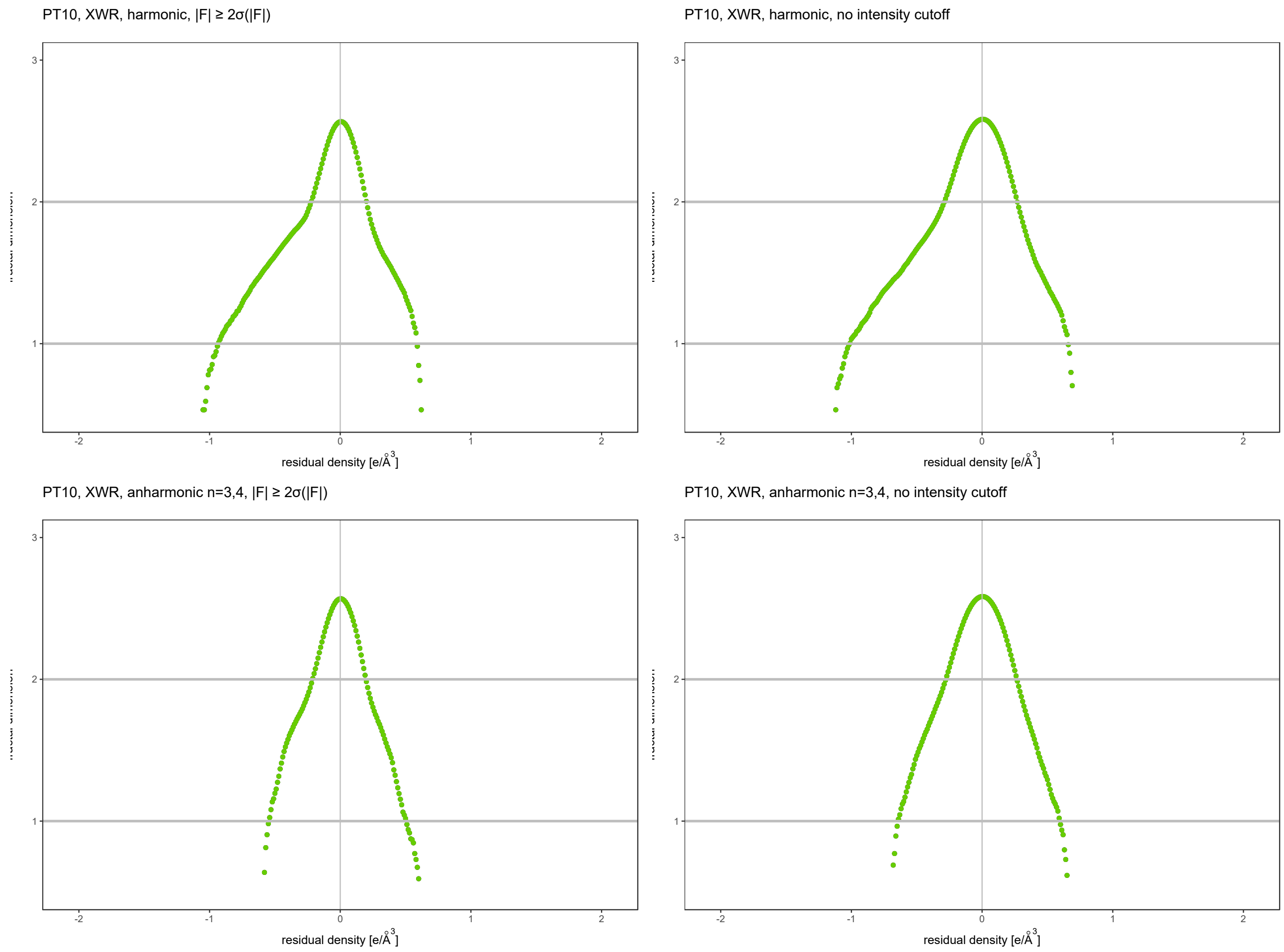

Figure 13: Fractal dimension plots for PT-10, XWR, left: $|F| \geq 2\sigma(|F|)$ and right: all reflections included.

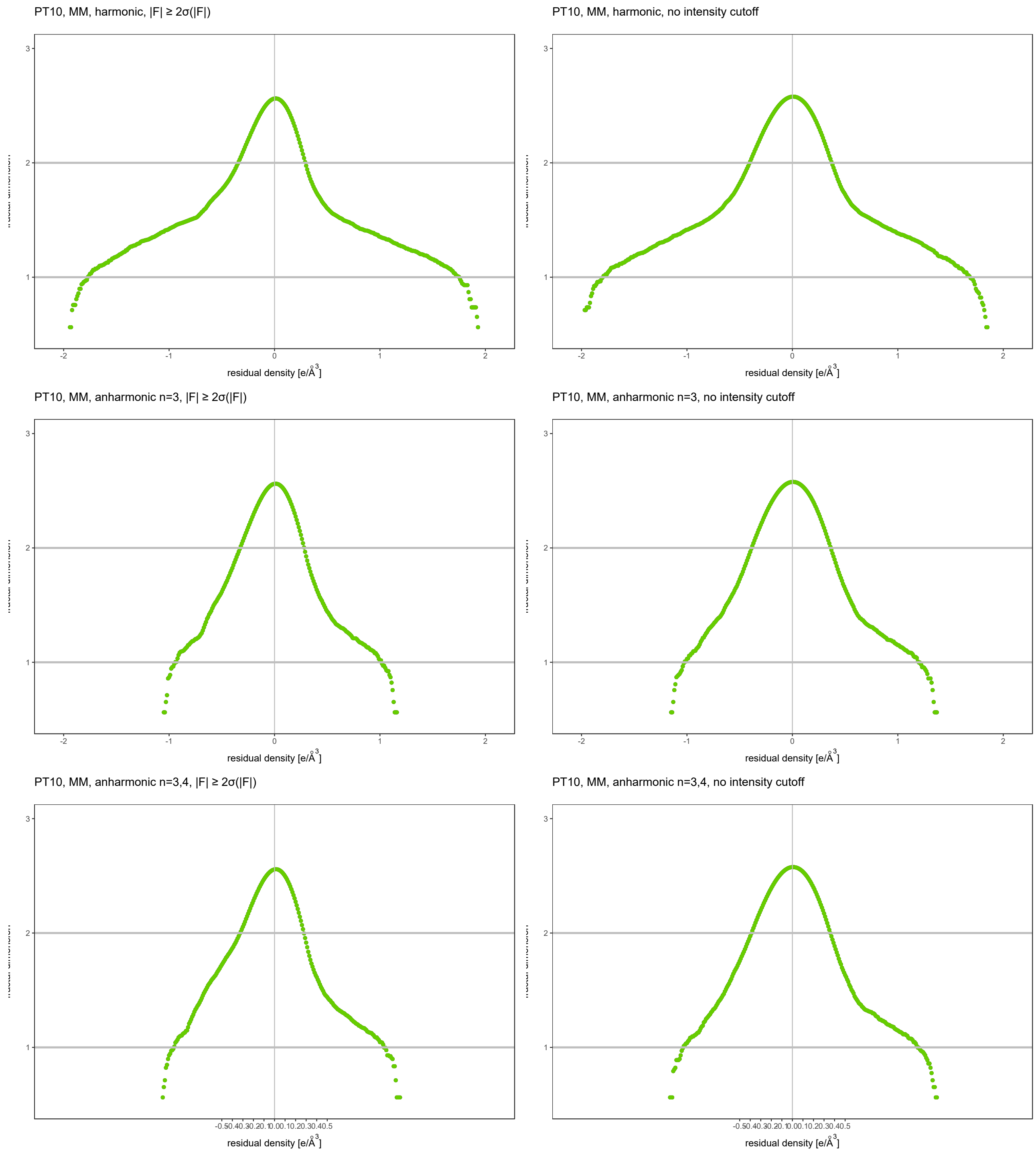

Figure 14: Fractal dimension plots for PT-10, MM, restrained X-H distances, left: $|F| \geq 2\sigma(|F|)$ and right: all reflections included.

4 Residaual density maps

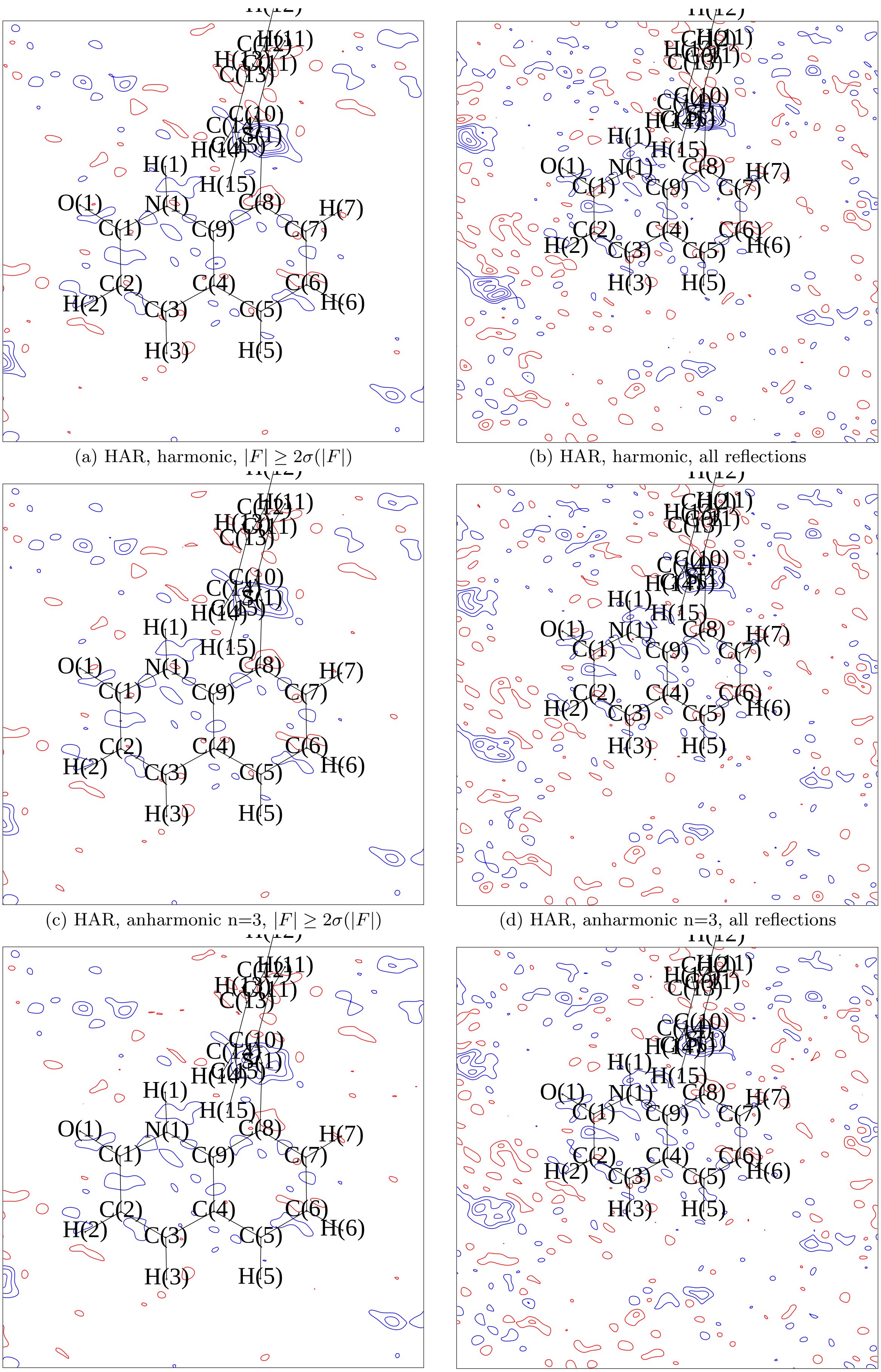

(a) HAR, harmonic, $|F| \geq 2\sigma(|F|)$

(b) HAR, harmonic, all reflections

(c) HAR, anharmonic n=3, $|F| \geq 2\sigma(|F|)$

(d) HAR, anharmonic n=3, all reflections

(e) HAR, anharmonic n=4, $|F| \geq 2\sigma(|F|)$

(f) HAR, anharmonic n=4, all reflections

Figure 15: Residual density maps for PT-11 in the plane of quinoline, HAR, left: $|F| \geq 2\sigma(|F|)$ and right: all reflections included. Contour level: 0.05 e/Å^3. Colours: blue - positive, red – negative.

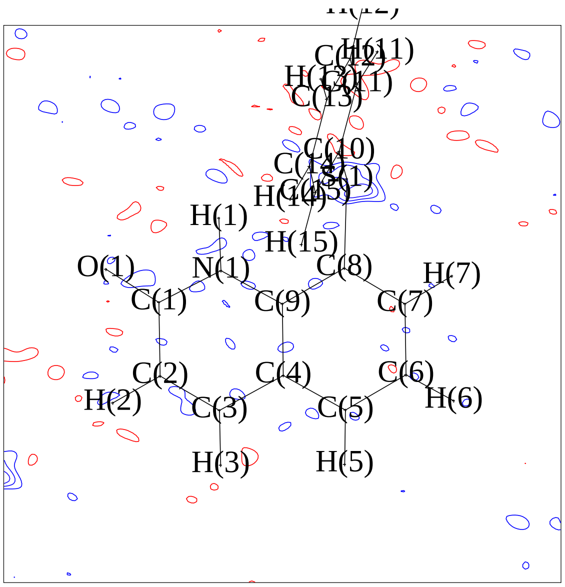

(a) XWR, harmonic, $|F| \geq 2\sigma(|F|)$

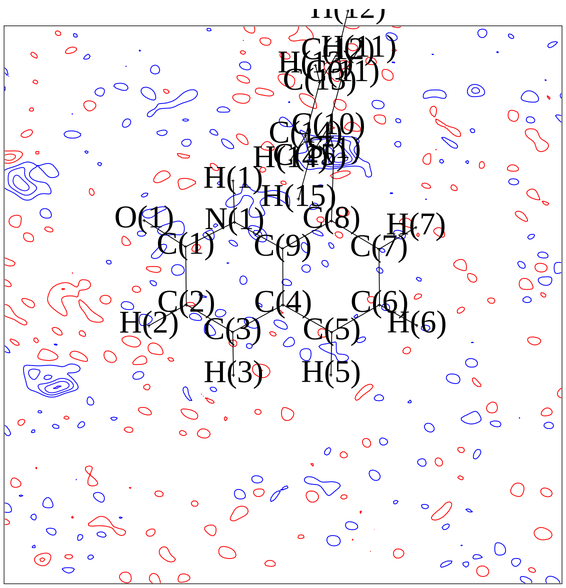

(b) XWR, harmonic, all reflections

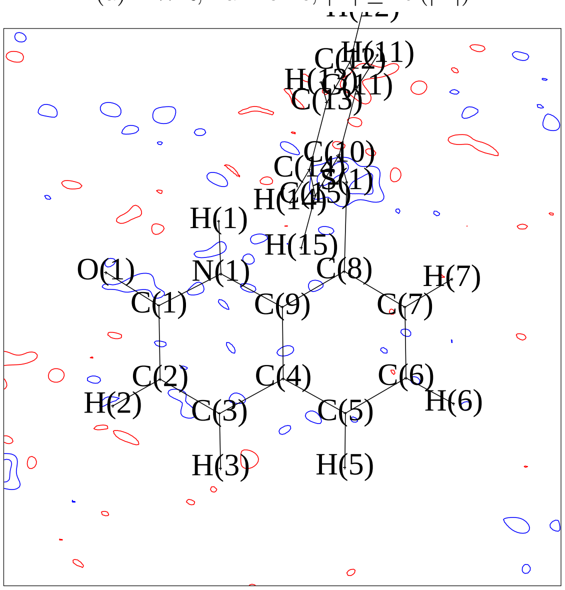

(c) XWR, anharmonic n=4, $|F| \geq 2\sigma(|F|)$

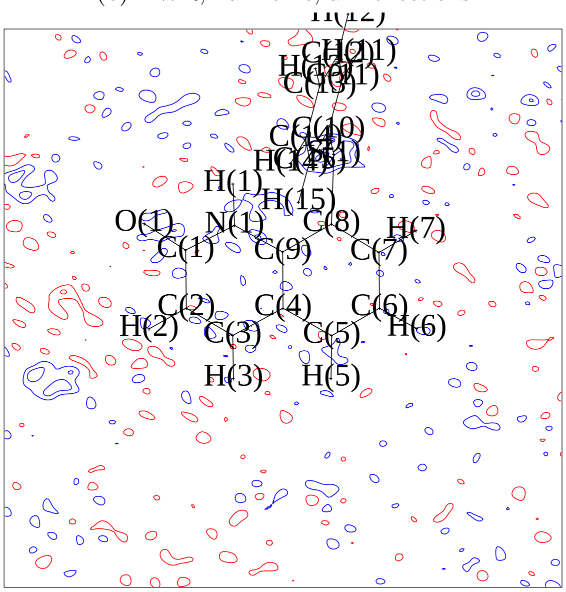

(d) XWR, anharmonic n=4, all reflections

Figure 16: Residual density maps for PT-11 in the plane of quinoline, XWR, left: $|F| \geq 2\sigma(|F|)$ and right: all reflections included. Contour level: 0.05 e/Å^3. Colours: blue - positive, red – negative.

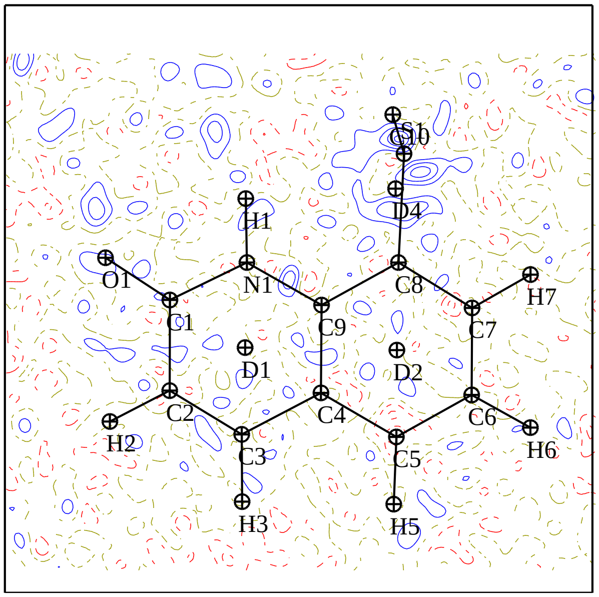

(a) MM, harmonic, $|F| \geq 2\sigma(|F|)$

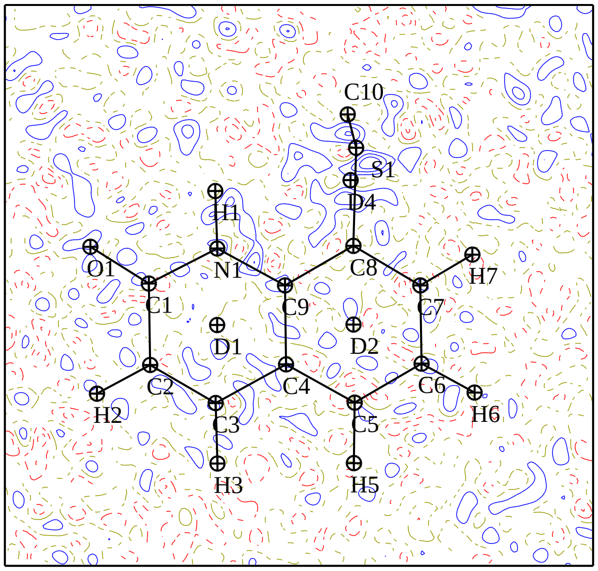

(b) MM, harmonic, all reflections

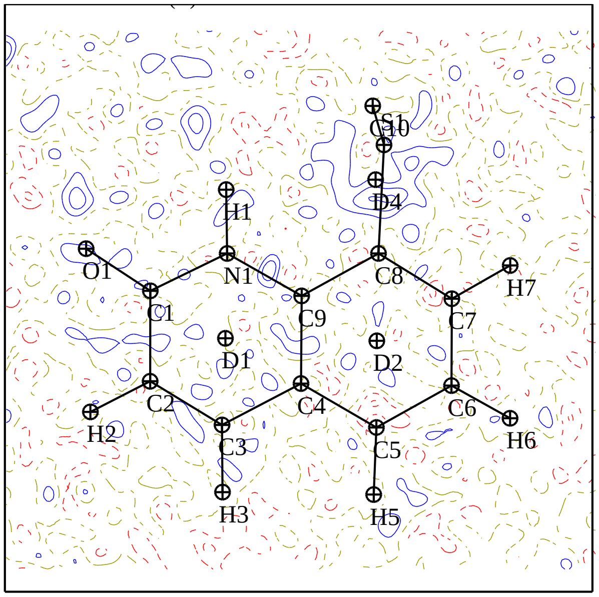

(c) MM, anharmonic n=3, $|F| \geq 2\sigma(|F|)$

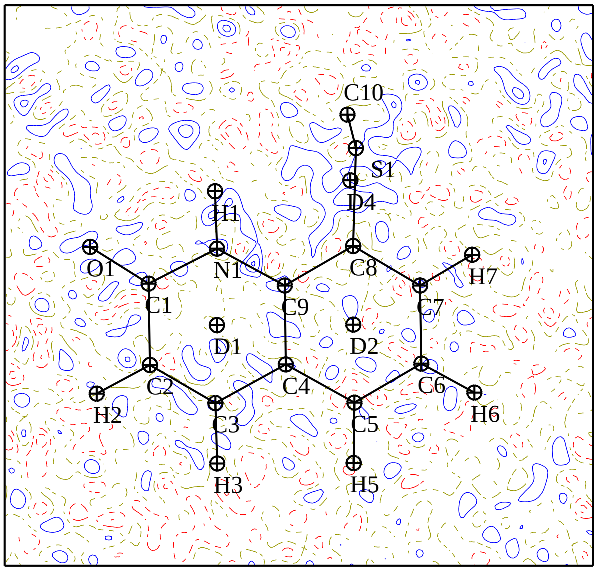

(d) MM, anharmonic n=3, all reflections

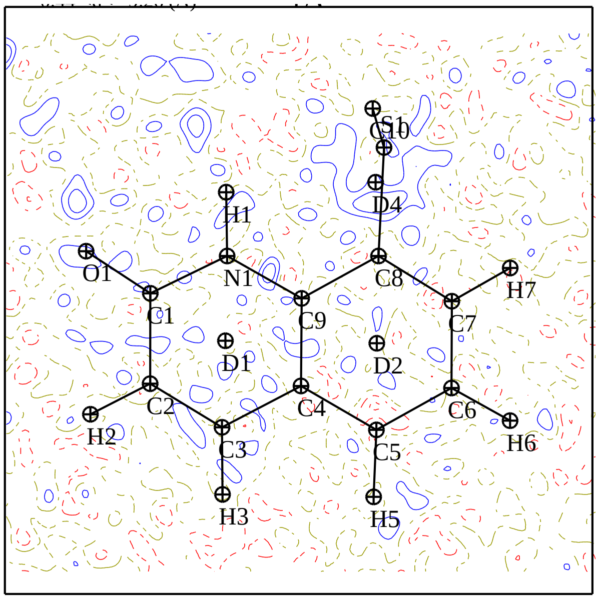

(e) MM, anharmonic n=4, $|F| \geq 2\sigma(|F|)$

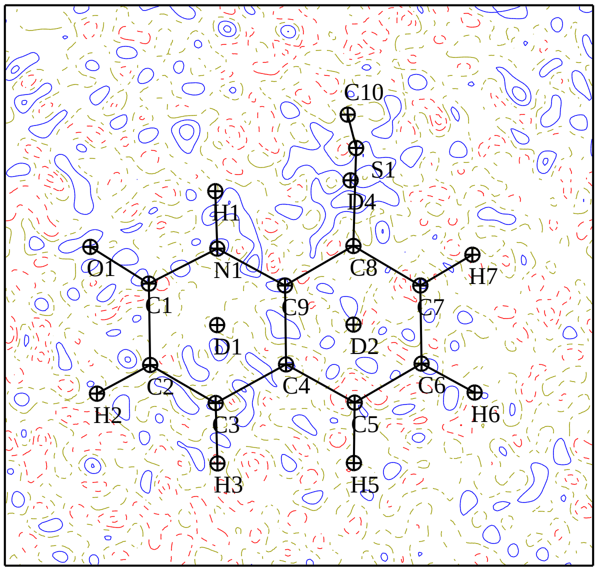

(f) MM, anharmonic n=4, all reflections

Figure 17: Residual density maps for PT-11 in the plane of quinoline, MM, restrained X-H distances, left: $|F| \geq 2\sigma(|F|)$ and right: all reflections included. Contour level: 0.05 e/Å^3. Colours: blue - positive, red – negative.

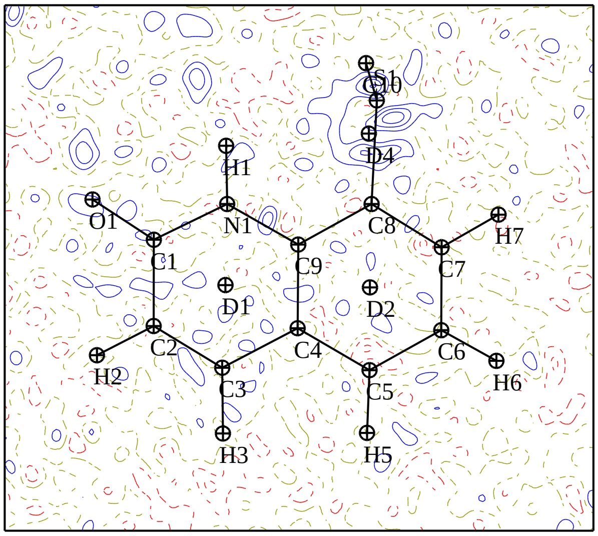

(a) MM, harmonic, $|F| \geq 2\sigma(|F|)$

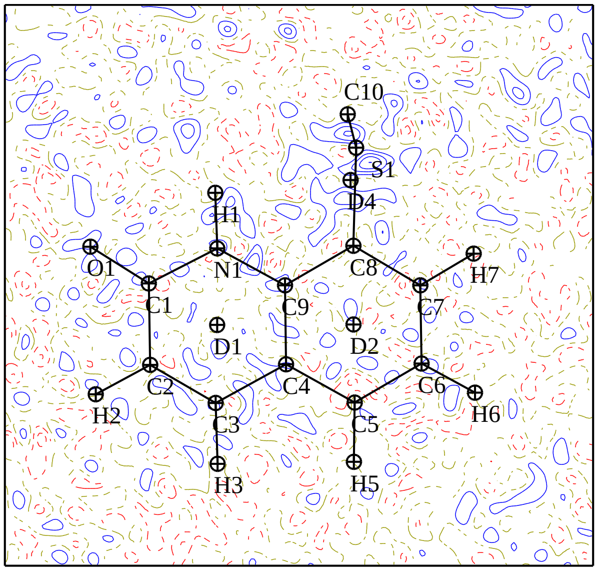

(b) MM, harmonic, all reflections

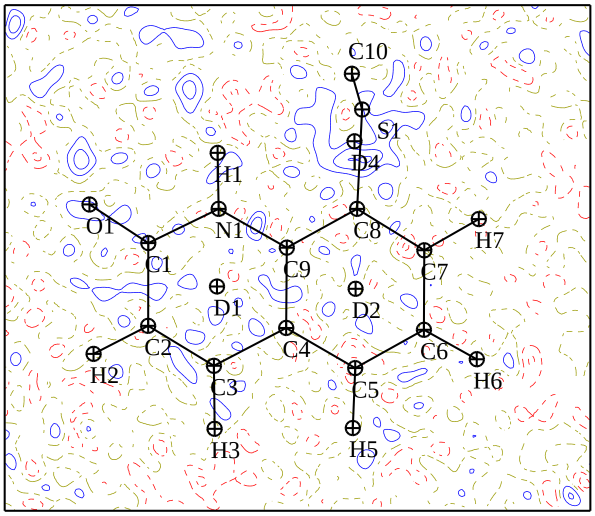

(c) MM, anharmonic n=3, $|F| \geq 2\sigma(|F|)$

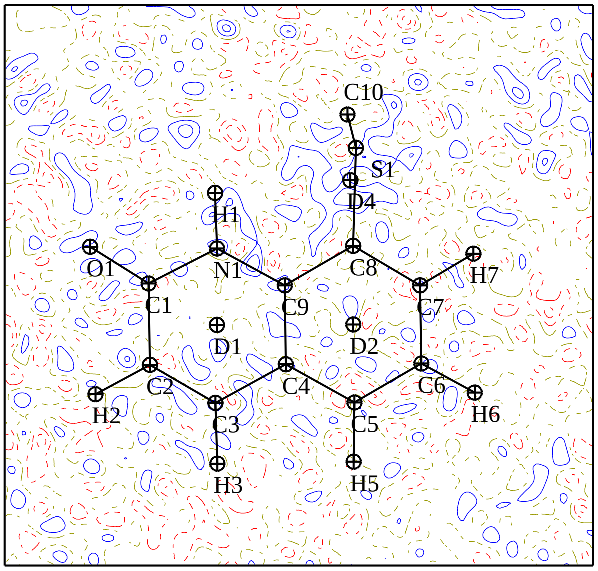

(d) MM, anharmonic n=3, all reflections

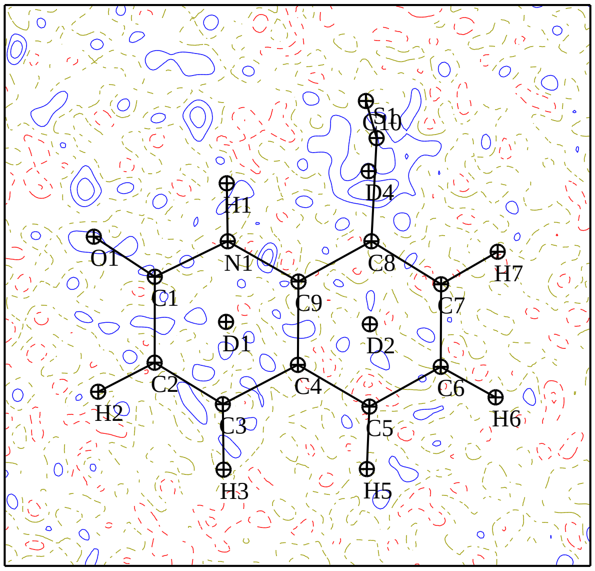

(e) MM, anharmonic n=4, $|F| \geq 2\sigma(|F|)$

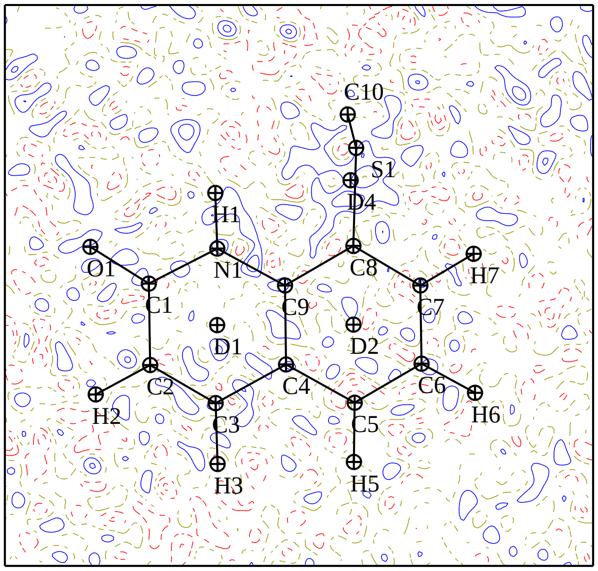

(f) MM, anharmonic n=4, all reflections

Figure 18: Residual density maps for PT-11 in the plane of quinoline, MM, unrestrained X-H distances, left: $|F| \geq 2\sigma(|F|)$ and right: all reflections included. Contour level: 0.05 e/Å^3. Colours: blue - positive, red – negative.

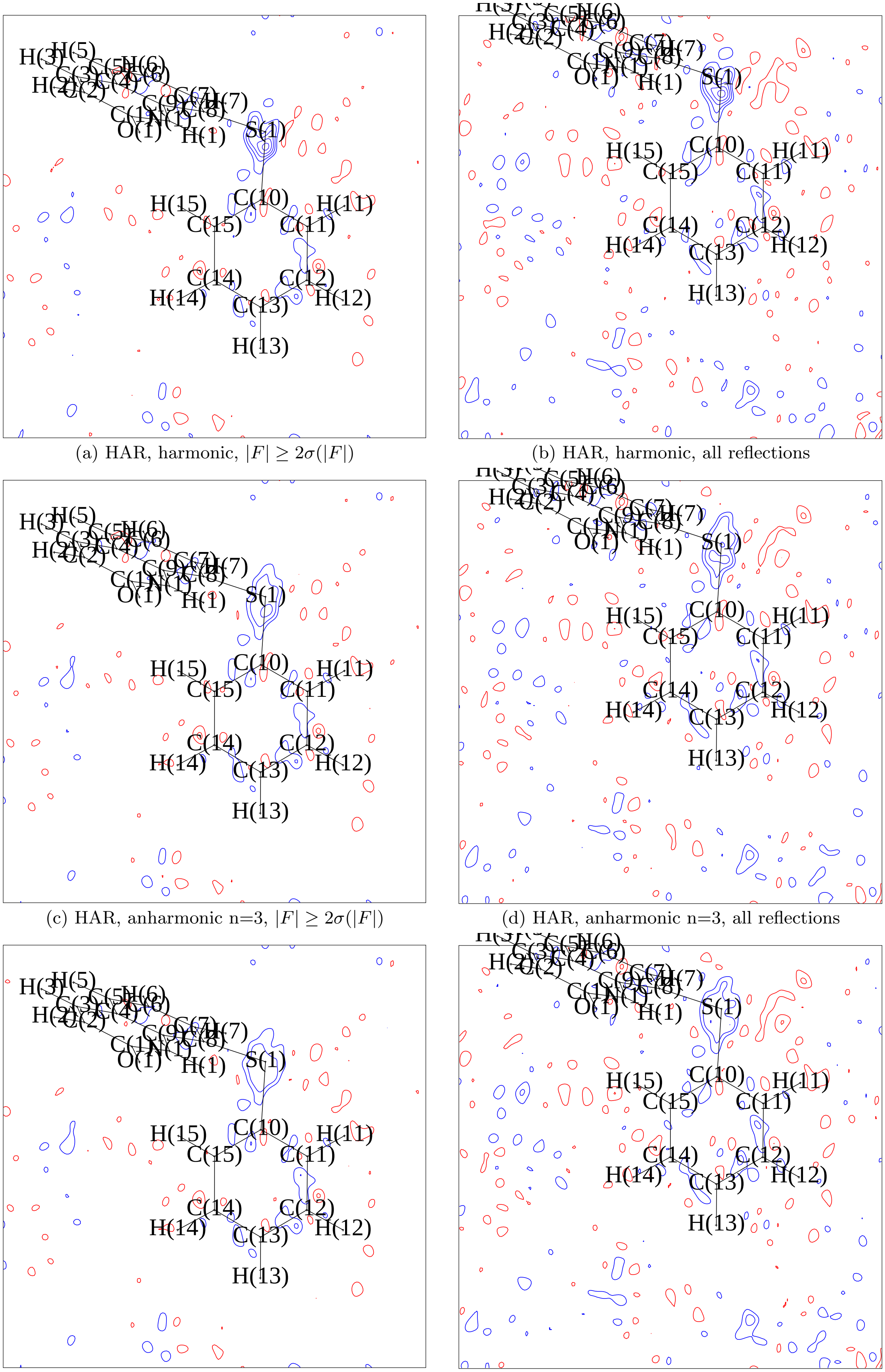

(a) HAR, harmonic, $|F| \geq 2\sigma(|F|)$

(b) HAR, harmonic, all reflections

(c) HAR, anharmonic n=3, $|F| \geq 2\sigma(|F|)$

(d) HAR, anharmonic n=3, all reflections

(e) HAR, anharmonic n=4, $|F| \geq 2\sigma(|F|)$

(f) HAR, anharmonic n=4, all reflections

Figure 19: Residual density maps for PT-11 in the plane of phenyl ring, HAR, left: $|F| \geq 2\sigma(|F|)$ and right: all reflections included. Contour level: 0.05 e/Å^3. Colours: blue - positive, red – negative.

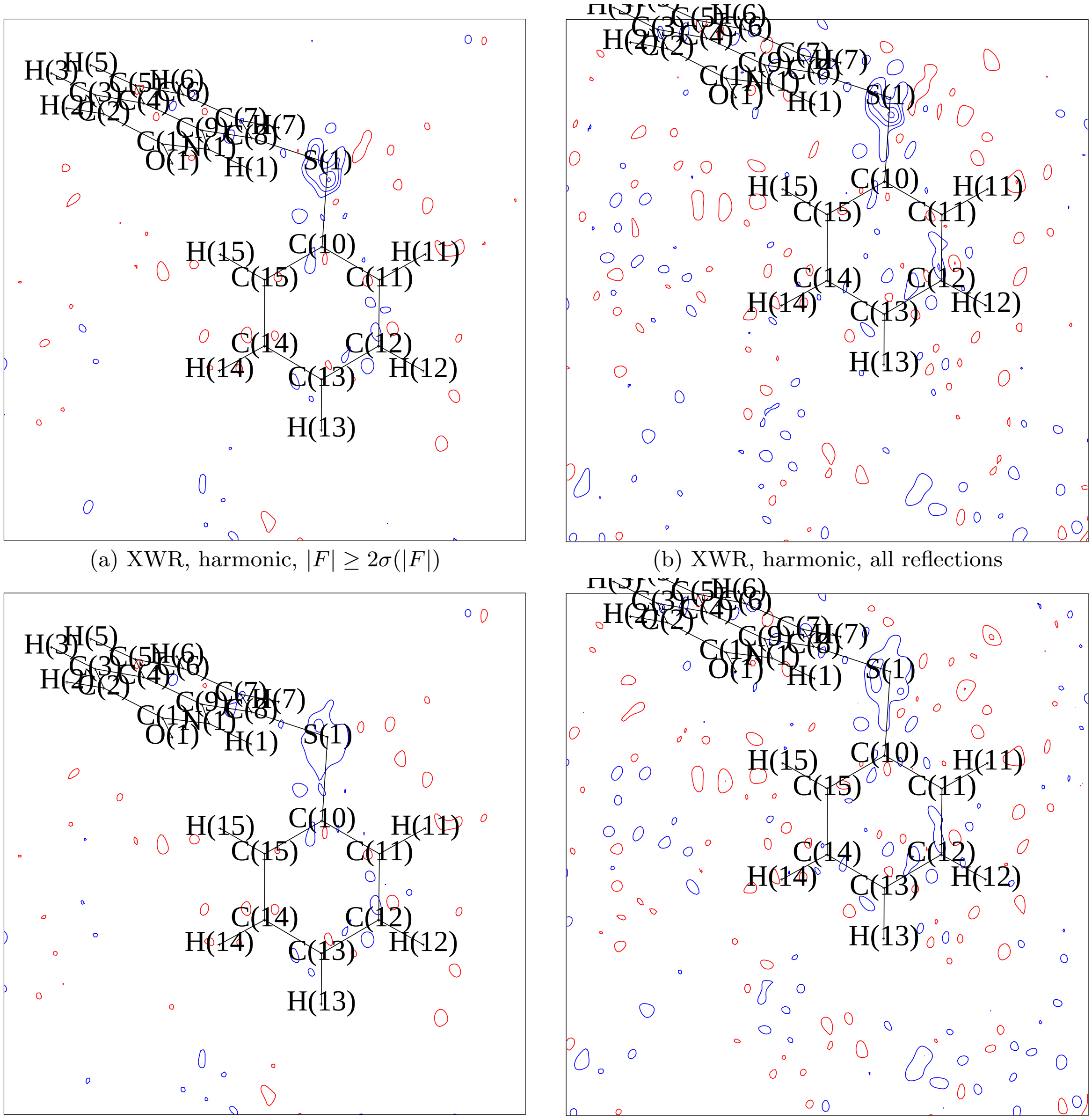

(a) XWR, harmonic, $|F| \geq 2\sigma(|F|)$

(b) XWR, harmonic, all reflections

(c) XWR, anharmonic n=4, $|F| \geq 2\sigma(|F|)$

(d) XWR, anharmonic n=4, all reflections

Figure 20: Residual density maps for PT-11 in the plane of phenyl ring, XWR, left: $|F| \geq 2\sigma(|F|)$ and right: all reflections included. Contour level: 0.05 e/Å^3. Colours: blue - positive, red – negative.

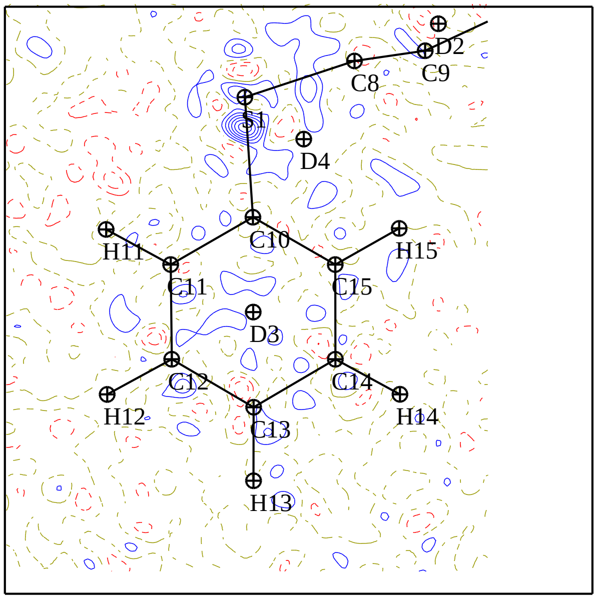

(a) MM, harmonic, $|F| \geq 2\sigma(|F|)$

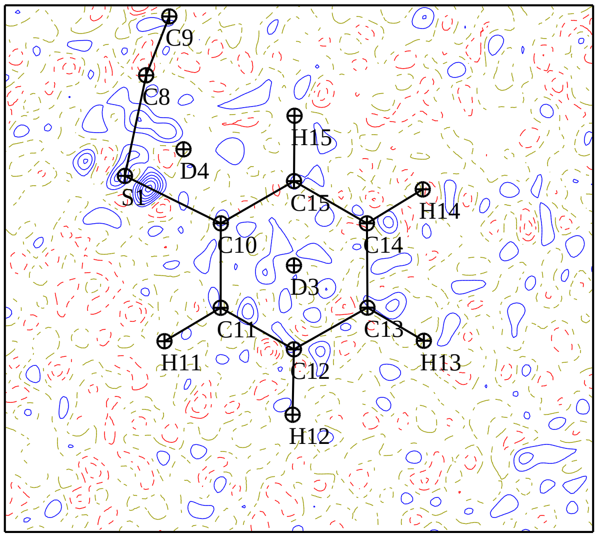

(b) MM, harmonic, all reflections

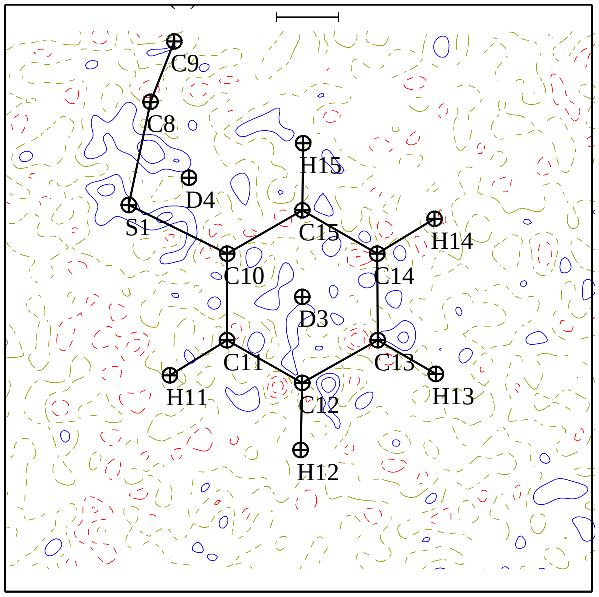

(c) MM, anharmonic n=3, $|F| \geq 2\sigma(|F|)$

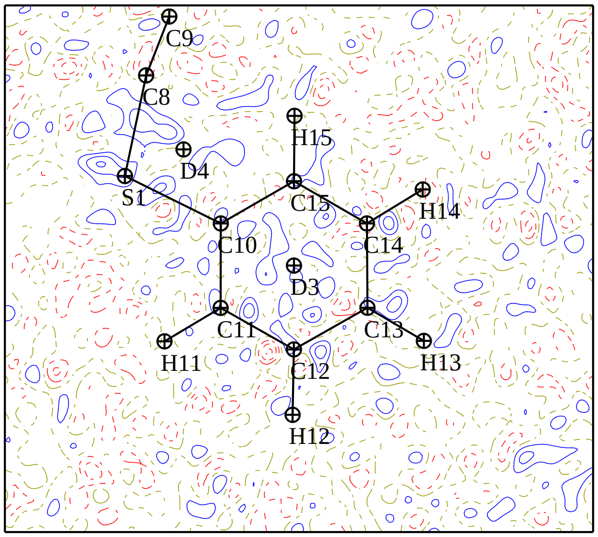

(d) MM, anharmonic n=3, all reflections

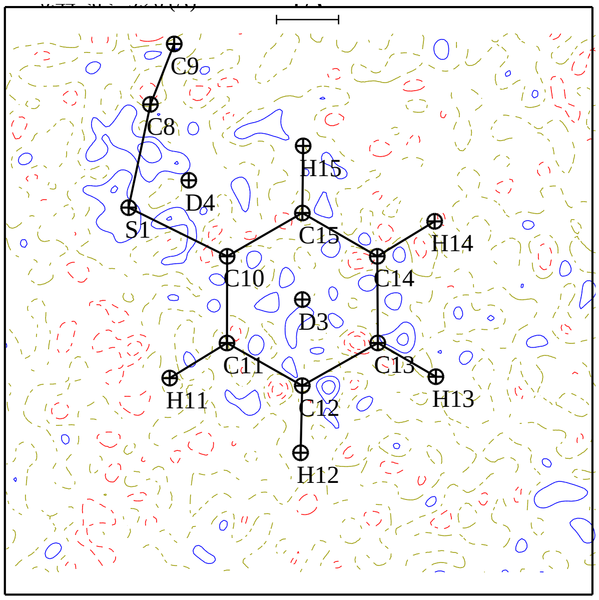

(e) MM, anharmonic n=4, $|F| \geq 2\sigma(|F|)$

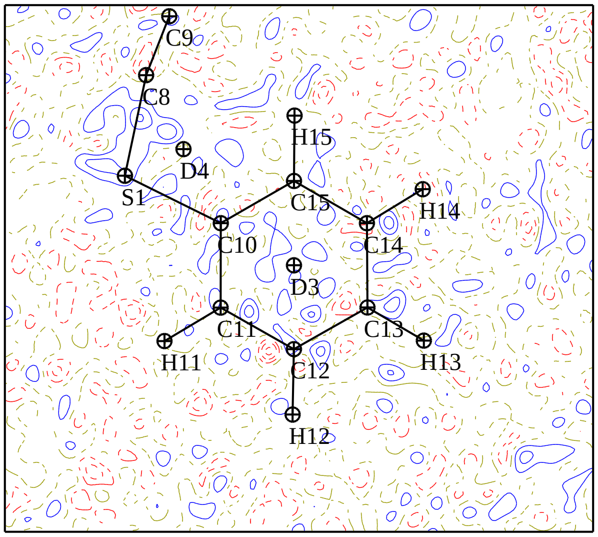

(f) MM, anharmonic n=4, all reflections

Figure 21: Residual density maps for PT-11 in the plane of phenyl ring, MM, restrained X-H distances, left: $|F| \geq 2\sigma(|F|)$ and right: all reflections included. Contour level: 0.05 e/Å^3. Colours: blue - positive, red – negative.

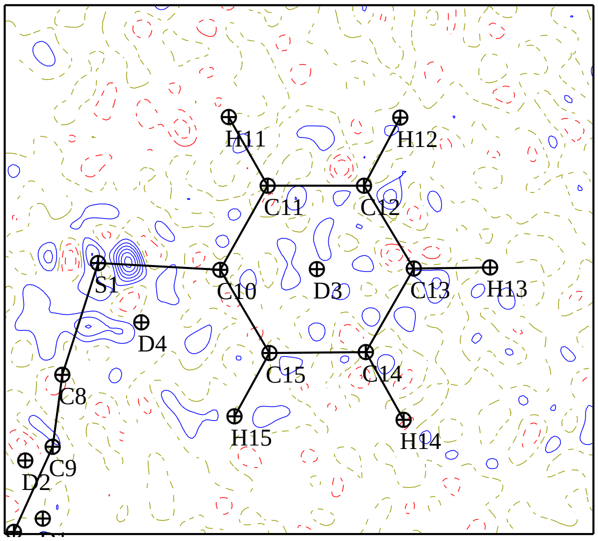

(a) MM, harmonic, $|F| \geq 2\sigma(|F|)$

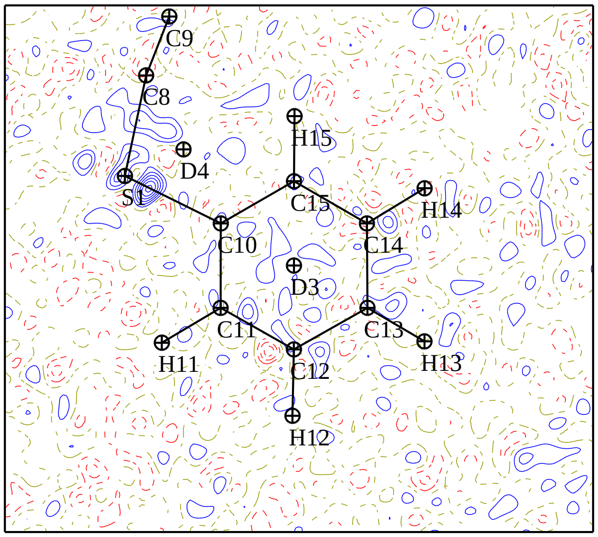

(b) MM, harmonic, all reflections

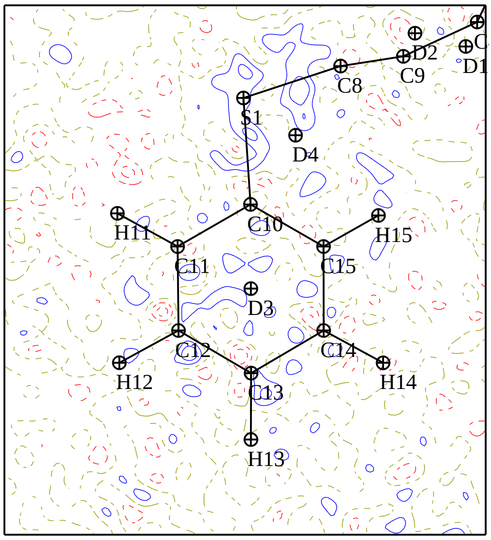

(c) MM, anharmonic n=3, $|F| \geq 2\sigma(|F|)$

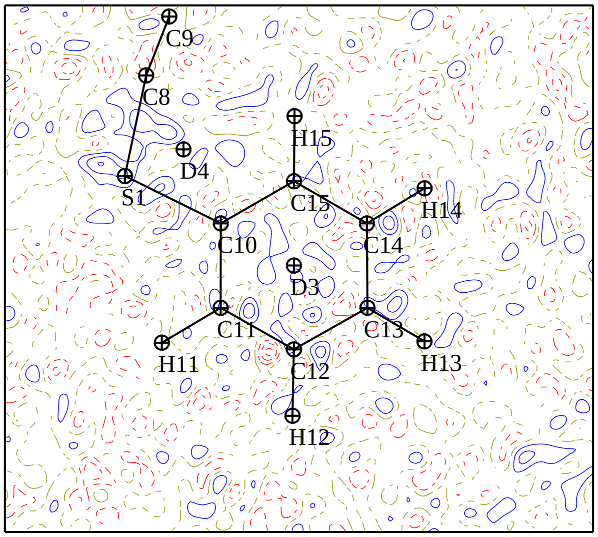

(d) MM, anharmonic n=3, all reflections

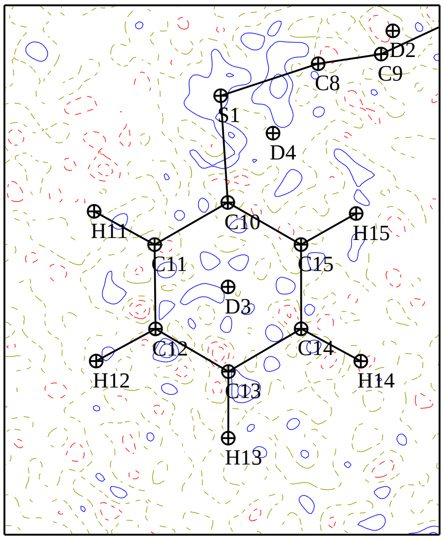

(e) MM, anharmonic n=4, $|F| \geq 2\sigma(|F|)$

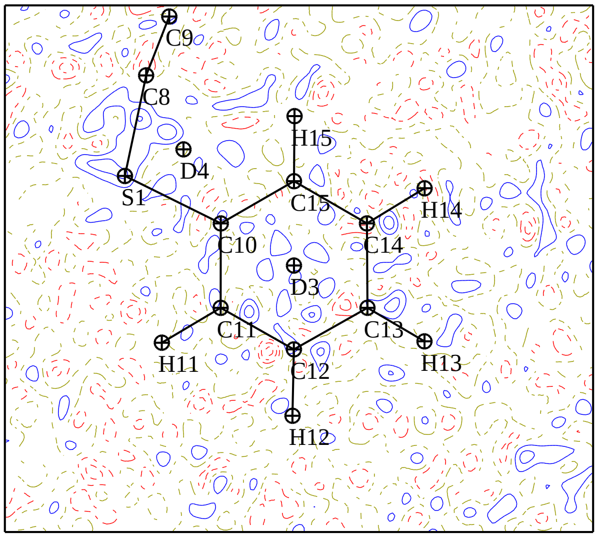

(f) MM, anharmonic n=4, all reflections

Figure 22: Residual density maps for PT-11 in the plane of phenyl ring, MM, unrestrained X-H distances, left: $|F| \geq 2\sigma(|F|)$ and right: all reflections included. Contour level: 0.05 e/Å^3. Colours: blue - positive, red – negative.

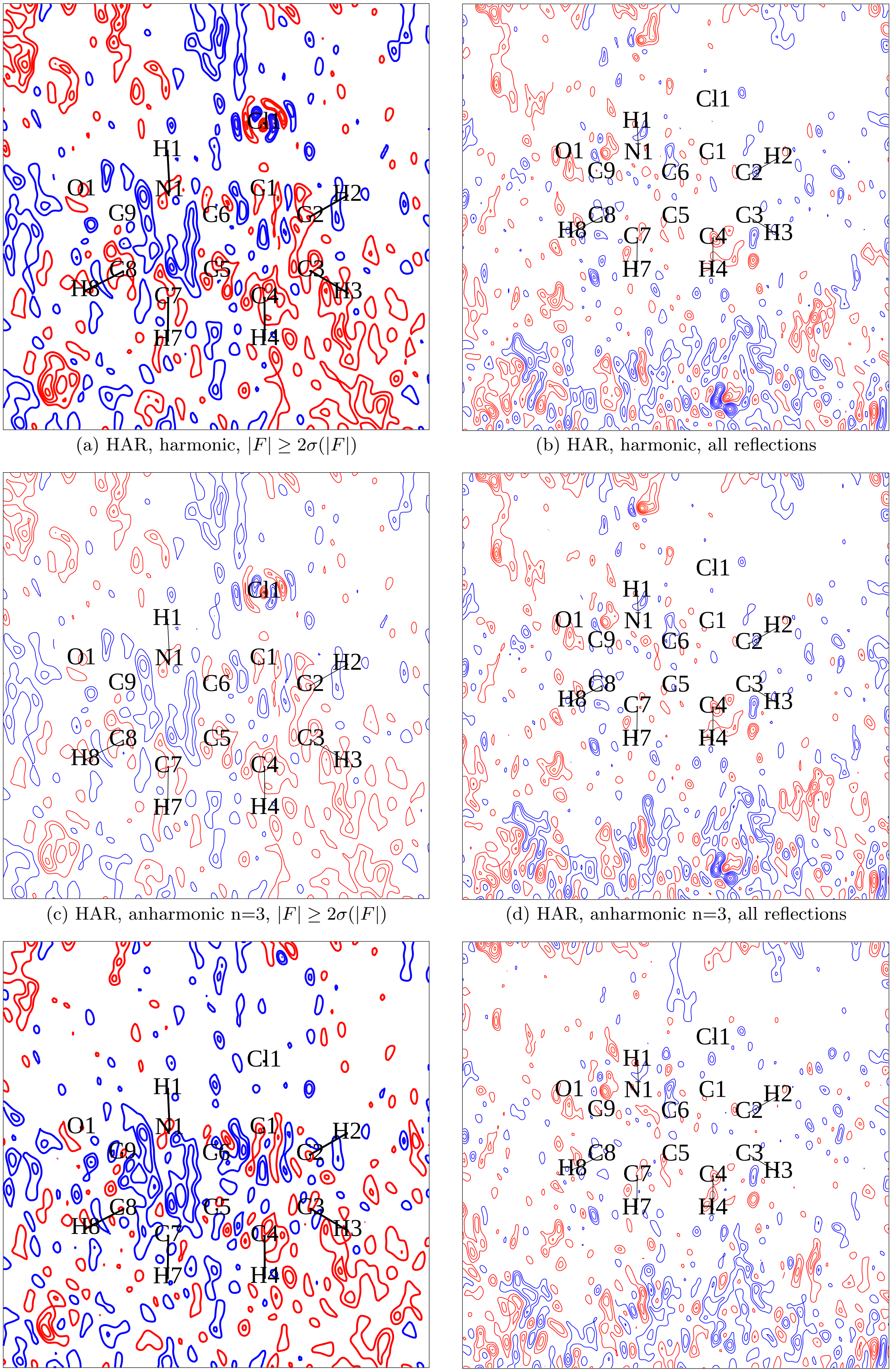

(a) HAR, harmonic, $|F| \geq 2\sigma(|F|)$

(b) HAR, harmonic, all reflections

(c) HAR, anharmonic n=3, $|F| \geq 2\sigma(|F|)$

(d) HAR, anharmonic n=3, all reflections

(e) HAR, anharmonic n=4, $|F| \geq 2\sigma(|F|)$

(f) HAR, anharmonic n=4, all reflections

Figure 23: Residual density maps for PT-2, HAR, left: $|F| \geq 2\sigma(|F|)$ and right: all reflections included. Contour level: 0.05 e/Å^3. Colours: blue - positive, red – negative.

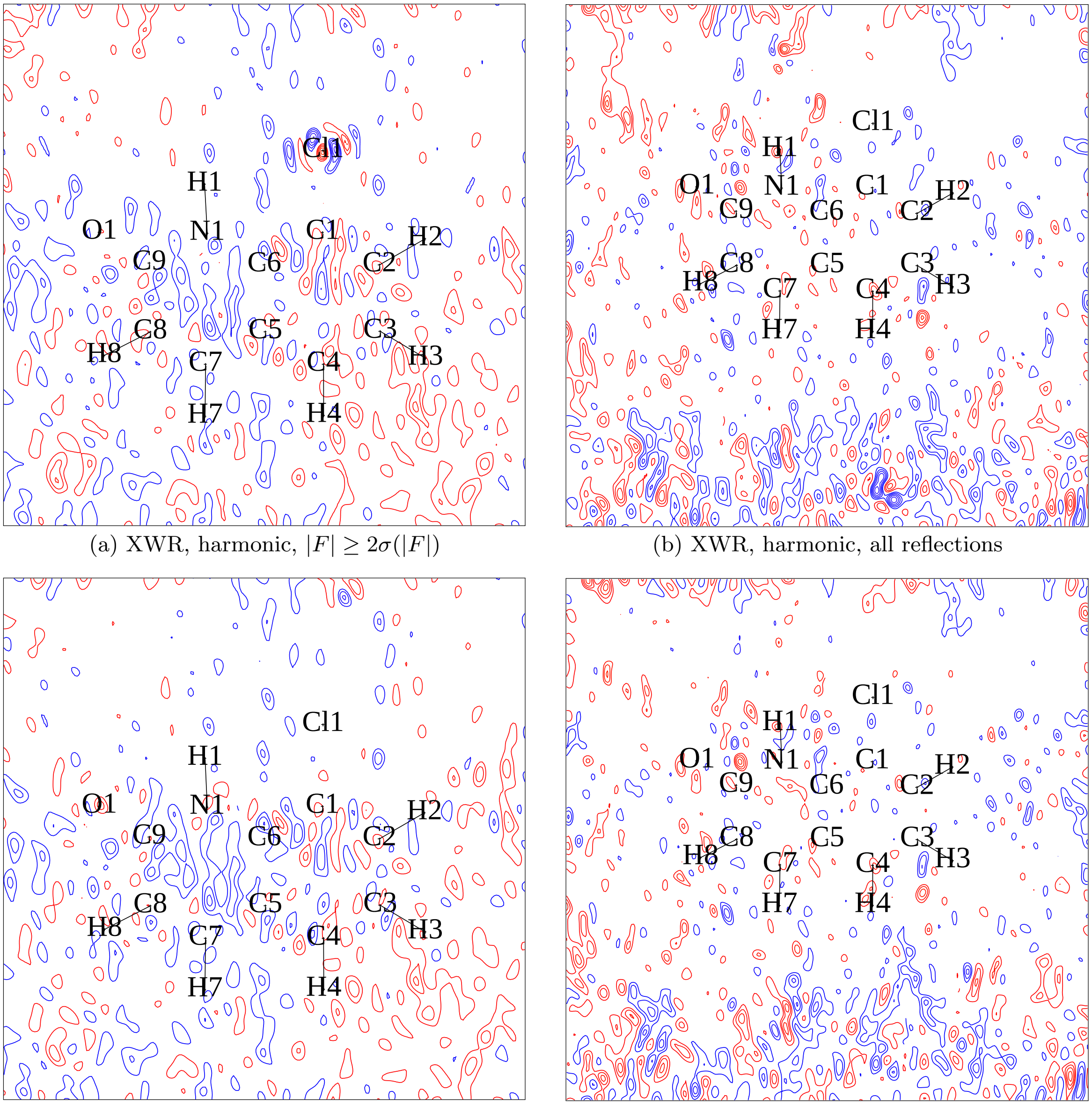

(a) XWR, harmonic, $|F| \geq 2\sigma(|F|)$

(b) XWR, harmonic, all reflections

(c) XWR, anharmonic n=4, $|F| \geq 2\sigma(|F|)$

(d) XWR, anharmonic n=4, all reflections

Figure 24: Residual density maps for PT-2, XWR, left: $|F| \geq 2\sigma(|F|)$ and right: all reflections included. Contour level: 0.05 e/Å^3. Colours: blue - positive, red – negative.

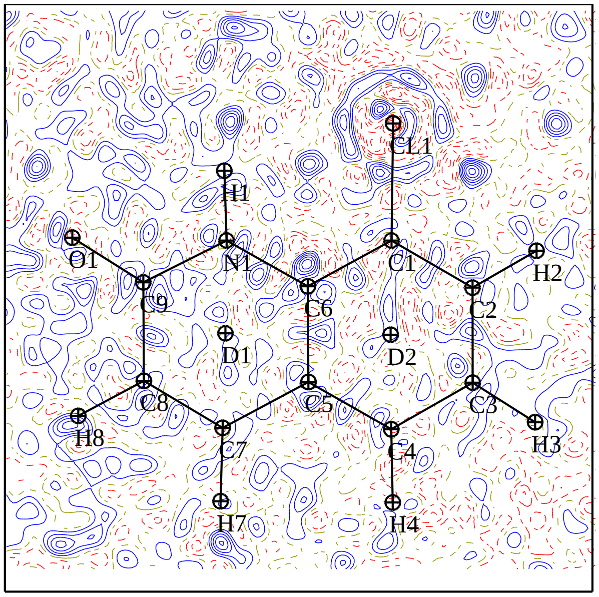

(a) MM, harmonic, $|F| \geq 2\sigma(|F|)$

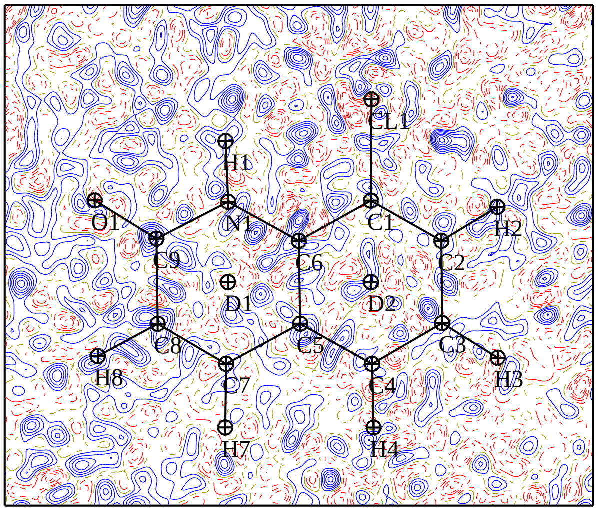

(b) MM, harmonic, all reflections

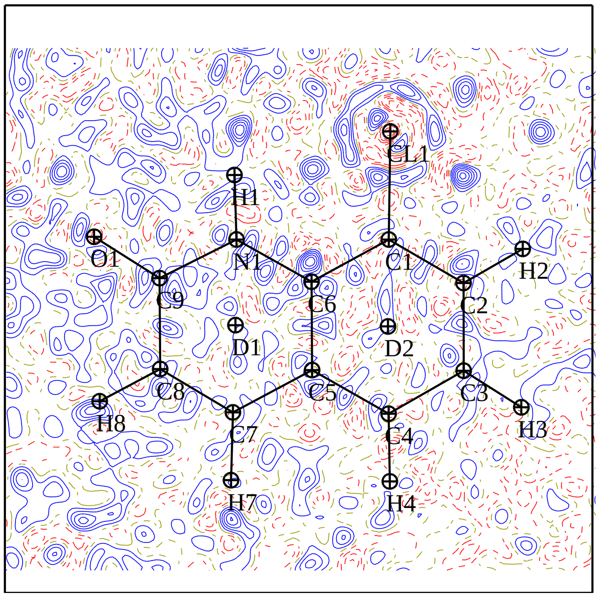

(c) MM, anharmonic n=3, $|F| \geq 2\sigma(|F|)$

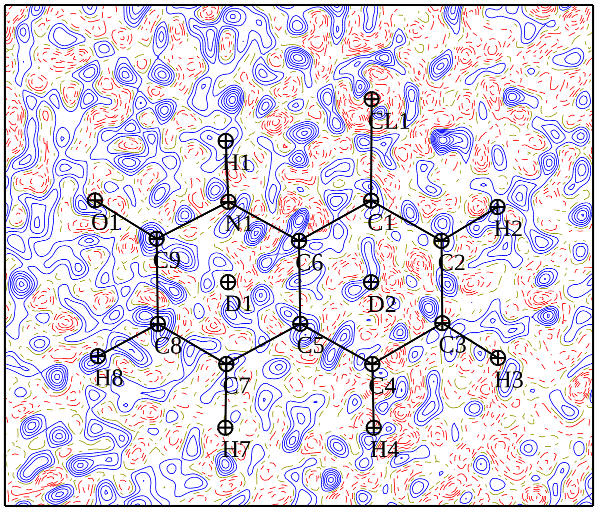

(d) MM, anharmonic n=3, all reflections

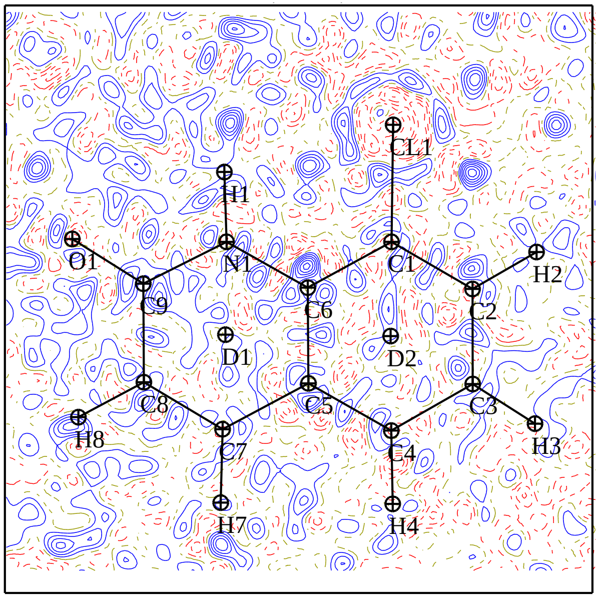

(e) MM, anharmonic n=4, $|F| \geq 2\sigma(|F|)$

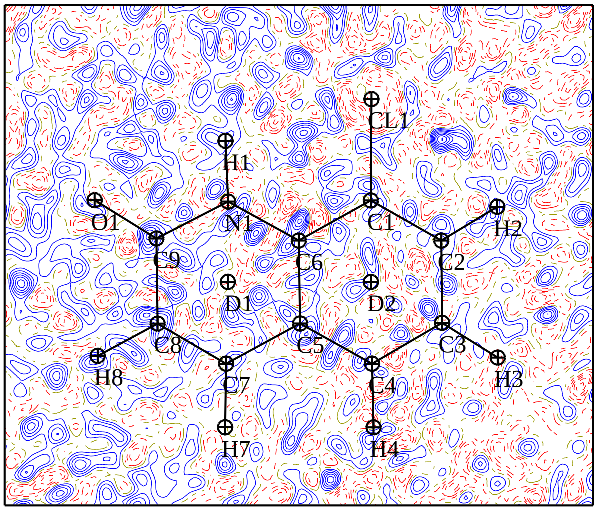

(f) MM, anharmonic n=4, all reflections

Figure 25: Residual density maps for PT-2, MM, restrained X-H distances, left: $|F| \geq 2\sigma(|F|)$ and right: all reflections included. Contour level: 0.05 e/Å^3. Colours: blue - positive, red – negative.

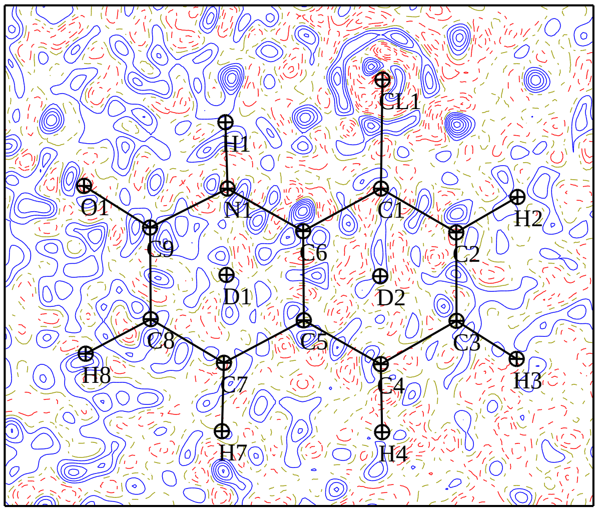

(a) MM, harmonic, $|F| \geq 2\sigma(|F|)$

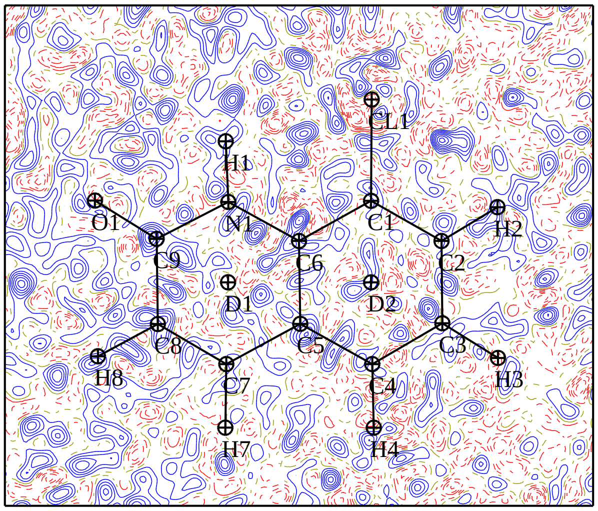

(b) MM, harmonic, all reflections

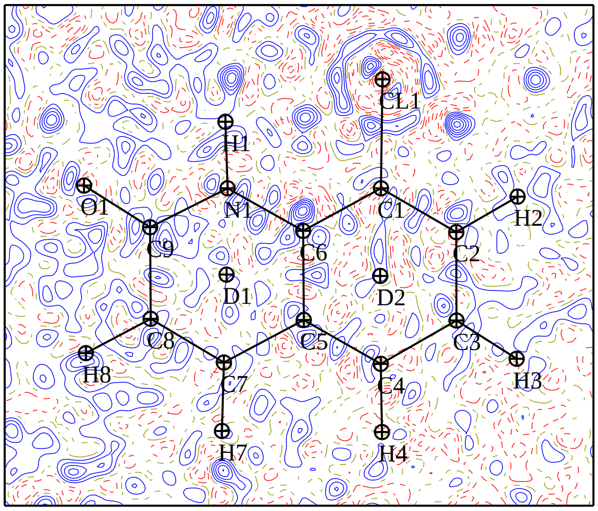

(c) MM, anharmonic n=3, $|F| \geq 2\sigma(|F|)$

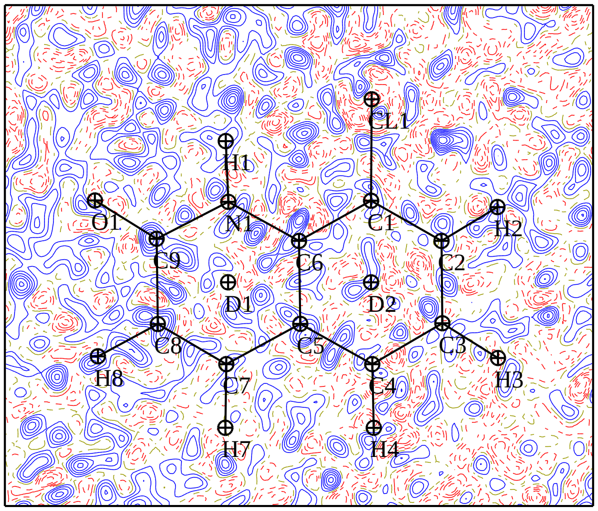

(d) MM, anharmonic n=3, all reflections

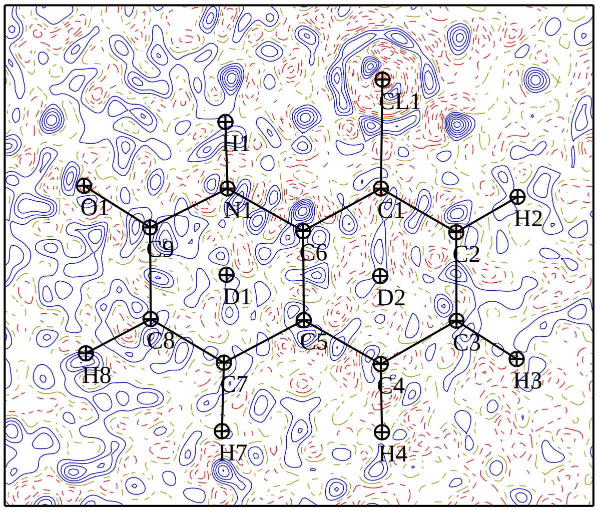

(e) MM, anharmonic n=4, $|F| \geq 2\sigma(|F|)$

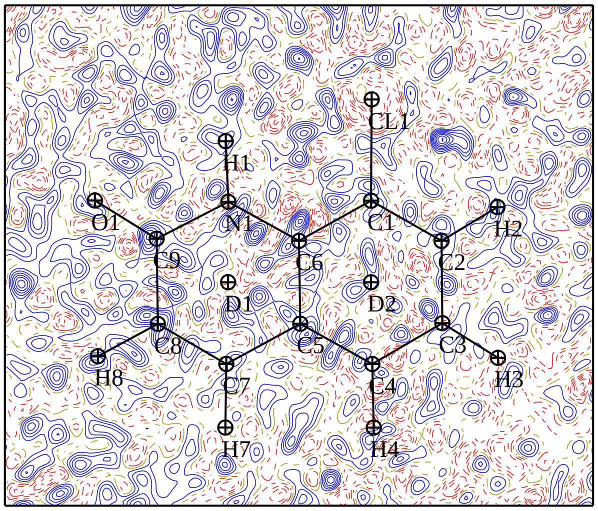

(f) MM, anharmonic n=4, all reflections

Figure 26: Residual density maps for PT-2, MM, unrestrained X-H distances, left: $|F| \geq 2\sigma(|F|)$ and right: all reflections included. Contour level: 0.05 e/Å^3. Colours: blue - positive, red – negative.

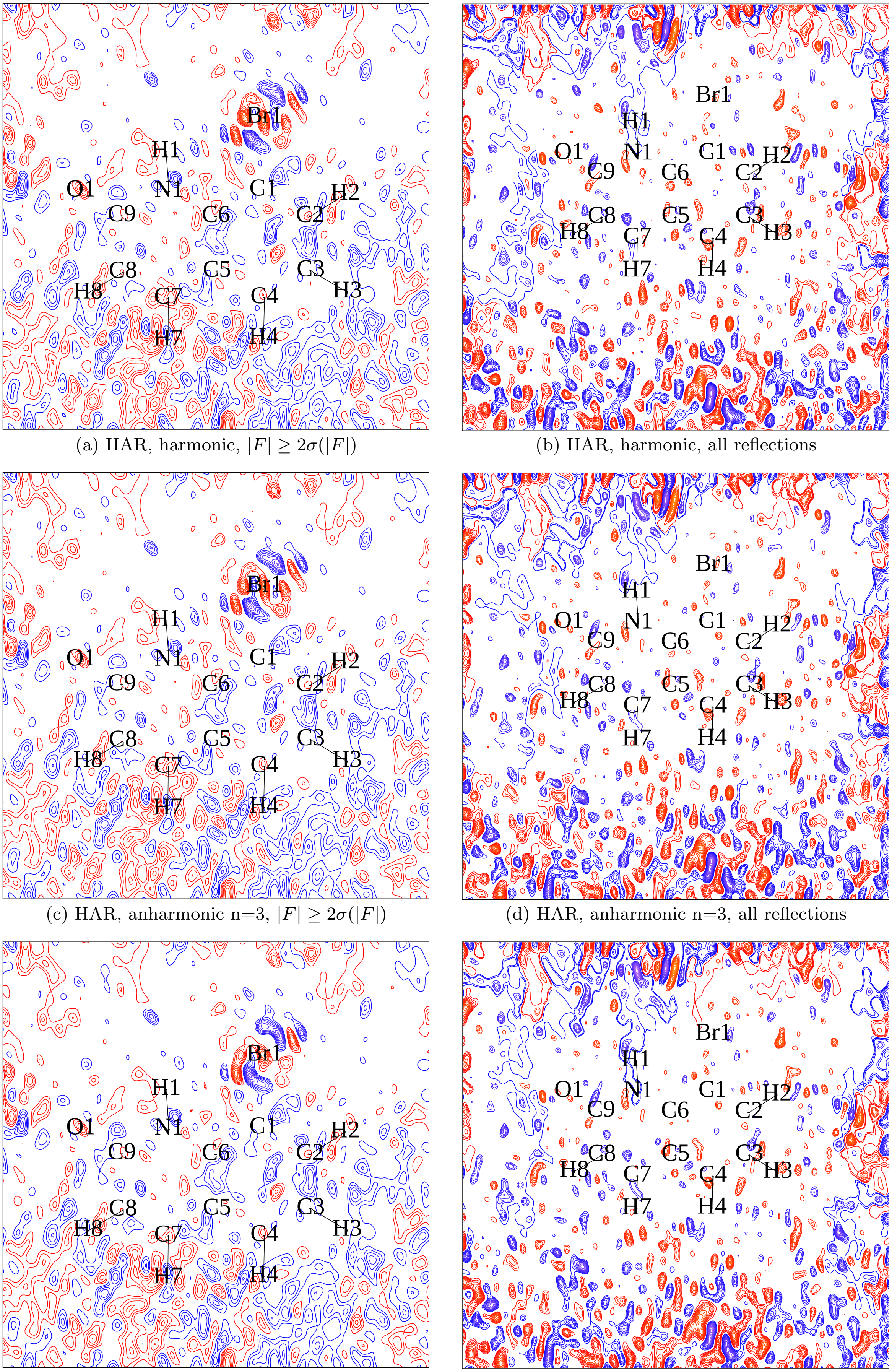

(a) HAR, harmonic, $|F| \geq 2\sigma(|F|)$

(b) HAR, harmonic, all reflections

(c) HAR, anharmonic n=3, $|F| \geq 2\sigma(|F|)$

(d) HAR, anharmonic n=3, all reflections

(e) HAR, anharmonic n=4, $|F| \geq 2\sigma(|F|)$

(f) HAR, anharmonic n=4, all reflections

Figure 27: Residual density maps for PT-8, HAR, left: $|F| \geq 2\sigma(|F|)$ and right: all reflections included. Contour level: 0.05 e/Å^3. Colours: blue - positive, red – negative.

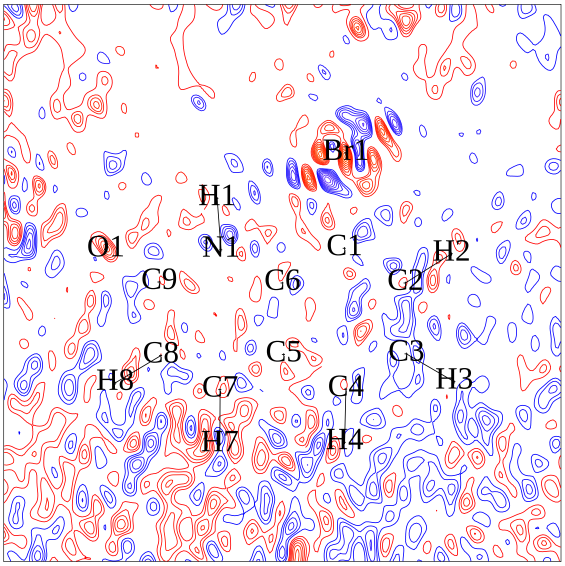

(a) XWR, harmonic, $|F| \geq 2\sigma(|F|)$

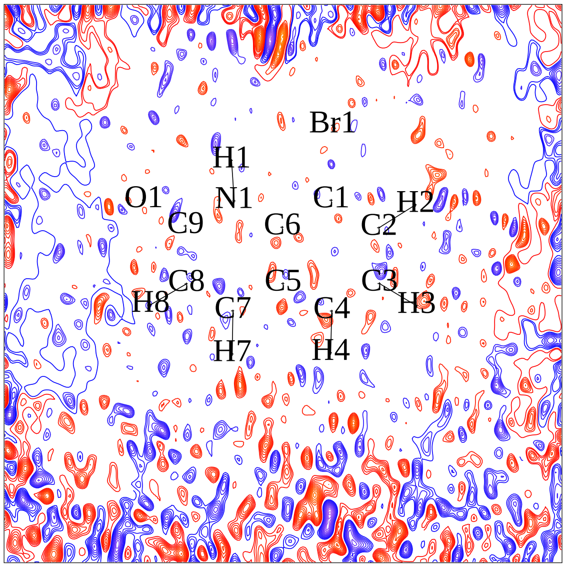

(b) XWR, harmonic, all reflections

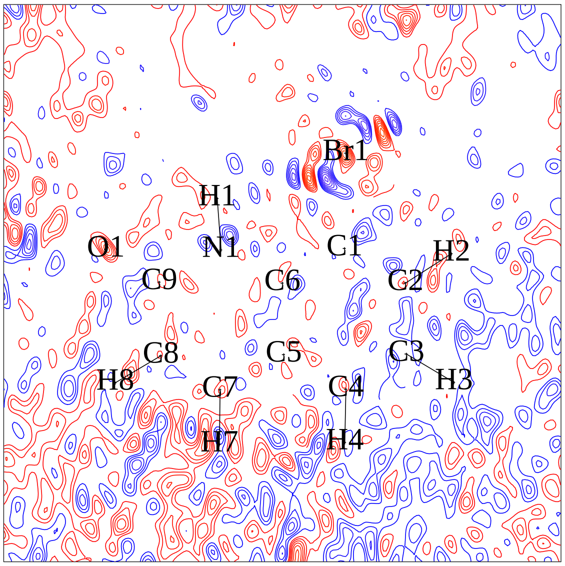

(c) XWR, anharmonic n=4, $|F| \geq 2\sigma(|F|)$

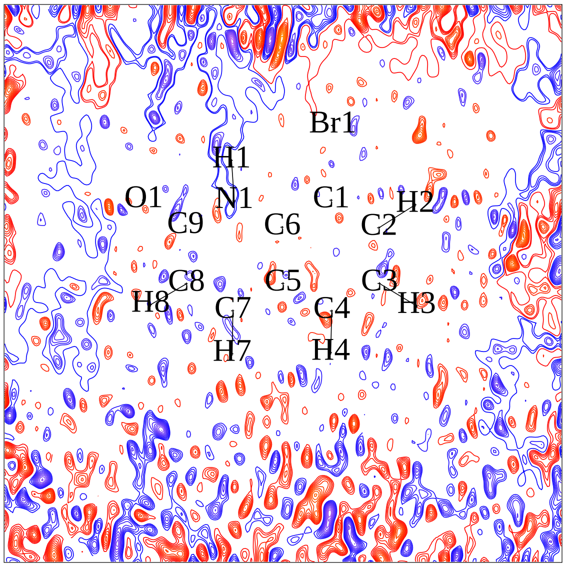

(d) XWR, anharmonic n=4, all reflections

Figure 28: Residual density maps for PT-8, XWR, left: $|F| \geq 2\sigma(|F|)$ and right: all reflections included. Contour level: 0.05 e/Å^3. Colours: blue - positive, red – negative.

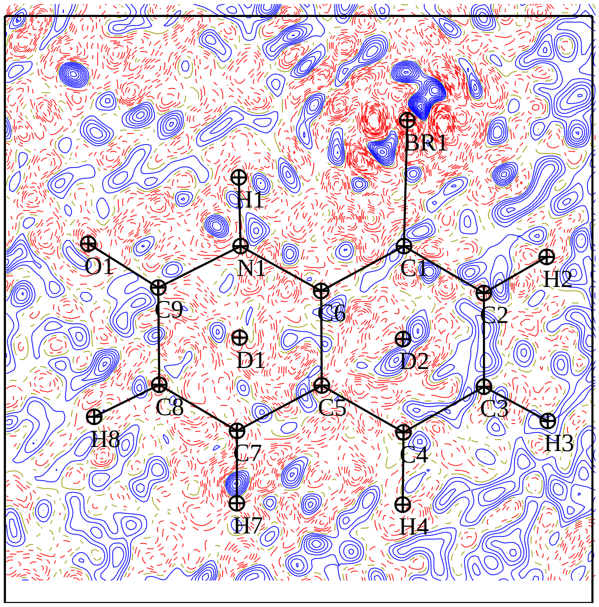

(a) MM, harmonic, $|F| \geq 2\sigma(|F|)$

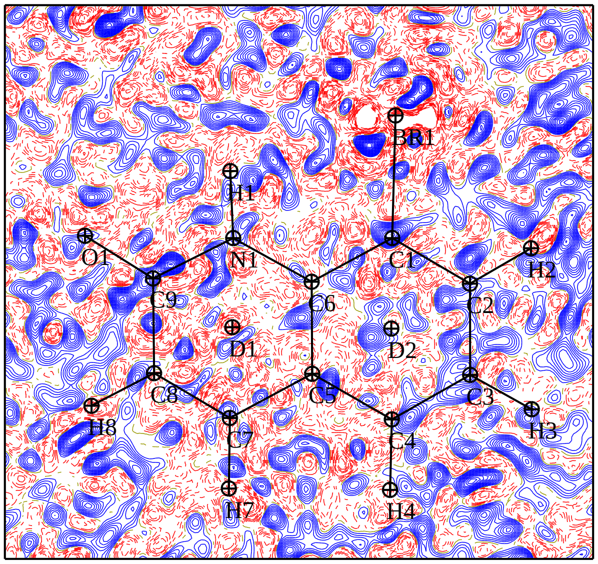

(b) MM, harmonic, all reflections

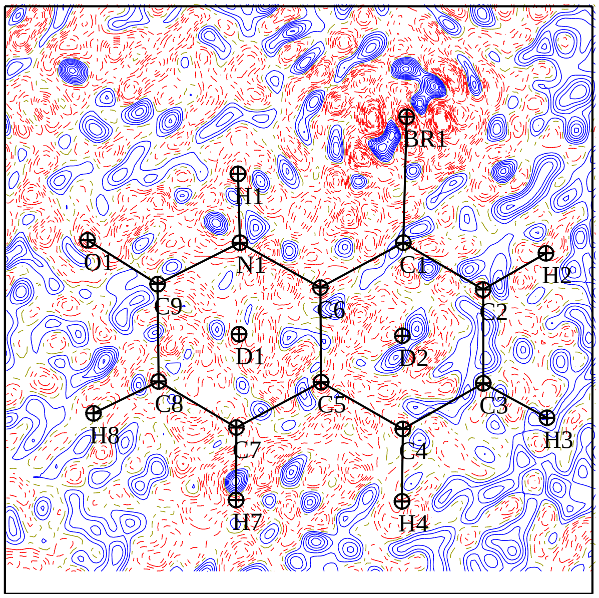

(c) MM, anharmonic n=3, $|F| \geq 2\sigma(|F|)$

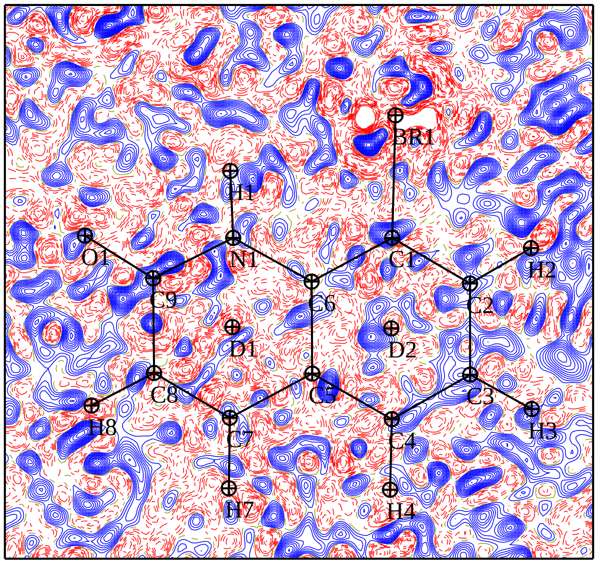

(d) MM, anharmonic n=3, all reflections

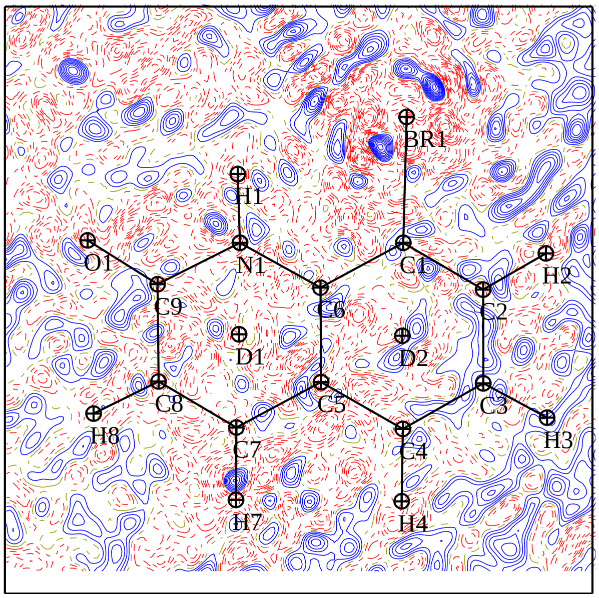

(e) MM, anharmonic n=4, $|F| \geq 2\sigma(|F|)$

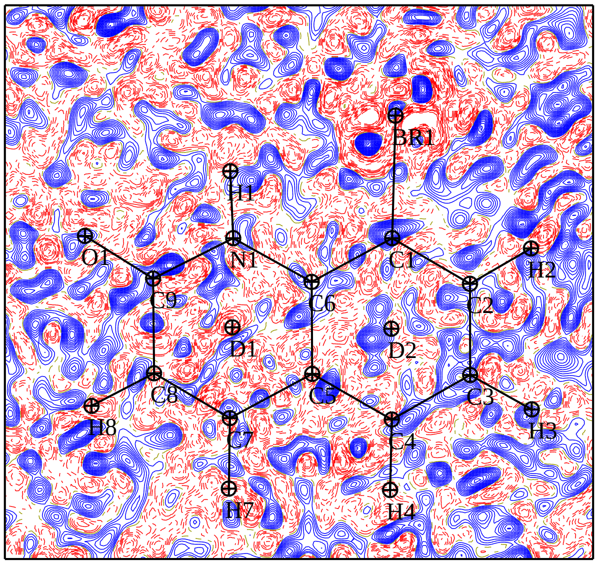

(f) MM, anharmonic n=4, all reflections

Figure 29: Residual density maps for PT-8, MM, restrained X-H distances, left: $|F| \geq 2\sigma(|F|)$ and right: all reflections included. Contour level: 0.05 e/Å^3. Colours: blue - positive, red – negative.

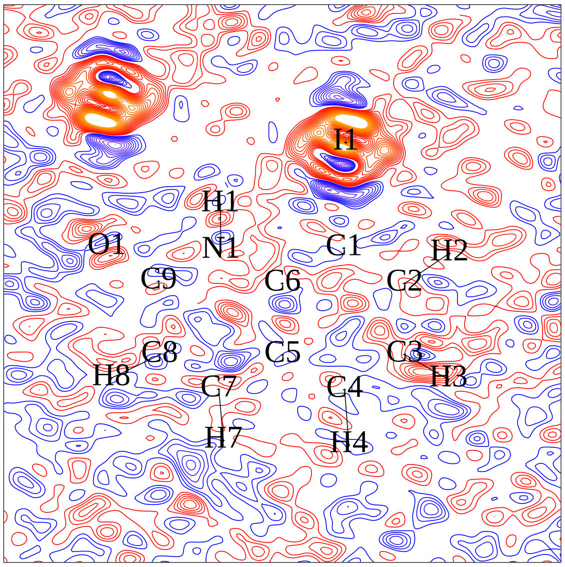

(a) HAR, harmonic, $|F| \geq 2\sigma(|F|)$

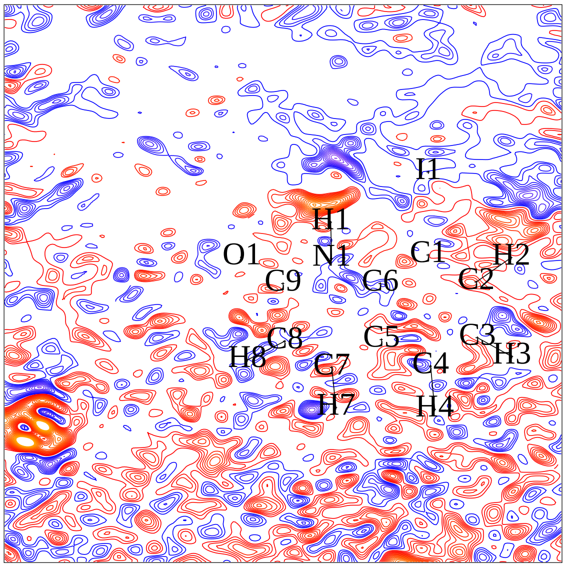

(b) HAR, harmonic, all reflections

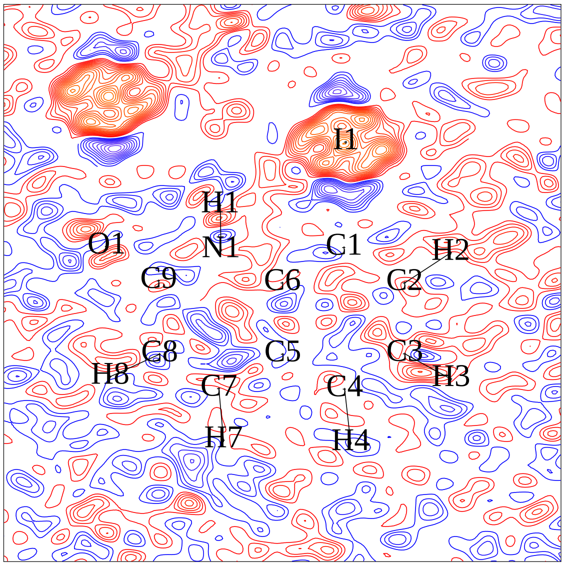

(c) HAR, anharmonic n=3, $|F| \geq 2\sigma(|F|)$

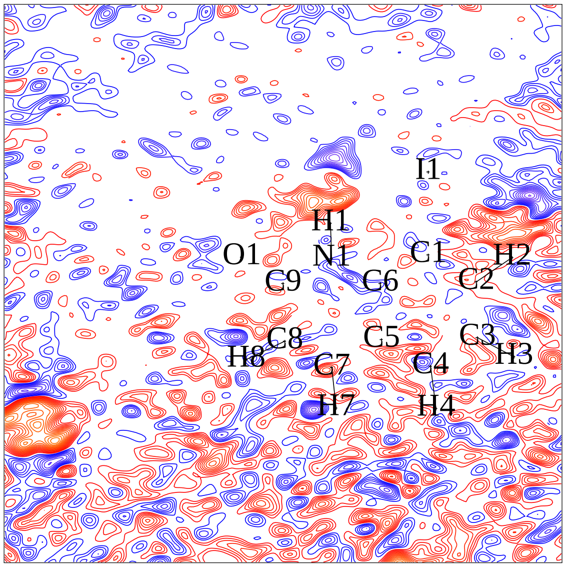

(d) HAR, anharmonic n=3, all reflections

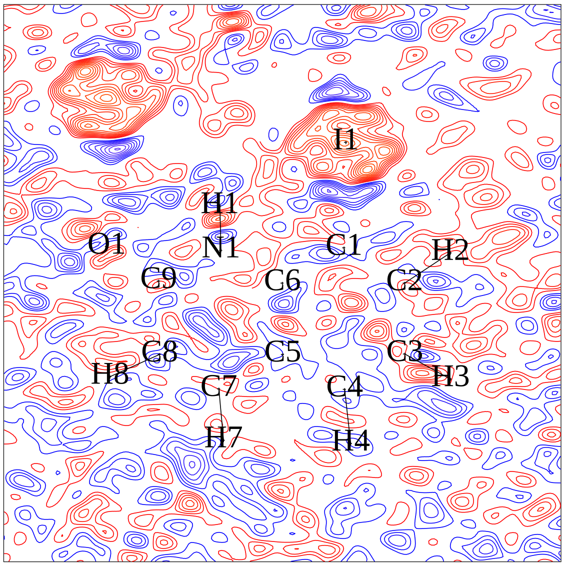

(e) HAR, anharmonic n=4, $|F| \geq 2\sigma(|F|)$

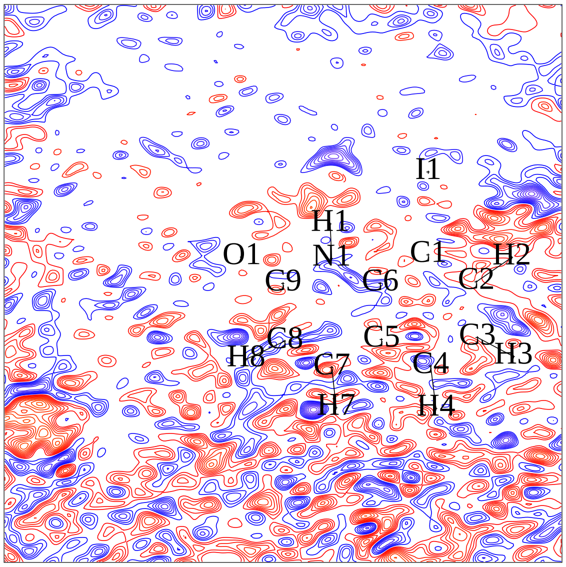

(f) HAR, anharmonic n=4, all reflections

Figure 30: Residual density maps for PT-10, HAR, left: $|F| \geq 2\sigma(|F|)$ and right: all reflections included. Contour level: 0.05 e/Å^3. Colours: blue - positive, red – negative.

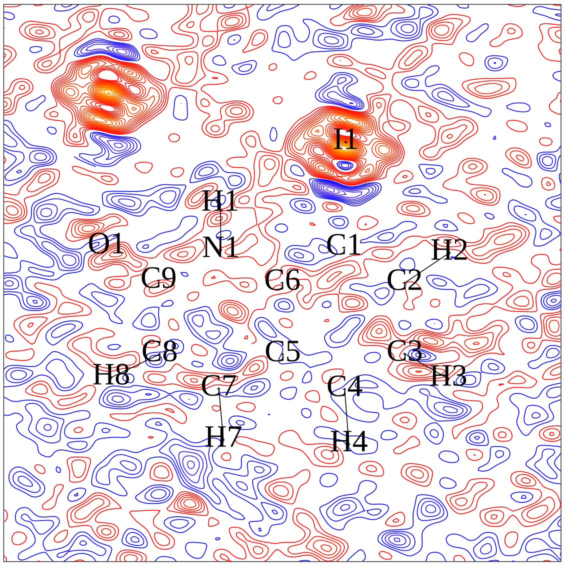

(a) XWR, harmonic, $|F| \geq 2\sigma(|F|)$

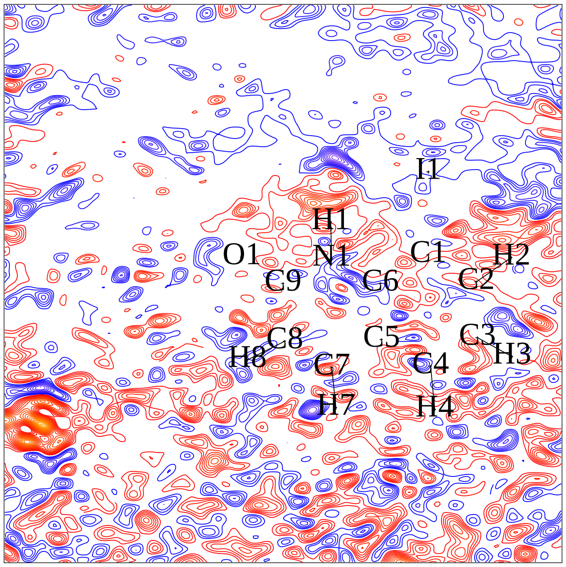

(b) XWR, harmonic, all reflections

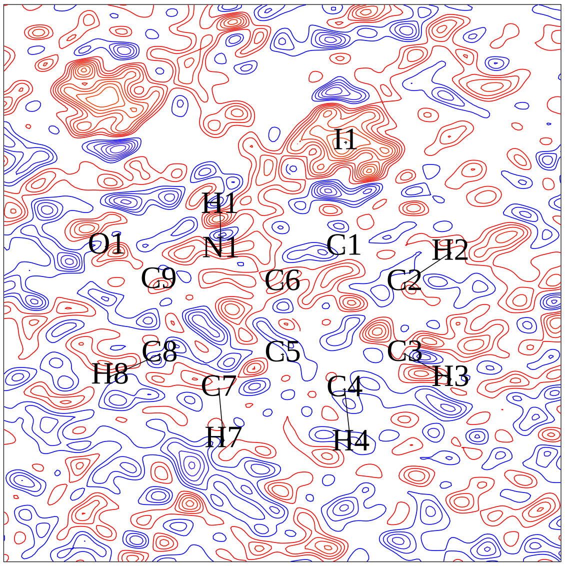

(c) XWR, anharmonic n=4, $|F| \geq 2\sigma(|F|)$

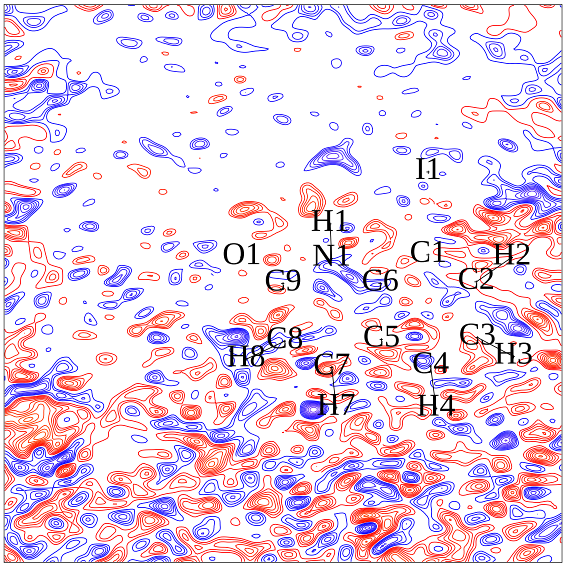

(d) XWR, anharmonic n=4, all reflections

Figure 31: Residual density maps for PT-10, XWR, left: $|F| \geq 2\sigma(|F|)$ and right: all reflections included. Contour level: 0.05 e/Å^3. Colours: blue - positive, red – negative.

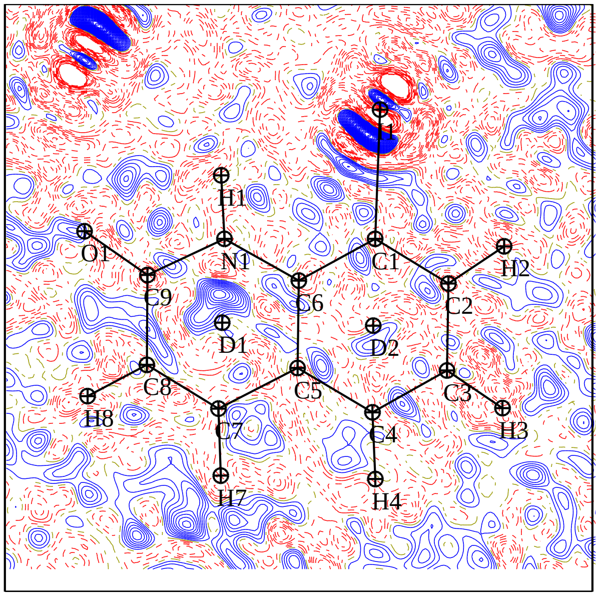

(a) MM, harmonic, $|F| \geq 2\sigma(|F|)$

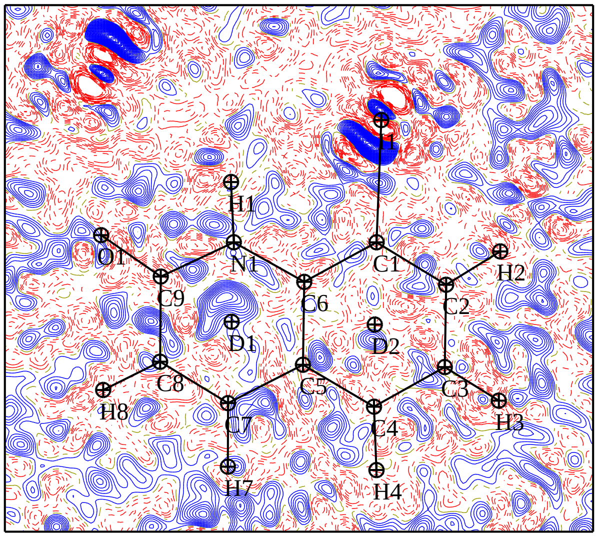

(b) MM, harmonic, all reflections

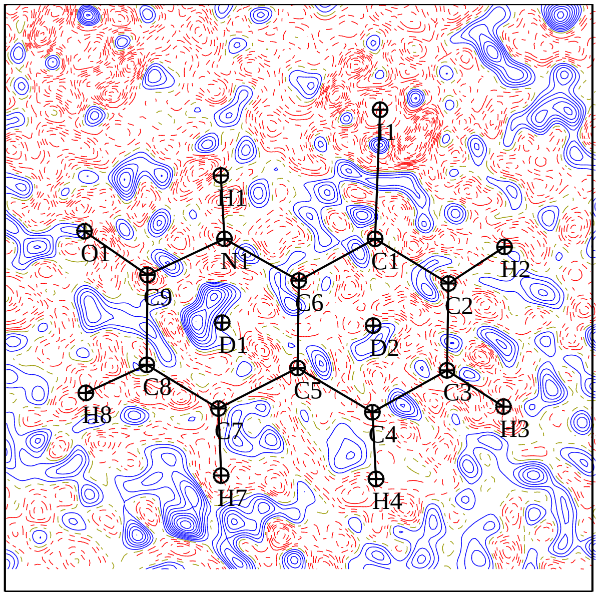

(c) MM, anharmonic n=3, $|F| \geq 2\sigma(|F|)$

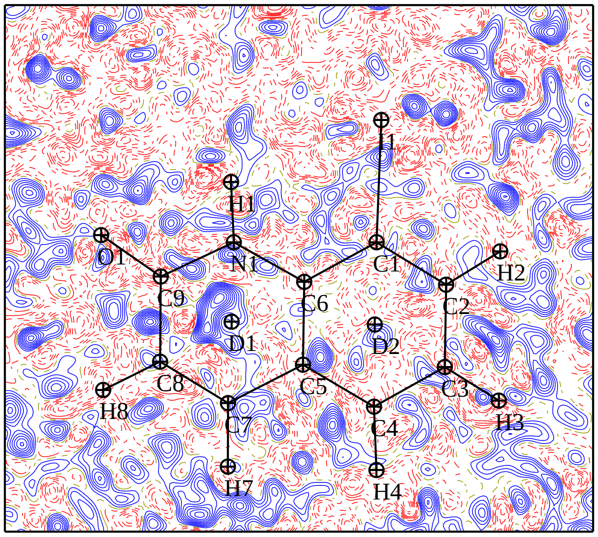

(d) MM, anharmonic n=3, all reflections

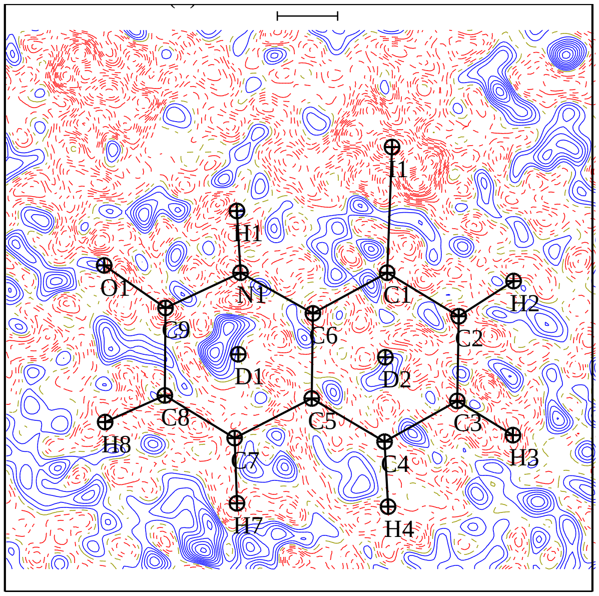

(e) MM, anharmonic n=4, $|F| \geq 2\sigma(|F|)$

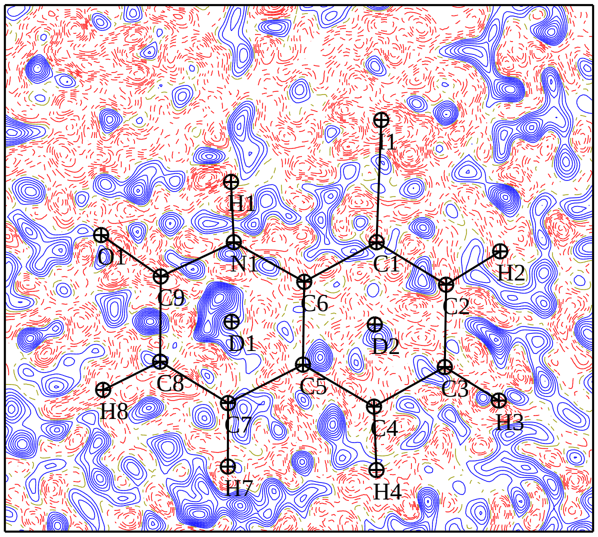

(f) MM, anharmonic n=4, all reflections

Figure 32: Residual density maps for PT-10, MM, restrained X-H distances, left: $|F| \geq 2\sigma(|F|)$ and right: all reflections included. Contour level: 0.05 e/Å^3. Colours: blue - positive, red – negative.

5 Deformation density maps

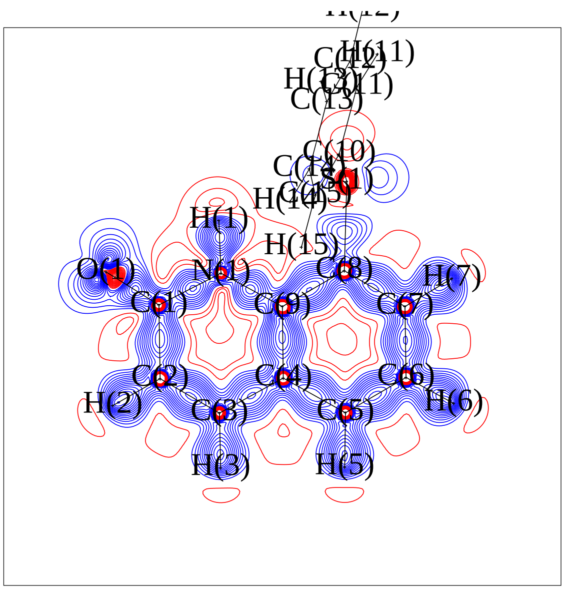

(a) HAR, harmonic, $|F| \geq 2\sigma(|F|)$

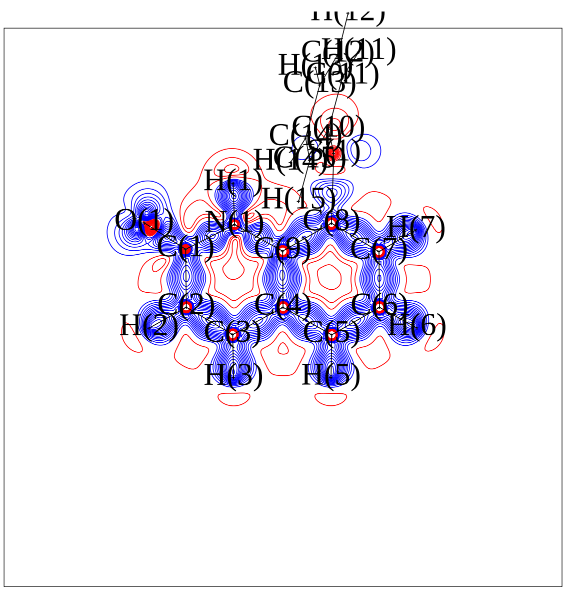

(b) HAR, harmonic, all reflections

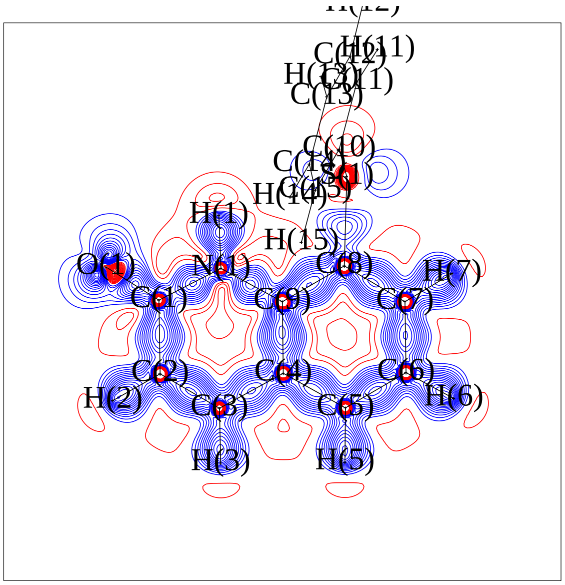

(c) HAR, anharmonic n=3, $|F| \geq 2\sigma(|F|)$

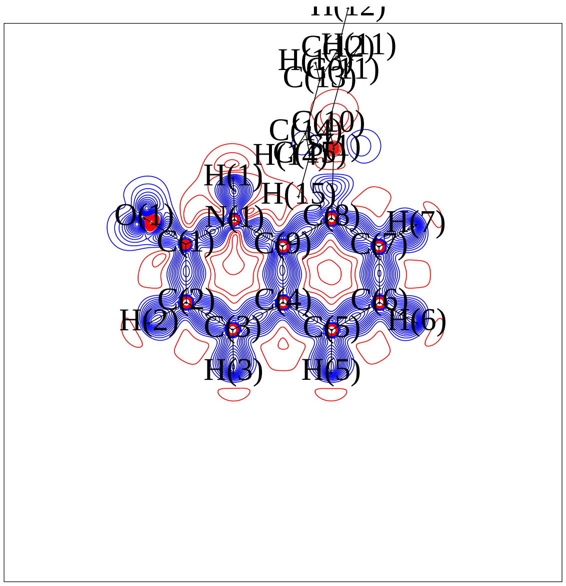

(d) HAR, anharmonic n=3, all reflections

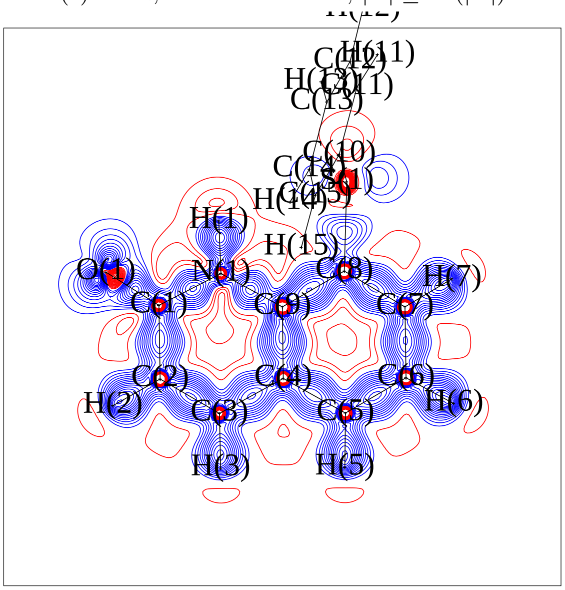

(e) HAR, anharmonic n=4, $|F| \geq 2\sigma(|F|)$

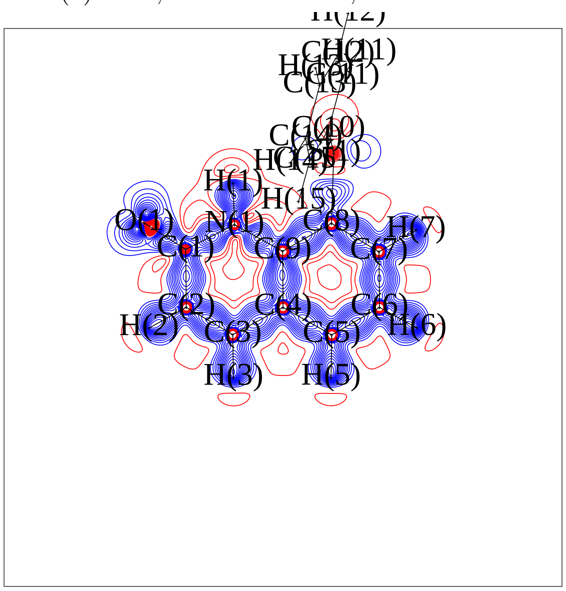

(f) HAR, anharmonic n=4, all reflections

Figure 33: Deformation density maps for PT-11 in the plane of quinoline, HAR, left: $|F| \geq 2\sigma(|F|)$ and right: all reflections included. Contour level: 0.05 e/Å^3. Colours: blue - positive, red – negative.

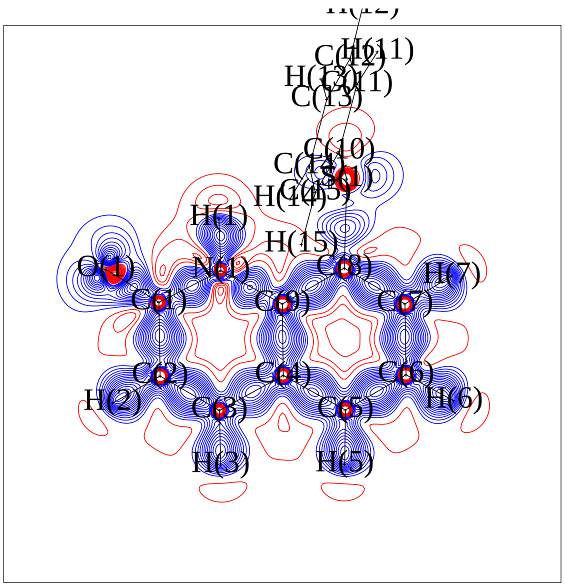

(a) XWR, harmonic, $|F| \geq 2\sigma(|F|)$

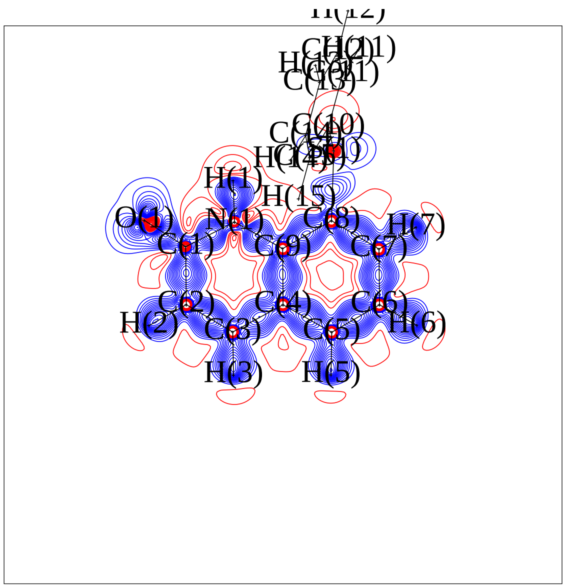

(b) XWR, harmonic, all reflections

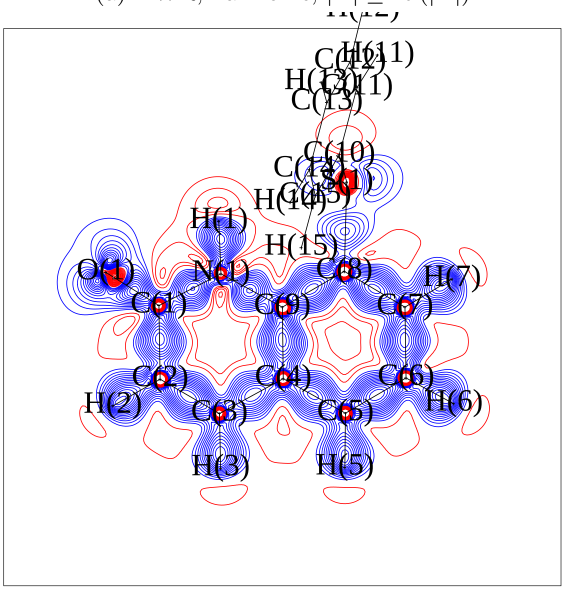

(c) XWR, anharmonic n=4, $|F| \geq 2\sigma(|F|)$

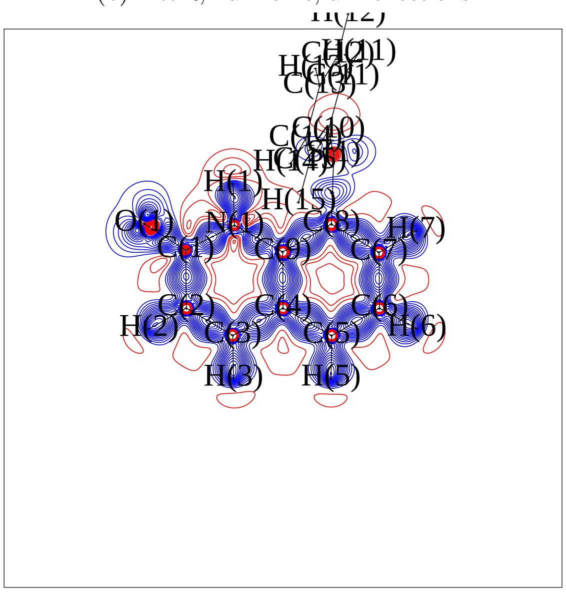

(d) XWR, anharmonic n=4, all reflections

Figure 34: Deformation density maps for PT-11 in the plane of quinoline, XWR, left: $|F| \geq 2\sigma(|F|)$ and right: all reflections included. Contour level: 0.05 e/Å^3. Colours: blue - positive, red – negative.

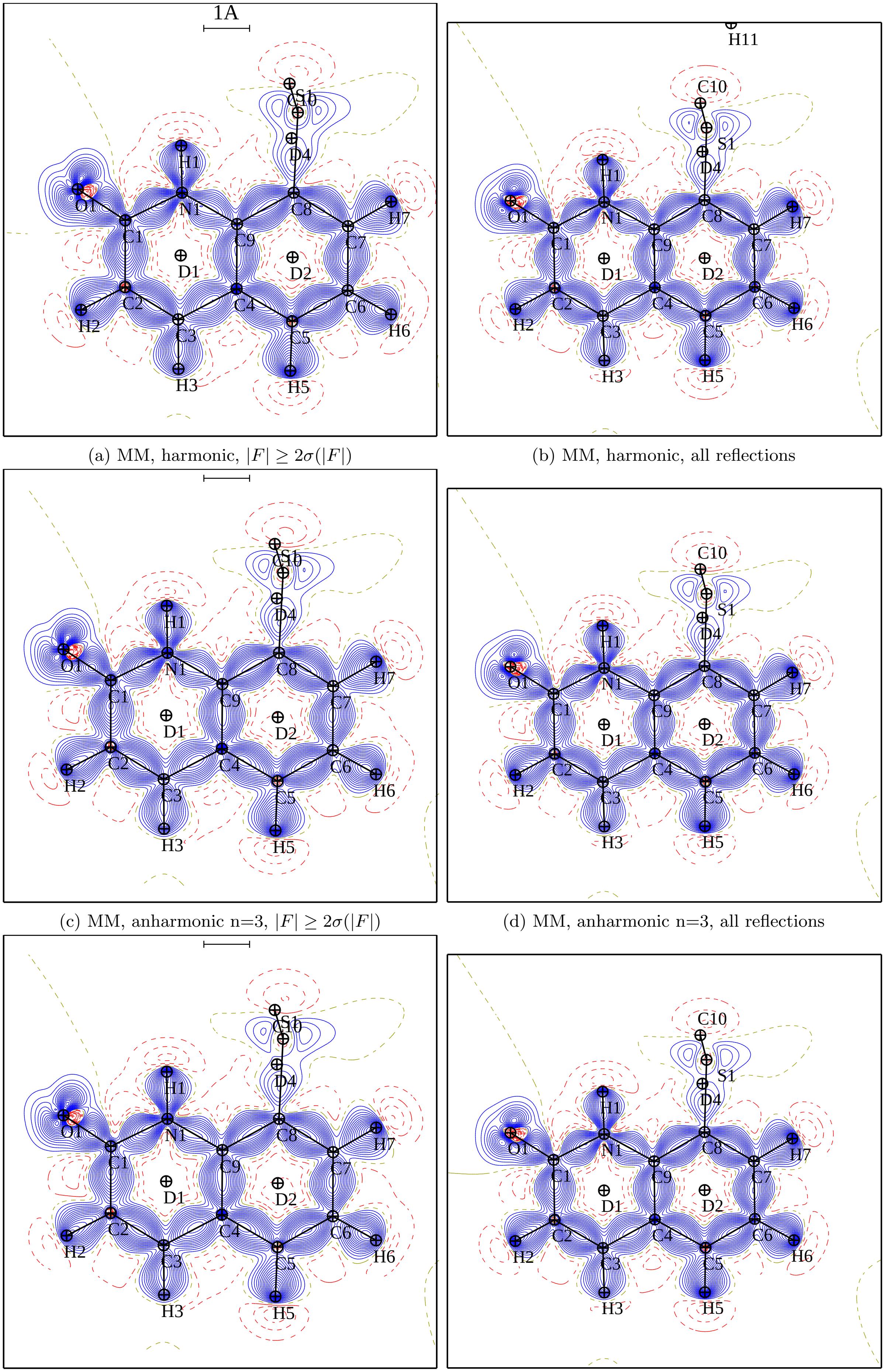

(a) MM, harmonic, $|F| \geq 2\sigma(|F|)$

(b) MM, harmonic, all reflections

(c) MM, anharmonic n=3, $|F| \geq 2\sigma(|F|)$

(d) MM, anharmonic n=3, all reflections

(e) MM, anharmonic n=4, $|F| \geq 2\sigma(|F|)$

(f) MM, anharmonic n=4, all reflections

Figure 35: Deformation density maps for PT-11 in the plane of quinoline, MM, restrained X-H distances, left: $|F| \geq 2\sigma(|F|)$ and right: all reflections included. Contour level: 0.05 e/Å^3. Colours: blue - positive, red – negative.

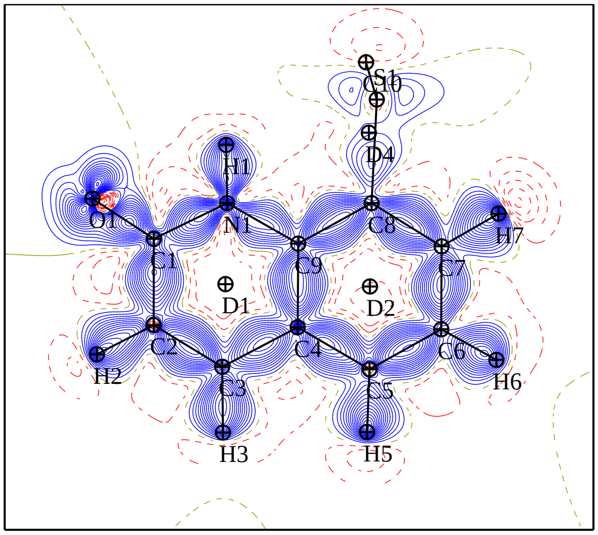

(a) MM, harmonic, $|F| \geq 2\sigma(|F|)$

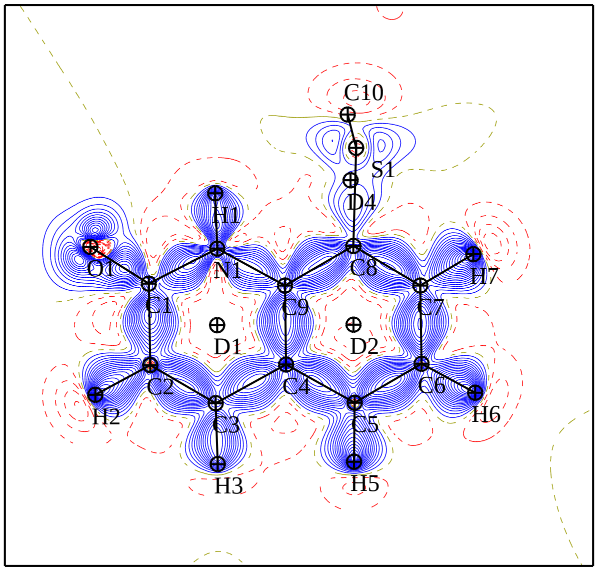

(b) MM, harmonic, all reflections

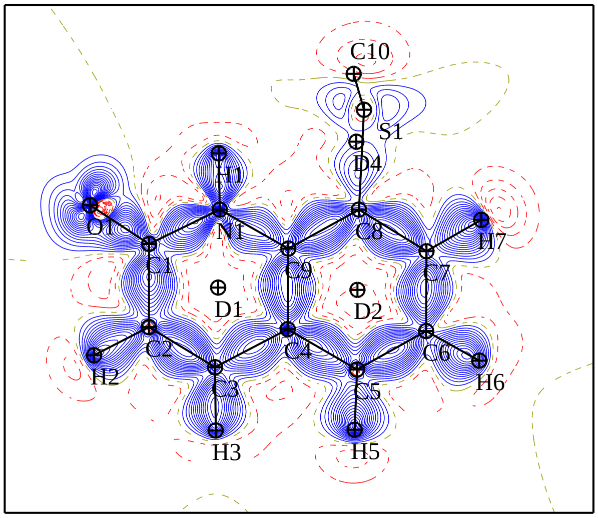

(c) MM, anharmonic n=3, $|F| \geq 2\sigma(|F|)$

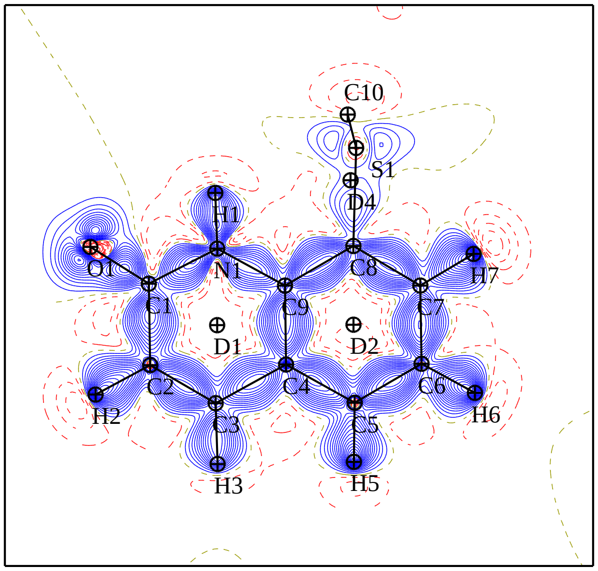

(d) MM, anharmonic n=3, all reflections

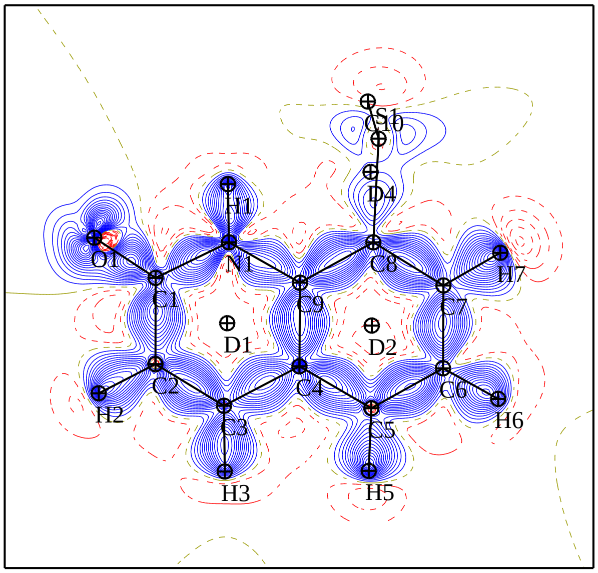

(e) MM, anharmonic n=4, $|F| \geq 2\sigma(|F|)$

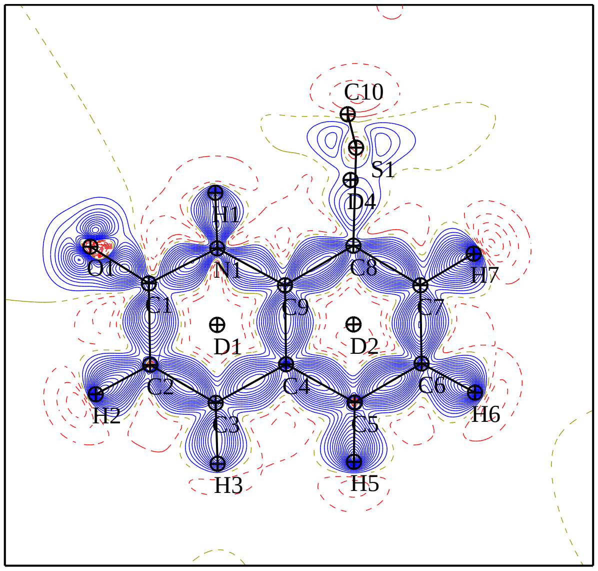

(f) MM, anharmonic n=4, all reflections

Figure 36: Deformation density maps for PT-11 in the plane of quinoline, MM, unrestrained X-H distances, left: $|F| \geq 2\sigma(|F|)$ and right: all reflections included. Contour level: 0.05 e/Å^3. Colours: blue - positive, red – negative.

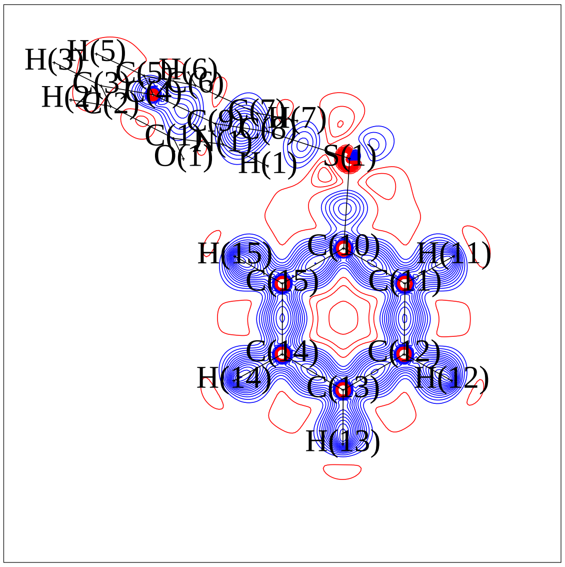

(a) HAR, harmonic, $|F| \geq 2\sigma(|F|)$

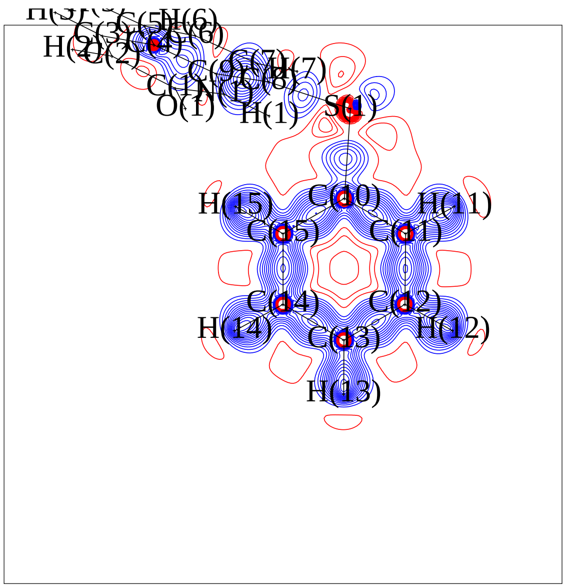

(b) HAR, harmonic, all reflections

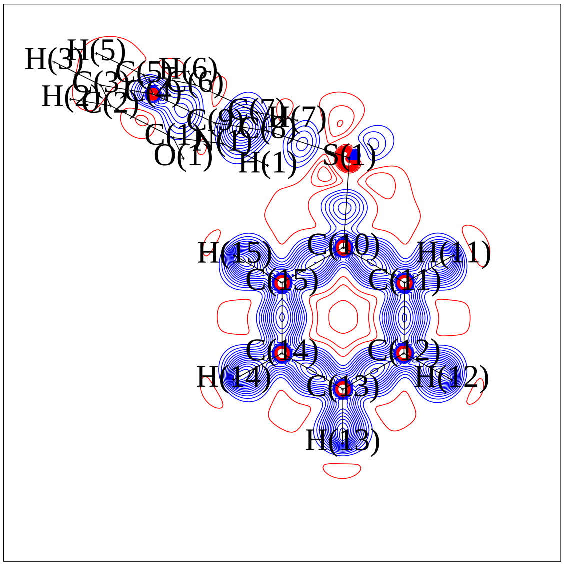

(c) HAR, anharmonic n=3, $|F| \geq 2\sigma(|F|)$

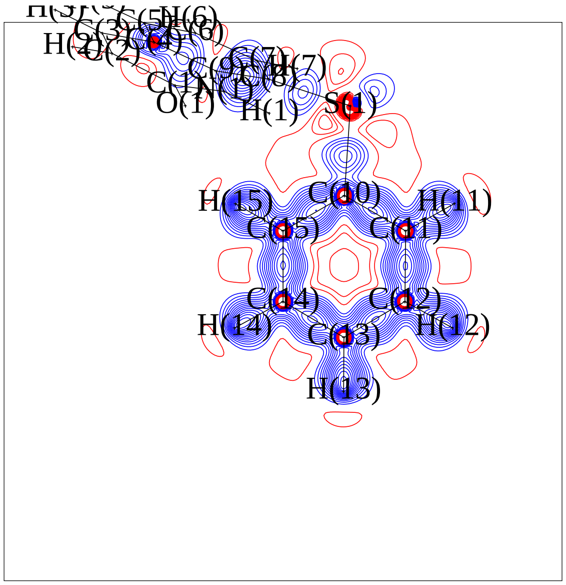

(d) HAR, anharmonic n=3, all reflections

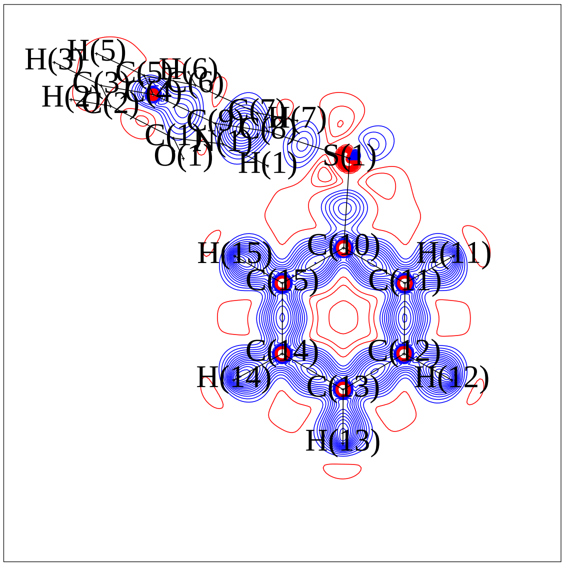

(e) HAR, anharmonic n=4, $|F| \geq 2\sigma(|F|)$

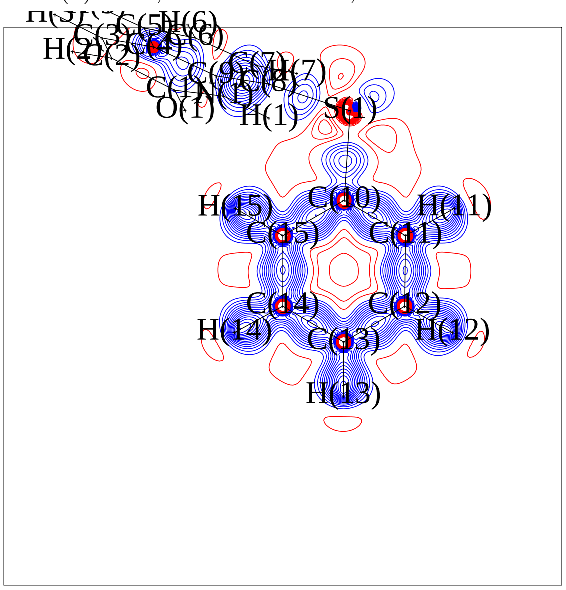

(f) HAR, anharmonic n=4, all reflections

Figure 37: Deformation density maps for PT-11 in the plane of phenyl ring, HAR, left: $|F| \geq 2\sigma(|F|)$ and right: all reflections included. Contour level: 0.05 e/Å^3. Colours: blue - positive, red – negative.

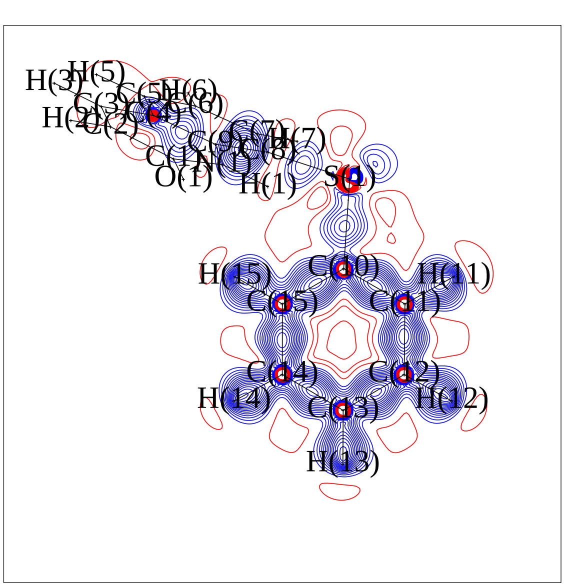

(a) XWR, harmonic, $|F| \geq 2\sigma(|F|)$

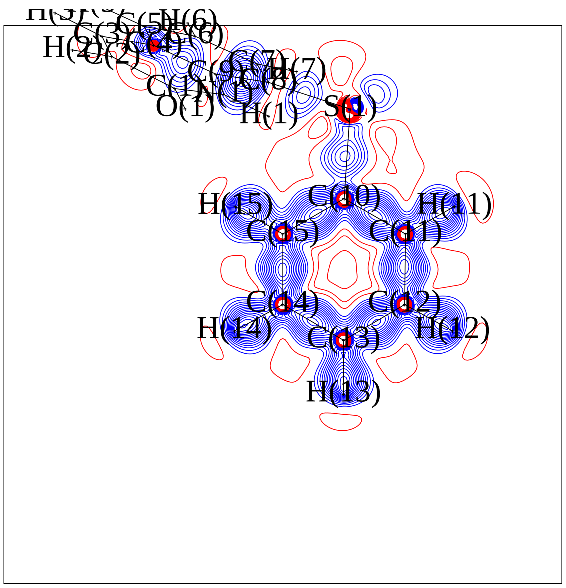

(b) XWR, harmonic, all reflections

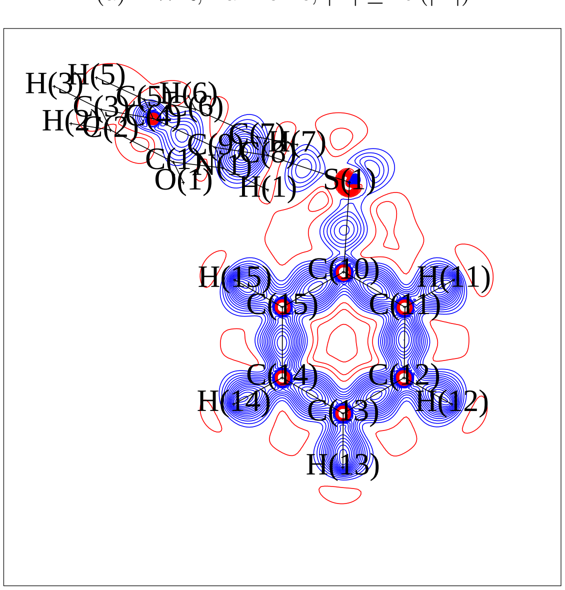

(c) XWR, anharmonic n=4, $|F| \geq 2\sigma(|F|)$

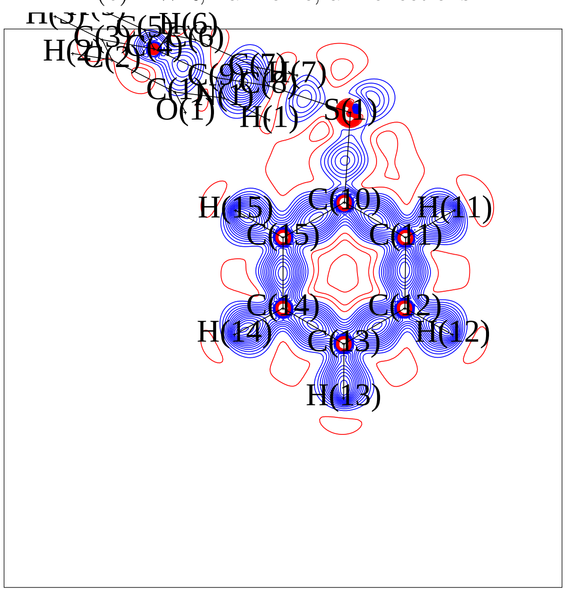

(d) XWR, anharmonic n=4, all reflections

Figure 38: Deformation density maps for PT-11 in the plane of phenyl ring, XWR, left: $|F| \geq 2\sigma(|F|)$ and right: all reflections included. Contour level: 0.05 e/Å^3. Colours: blue - positive, red – negative.

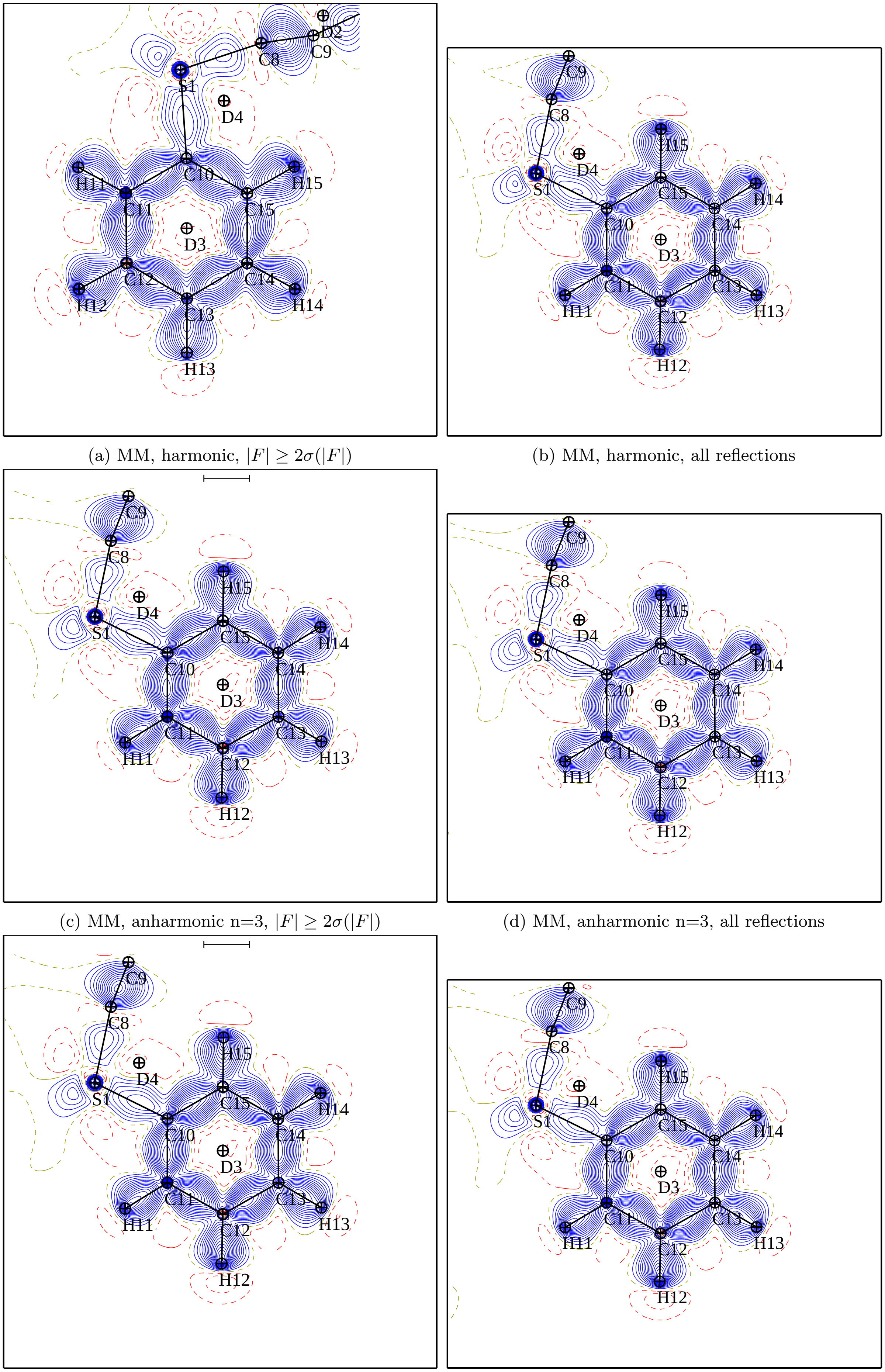

(a) MM, harmonic, $|F| \geq 2\sigma(|F|)$

(b) MM, harmonic, all reflections

(c) MM, anharmonic n=3, $|F| \geq 2\sigma(|F|)$

(d) MM, anharmonic n=3, all reflections

(e) MM, anharmonic n=4, $|F| \geq 2\sigma(|F|)$

(f) MM, anharmonic n=4, all reflections

Figure 39: Deformation density maps for PT-11 in the plane of phenyl ring, MM, restrained X-H distances, left: $|F| \geq 2\sigma(|F|)$ and right: all reflections included. Contour level: 0.05 e/Å^3. Colours: blue - positive, red – negative.

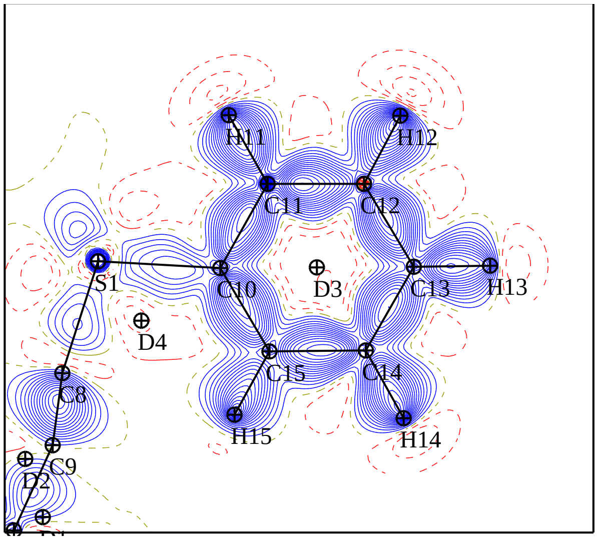

(a) MM, harmonic, $|F| \geq 2\sigma(|F|)$

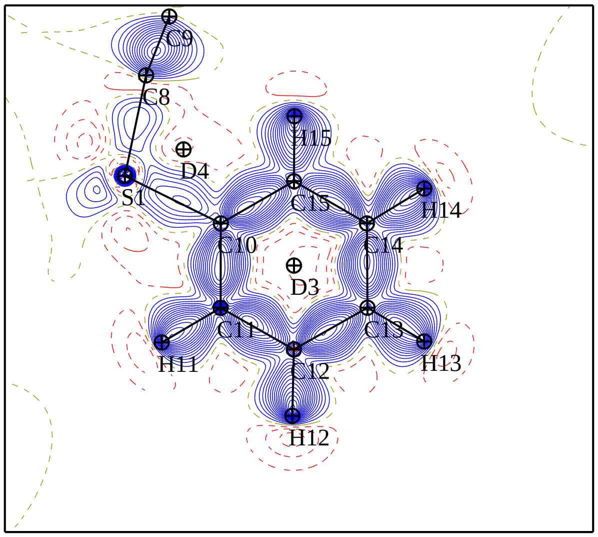

(b) MM, harmonic, all reflections

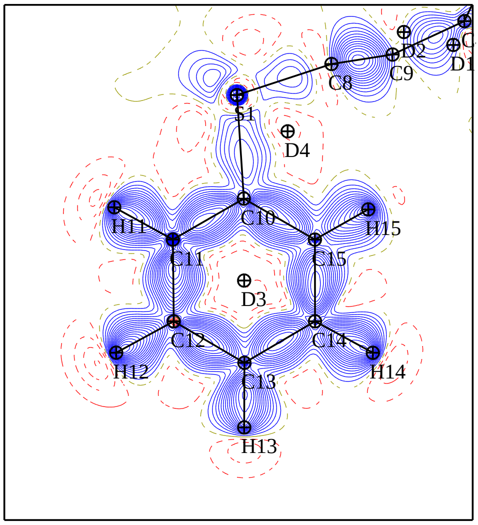

(c) MM, anharmonic n=3, $|F| \geq 2\sigma(|F|)$

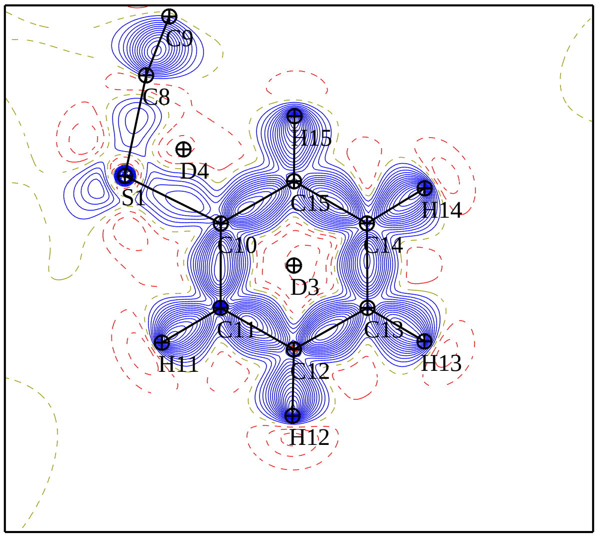

(d) MM, anharmonic n=3, all reflections

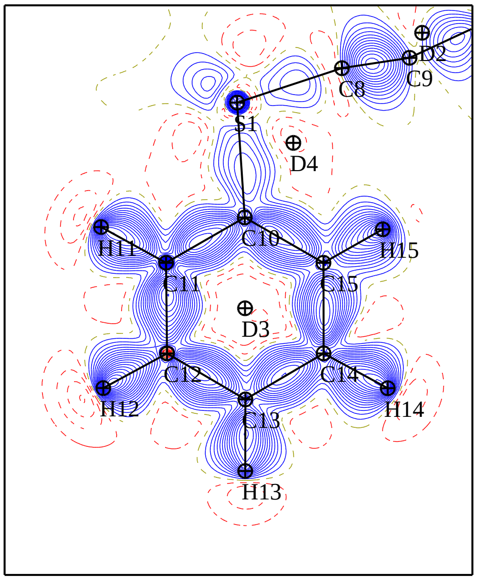

(e) MM, anharmonic n=4, $|F| \geq 2\sigma(|F|)$

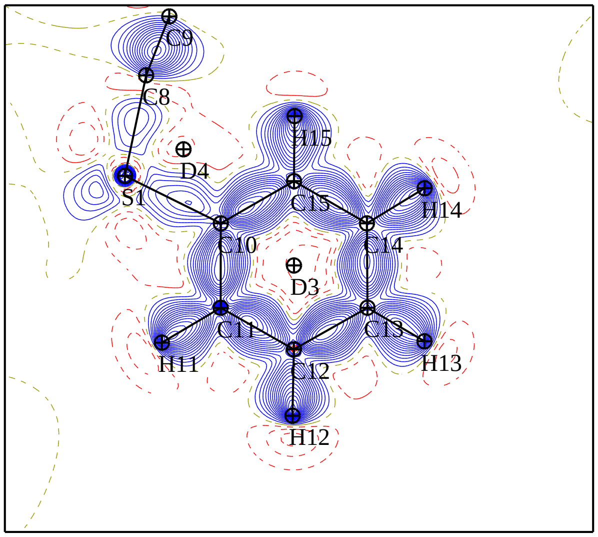

(f) MM, anharmonic n=4, all reflections

Figure 40: Deformation density maps for PT-11 in the plane of phenyl ring, MM, unrestrained X-H distances, left: $|F| \geq 2\sigma(|F|)$ and right: all reflections included. Contour level: 0.05 e/Å^3. Colours: blue - positive, red – negative.

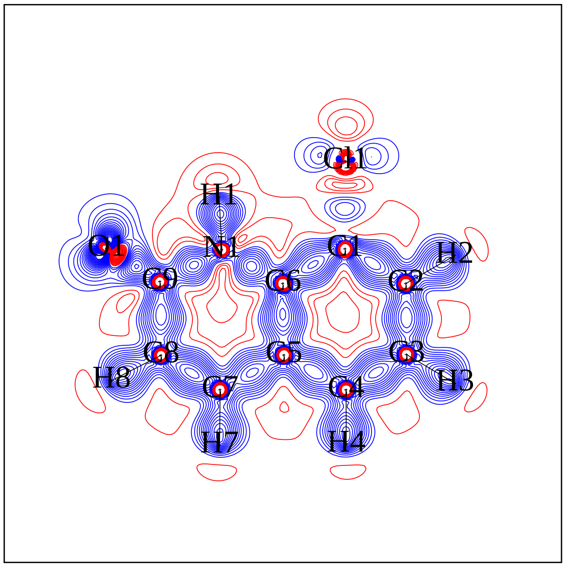

(a) HAR, harmonic, $|F| \geq 2\sigma(|F|)$

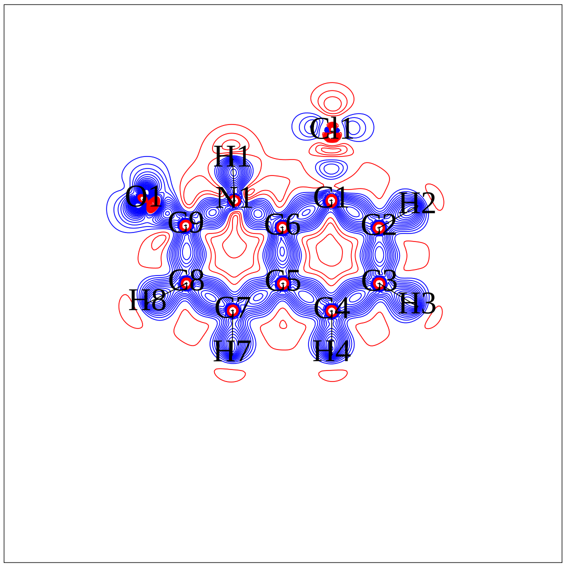

(b) HAR, harmonic, all reflections

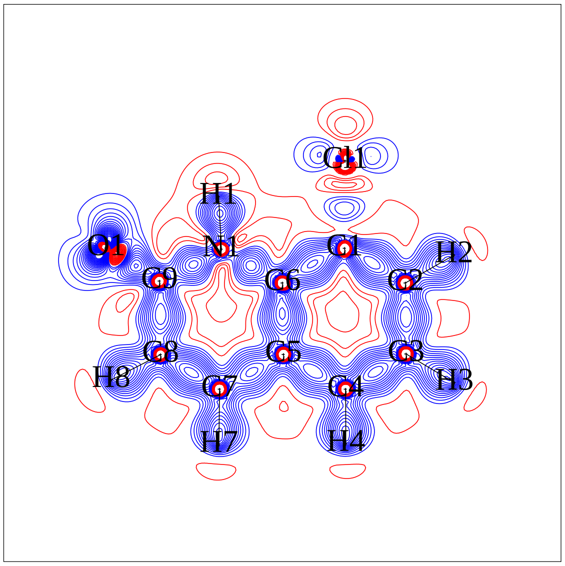

(c) HAR, anharmonic n=3, $|F| \geq 2\sigma(|F|)$

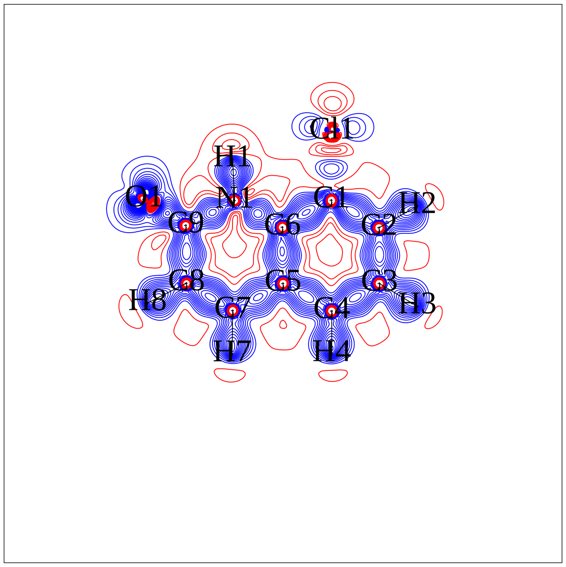

(d) HAR, anharmonic n=3, all reflections

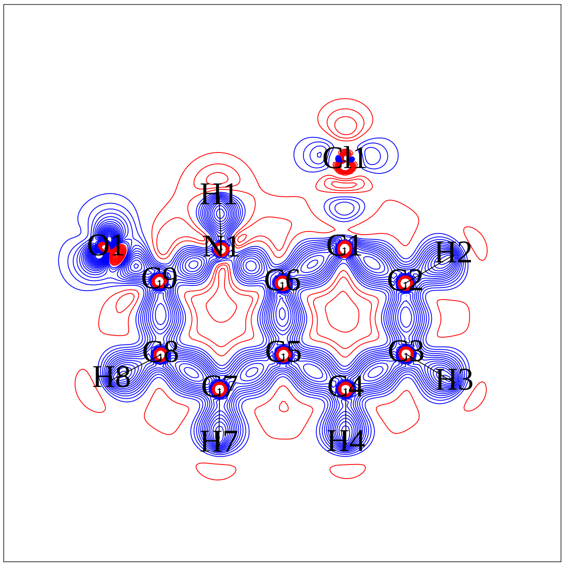

(e) HAR, anharmonic n=4, $|F| \geq 2\sigma(|F|)$

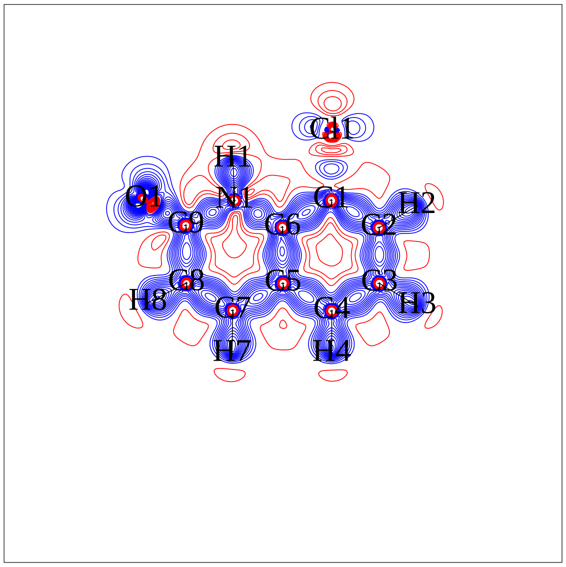

(f) HAR, anharmonic n=4, all reflections

Figure 41: Deformation density maps for PT-2, HAR, left: $|F| \geq 2\sigma(|F|)$ and right: all reflections included. Contour level: 0.05 e/Å^3. Colours: blue - positive, red – negative.

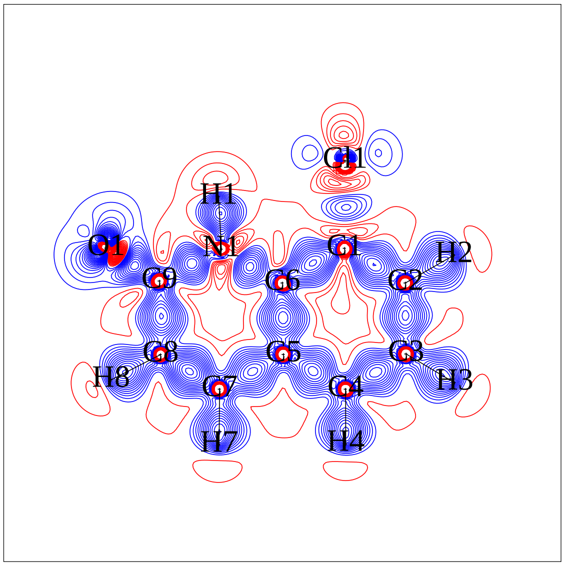

(a) XWR, harmonic, $|F| \geq 2\sigma(|F|)$

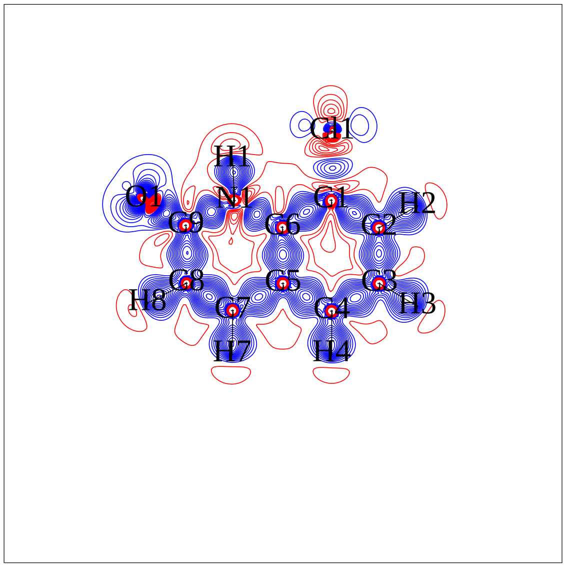

(b) XWR, harmonic, all reflections

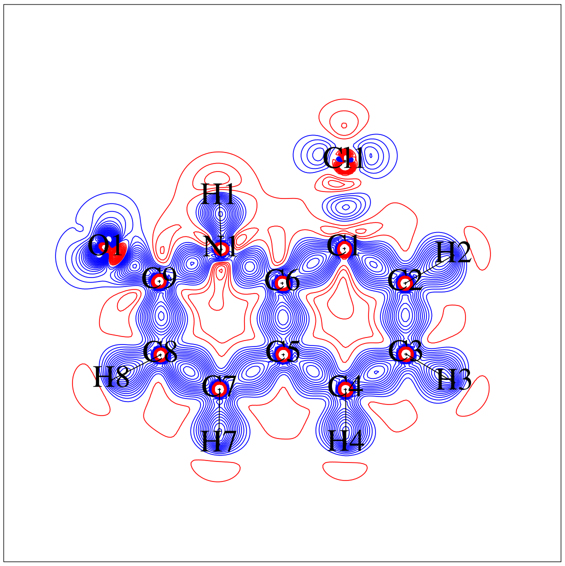

(c) XWR, anharmonic n=4, $|F| \geq 2\sigma(|F|)$

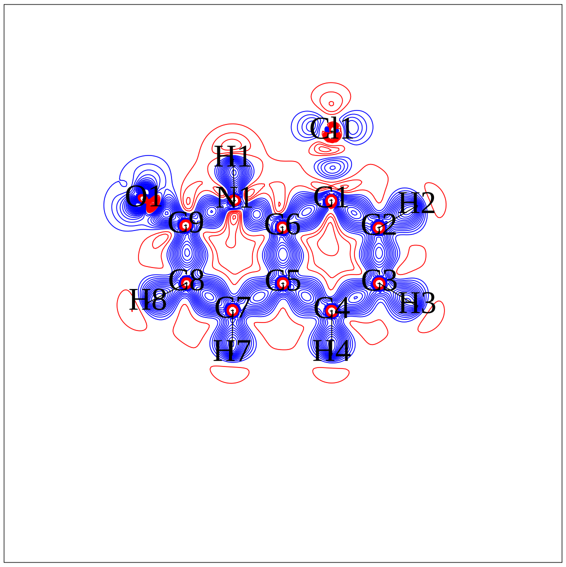

(d) XWR, anharmonic n=4, all reflections

Figure 42: Deformation density maps for PT-2, XWR, left: $|F| \geq 2\sigma(|F|)$ and right: all reflections included. Contour level: 0.05 e/Å^3. Colours: blue - positive, red – negative.

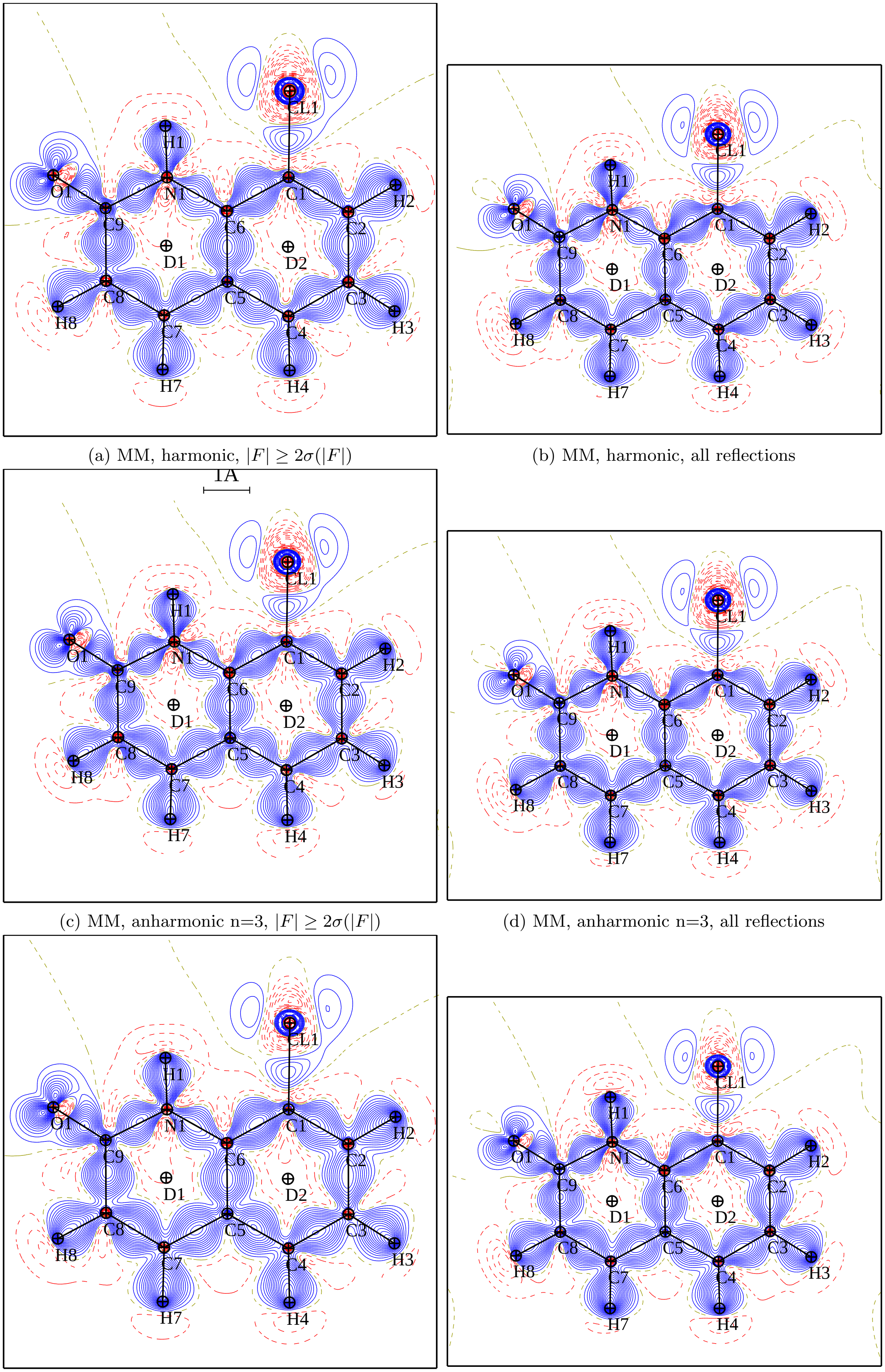

(a) MM, harmonic, $|F| \geq 2\sigma(|F|)$

(b) MM, harmonic, all reflections

(c) MM, anharmonic n=3, $|F| \geq 2\sigma(|F|)$

(d) MM, anharmonic n=3, all reflections

(e) MM, anharmonic n=4, $|F| \geq 2\sigma(|F|)$

(f) MM, anharmonic n=4, all reflections

Figure 43: Deformation density maps for PT-2, MM, restrained X-H distances, left: $|F| \geq 2\sigma(|F|)$ and right: all reflections included. Contour level: 0.05 e/Å^3. Colours: blue - positive, red – negative.

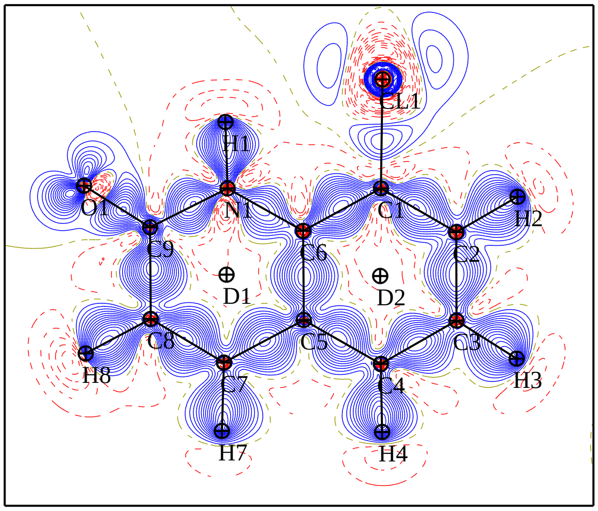

(a) MM, harmonic, $|F| \geq 2\sigma(|F|)$

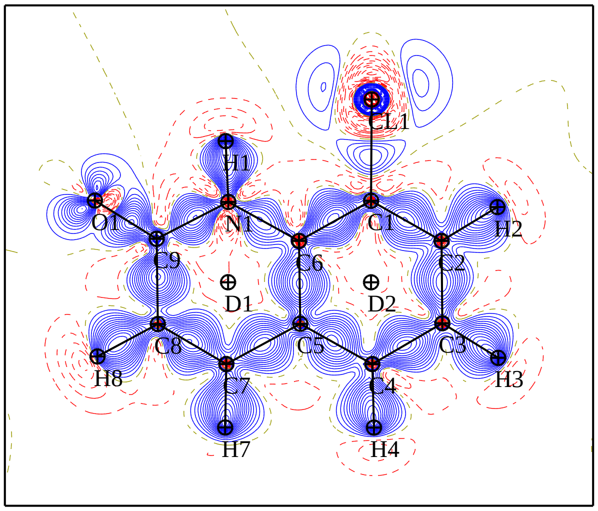

(b) MM, harmonic, all reflections

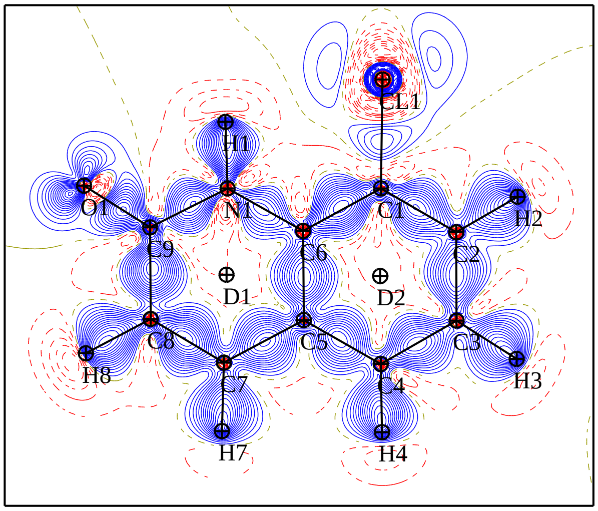

(c) MM, anharmonic n=3, $|F| \geq 2\sigma(|F|)$

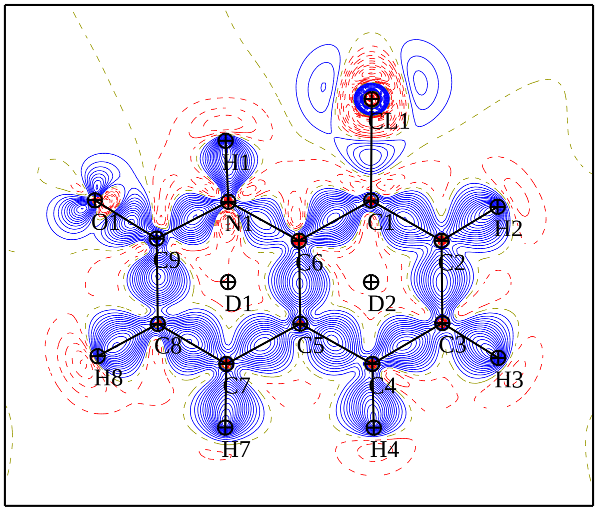

(d) MM, anharmonic n=3, all reflections

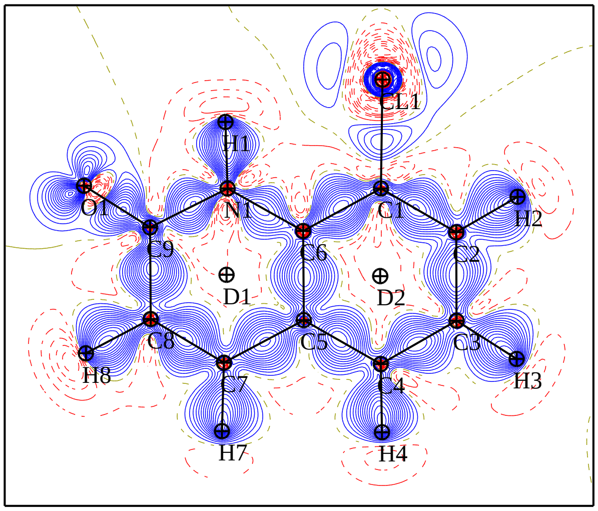

(e) MM, anharmonic n=4, $|F| \geq 2\sigma(|F|)$

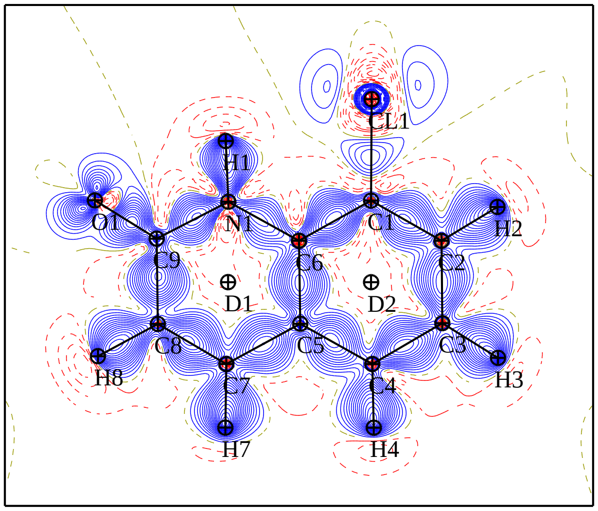

(f) MM, anharmonic n=4, all reflections

Figure 44: Deformation density maps for PT-2, MM, unrestrained X-H distances, left: $|F| \geq 2\sigma(|F|)$ and right: all reflections included. Contour level: 0.05 e/Å^3. Colours: blue - positive, red – negative.

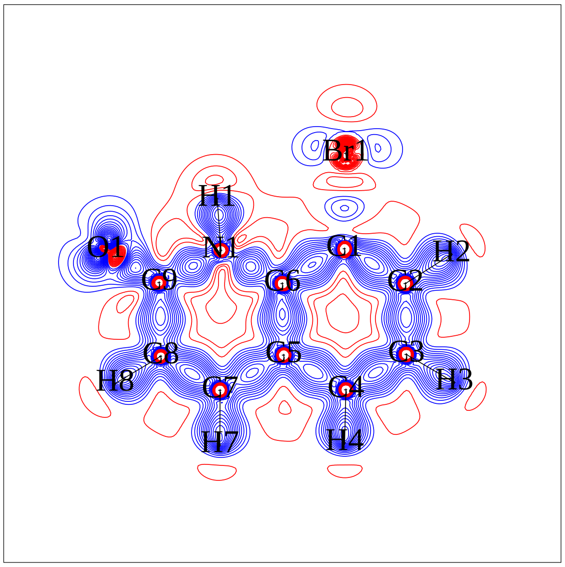

(a) HAR, harmonic, $|F| \geq 2\sigma(|F|)$

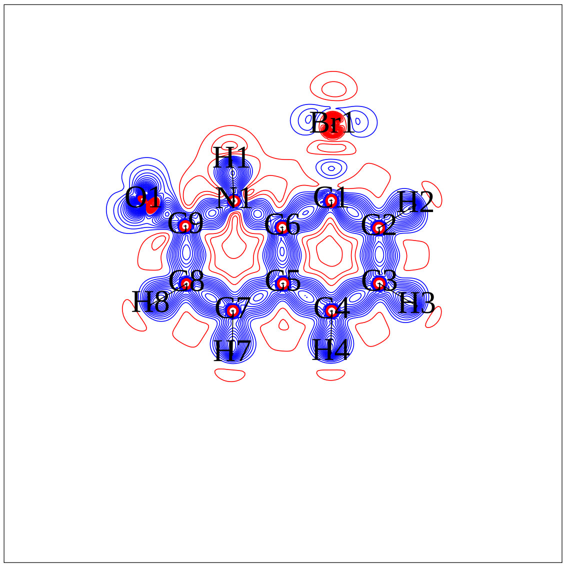

(b) HAR, harmonic, all reflections

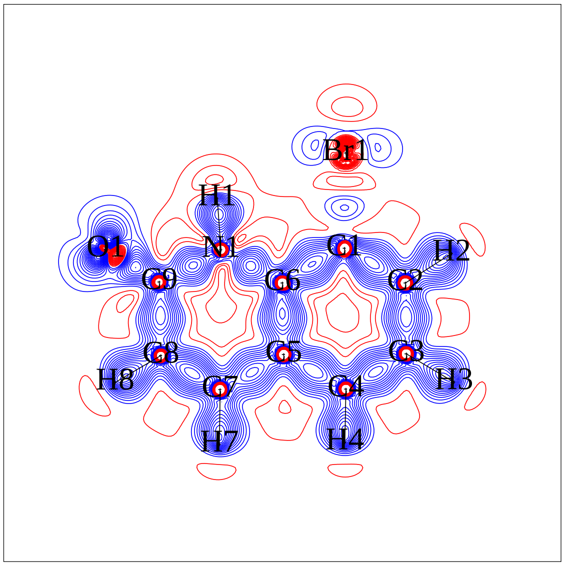

(c) HAR, anharmonic n=3, $|F| \geq 2\sigma(|F|)$

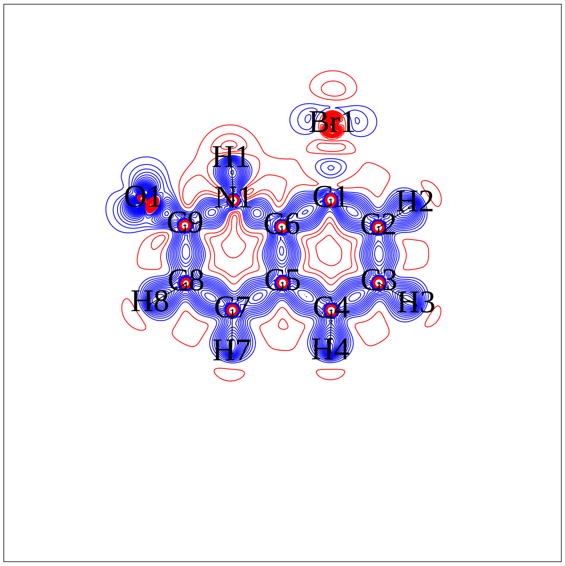

(d) HAR, anharmonic n=3, all reflections

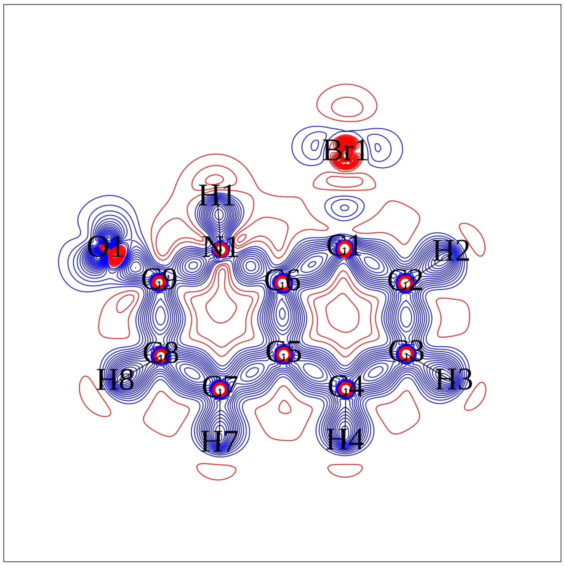

(e) HAR, anharmonic n=4, $|F| \geq 2\sigma(|F|)$

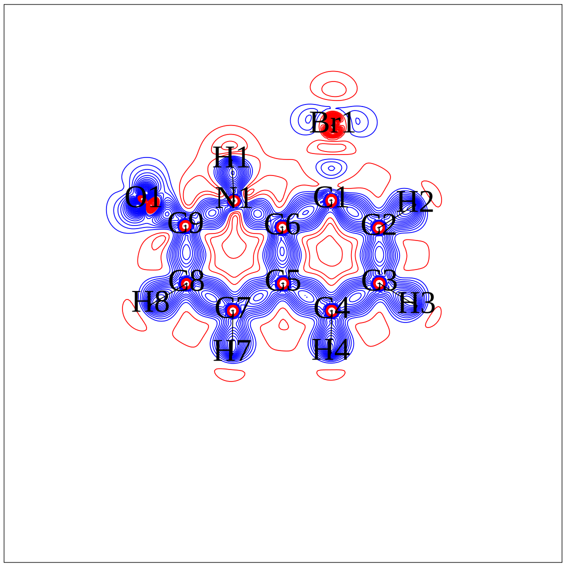

(f) HAR, anharmonic n=4, all reflections

Figure 45: Deformation density maps for PT-8, HAR, left: $|F| \geq 2\sigma(|F|)$ and right: all reflections included. Contour level: 0.05 e/Å^3. Colours: blue - positive, red – negative.

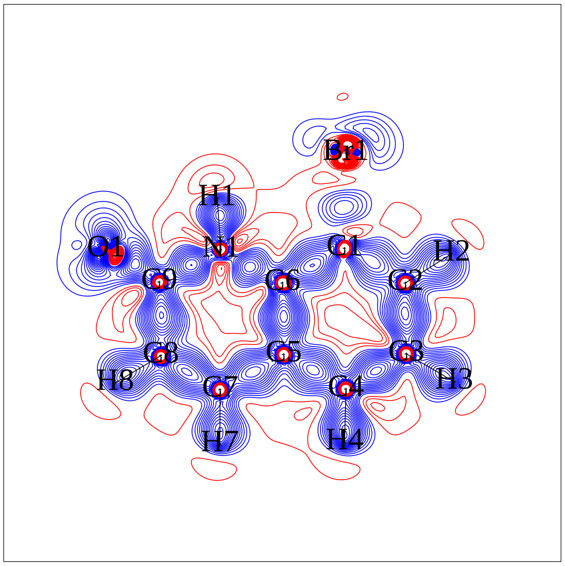

(a) XWR, harmonic, $|F| \geq 2\sigma(|F|)$

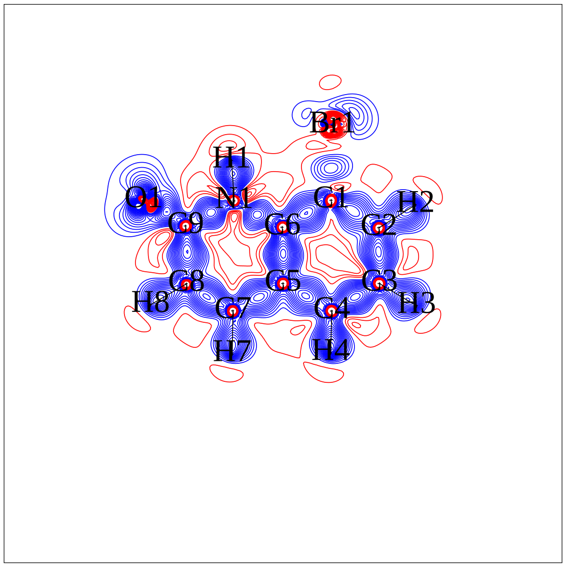

(b) XWR, harmonic, all reflections

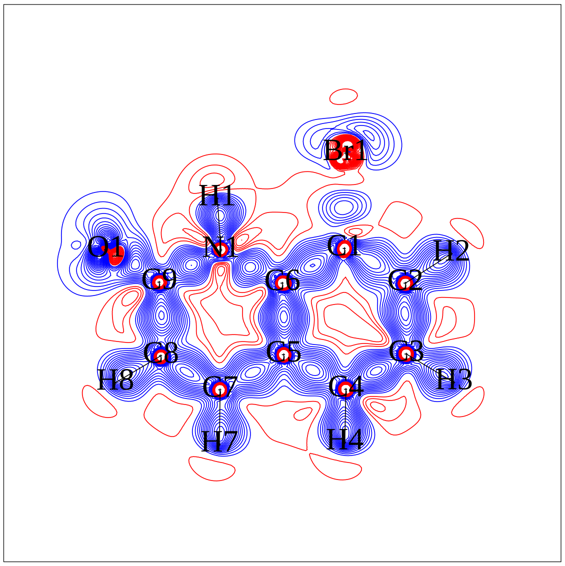

(c) XWR, anharmonic n=4, $|F| \geq 2\sigma(|F|)$

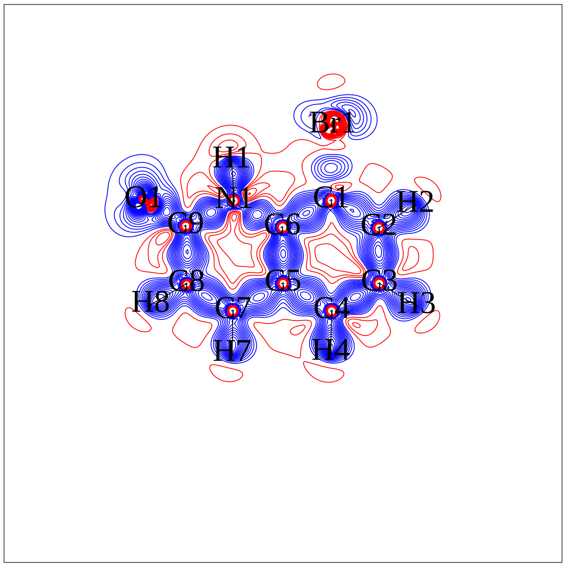

(d) XWR, anharmonic n=4, all reflections

Figure 46: Deformation density maps for PT-8, XWR, left: $|F| \geq 2\sigma(|F|)$ and right: all reflections included. Contour level: 0.05 e/Å^3. Colours: blue - positive, red – negative.

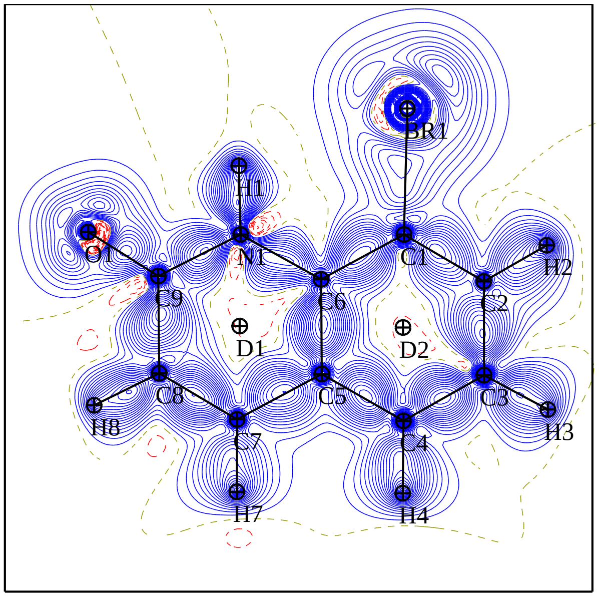

(a) MM, harmonic, $|F| \geq 2\sigma(|F|)$

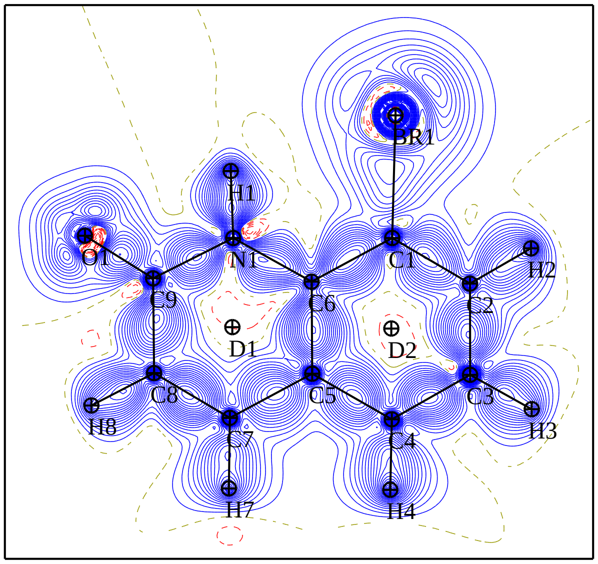

(b) MM, harmonic, all reflections

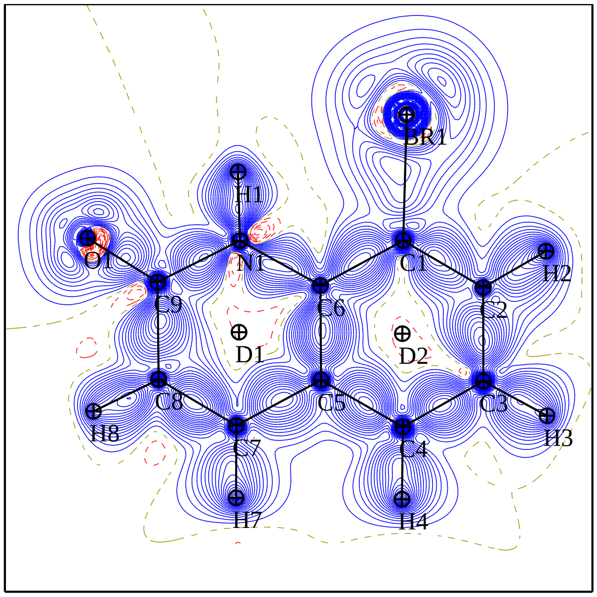

(c) MM, anharmonic n=3, $|F| \geq 2\sigma(|F|)$

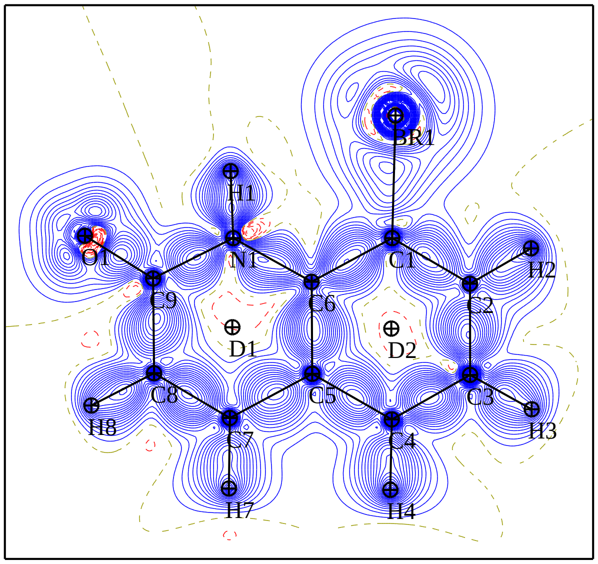

(d) MM, anharmonic n=3, all reflections

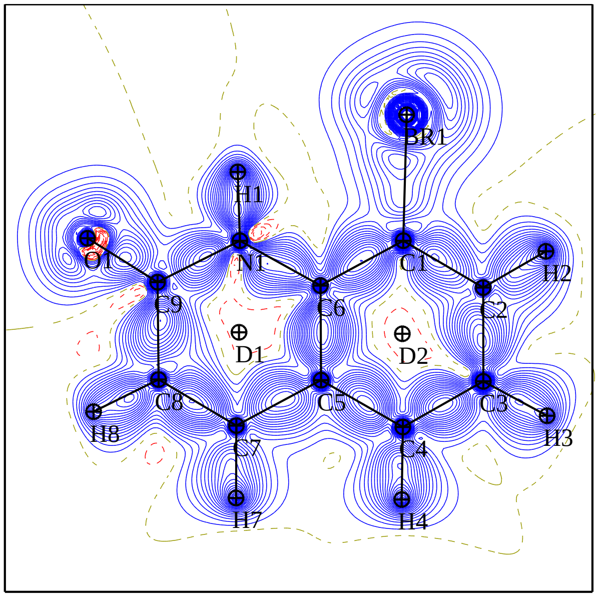

(e) MM, anharmonic n=4, $|F| \geq 2\sigma(|F|)$

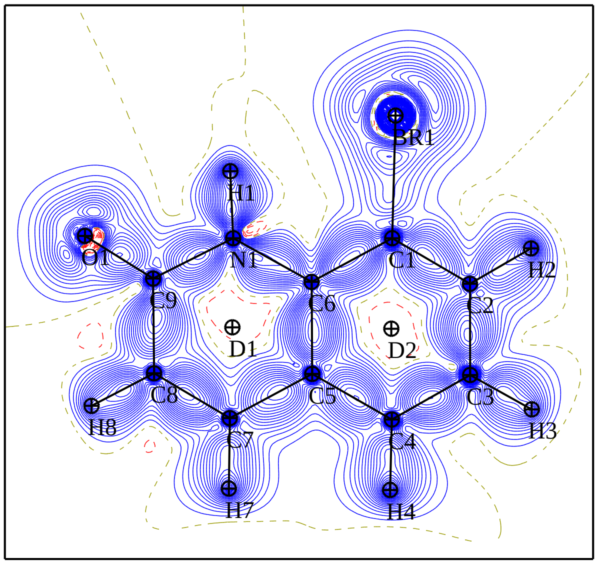

(f) MM, anharmonic n=4, all reflections

Figure 47: Deformation density maps for PT-8, MM, restrained X-H distances, left: $|F| \geq 2\sigma(|F|)$ and right: all reflections included. Contour level: 0.05 e/Å^3. Colours: blue - positive, red – negative.

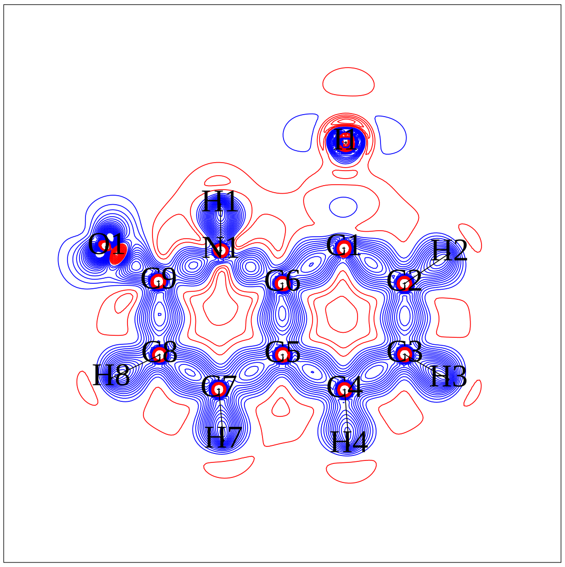

(a) HAR, harmonic, $|F| \geq 2\sigma(|F|)$

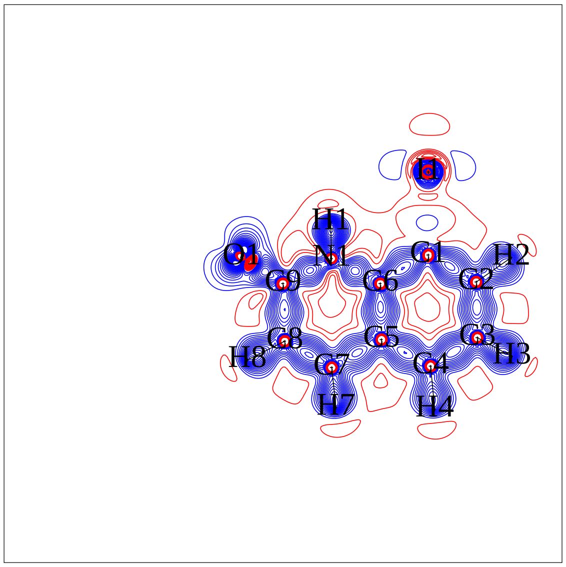

(b) HAR, harmonic, all reflections

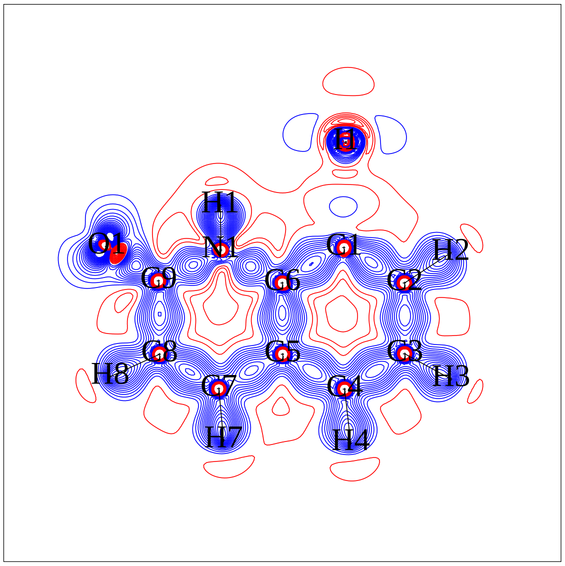

(c) HAR, anharmonic n=3, $|F| \geq 2\sigma(|F|)$

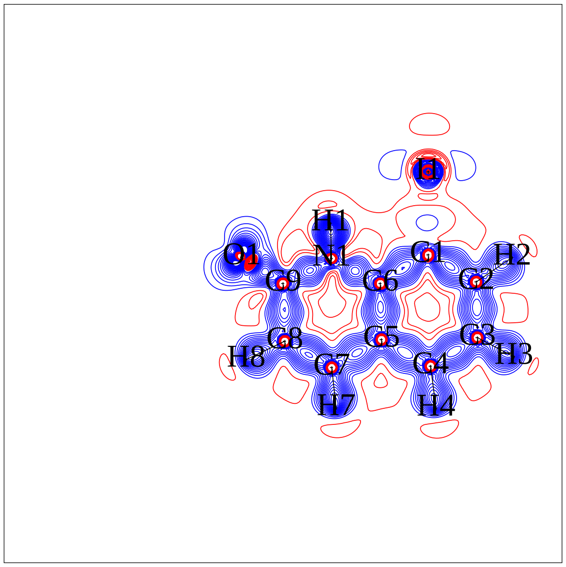

(d) HAR, anharmonic n=3, all reflections

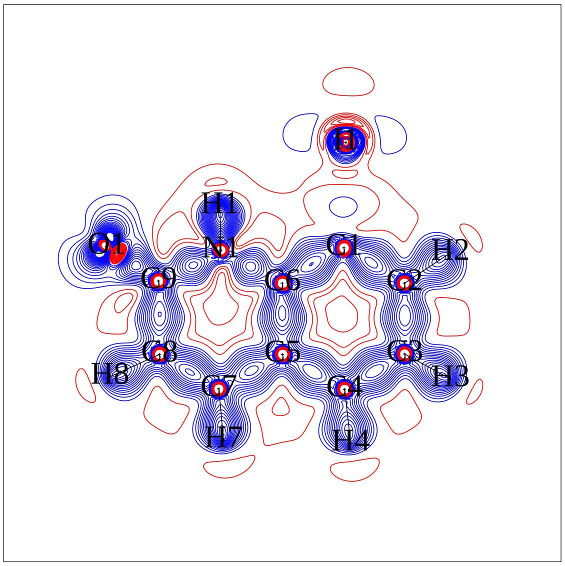

(e) HAR, anharmonic n=4, $|F| \geq 2\sigma(|F|)$

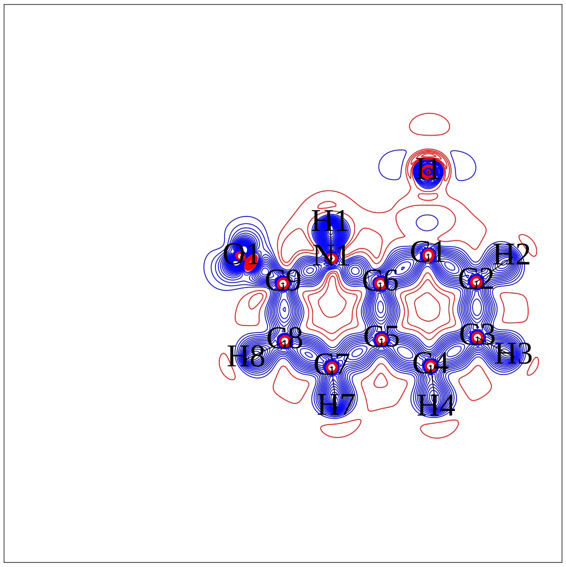

(f) HAR, anharmonic n=4, all reflections

Figure 48: Deformation density maps for PT-10, HAR, left: $|F| \geq 2\sigma(|F|)$ and right: all reflections included. Contour level: 0.05 e/Å^3. Colours: blue - positive, red – negative.

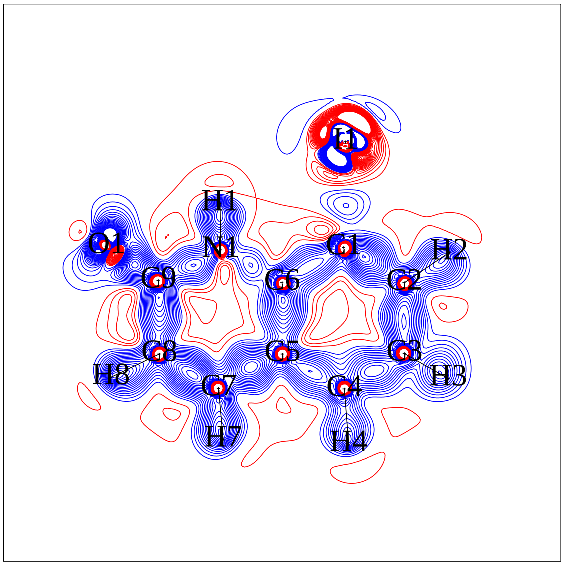

(a) XWR, harmonic, $|F| \geq 2\sigma(|F|)$

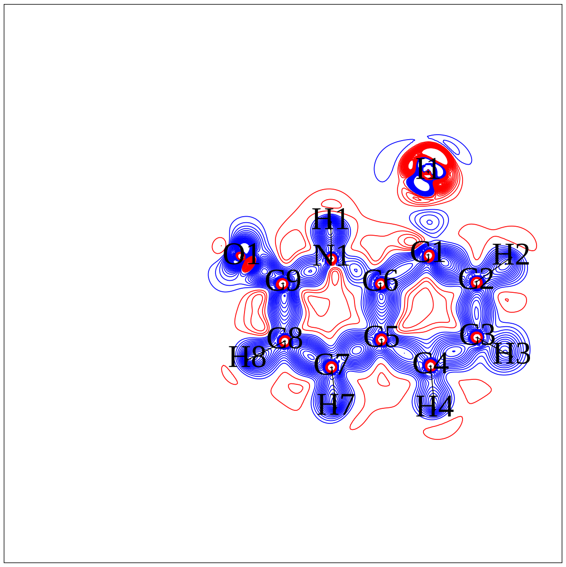

(b) XWR, harmonic, all reflections

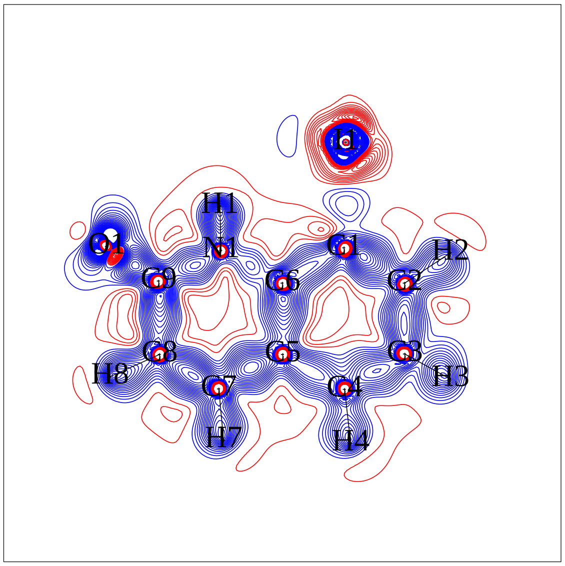

(c) XWR, anharmonic n=4, $|F| \geq 2\sigma(|F|)$

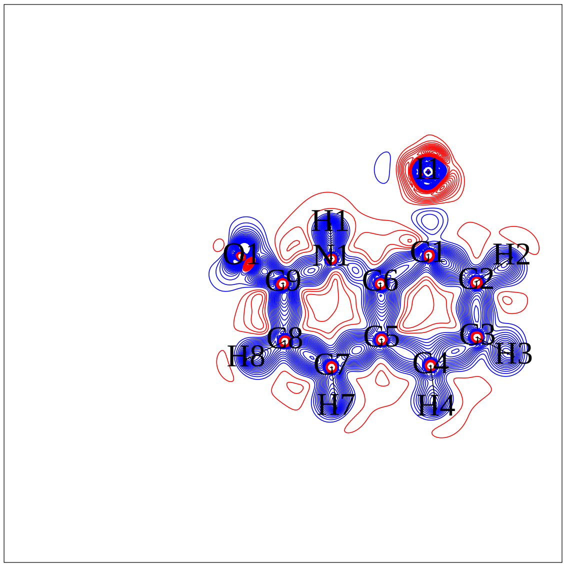

(d) XWR, anharmonic n=4, all reflections

Figure 49: Deformation density maps for PT-10, XWR, left: $|F| \geq 2\sigma(|F|)$ and right: all reflections included. Contour level: 0.05 e/Å^3. Colours: blue - positive, red – negative.

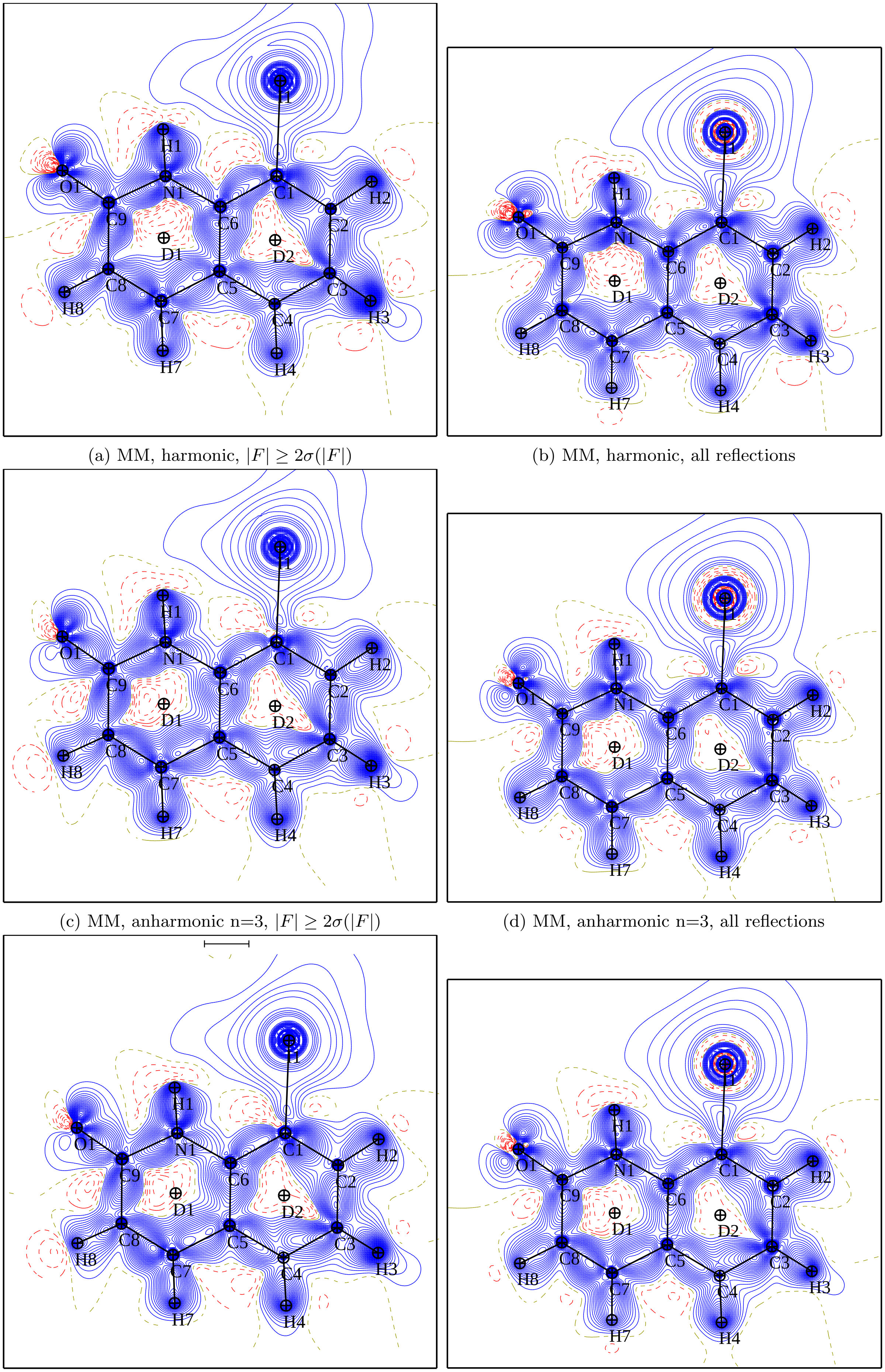

(a) MM, harmonic, $|F| \geq 2\sigma(|F|)$

(b) MM, harmonic, all reflections

(c) MM, anharmonic n=3, $|F| \geq 2\sigma(|F|)$

(d) MM, anharmonic n=3, all reflections

(e) MM, anharmonic n=4, $|F| \geq 2\sigma(|F|)$

(f) MM, anharmonic n=4, all reflections

Figure 50: Deformation density maps for PT-10, MM, restrained X-H distances, left: $|F| \geq 2\sigma(|F|)$ and right: all reflections included. Contour level: 0.05 e/Å^3. Colours: blue - positive, red – negative.

6 Structural motifs

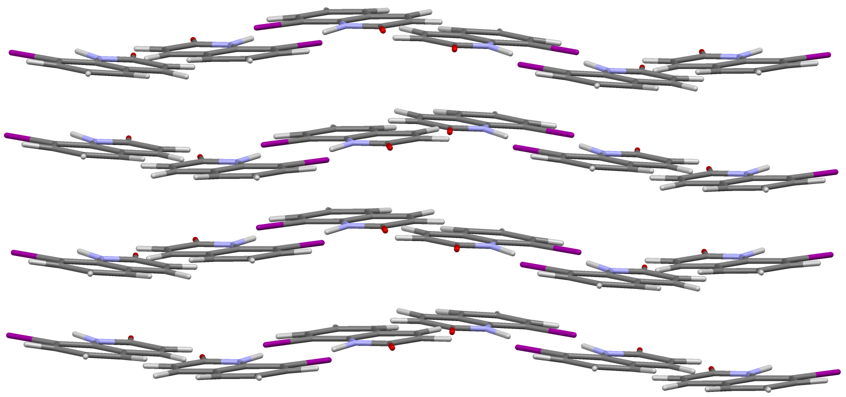

(a) Layer motifs in (102) plane.

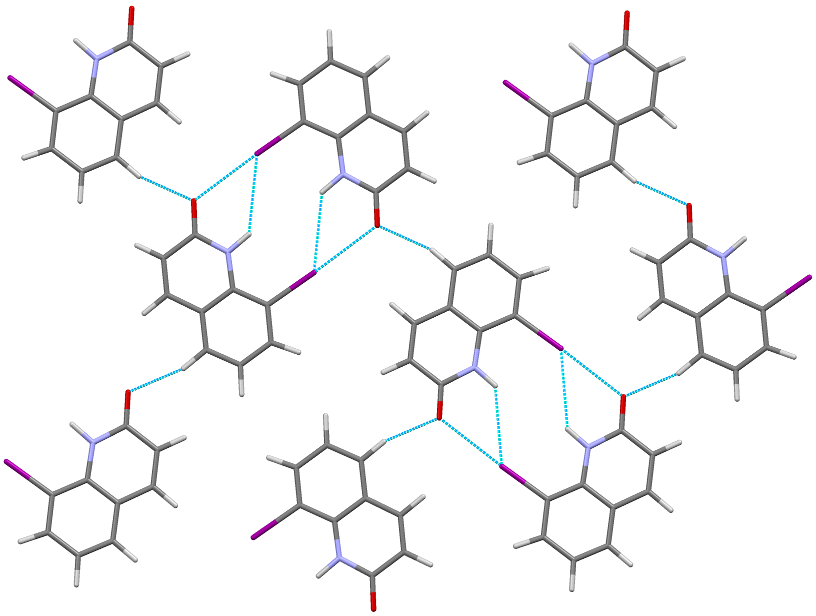

(b) Network of short contacts within a slab.

Figure 51: The layered structure of PT-10(I).

Table 28: QTAIM parameters at bond critical points of selected interactions. Intensity cutoff: $|F| \geq 2\sigma(|F|)$.

structure	dimer	interaction	method	R $[\AA]$	R_1 $[\AA]$	R_2 $[\AA]$	$\rho(r_{CP})$ $[e/\AA^3]$	$\nabla^2\rho(r_{CP})$ $[e/\AA^5]$	$G(r_{CP})$ $[kJ/(mol\cdot a_0^3)]$	$V(r_{CP})$ $[kJ/(mol\cdot a_0^3)]$
PT-11(S-Ph)	dimer1	H1···O1	MM(freeXH), harm	2.0256	0.8004	1.2254	0.16424	1.821	48.47	-47.35
			MM(freeXH), anh, n=3	2.0162	0.7899	1.2265	0.16381	1.781	47.67	-46.85
			MM(freeXH), anh, n=3,4	2.0183	0.7929	1.2257	0.16406	1.787	47.82	-46.98
			MM, harm res	1.9747	0.7403	1.2350	0.15993	1.593	43.66	-43.94
			MM, anh, n=3 res	1.9753	0.7388	1.2371	0.15789	1.595	43.40	-43.34
			MM, anh, n=3,4 res	1.9753	0.7387	1.2372	0.15736	1.600	43.40	-43.22
PT-2(Cl)	dimer1	Cl1···O1	MM(freeXH), harm	3.1355	1.7296	1.4101	0.07043	0.968	21.33	-16.30
			MM(freeXH), anh, n=3	3.1355	1.7298	1.4100	0.07047	0.969	21.35	-16.32
			MM(freeXH), anh, n=3,4	3.1355	1.7298	1.4100	0.07047	0.969	21.35	-16.32
			MM, harm	3.1355	1.7300	1.4093	0.07037	0.967	21.32	-16.29
			MM, anh, n=3	3.1355	1.7300	1.4095	0.07036	0.970	21.36	-16.31
			MM, anh, n=3,4	3.1355	1.7223	1.4165	0.06616	0.949	20.61	-15.38
		H1···O1	MM(freeXH), harm	1.8202	0.6507	1.1704	0.23539	1.451	54.42	-69.32
			MM(freeXH), anh, n=3	1.8175	0.6481	1.1703	0.23717	1.387	53.61	-69.44
			MM(freeXH), anh, n=3,4	1.8175	0.6481	1.1703	0.23717	1.387	53.61	-69.44
			MM, harm	1.8422	0.6735	1.1691	0.23270	1.699	58.39	-70.50
			MM, anh, n=3	1.8418	0.6740	1.1683	0.23427	1.663	58.04	-70.80
			MM, anh, n=3,4	1.8421	0.6687	1.1738	0.22730	1.696	57.27	-68.36
	halogen	Cl1···C8	MM(freeXH), harm	3.4120	1.8138	1.6501	0.05044	0.525	11.69	-9.07
			MM(freeXH), anh, n=3	3.4123	1.8144	1.6478	0.05034	0.524	11.67	-9.05
			MM(freeXH), anh, n=3,4	3.4123	1.8144	1.6478	0.05034	0.524	11.67	-9.06
			MM, harm	3.4120	1.8143	1.6362	0.05030	0.533	11.82	-9.13
			MM, anh, n=3	3.4123	1.8142	1.6361	0.05021	0.531	11.78	-9.10
			MM, anh, n=3,4	3.4123	1.8052	1.6430	0.04604	0.511	11.12	-8.34
PT-8(Br)	dimer1	Br1···O1	MM, harm	3.1964	1.7822	1.4189	0.09298	1.120	26.30	-22.11
			MM, anh, n=3	3.1965	1.7846	1.4169	0.09329	1.128	26.49	-22.25
			MM, anh, n=3,4	3.1964	1.7692	1.4330	0.08609	1.080	24.86	-20.31
		H1···O1	MM, harm	1.8784	0.7166	1.1623	0.27880	2.450	81.70	-96.68
			MM, anh, n=3	1.8780	0.7195	1.1590	0.28401	2.411	82.17	-98.66
			MM, anh, n=3,4	1.8772	0.7176	1.1600	0.28483	2.479	83.58	-99.65
	halogen	Br1···H7	MM, harm	3.0654	1.8280	1.2764	0.08330	0.800	19.49	-17.20
			MM, anh, n=3	3.0623	1.8302	1.2712	0.08239	0.806	19.52	-17.08
			MM, anh, n=3,4	3.0611	1.8134	1.2899	0.07620	0.781	18.46	-15.65
PT-10(I)	halogen	I1···O1	MM, harm	2.9167	1.6834	1.2438	0.24661	1.705	61.30	-76.16
			MM, anh, n=3	2.9215	1.6822	1.2477	0.24348	1.737	61.24	-75.17
			MM, anh, n=3,4	2.9217	1.6794	1.2459	0.24788	1.764	62.63	-77.21
		I1···H1	MM, harm	2.9691	2.1952	1.3561	0.15512	0.011	14.21	-28.12
			MM, anh, n=3	2.9677	2.1237	1.2149	0.13742	0.062	12.58	-23.46
			MM, anh, n=3,4	2.9665	2.2478	1.3409	0.13833	-0.009	11.42	-23.07
	dimer1	C3···C6	MM, harm	3.3318	1.7156	1.7170	0.03239	0.478	9.70	-6.39
			MM, anh, n=3	3.3314	1.7332	1.7344	0.03510	0.462	9.57	-6.55
			MM, anh, n=3,4	3.3315	1.7355	1.7474	0.03605	0.454	9.48	-6.59
		C3···N1	MM, harm	3.6205	1.8064	1.8649	0.04201	0.478	10.26	-7.51
			MM, anh, n=3	3.6204	1.8041	1.8638	0.04242	0.473	10.21	-7.53
			MM, anh, n=3,4	3.6205	1.8090	1.8591	0.04276	0.475	10.25	-7.58
		C4···C7	MM, harm	3.6017	1.7391	1.8696	0.03444	0.404	8.47	-5.95
			MM, anh, n=3	3.6015	1.7525	1.8564	0.03650	0.403	8.57	-6.17
			MM, anh, n=3,4	3.6016	1.7453	1.8625	0.03548	0.403	8.51	-6.05
		N1···C5	MM, harm	3.5729	1.8295	1.7441	0.03934	0.469	9.93	-7.10
			MM, anh, n=3	3.5732	1.8362	1.7387	0.04117	0.475	10.17	-7.39
			MM, anh, n=3,4	3.5732	1.8244	1.7492	0.04214	0.473	10.18	-7.48
		C7···O1	MM, harm	3.6387	1.8526	1.8099	0.03964	0.361	7.99	-6.16
			MM, anh, n=3	3.6389	1.8566	1.8085	0.04006	0.372	8.22	-6.31
			MM, anh, n=3,4	3.6390	1.8546	1.8166	0.04015	0.368	8.16	-6.29
		C7···C8	MM, harm	3.6100	1.8829	1.8197	0.02878	0.292	6.14	-4.34
			MM, anh, n=3	3.6089	1.8862	1.8401	0.03329	0.298	6.49	-4.86
			MM, anh, n=3,4	3.6088	1.8681	1.8228	0.03328	0.302	6.55	-4.89

Table 29: QTAIM parameters at bond critical points of selected interactions. No intensity cutoff. In the case of PT-10(I) calculations of QTAIM properties for certain critical points failed.

structure	dimer	interaction	method	R $[\mathring{A}]$	R_1 $[\mathring{A}]$	R_2 $[\mathring{A}]$	$\rho(r_{CP})$ $[e/\mathring{A}^3]$	$\nabla^2\rho(r_{CP})$ $[e/\mathring{A}^5]$	$G(r_{CP})$ $[kJ/(mol\cdot a_0^3)]$	$V(r_{CP})$ $[kJ/(mol\cdot a_0^3)]$
PT-11(S-Ph)	dimer1	H1···O1	MM(freeXH), harm	2.0104	0.7772	1.2336	0.1612	1.716	46.09	-45.45
			MM(freeXH), anh, n=3	2.0046	0.7702	1.2349	0.1603	1.688	45.44	-44.92
			MM(freeXH), anh, n=3,4	2.0061	0.7724	1.2342	0.1606	1.696	45.63	-45.07
			MM, harm res	1.9750	0.7338	1.2420	0.1579	1.539	42.37	-42.83
			MM, anh, n=3 res	1.9756	0.7353	1.2412	0.1579	1.532	42.24	-42.76
			MM, anh, n=3,4 res	1.9755	0.7343	1.2421	0.1569	1.536	42.16	-42.49
PT-2(Cl)	dimer1	Cl1···O1	MM(freeXH) harm	3.1356	1.7387	1.4012	0.0708	0.953	21.09	-16.23
			MM(freeXH) anh, n=3	3.1356	1.7383	1.4017	0.0706	0.953	21.08	-16.19
			MM(freeXH) anh, n=3,4	3.1356	1.7303	1.4093	0.0665	0.933	20.35	-15.29
			MM, harm	3.1356	1.7387	1.4011	0.0708	0.953	21.10	-16.23
			MM, anh, n=3	3.1356	1.7383	1.4017	0.0706	0.953	21.08	-16.19
			MM, anh, n=3,4	3.1356	1.7303	1.4093	0.0665	0.933	20.35	-15.29
		H1···O1	MM(freeXH) harm	1.8339	0.6538	1.1804	0.2130	1.774	55.98	-63.64
			MM(freeXH) anh, n=3	1.8325	0.6525	1.1804	0.2140	1.730	55.36	-63.62
			MM(freeXH) anh, n=3,4	1.8356	0.6497	1.1861	0.2073	1.790	55.22	-61.68
			MM, harm	1.8339	0.6539	1.1804	0.2130	1.774	55.97	-63.63
			MM, anh, n=3	1.8325	0.6525	1.1804	0.2140	1.730	55.36	-63.62
			MM, anh, n=3,4	1.8356	0.6497	1.1861	0.2073	1.790	55.22	-61.68
	halogen	Cl1···C8	MM(freeXH) harm	3.4121	1.8076	1.6653	0.0510	0.519	11.62	-9.10
			MM(freeXH) anh, n=3	3.4123	1.8073	1.6628	0.0511	0.519	11.62	-9.11
			MM(freeXH) anh, n=3,4	3.4124	1.7983	1.6698	0.0467	0.500	10.97	-8.33
			MM, harm	3.4121	1.8076	1.6654	0.0510	0.519	11.62	-9.10
			MM, anh, n=3	3.4123	1.8073	1.6628	0.0511	0.519	11.62	-9.11
			MM, anh, n=3,4	3.4124	1.7983	1.6698	0.0467	0.500	10.97	-8.33
PT-8(Br)	dimer1	Br1···O1	MM harm	3.1966	1.7868	1.4150	0.0923	1.123	26.28	-21.98
			MM, anh, n=3	3.1966	1.7887	1.4134	0.0926	1.130	26.45	-22.11
			MM, anh, n=3,4	3.1966	1.7632	1.4400	0.0809	1.052	23.84	-19.03
		H1···O1	MM, harm	1.8750	0.7157	1.1597	0.2780	2.376	80.19	-95.67
			MM, anh, n=3	1.8746	0.7181	1.1570	0.2823	2.356	80.79	-97.41
			MM, anh, n=3,4	1.8735	0.7162	1.1577	0.2855	2.438	82.98	-99.56
	halogen	Br1···H7	MM, harm	3.0643	1.8311	1.2591	0.0830	0.798	19.43	-17.13
			MM, anh, n=3	3.0610	1.8334	1.2532	0.0820	0.803	19.42	-16.97
			MM, anh, n=3,4	3.0604	1.8077	1.2784	0.0701	0.749	17.34	-14.26
PT-10(I)	halogen	I1···O1	MM harm	2.9168	1.6929	1.2259	0.2653	2.024	71.04	-86.93
			MM, anh, n=3	2.9212	1.6998	1.2233	0.2768	1.964	72.45	-91.40
			MM, anh, n=3,4	2.9213	1.6949	1.2274	0.2672	1.980	70.64	-87.33
		I1···H1	MM, harm	2.9682	2.0278	1.0935	0.1402	0.263	16.61	-26.05
			MM, anh, n=3	2.9747	2.0646	1.0182	0.1325	0.252	15.35	-23.84
			MM, anh, n=3,4	2.9740	2.0796	1.0369	0.1274	0.236	14.37	-22.31
	dimer1	C3···C6	MM, harm	N/A	N/A	N/A	N/A	N/A	N/A	N/A
			MM, anh, n=3	3.3309	1.7436	1.6890	0.0384	0.485	10.16	-7.13
			MM, anh, n=3,4	3.3309	1.7387	1.6903	0.0386	0.488	10.24	-7.19
		C3···N1	MM, harm	N/A	N/A	N/A	N/A	N/A	N/A	N/A
			MM, anh, n=3	3.6212	1.8986	1.8213	0.0430	0.484	10.43	-7.70
			MM, anh, n=3,4	N/A	N/A	N/A	N/A	N/A	N/A	N/A
		C4···C7	MM, harm	N/A	N/A	N/A	N/A	N/A	N/A	N/A
			MM, anh, n=3	3.6013	1.7553	1.8867	0.0393	0.399	8.66	-6.46
			MM, anh, n=3,4	3.6013	1.7599	1.8796	0.0411	0.404	8.87	-6.73
		N1···C5	MM, harm	N/A	N/A	N/A	N/A	N/A	N/A	N/A
			MM, anh, n=3	N/A	N/A	N/A	N/A	N/A	N/A	N/A
			MM, anh, n=3,4	N/A	N/A	N/A	N/A	N/A	N/A	N/A
		C7···O1	MM, harm	3.6381	1.8187	1.8313	0.0397	0.354	7.88	-6.10
			MM, anh, n=3	3.6382	1.8184	1.8358	0.0386	0.350	7.73	-5.94
			MM, anh, n=3,4	3.6383	1.8231	1.8355	0.0393	0.352	7.81	-6.04
		C7···C8	MM, harm	N/A	N/A	N/A	N/A	N/A	N/A	N/A
			MM, anh, n=3	N/A	N/A	N/A	N/A	N/A	N/A	N/A
			MM, anh, n=3,4	N/A	N/A	N/A	N/A	N/A	N/A	N/A

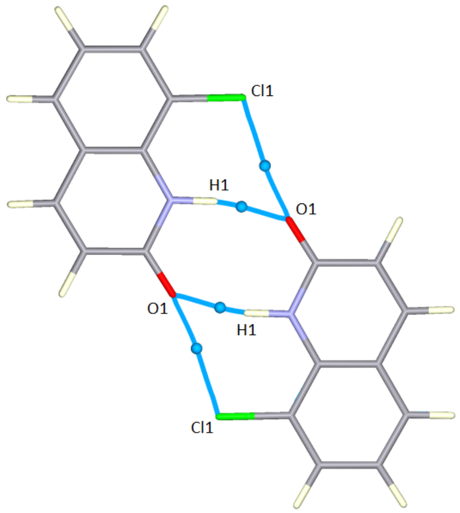

(a) PT-2(Cl), dimer1

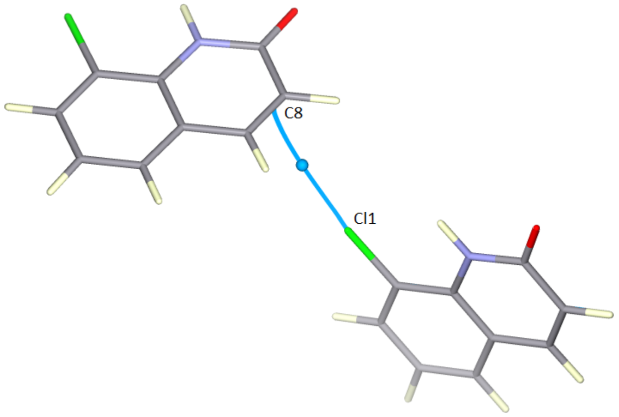

(b) PT-2(Cl), halogen

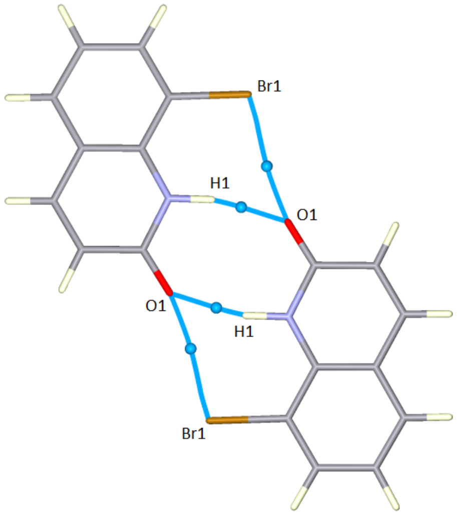

(c) PT-8(Br), dimer1

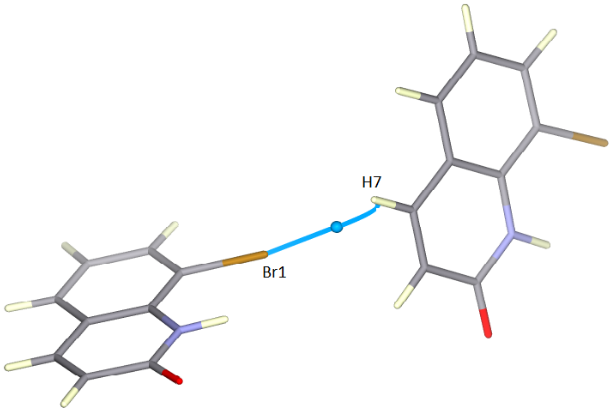

(d) PT-8(Br), halogen

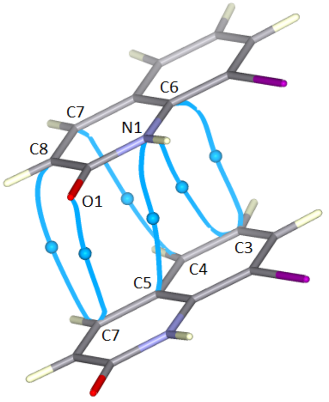

(e) PT-10(I), dimer1

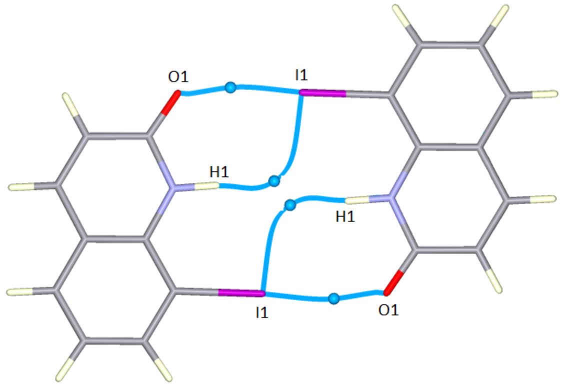

(f) PT-10(I), halogen

Figure 52: Bond critical points found for hydrogen, halogen and stacking interactions.

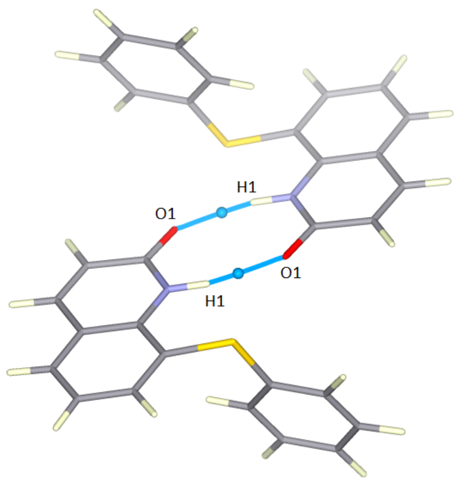

(a) PT-11(S-Ph), dimer1

Figure 53: Bond critical points found for hydrogen, halogen and stacking interactions.

7 Dimer interaction energy as a function of centroid separation of dimers

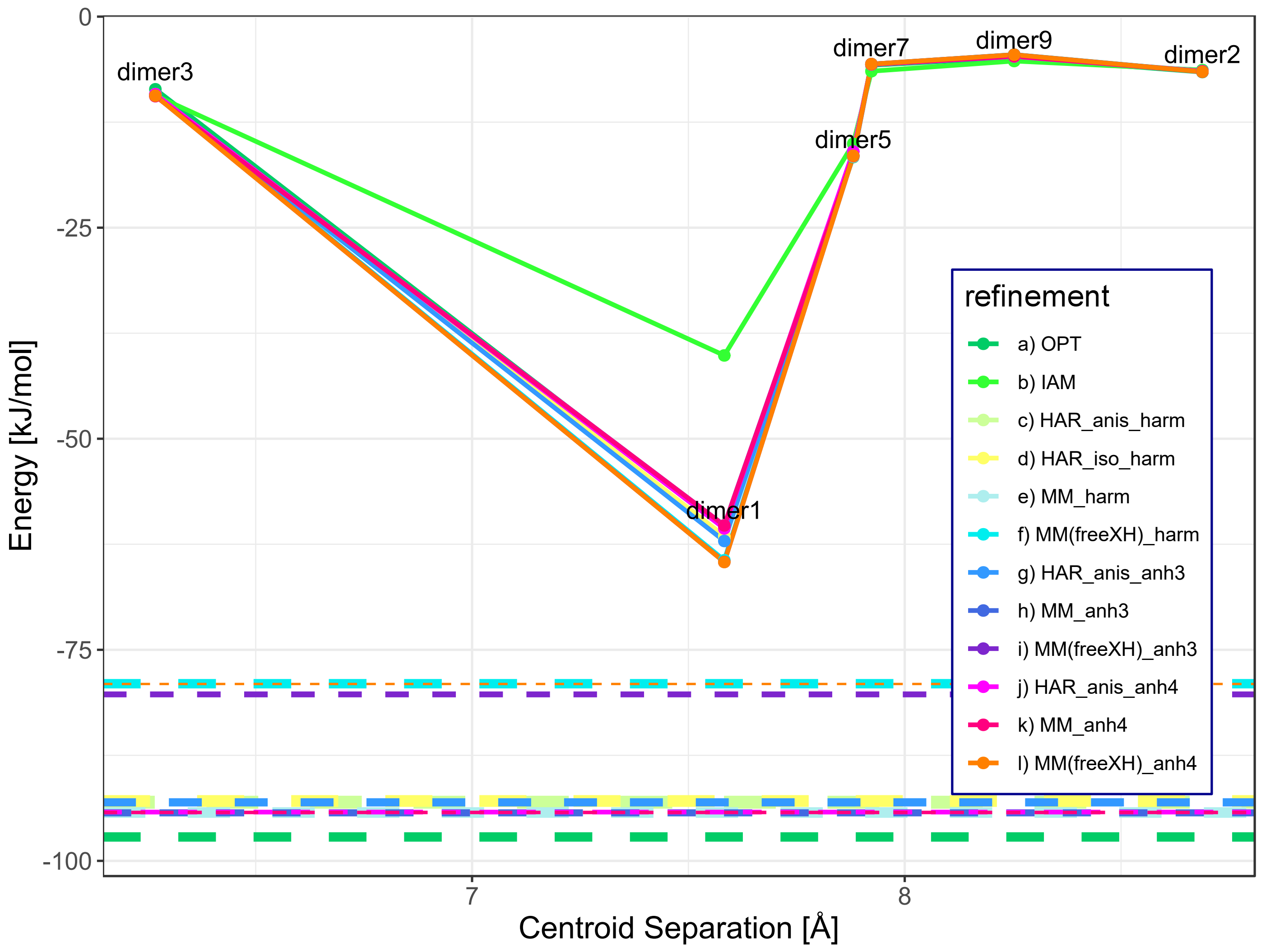

Figure 54: Dimer interaction energies (data points linked with solid lines) obtained for the structure of PT-2(Cl) refined with intensity cut-off ($|F| \geq 2\sigma(|F|)$) using various methods presented as a function of centroid separation of dimers. Cohesive energies are marked with dashed lines.

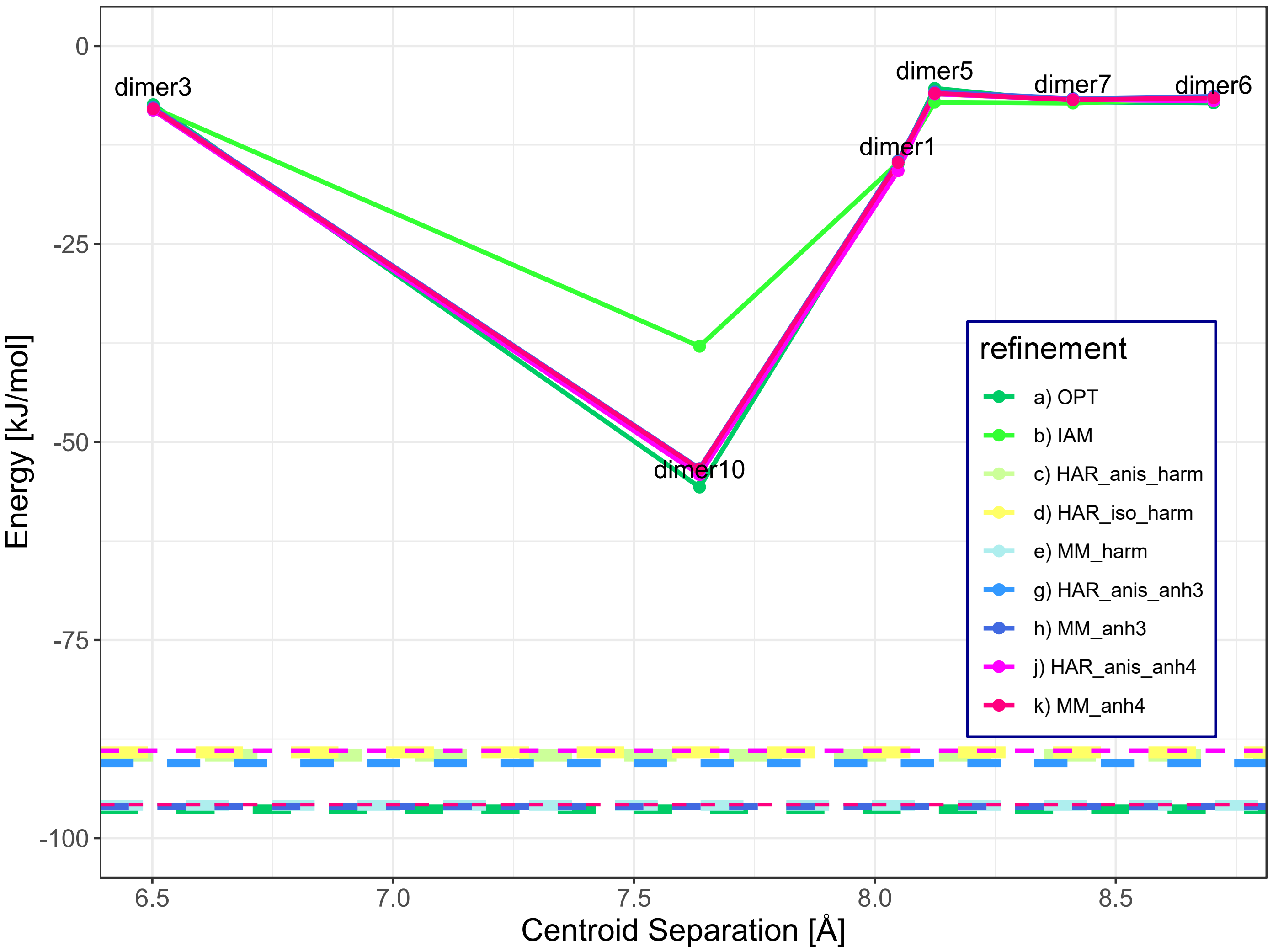

Figure 55: Dimer interaction energies (data points linked with solid lines) obtained for the structure of PT-8(Br) refined with intensity cut-off ($|F| \geq 2\sigma(|F|)$) using various methods presented as a function of centroid separation of dimers. Cohesive energies are marked with dashed lines.

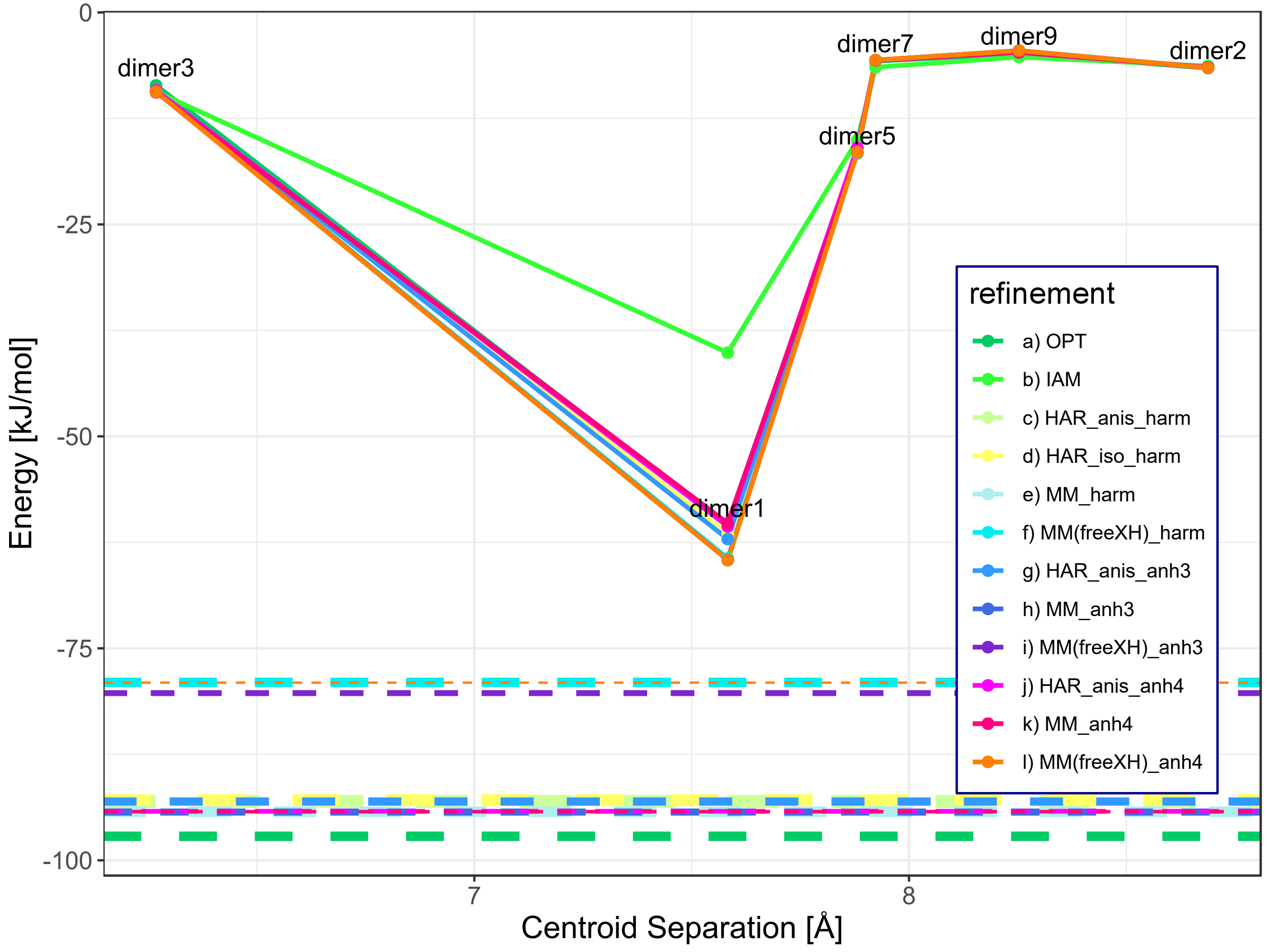

Figure 56: Dimer interaction energies (data points linked with solid lines) obtained for the structure of PT-10(I) refined with intensity cut-off ($|F| \geq 2\sigma(|F|)$) using various methods presented as a function of centroid separation of dimers. Cohesive energies are marked with dashed lines.

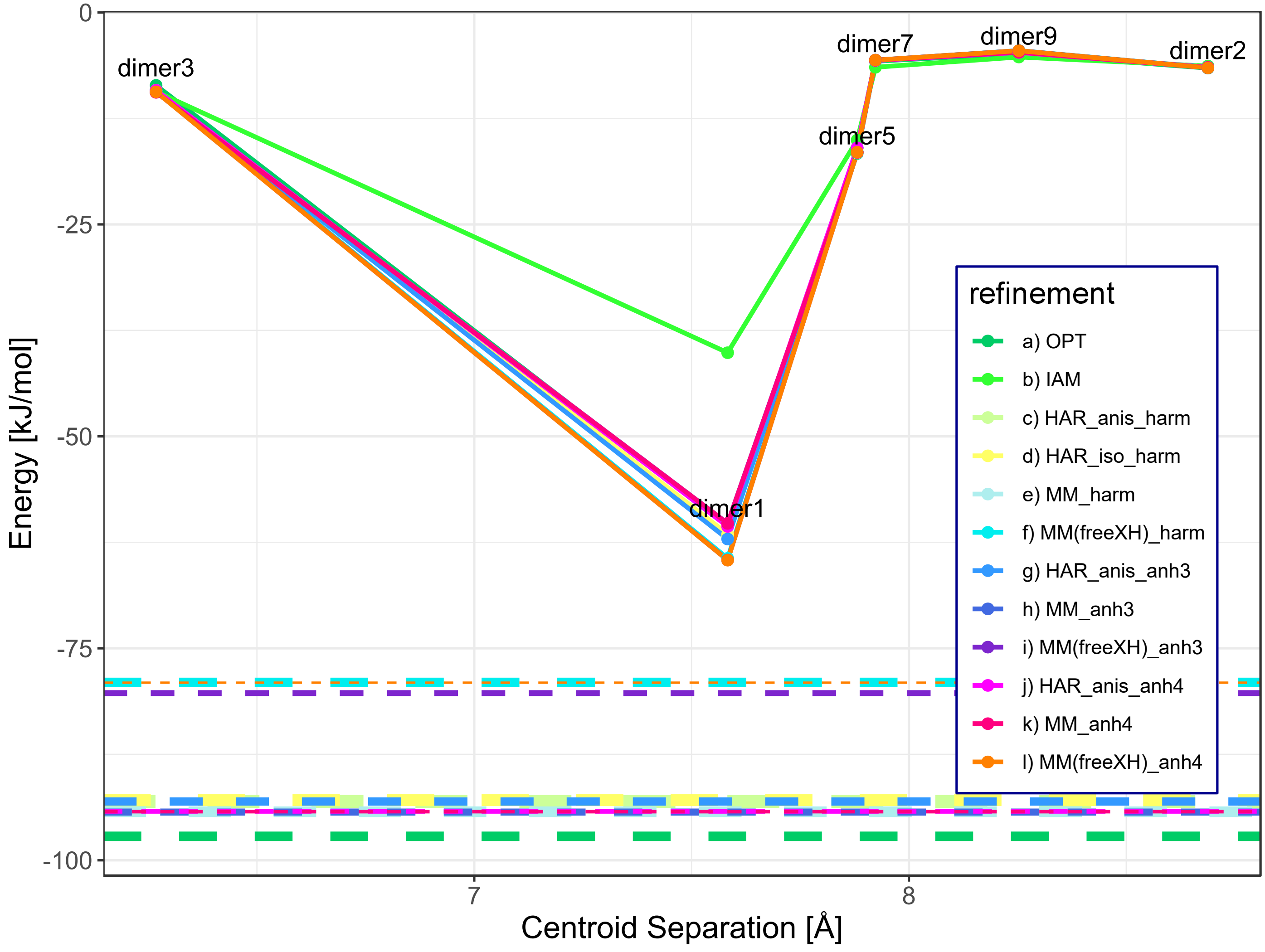

Figure 57: Dimer interaction energies (data points linked with solid lines) obtained for the structure of PT-11(S) refined with intensity cut-off ($|F| \geq 2\sigma(|F|)$) using various methods presented as a function of centroid separation of dimers. Cohesive energies are marked with dashed lines.

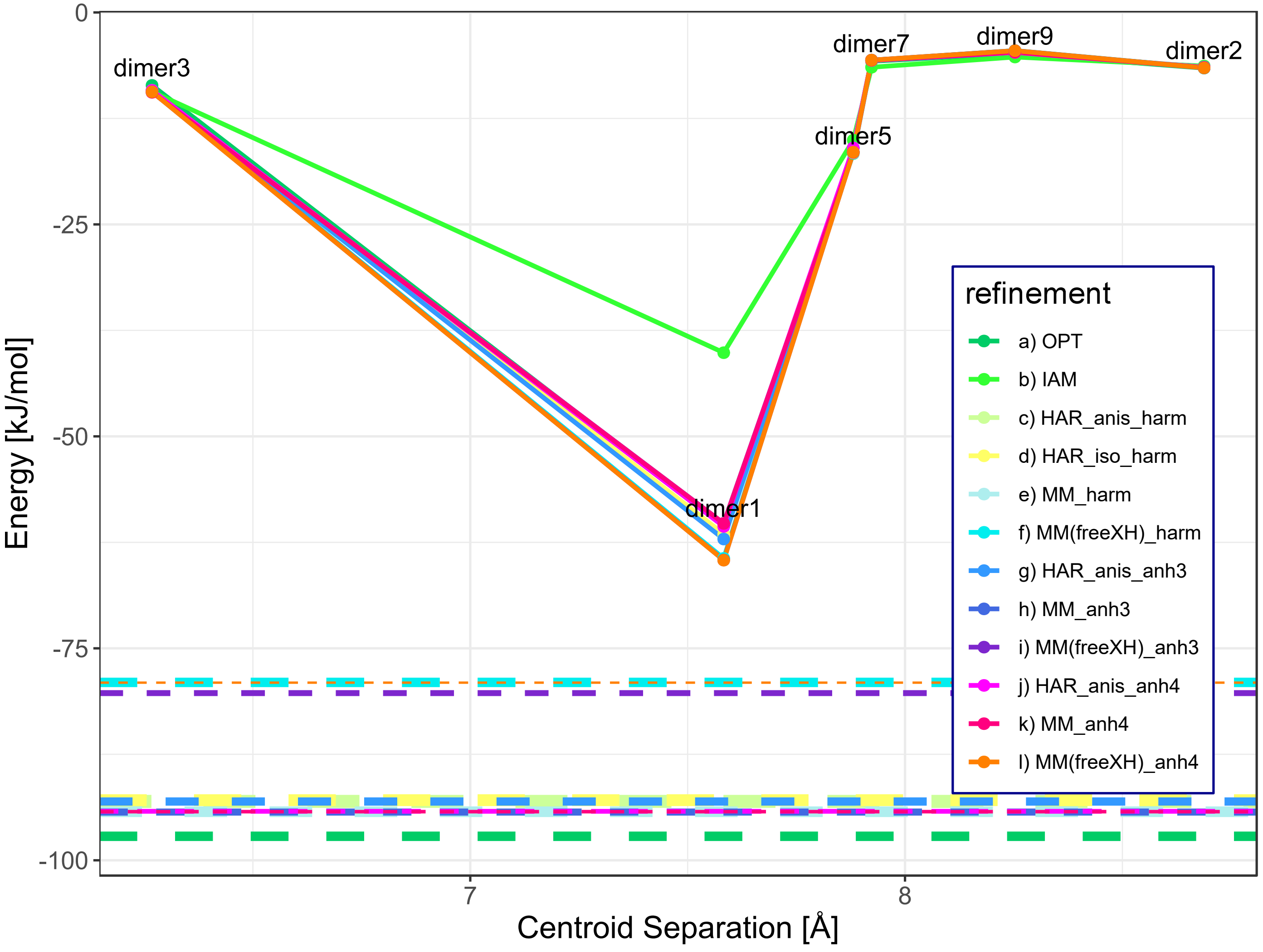

Figure 58: Dimer interaction energies (data points linked with solid lines) obtained for the structure of PT-2(Cl) refined without intensity cut-off using various methods presented as a function of centroid separation of dimers. Cohesive energies are marked with dashed lines.

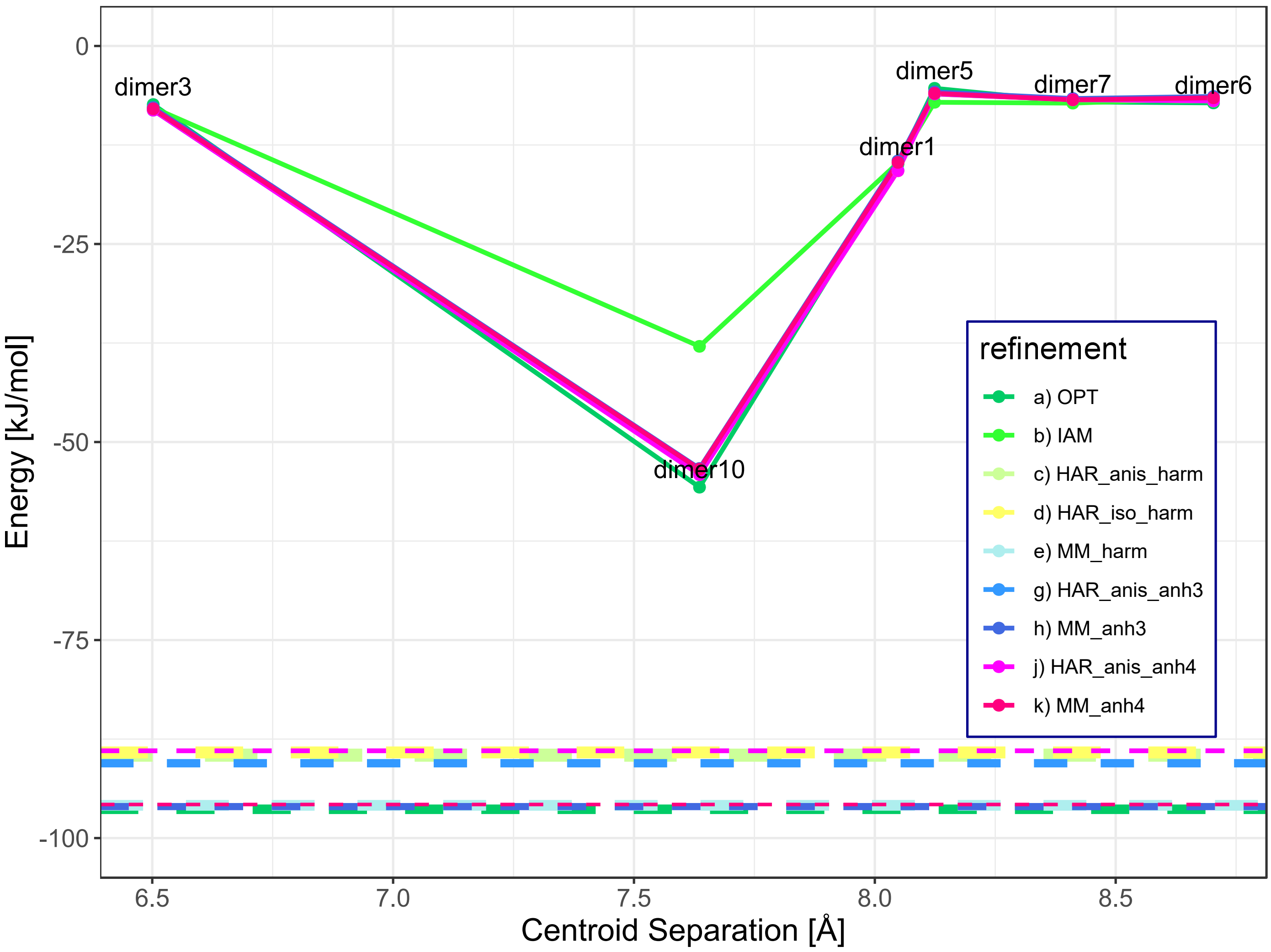

Figure 59: Dimer interaction energies (data points linked with solid lines) obtained for the structure of PT-8(Br) refined without intensity cut-off using various methods presented as a function of centroid separation of dimers. Cohesive energies are marked with dashed lines.

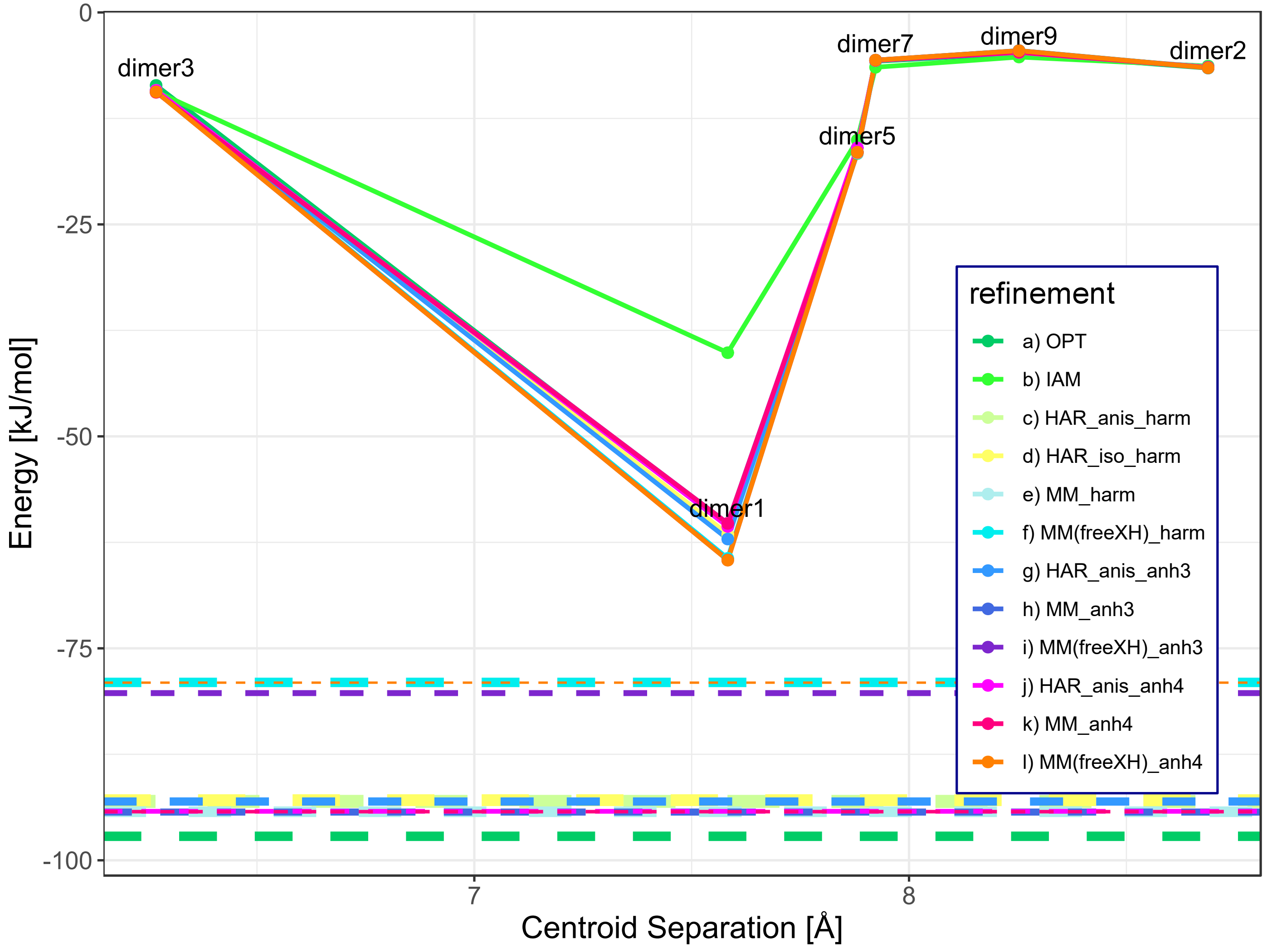

Figure 60: Dimer interaction energies (data points linked with solid lines) obtained for the structure of PT-10(I) refined without intensity cut-off using various methods presented as a function of centroid separation of dimers. Cohesive energies are marked with dashed lines.

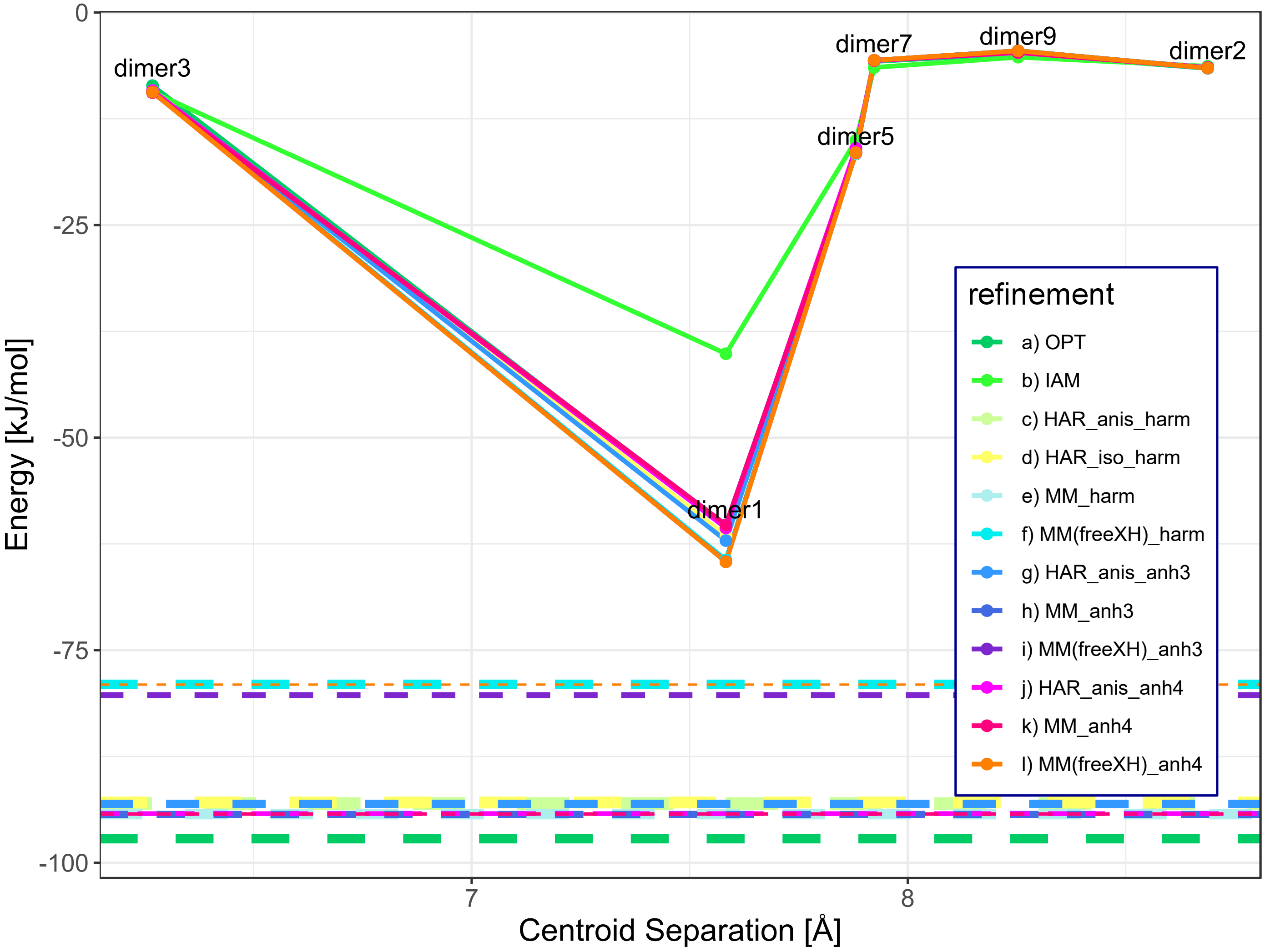

Figure 61: Dimer interaction energies (data points linked with solid lines) obtained for the structure of PT-11(S) refined without intensity cut-off using various methods presented as a function of centroid separation of dimers. Cohesive energies are marked with dashed lines.

8 Probability density function

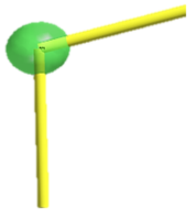

(a) HAR, anharmonic n=3, all reflections

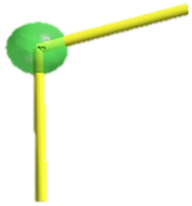

(b) HAR, anharmonic n=4, all reflections

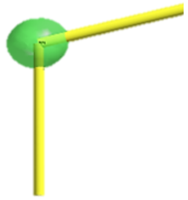

(c) HAR, anharmonic n=3, $|F| \geq 2\sigma(|F|)$

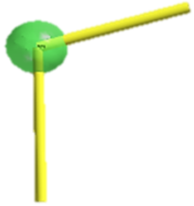

(d) HAR, anharmonic n=4, $|F| \geq 2\sigma(|F|)$

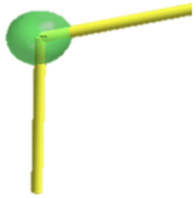

(e) MM, anharmonic n=3, all reflections

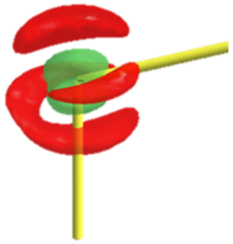

(f) MM, anharmonic n=4, all reflections

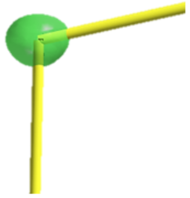

(g) MM, anharmonic n=3, $|F| \geq 2\sigma(|F|)$

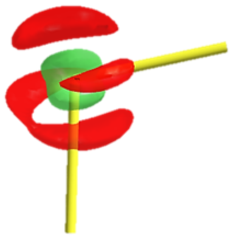

(h) MM, anharmonic n=4, $|F| \geq 2\sigma(|F|)$

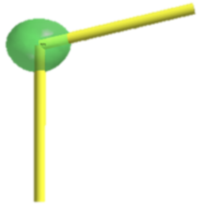

(i) MM(freeXH), anharmonic n=3, all reflections

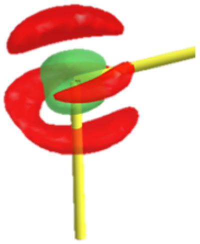

(j) MM(freeXH), anharmonic n=4, all reflections

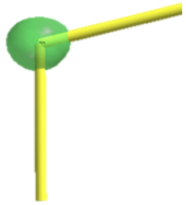

(k) MM(freeXH), anharmonic n=3, $|F| \geq 2\sigma(|F|)$

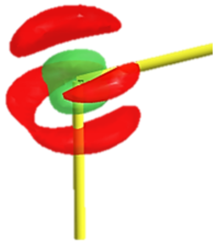

(l) MM(freeXH), anharmonic n=4, $|F| \geq 2\sigma(|F|)$

Figure 62: Probability density function of the S atom in PT-11(S-Ph). Probability contour level: 50%. Colours: green - positive, red – negative.

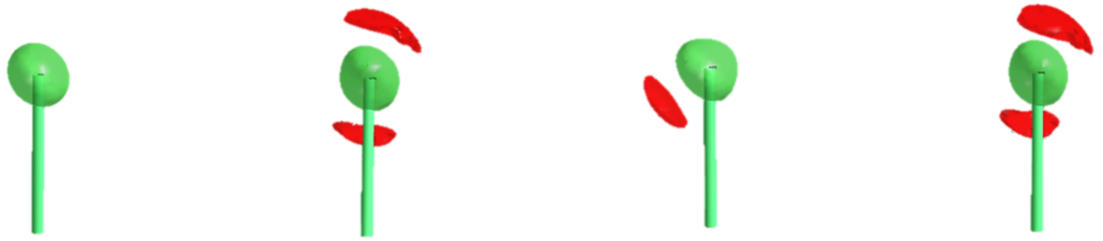

(a) HAR, anharmonic n=3, all reflections

(b) HAR, anharmonic n=4, all reflections

(c) HAR, anharmonic n=3, $|F| \geq 2\sigma(|F|)$

(d) HAR, anharmonic n=4, $|F| \geq 2\sigma(|F|)$

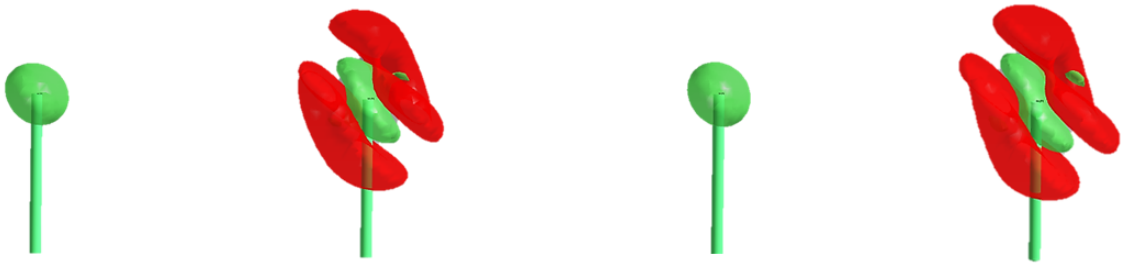

(e) MM, anharmonic n=3, all reflections

(f) MM, anharmonic n=4, all reflections

(g) MM, anharmonic n=3, $|F| \geq 2\sigma(|F|)$

(h) MM, anharmonic n=4, $|F| \geq 2\sigma(|F|)$

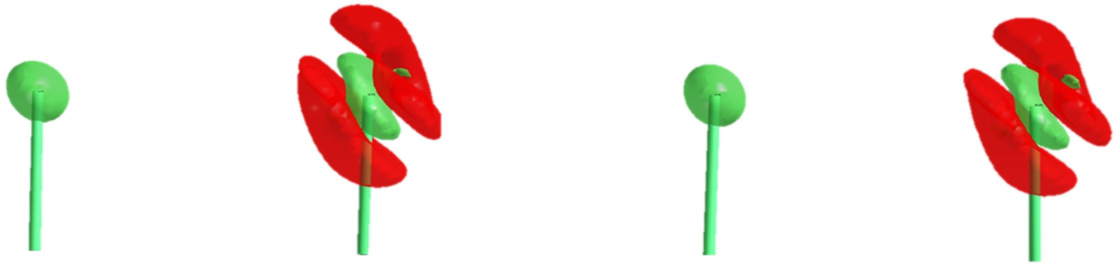

(i) MM(freeXH), anharmonic n=3, all reflections

(j) MM(freeXH), anharmonic n=4, all reflections

(k) MM(freeXH), anharmonic n=3, $|F| \geq 2\sigma(|F|)$

(l) MM(freeXH), anharmonic n=4, $|F| \geq 2\sigma(|F|)$

Figure 63: Probability density function of the Cl atom in PT-2(Cl). Probability contour level: 50%. Colours: green - positive, red – negative.

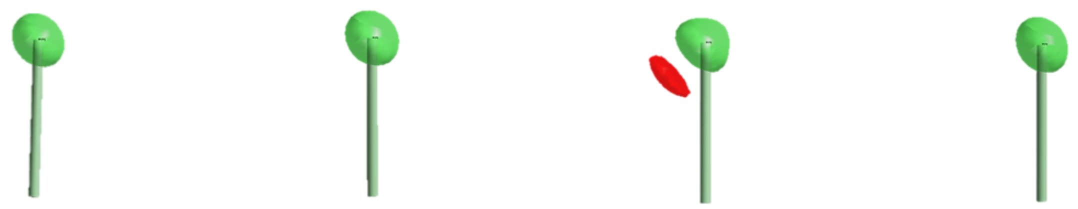

(a) HAR, anharmonic n=3, all reflections

(b) HAR, anharmonic n=4, all reflections

(c) HAR, anharmonic n=3, $|F| \geq 2\sigma(|F|)$

(d) HAR, anharmonic n=4, $|F| \geq 2\sigma(|F|)$

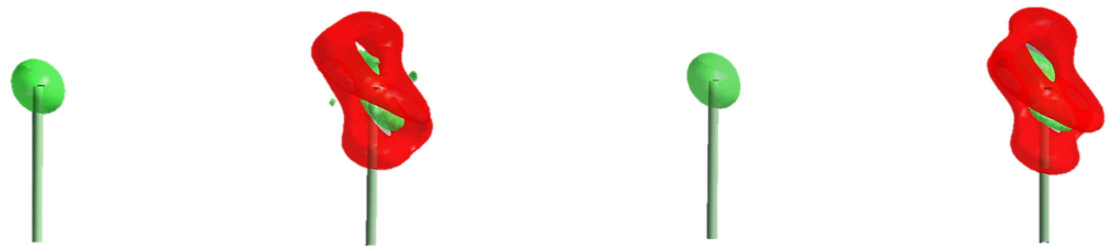

(e) MM, anharmonic n=3, all reflections

(f) MM, anharmonic n=4, all reflections

(g) MM, anharmonic n=3, $|F| \geq 2\sigma(|F|)$

(h) MM, anharmonic n=4, $|F| \geq 2\sigma(|F|)$

Figure 64: Probability density function of the Br atom in PT-8(Br). Probability contour level: 50%. Colours: green - positive, red – negative.

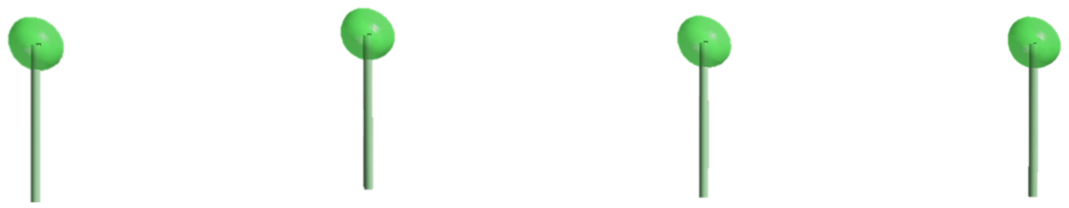

(a) HAR, anharmonic n=3, all reflections

(b) HAR, anharmonic n=4, all reflections

(c) HAR, anharmonic n=3, $|F| \geq 2\sigma(|F|)$

(d) HAR, anharmonic n=4, $|F| \geq 2\sigma(|F|)$

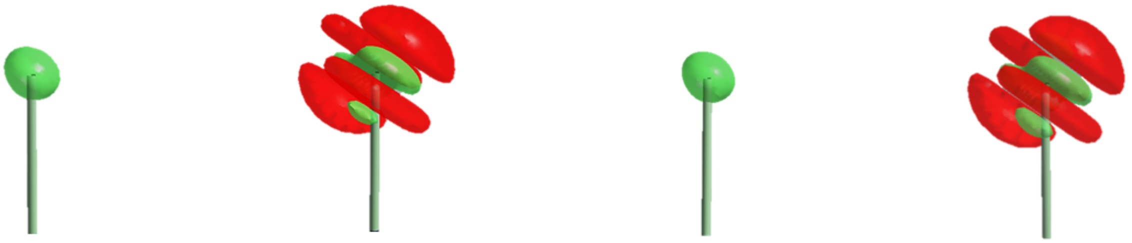

(e) MM, anharmonic n=3, all reflections

(f) MM, anharmonic n=4, all reflections

(g) MM, anharmonic n=3, $|F| \geq 2\sigma(|F|)$

(h) MM, anharmonic n=4, $|F| \geq 2\sigma(|F|)$

Figure 65: Probability density function of the I atom in PT-10(I). Probability contour level: 50%. Colours: green - positive, red – negative.
